# Interaction with IGF1 overrides ANXA2-mediated anti-inflammatory functions of IGFBP5 *in vivo*

**DOI:** 10.3389/fimmu.2024.1539317

**Published:** 2025-01-10

**Authors:** Yan Fan, Yi-Jin Wu, Kai Guo, Xia-Qing Zhou, Abulizi Abulaiti, Opeyemi Joshua Olatunji, Cong-Lan Ji, Jian Zuo

**Affiliations:** ^1^ Xin’an Medicine Research Center, The First Affiliated Hospital of Wannan Medical College (Yijishan Hospital), Wuhu, China; ^2^ Department of Pharmacy, The Second Affiliated Hospital of Wannan Medical College, Wuhu, China; ^3^ Department of Pharmacology, West China School of Pharmacy, Sichuan University, Chengdu, China; ^4^ Institute of Traditional Chinese Medicine and Ethnic Medicine, Uyghur Medicine Hospital of Akesu Prefecture, Akesu, Xinjiang, China; ^5^ African Genome Center, Mohammed VI Polytechnic University, Ben Guerir, Morocco; ^6^ School of Pharmacy, Anhui College of Traditional Chinese Medicine, Wuhu, China

**Keywords:** NF-κB, IGF1/IGF1R, ANXA2, rheumatoid arthritis, acute lung injury

## Abstract

**Background:**

*IGFBP5* is a differentially expressed gene (DEG) between M1 and M2 macrophages. This study explained why it causes opposite effects in different circumstances.

**Methods:**

Gene expression profiles of various cell subsets were compared by mining a public database. THP-1 cells were treated by siRNAs, recombinant IGFBP5, lipopolysaccharide (LPS), picropodophyllin, IGF1 or the combinations. Clinical implication of IGFBP5 changes was investigated using rheumatoid arthritis (RA) and acute lung injury (ALI) models. IGFBP5-bound and differential proteins were identified by Liquid Chromatography Mass Spectrometry method.

**Results:**

IGFBP5 situated in the center of a network constructed by the DEGs of M0 and M1/2 macrophages. Its expression negatively correlated to inflammation *in vitro*. When IGFBP5 was silenced, monocytes released more IL-1β and IL-6. NF-κB downstream proteins were overexpressed. IGFBP5 interacted with ANXA2 directly. In ANXA2-silenced cells, it showed no anti-inflammatory effect. Monocytes of adjuvant-induced arthritis rats and RA patients expressed less IGFBP5 than normal controls, but its blood levels increased significantly. Adipocytes secreted large amounts of IGFBP5. This secretion was reinforced by the above sera. IGFBP5 decreased in ALI mice’s blood, while its supplement exacerbated inflammation. By binding to IGF1, IGFBP5 prevented its interaction with IGF1R. An IGF1R inhibitor picropodophyllin antagonized functions of IGF1/IGF1R too, but didn’t reinforce the effects of IGFBP5.

**Conclusion:**

IGFBP5 eases inflammation by interacting with ANXA2, an activator of NF-κB; as an antagonist of IGF1/IGF1R, IGFBP5 may disrupt immune homeostasis *in vivo*, due to impairment of the latter’s anti-inflammatory functions; excessive IGFBP from adipocytes would be a pathogenic factor in certain diseases.

## 1 Introduction

The innate immune system is rapidly activated after infection, leading to elimination of pathogens and providing critical danger signals to the adaptive immune system (1). This evolutionary mechanism ensures both rapid and long-lasting defense functions. The innate immunity is essential for mammalian survival. Its compromise will result in devastating conditions, like severe infection and cancer (2). But its hyper-activation can also trigger unfavorable consequences (1, 2). Monocytes and macrophages are key components of the innate immune system. They have the similar immune functions. The majority of macrophages are replenished by circulating monocytes (3). There are typically two functional subsets: classically activated (M1) and alternatively activated (M2) monocytes/macrophages, which govern inflammatory defense and tissue repair, respectively (3, 4). Comparatively, M1 subset is more attractive in researches because of its great contribution to a broad spectrum of diseases, such as rheumatoid arthritis (RA), hepatitis, influenza and metabolic disorders. Thus, elucidating the mechanisms driving their unbalanced polarization has great clinical significance.

According to the current understanding, these cells are inflammatorily activated by pattern recognition receptors, represented by the TLR family (2). Pathogen-derived endotoxins are their typical ligands. For instance, lipopolysaccharide (LPS) is a potent agonist of TLR4 (5). This mechanism allows the cells to sense infection risks quickly. Involvements in chronic diseases suggest that their status is regulated by some other mediators too. Abnormal immune-metabolism interaction has been recognized as an important aspect of chronic diseases. Accordingly, metabolic regulators are emerging as key players in the immune system (6, 7). Since glucose metabolism is essential for all living cells, its alteration is especially notable under inflammation, with glycolysis acceleration as a hallmark. In this context, a great attention is paid on those signals controlling glucose metabolism (6–8). We had made some efforts in this aspect, and revealed a glycolytic enzyme TPI1 as a diagnostic indicator of RA (9).

In the current study, we discovered *IGFBP5* as a gene that distinguishes M0 and M1/2 monocytes/macrophages. It is a regulator of glucose metabolism. By binding to IGF1, IGFBP5 prevents its interaction with IGF1R. From this sense, it is a switch for this metabolic pathway (10). But its role in the immune system is unknown. Herein, we focused on this issue. IGFBP5 exhibited both inflammatory and anti-inflammatory properties in the experiments. We assumed that IGFBP5 links to diversified pathways, which have the totally different immune functions; the exact outcome from IGFBP5 stimulus is decided by the specific environment, when certain one signal branch is predominant. The hypothesis was validated. Furthermore, this study underscored the pathogenic role of IGFBP5 by acting as an antagonist of IGF1/IGF1R pathway.

## 2 Materials and methods

### 2.1 Chemicals and reagents

Enzyme linked immunosorbent assay (ELISA) kits were obtained from Multi-Science (Hangzhou, Zhejiang, China). Primary antibodies were supplied by ABclonal (Wuhan, Hubei, China), while horseradish peroxidase (HRP)/fluorescein-conjugated secondary antibodies were procured from Boster (Wuhan, Hubei, China). Cell culture reagents were supplied by Thermo Fisher Scientific (Rockford, IL, USA). Lipopolysaccharide (LPS) from *Escherichia coli* 055 was purchased from Sigma-Aldrich (St. Louis, MO, USA). Recombinant human proteins IGFBP5 and IGF1 were sourced from Fine Biotech (Wuhan, Hubei, China). Other reagents were all obtained from Jiancheng Bioengineering Institute (Nanjing, Jiangsu, China). Ultrapure water was produced by using a Milli-Q system (Millipore, Bedford, MA, USA).

### 2.2 Gene expression comparison of M1 and M2 macrophages

The dataset GSE146895 was downloaded from the Gene Expression Omnibus (GEO) database (https://www.ncbi.nlm.nih.gov/), which includes the normalized expression matrices of M0, M1 and M2 macrophages, with three samples in each cell subset. To identify the regulators involved in macrophage polarization, differentially expressed genes (DEGs) between M0 and M1/M2 macrophages were screened out (fold change > 2, p < 0.05). This analysis was performed based on R software (version 4.1.1) with a “Limma” package (https://cran.r-project.org/).

### 2.3 Construction of protein-protein interaction network

The Search Tool for Retrieving Interacting Genes (STRING) is an online tool for PPI network information assessment (http://www.string-db.org) (11). We used this tool to construct a PPI network based on the above DEGs. Only interaction with a combined score over 0.4 was illustrated in the diagram. The visualization of PPI network was achieved using Cytoscape software 3.10.1. A built-in function for molecular complex detection was used to identify modules of the PPI network. We set the parameters as follows: degree cutoff, 2; node score cutoff, 0.2; K-core, 2; max depth, 100.

### 2.4 Functional validation of IGFBP5 in monocytes

THP-1 cells were used for functional validation, which grew in complete RPMI 1640 medium under standard conditions (37°C, humidified atmosphere with 5% CO_2_). A portion of cells grown in 6-well plates were stimulated with LPS (500 ng/ml) or in the combination with IGFBP5 (50 ng/ml) for 6 h. The medium was used for ELISA test of IGFBP5, and the harvested cells were analyzed by western blot (WB) to assess expression of immune regulators and IGFBP5. Subsequently, IGFBP5 expression was silenced in normal THP-1 cells by siRNA, which was synthesized by Genepharma Co., Ltd. (Shanghai, China) with the sequences in **Supplementary Table S1**. The cells were serum-starved for 12 h, before being cultured in the medium containing a transfection mixture (200 μl Opti-MEM + 5 μl Lipofectamine 3000) and siRNA solution (5 μl siRNA in 200 μl Opti-MEM). Four hours later, the supernatant was replaced with complete medium, and the cells were incubated for an additional 48 h to complete the transfection. The medium was collected for ELISA analysis of IL-1β and IL-6. In the flow cytometry (FCM) analyses, the cells were first incubated by PE-CD86 antibody, followed by fixation, permeabilization and APC-CD206 antibody staining procedures. Then, they were subjected to a flow cytometer (FC500, Beckman). CD86 and CD206 served as the marker for M1 and M2 cell subsets, respectively.

### 2.5 Label-free proteomics study

This service was provided by Biotree Tech (Shanghai, China). Proteins were extracted by lysis buffer with the aid of sonication at 4°C, and quantified by a kit. The samples were diluted by acetone, kept overnight at -20°C, and centrifuged at 13,000 rpm for 10 min. The precipitation was washed with 80% acetone, and resolved. Dithiothreitol was added to achieve a concentration of 5 mM. The solution was incubated at 55°C for 20 min. The product reacted with iodoacetamide (15 mM) in the dark for 30 min. Subsequently, the proteins were digested with trypsin at 37°C overnight. Afterwards, the reaction was ceased, and the mixture was centrifuged at 13,000 rpm for 10 min. The supernatant was taken as polypeptide samples. Desalting was performed by using C_18_ mini tubes. The eluents were collected, and dried at 4°C in vacuum.

Total peptides (2 μg) in samples were separated by an EASY-nLC1200 UHPLC system, and detected by a Q Exactive HFX Orbitrap detector with an electrospray ion source (Thermo Fisher Scientific). Separation was achieved on a column (100 μm × 15 cm, Reprosil-Pur 120 C18AQ, 1.9 μm, Dr. Maisch). Aqueous solution 0.1% formic acid + 2% acetonitrile and 80% acetonitrile + 0.1% formic acid served as the phase A and B, respectively. A 120 min gradient elution program running at 300 nl/min was adopted: 25% (phase B) for 2 min, 5-22% for 88 min, 22-45% for 26 min, 45-95% for 2 min, 95% for 2 min. Data dependent acquisition was performed at the profile and positive mode. The resolution for MS1 and MS2 detection was set at 120,000 and 15,000, respectively. Top 20 abundant ions were fragmented with the normalized collision energy of 27%. The isolation window was set at 1.2 m/z. The peaks with charge(s) of only one or over 6 were excluded in the following analyses.

Data mining was achieved by Proteome Discoverer (Version 2.4.0.305) and the built-in Sequest HT search engine. Protein signals in the raw data were identified by searching a UniProt FASTA database (uniprot-Homo sapiens-9606-2022-11). In addition, proteins were quantified in samples based on the signal intensity of unique peptides and razor peptides. Differentially expressed proteins (DEPs) were visualized in a heatmap and used for clustering analysis. The DEPs were then mapped onto Gene Ontology (GO) terms as well as Kyoto Encyclopedia of Genes and Genomes (KEGG) pathways. Key nodes in the PPI network were highlighted in a quantification diagram.

### 2.6 Investigation of IGFBP5 subcellular distribution

For this immunofluorescence (IF) experiment, untreated and LPS-stimulated THP-1 cells were fixed in 4% formaldehyde for 30 min, permeabilized by 0.3% Triton X-100, and then incubated with antigen repair solution for 10 min. Subsequently, these cells were blocked with 5% bovine serum albumin solution in an incubator at 37°C for 1 h. After that, they were incubated overnight at 4°C with diluted anti-IGFBP5 (1:100) or IGF1 (1:200) antibody solutions, followed by treatments with fluorescein-conjugated secondary antibodies (1:500) at room temperature for 1 h. Finally, the cellular nuclei were stained with 4’,6-diamidino-2-phenylindole (DAPI). These cells were observed using a SP8 LIGHTNING confocal microscope (Leica).

### 2.7 IGFBP5-bound proteins identification and co-immunoprecipitation

Cells were lysed in NP-40 lysis buffer on ice. After centrifugation at 12,000 rpm for 5 min, the supernatant was collected. An antibody specific for the target protein was added into the cell lysate, and incubated overnight at 4°C under occasionally shaking. On the second day, pre-washed protein A/G magnetic beads were incubated with the sample for 1.5 h. These beads were retrieved in a magnetic field, and washed 5 times with NP-40 buffer. Proteins binding to the beads were then eluted, and boiled at 95°C in diluted loading buffer. In normal co-IP assays, samples obtained before and after the above steps were subjected to normal WB assays.

To detected IGFBP5-bound proteins, the eluate from the beads was separated by electrophoresis. The separation gels were stained with Coomassie Brilliant Blue. The bands exhibiting a distinct density difference between control and IGFBP5-IP sample were excised and cut into pieces (1 mm). The gel fragments were decolorized and kept in acetonitrile for 10 min. Thereafter, they were treated with dithiothreitol and iodoacetamide, followed by digestion, desalting and Liquid Chromatography Mass Spectrometry (LC-MS) detection, as those described in the proteomics study. Proteins exclusively detected in IGFBP5-IP sample were subjected to PPI analysis. The most abundant protein in center of the network was selected for further experiments.

### 2.8 Discovery of IGFBP5/ANXA2 signal transduction mechanism

We cultured normal THP-1 cells beforehand. The protein samples collected from their lysates before and after ANXA2/IGFBP5 co-IP were analyzed by WB. In subsequent experiments, IGFBP5 was silenced in some of the cells, or THP-1 cells were treated with LPS (500 ng/ml) combined with IGFBP5 (50 ng/ml) for 6 h. co-IP assays were performed once again to assess the binding status of ANXA2 and TLR4 under these differed conditions. Afterwards, the experiment involving LPS + IGFBP5 treatments was repeated. The medium and cells were collected for ELISA and WB experiments, respectively. Based on the results, the experiment was optimized. The concentrations of LPS and IGFBP5 were increased by 2-fold. The treatment duration was extended to 12 h. The replicate experiments used both normal and ANXA2-silenced THP-1 cells, and the medium was collected to measure IL-1β levels.

For WB assays, total cellular proteins were extracted using RIPA buffer. Protein samples were separated by electrophoresis, and transferred onto PVDF membranes. The membranes were then sequentially incubated with skim milk, primary antibodies and HRP-conjugated secondary antibodies. Protein bands were visualized by using an enhanced chemiluminescence substrate solution.

### 2.9 Analyses of IGFBP5 levels in samples from rheumatic subjects

To clarify the role of IGFBP5 in inflammatory diseases, we measured its blood levels in adjuvant-induced arthritis (AIA) rats and RA patients. The samples were from our previous studies (12–14). Anticoagulated blood was centrifuged. The obtained plasma was used for ELISA assays. Red blood cells in the sediment were lysed. Protein and RNA in remaining white blood cells (WBCs) were extracted for WB and polymerase chain reaction (PCR) analyses, respectively. Additional blood from RA patients and healthy participants was processed to isolate monocytes by a commercial kit. IGFBP5 expression in these monocytes was analyzed by PCR method. Briefly, the samples were lysed in TRIzol reagent. Total RNA was extracted with chloroform, purified with isopropanol and ethanol, and used to synthesize cDNA. Quantitative PCR analysis was conducted on a Life Tech 7500 Real Time PCR detection system (Carlsbad). Those experiments involving human blood samples were approved by the Institutional Ethics Research Committee of Yijishan Hospital (No. 2022-20), and the written consent was obtained from all participants. Next, 3T3-L1 cells were induced to mature according to a reported method (9), and cultured with sera from the aforementioned rats and human volunteers for 12 h. The medium was then collected for ELISA analysis of IGFBP5. Some mature 3T3-L1 adipocytes were treated with IGFBP5 (100 ng/ml) for 12 h, and levels of TNF-α and IL-6 in the medium were detected using ELISA kits.

### 2.10 Investigation of the role of IGFBP5 in acute lung injury

*In vivo* experiments were performed on male C57 mice (8 weeks old, obtained from Tianqin Biotechnology, Changsha, Hunan, China). Mice were housed in groups of four per cage in a specific pathogen-free laboratory, and provided with commercial rodent chow and boiled tap water. After one week of acclimation, 18 mice were randomly divided into 3 groups: healthy, ALI, and ALI + IGFBP5. LPS-induced ALI progresses rapidly, and will eventually cause mortality. Hence, experiment time window is short. IGFBP5-brought consequences were uncertain. It would amplify experiment risks. By taking these risks into consideration, we pre-treated mice with IGFBP5 (10 μg/kg) for 3 days. Then, ALI and ALI + IGFBP5 groups received an intraperitoneal injection of LPS (5 mg/kg). This experiment design highlights importance of IGFBP5-related environment difference during ALI progress. Twelve hours after LPS administration, all mice were euthanized via an overdose injection of pentobarbital sodium. Blood and organs were collected. Lung weight was recorded to assess edema severity. Levels of IGFBP5 in plasma and tissue homogenates along with IL-6, IL-1β, and MCP-1 were determined by ELISA kits. Anticoagulated blood was used for FCM and complete blood count (CBC) analyses. Monocytes were identified by FITC-CD11b antibodies, and those expressing high levels of Ly6c were classified as classical monocytes. CBC was performed on a PE-6800 VIT blood cell counter (Pukang Biotech). Lungs were sectioned, and stained by hematoxylin and eosin (H&E) for histological examination with standard procedures (13). The animal experiments were approved by the Ethical Committee of Wannan Medical College (No. LLSC-2022-223).

### 2.11 Investigation on the relationship of IGFBP5 and IGF1/IGF1R pathway

THP-1 cells were treated with IGFBP5 protein at various concentrations for 12 h, and cell viability was assessed using Cell Counting Kit-8. In the subsequent experiment, THP-1 cells were stimulated with LPS (1 μg/ml). A subset was co-treated by IGFBP5 within the nontoxic concentration range. IL-1β levels in the medium were measured by ELISA kits. This experiment was repeated, and IGF1 was used instead of IGFBP5. Next, we separately silenced IGFBP5 and IGF1R in THP-1 cells. The cells and their culture medium were collected for WB and ELISA analyses, respectively. Afterwards, some LPS-primed THP-1 cells were treated with IGFBP5 in the presence of IGF1 or not. The resulting medium was analyzed for IL-1β levels, while IGF1 localization was examined by IF method. In addition, some LPS-stimulated THP-1 cells were treated with IGFBP5, IGF1, picropodophyllin (PPP, a selective IGF1R inhibitor) or their combinations. Levels of IL-1β and ARG-1 in the medium were measured by using the appropriate ELISA kits.

### 2.12 Statistical analysis

Sample size in experiments was calculated by G*power (www.psychologie.hhu.de). Results of WB and IF experiments were quantified by Image J (1.52a, NIH, Bethesda, MD). Data were presented in the form of mean and standard deviation. Shapiro-Wilk test and Bartlett’s test were adopted to check normality and homogeneity of variance, respectively. Statistical difference among/between groups was evaluated by one-way analysis of variance followed by Tukey’s *post hoc* test or t-test by the aid of GraphPad Prism 8.0 (Cary, NC).

## 3 Results

### 3.1 *IGFBP5* negatively regulates M1 polarization of monocytes/macrophages

**Figure 1:**
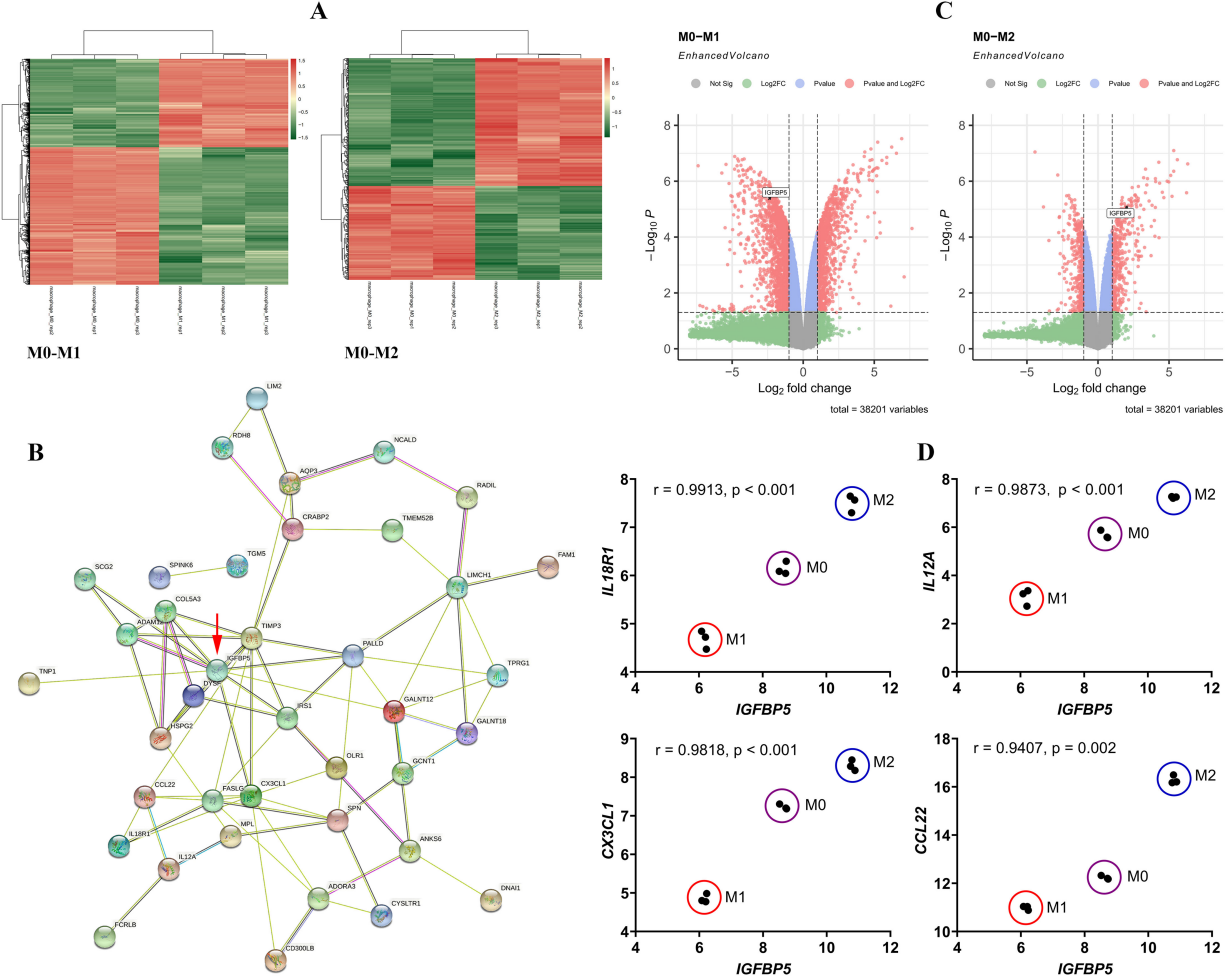
*IGFBP5* expression status differentiated M1 and M2 macrophages. **(A)** the DEGs between M0 and M1/M2 macrophage subsets; **(B)** a PPI network constructed by the DEGs; **(C)** *IGFBP5* expression levels in different macrophage subsets indicated in volcano diagrams; **(D)** the expression correlation between *IGFBP5* and representative immune genes in different macrophages.

Macrophages at different polarization states display distinct gene expression profiles. The DEGs between M0 and M1/M2 subsets are shown in **Figure 1A**, and we focused on those showing the opposite change trends between M1 and M2 subsets by taking M0 cells as the reference. A PPI network was constructed using these DEGs (**Figure 1B**). It reveals *IGFBP5* as a central node, a gene that is regulated by metabolic hormones. Its expression decreased in M1 subset and increased in M2 subset (**Figure 1C**). *IGFBP5* showed strong correlations with key immune genes such as *IL18R1*, *IL12A*, *CX3CL1*, and *CCL22*. Different macrophage subsets were clearly distinguishable in scatter plots illustrated by *IGFBP5* and these immune genes (**Figure 1D**).

**Figure 2:**
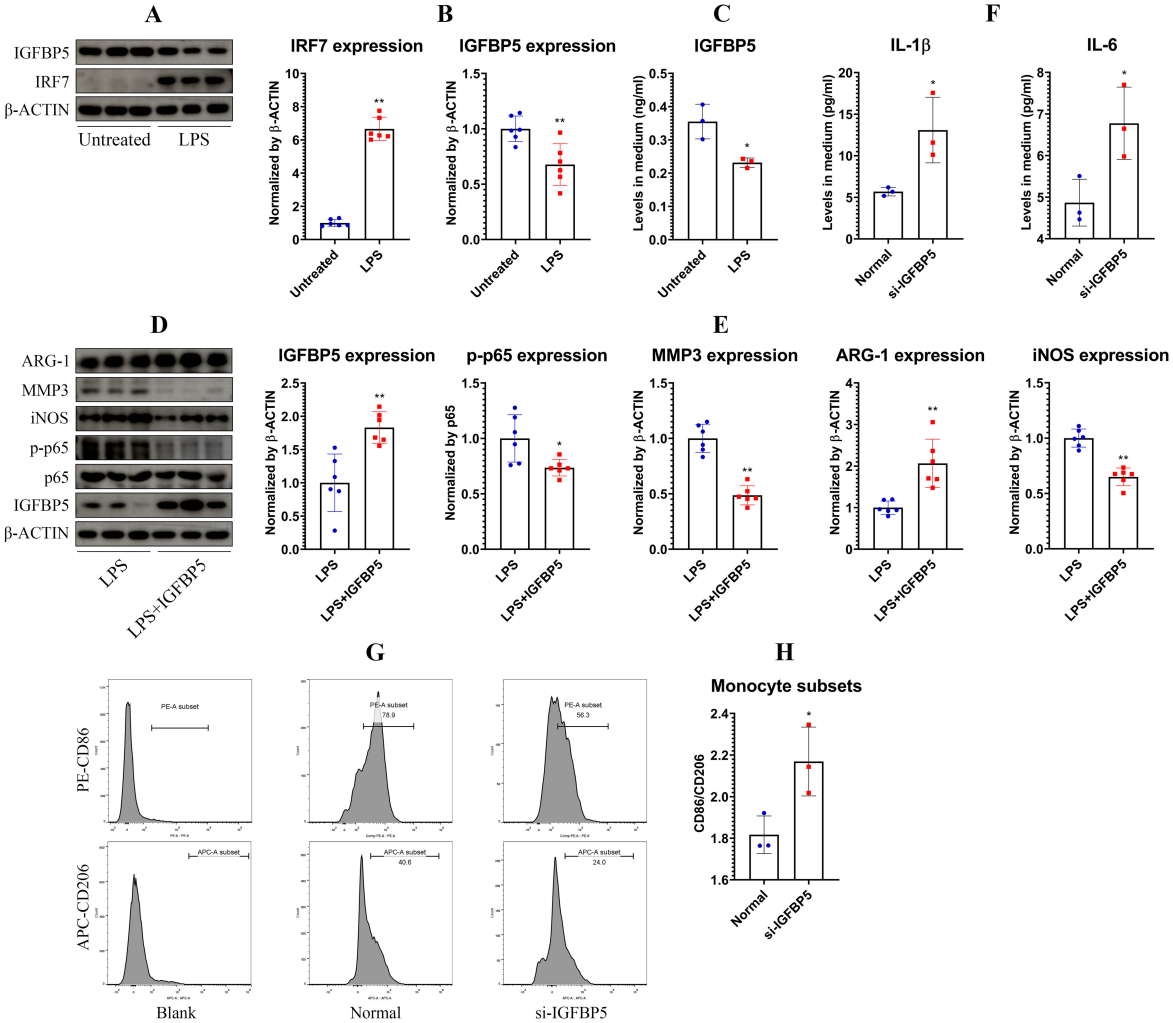
IGFBP5 played an anti-inflammatory role in monocytes. **(A)** expression of IGFBP5 and IRF7 in untreated and LPS-treated THP-1 cells; **(B)** quantification results of experiment A; **(C)** levels of IGFBP5 in the medium from assay A; **(D)** expression of immune indicators in untreated and IGFBP5-treated THP-1 cells; **(E)** quantification results of experiment D; **(F)** levels of IL-1β and IL-6 in the medium from normal and IGFBP5-silenced THP-1 cells; **(G)** FCM analysis of the cells from experiment F; **(H)** quantification results of experiment **(G)** Statistical significance: ^*^ *p* < 0.05 compared with normal or untreated cells.

LPS skewed THP-1 monocytes toward an inflammatory phenotype, indicated by up-regulation of IRF7 and concurrent down-regulation of IGFBP5 (**Figures 2A, B**). This change of IGFBP5 was confirmed by an ELISA test (**Figure 2C**). When monocytes were sufficiently activated by LPS, IGFBP5 treatment suppressed inflammation with the high efficiency. It suppressed expression of MMP3, iNOS and p-p65, while promoted expression of ARG-1 (**Figures 2D, E**). Conversely, IGFBP5 silencing led to increased secretion of IL-1β and IL-6 (**Figure 2F**). Meanwhile, the ratio of M1/M2 cell subset was increased (**Figures 2G, H**). In this experiment, IGFBP5 was silenced in normal untreated THP-1monocytes. The collective evidence demonstrates that IGFBP5 basically plays an anti-inflammatory effect *in vivo* regardless of cellular immune status difference.

**Figure 3:**
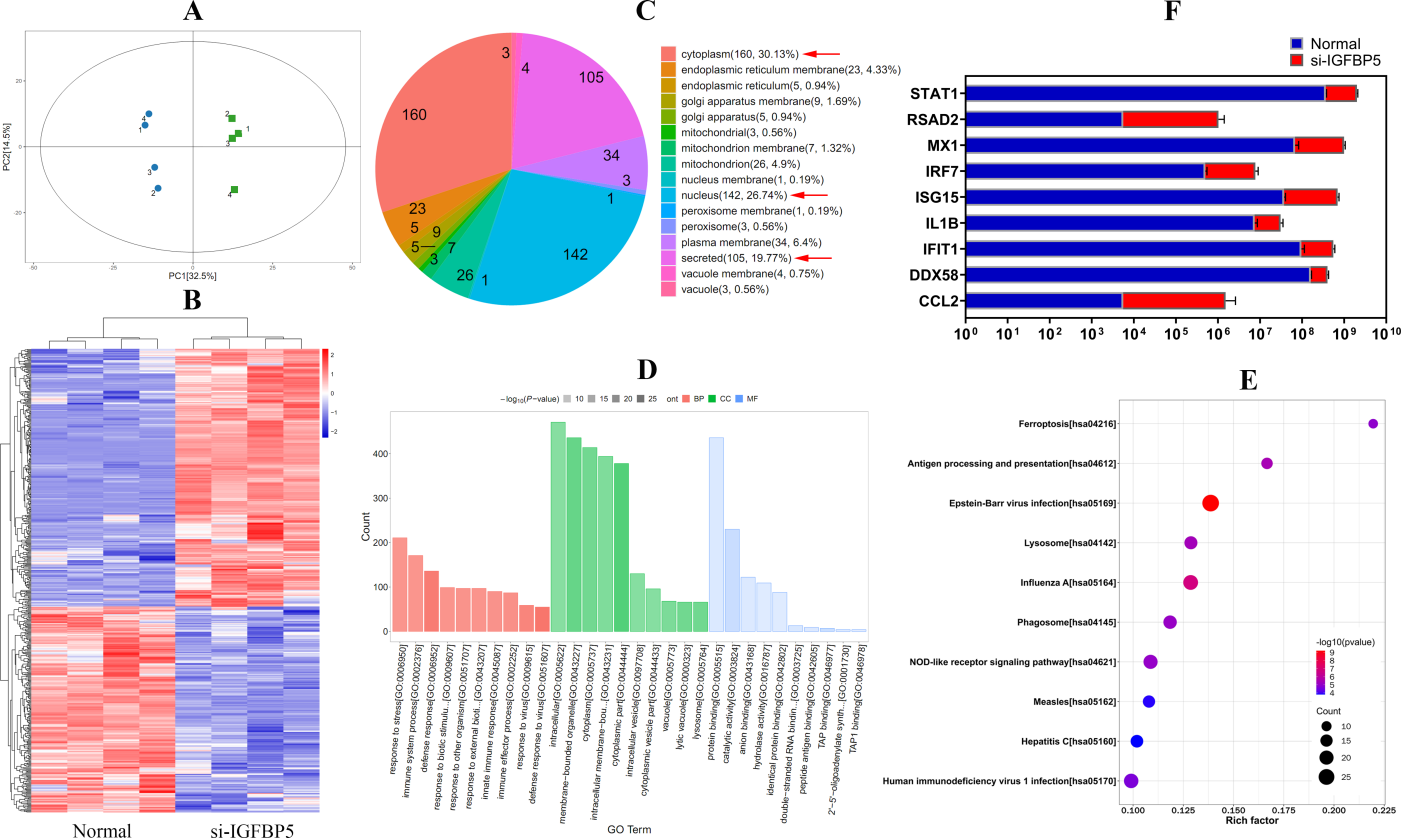
IGFBP5-silencing induced inflammatory polarization of monocytes. **(A)** the differed protein expression profiles between IGFBP5-silenced and normal THP-1 cells; **(B)** results of clustering analysis using DEPs between the two cell sets; **(C)** subcellular localization of the DEPs; **(D)** results of GO pathway enrichment; **(E)** results of KEGG pathway enrichment; **(F)** relative expression levels of node proteins in a PPI network constructed by the DEPs.

Principal component analysis reveals distinct protein expression profiles between normal and IGFBP5-silenced THP-1 cells (**Figure 3A**). All the samples were correctly assigned into different groups in the clustering analysis (**Figure 3B**). Most DEPs were localized in the cytoplasm and nucleus, with a smaller portion belonging to secreted proteins (**Figure 3C**). It suggests that IGFBP5 potently influences intracellular signaling and cytokine secretion. GO enrichment exhibits that IGFBP5 is basically an immune regulator. IGFBP5 silencing affected many immune pathways in monocytes (**Figure 3D**). KEGG enrichment provides more insights into immune-regulatory effects of IGFBP5 (**Figure 3E**). Except ferroptosis, all those identified pathways are involved in immune regulation. NF-κB ranked as the 12^th^ most enriched pathway (**Supplementary Table S2**). It is a common downstream target of many immune pathways. This finding underscores broader significance of IGFBP5 in the immune system. Expression of node proteins from the PPI network is shown in **Figure 3F**. They are all with inflammatory properties and up-regulated, when IGFBP5 was silenced. It confirms the anti-inflammatory role of IGFBP5 in monocytes.

### 3.2 IGFBP5 inhibits inflammation in monocytes by binding to ANXA2

**Figure 4:**
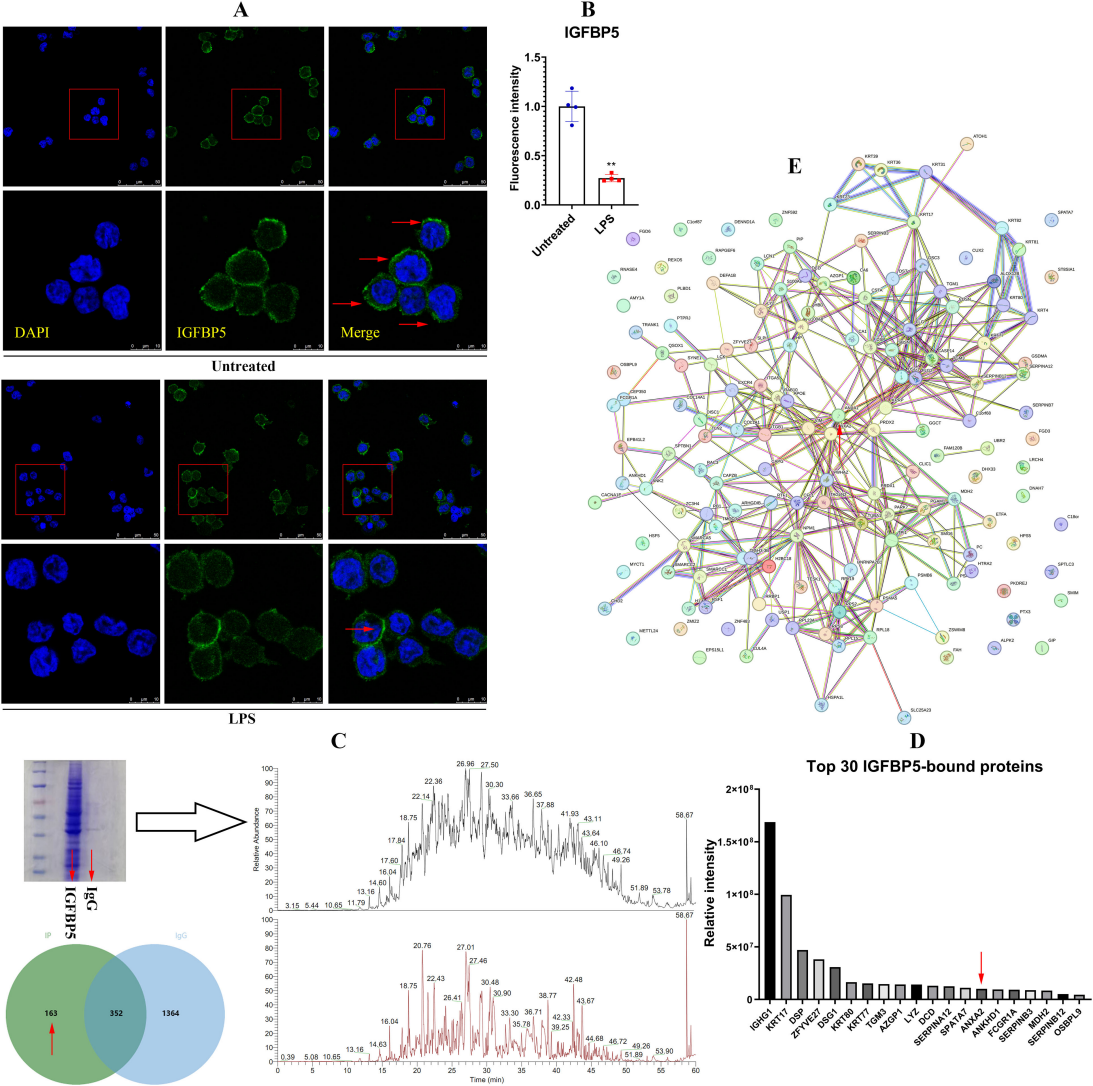
IGFBP5 bound to ANXA2. **(A)** IGFBP5 distribution in LPS-stimulated and untreated THP-1 cells; **(B)** quantification results of experiment A; **(C)** LC-MS-based identification strategy of IGFBP5-bound proteins; **(D)** top 30 IGFBP5-bound proteins, ranked by their relative intensities in the chromatogram; **(E)** a PPI network constructed by the 163 proteins only detected in IGFBP5-IP sample. Statistical significance: ^**^ *p* < 0.01 compared with untreated cells.

IGFBP5 was localized on the surface of THP-1 monocytes (**Figure 4A**). Its distribution was decreased after LPS stimulus (**Figure 4B**). Hence, IGFBP5 may interact with certain receptors on cells. Then, a comparative analysis of IGFBP5-IP and control samples was performed based on LC-MS analysis, identifying 163 proteins exclusively bound to IGFBP5 (**Figure 4C**). The detailed protein information is provided in **Supplementary Table S3**. Among the top 30, ANXA2 was the only membrane protein (**Figure 4D**). In addition, it was a central node in the PPI network constructed by these 163 proteins (**Figure 4E**). The results show the key role of ANXA2 in IGFBP5-cuased immune consequences.

**Figure 5:**
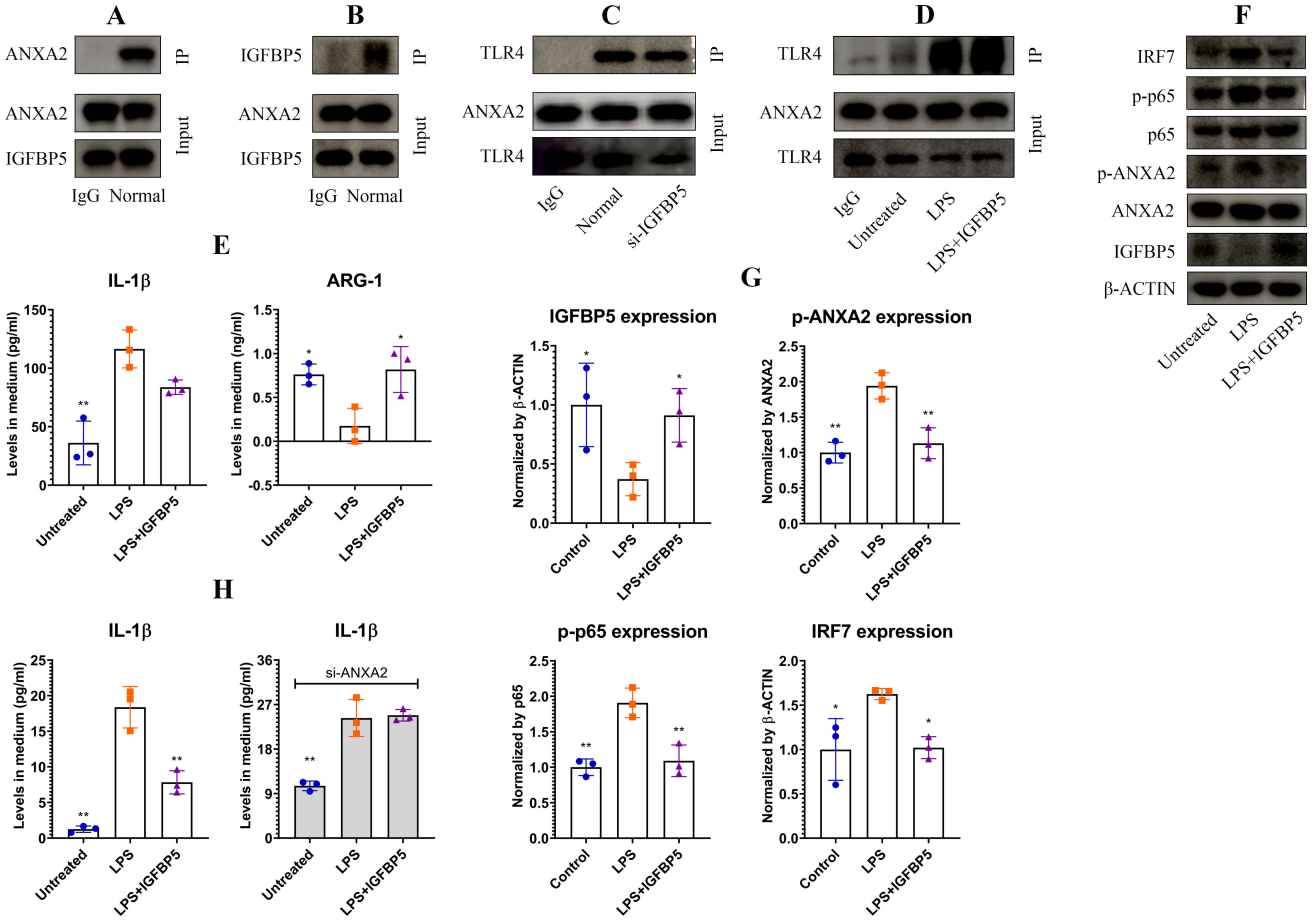
IGFBP5 inhibited inflammation in monocytes by binding to ANXA2. **(A)** ANXA2 expression in IGFBP5-IP sample of normal THP-1 cell lysate; **(B)** IGFBP5 expression in ANXA2-IP sample of normal THP-1 cell lysate; **(C)** TLR4 expression in ANXA2-IP samples of IGFBP5-silenced and normal THP-1 cell lysates; **(D)** TLR4 expression in ANXA2-IP samples of LPS/+IGFBP5-treated and untreated THP-1 cell lysates; **(E)** IL-1β and ARG-1 levels in the medium from experiment D; **(F)** expression of (p)-ANXA2 as well as some immune indicators in the cells from experiment D; **(G)** quantification results of experiment F; **(H)** varied impacts of IGFBP5 on LPS-induced IL-1β secretion in normal and ANXA2-silenced THP-1 cells. Statistical significance: **(B, C, F)**, *p<0.05 and **p<0.01 compared with LPS-stimulated cells; **(E)**, *p<0.05 and **p<0.01 compared with LPS-stimulated cells; **(H)**, *p<0.05 compared with normal.

Co-IP assays confirmed a strong interaction between IGFBP5 and ANXA2 (**Figures 5A, B**). ANXA2 potently activates TLR4 signaling (15). We assumed that intervention of IGFBP5 into their interaction would a cause for the observed immune changes. To test this hypothesis, we silenced IGFBP5, but observed that the interaction between ANXA2 and TLR4 was unaffected (**Figure 5C**). Being a typical agonist of TLR4, LPS reinforced its affinity to ANXA2. IGFBP5 exerted no impact on this outcome (**Figure 5D**). Hence, IGFBP5 does not influence immune status of monocyte/macrophage via modulation of ANXA2-mediated TLR4 activation. Despite this, IGFBP5 treatment effectively suppressed LPS-induced inflammatory polarization of THP-1 monocytes, evidenced by decreased IL-1β level and increased ARG-1 expression (**Figure 5E**). It was reported that ANXA2 can activate NF-κB directly (16). We investigated whether this mechanism is involved in immune-regulatory functions of IGFBP5. IGFBP5 reduced expression of p-ANXA2 and p-p65, leading to IRF7 down-regulation (**Figures 5F, G**). Of note, anti-inflammatory effects of IGFBP5 disappeared when ANXA2 was silenced, but this condition didn’t affect the outcome of LPS stimulus (**Figure 5H**). These findings confirm that ANXA2 is essential for IGFBP5-brought immune changes in monocytes, which are irrelevant to LPS/TLR4 signaling.

### 3.3 IGFBP5 conditionally amplifies inflammation *in vivo*

**Figure 6:**
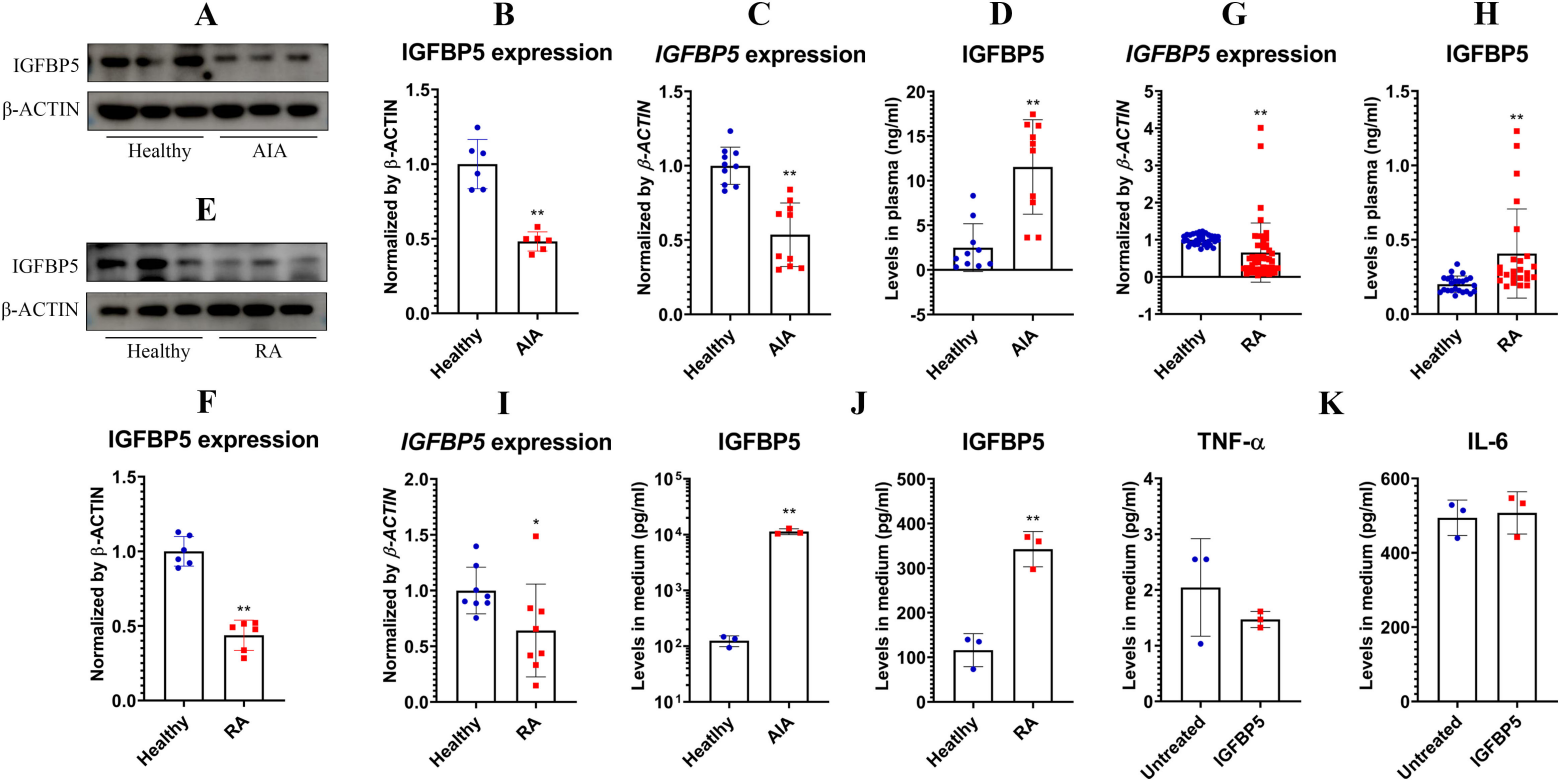
IGFBP5 showed complicated correlations to rheumatic status. **(A)**, IGFBP5 protein expression in healthy and AIA rats’ WBCs; **(B)**, quantification results of experiment A; **(C)**, IGFBP5 mRNA expression in healthy and AIA rats’ WBCs; **(D)**, IGFBP5 levels in healthy and AIA rats’ blood; **(E)**, IGFBP5 protein expression in healthy and RA people’s WBCs; **(F)**, quantification results of experiment D; **(G)**, IGFBP5 mRNA expression in healthy and RA people’s WBCs; **(H)**, IGFBP5 levels in healthy and RA people’s blood; **(I)**, IGFBP5 mRNA expression in healthy and RA people’s monocytes; **(J)**, IGFBP5 secreted by adipocytes cultured in healthy and rheumatic subjects’ blood serum; **(K)**, TNF-α and IL-6 secreted by IGFBP5-stimulated and untreated adipocytes. Statistical significance: p < 0.05 and *p < 0.01 compared with healthy controls or untreated cells.

Based on these findings above, we hypothesized that IGFBP5 expression negatively correlates with inflammation, and its decrease might serve as a diagnostic marker for inflammatory diseases. Indeed, IGFBP5 expression in AIA rats’ WBCs was lower than healthy controls. This result was confirmed by both WB (**Figures 6A, B**) and PCR (**Figure 6C**) analyses. But unexpectedly, IGFBP5 levels in their blood were increased by 5-fold approximately (**Figure 6D**). IGFBP5 expression declined in RA patients’ WBCs too, at both protein (**Figures 6E, F**) and mRNA (**Figure 6G**) levels. Meanwhile, blood levels of IGFBP5 were notably increased (**Figure 6H**). PCR analyses using the *in vitro* cultured human monocytes confirm that IGFBP5 expression was impaired in RA conditions (**Figure 6I**). It suggests that increased IGFBP5 in the blood might originate from other tissues. White adipose tissue (WAT) is the largest secretion organ, and participates in both metabolism and immune regulation (17). It would be a major source of IGFBP5. Indeed, 3T3-L1 cells secreted IGFBP5 at large amounts. This secretion capacity was promoted by the sera of AIA rats and RA patients significantly (**Figure 6J**). But IGFBP5 did not alter immune status of adipocytes. Secretion of TNF-α and IL-6 in these cells was unchanged after IGFBP5 stimulus (**Figure 6K**). The above findings show that WAT contributes more to blood IGFBP5 pool than monocytes.

**Figure 7:**
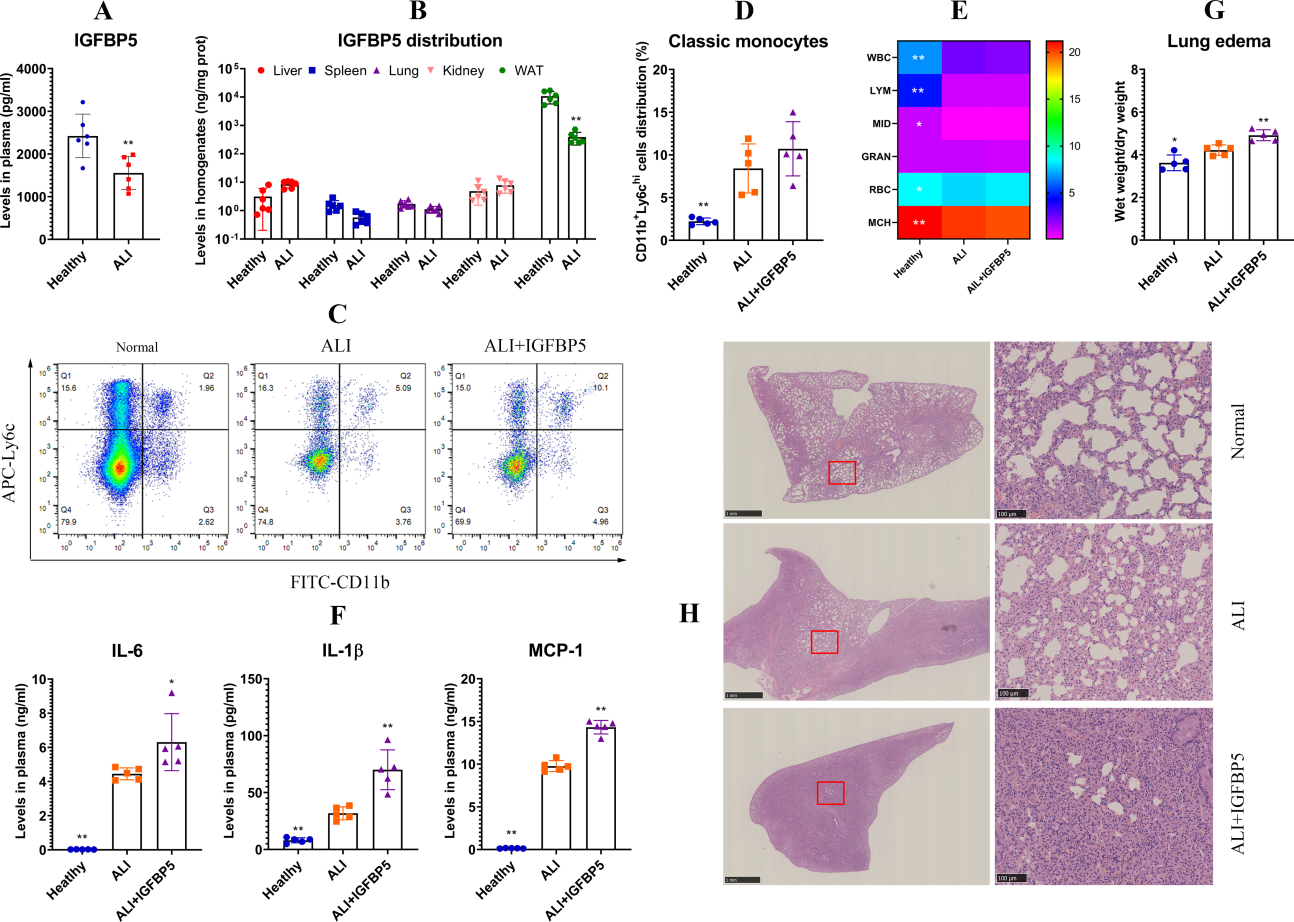
IGFBP5 aggravated inflammation in ALI mice. **(A)** IGFBP5 levels in healthy and ALI mice’s blood; **(B)** IGFBP5 distribution in various tissues of healthy and ALI mice; **(C)** FCM analysis of blood monocytes from healthy, ALI and IGFBP5-treated ALI mice; **(D)** quantification results of experiment C; **(E)** CBC results; **(F)** levels of IL-6, IL-1β and MCP-1 in the mice’s blood; **(G)** lung edema shown by weight changes; **(H)** examination of H&E-stained lung sections. Statistical significance in experiment A-B: ^**^ *p* < 0.01 compared with healthy mice; statistical significance in experiment D-F: ^*^ *p* < 0.05 and ^**^ *p* < 0.01 compared with ALI model mice.

RA patients experience mild and chronic inflammation. The role of IGFBP5 in acute inflammation might be different. To thoroughly clarify the relevance of IGFBP5 to inflammation, we detected IGFBP5 levels in ALI mice, and studied their response to IGFBP5 treatment. Unlike the situation in RA, ALI mice exhibited a significant decrease of IGFBP5 in blood (**Figure 7A**). ELISA analyses of various tissues reveal that circulating IGFBP5 primarily originated from WAT in mice, which was accountable for ALI-caused blood IGFBP5 decline (**Figure 7B**). Although monocytes were not the main source of IGFBP5, they responded actively to IGFBP5. This stimulus further increased the proportion of classic subset in monocytes (**Figures 7C, D**). IGFBP5 did not affect CBC results (**Figure 7E**), but did enhance secretion of IL-6, IL-1β, and MCP-1 in ALI mice (**Figure 7F**). As a result, lung edema was aggravated (**Figure 7G**). Histological examination confirms the detrimental effects of IGFBP5 on ALI. In IGFBP5-treated ALI mice, pathological changes such as inflammatory cell infiltration, alveolar walls and cellular swelling were all worsened (**Figure 7H**).

### 3.4 IGFBP5 competes with IGF1 to promote inflammation

**Figure 8:**
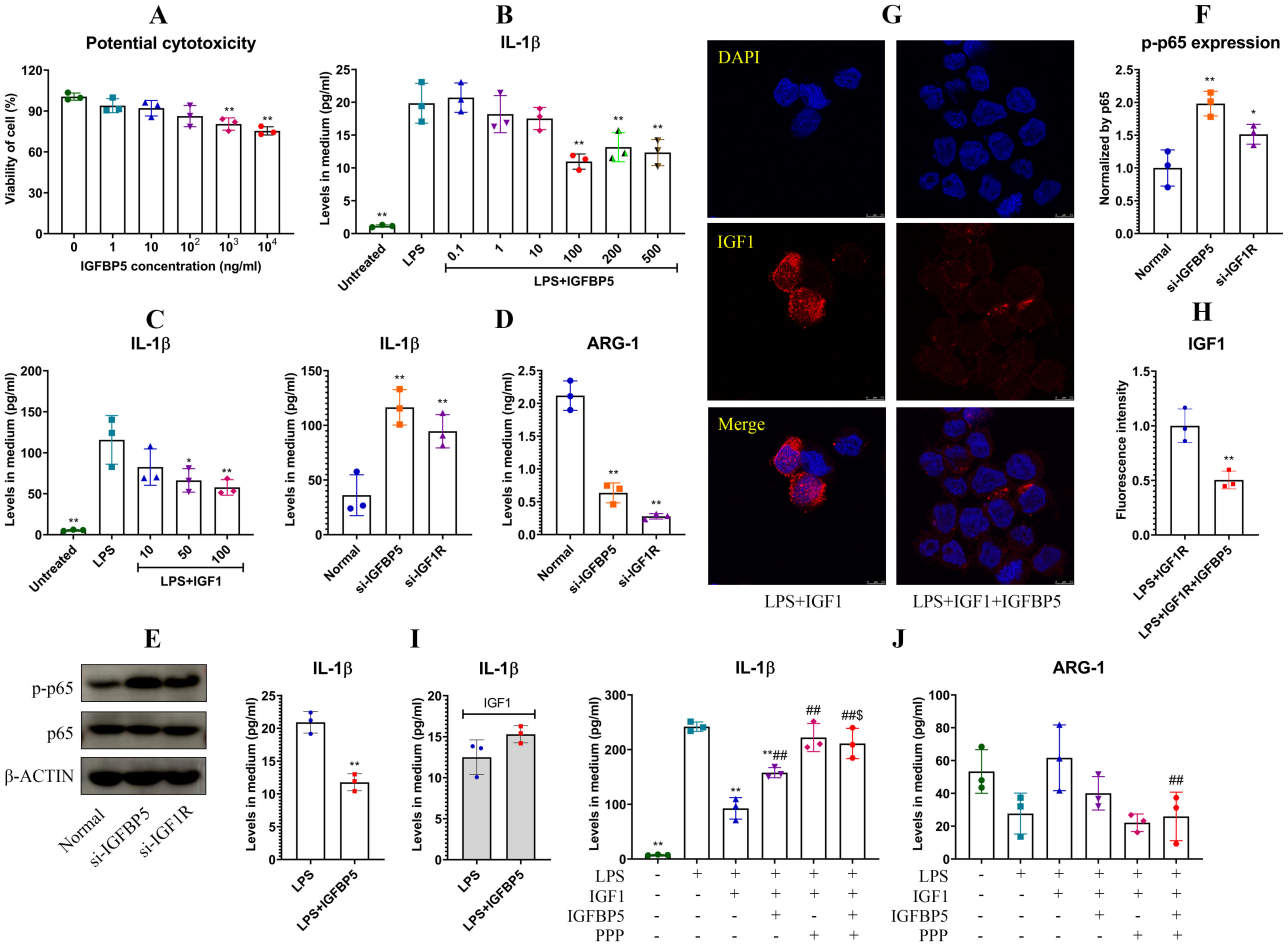
IGFBP5 induced inflammation by antagonizing IGF1/IGF1R in monocytes. **(A)** IGFBP5-brought impacts on viability of THP-1 cells at various concentrations; **(B)** effects of IGFBP5 treatments on IL-1β secretion in LPS-stimulated THP-1 cells; **(C)** effects of IGF1 treatments on IL-1β secretion in LPS-stimulated THP-1 cells; **(D)** IL-1β secreted by normal and IGFBP5/IGF1R-silenced THP-1 cells; **(E)** expression of p-p65 in the cells from experiment D; **(F)** quantification results of experiment E; **(G)** IGFBP5 reduced cytomembrane-bound IGF1; **(H)** quantification results of experiment G; **(I)** differed impacts of IGFBP5 on IL-1β secretion in LPS-stimulated THP-1 cells in the presence of IGF1 or not; **(J)** IGFBP5 and PPP antagonized the effects of IGF1 on LPS-treated THP1 cells. Statistical significance: ^**^ *p* < 0.01 in experiment **(A, D, F)** compared with untreated/normal cells; ^*^ *p* < 0.05 and ^**^ *p* < 0.01 in other experiments compared with LPS-stimulated cells; ^##^ *p* < 0.01 and ^$^ *p* < 0.05 compared with LPS+IGF1-stimulated and LPS+IGF1+IGFBP5-stimulated cells, respectively.

The best known role of IGFBP5 is the regulator of IGF1. By binding to this hormone, IGFBP5 controls its accessibility to IGF1R, which has anti-inflammatory properties (10). This mechanism is possibly related to immune-regulatory functions of IGFBP5, and this theory was investigated afterwards. IGFBP5 did not affect viability of THP-1 cells significantly until reaching 1000 ng/ml (**Figure 8A**). Hence, we investigated effects of IGFBP5 on IL-1β secretion in LPS-treated THP-1 cells in a concentration range of 0.1 to 500 ng/ml. IGFBP5 exerted an anti-inflammatory effect (**Figure 8B**). The main difference of ALI mice from the conditions *in vitro* was the presence of IGF1. This difference would be a decisive variable for these varied outcomes. We next validated anti-inflammatory functions of IGF1/IGF1R. As anticipated, IGF1 stimulus inhibited LPS-induced IL-1β secretion in THP-1 cells in a concentration-dependent manner (**Figure 8C**). Silencing of IGFBP5 and IGF1R both increased IL-1β secretion, while decreased ARG-1 production in the monocytes (**Figure 8D**). In these conditions, p65 was highly phosphorylated (**Figures 8E, F**). Albeit both IGFBP5 and IGF1 negatively regulated inflammation, their co-existence was not harmonious. IGFBP5 binds to IGF1 with the higher affinity than IGF1R (10). Because of that, it prevented IGF1 activating IGF1R on cells (**Figures 8G, H**). Apparently, its interaction with IGF1 reduced free IGFBP5, which can interact with ANXA2 and inhibit NF-κB activation. From this sense, IGFBP5 supplement in medium led to a lose-lose situation for both of them. As such, IGFBP5 suppressed IL-1β secretion in LPS-stimulated THP-1 monocytes under normal conditions, but it exaggerated inflammation in the presence of excessive IGF1 (**Figure 8I**). In the next experiment, we found that IGF1 reduced IL-1β secretion in LPS-stimulated THP-1 cells by more than half. However, this effect was diminished by IGFBP5 and PPP (a selective IGF1R inhibitor). IGFBP5 did not reinforce the effect of PPP, indicating that they used the similar mechanism to antagonize the anti-inflammatory effect of IGF1. The change of ARG-1 mirrored the opposite pattern to that of IL-1β (**Figure 8J**). These findings demonstrate that IGFBP5 disrupts immune homeostasis *in vivo* by repressing anti-inflammatory functions of IGF1/IGF1R.

## 4 Discussion

Guided by the bioinformatics analyses, we discovered IGFBP5 as a novel regulator in polarization of monocytes/macrophages. As a member of the IGFBP family, IGFBP5 is known as a switch of IGF1/IGF1R (18). But less is known about its involvement in immune regulation. This work shows that it regulates functions of ANXA2, a director activator of NF-κB. Via the crosstalk with IGF1R and NF-κB pathways, it must affect internal environment profoundly. Its changes would have certain clinical implications. But the consequences are hard to be predicted, because of the diversified effects from different signal transduction mechanisms. Findings from this work added knowledge about metabolism-immune feedback under pathological conditions, and inspired us to think some tricky phenomena from a comprehensive perspective.

We had known that IGFBP5 can exert effects independently of IGF1R (19, 20). Our work consolidates the standpoint. IGFBP5 mainly localized on the cytomembrane of THP-1 monocytes (**Figure 4A**). Interestingly, ANXA2 was the main IGFBP5-bound membrane protein rather than IGF1R (**Figure 4D**). ANXA2 has long been recognized as a mediator of macrophage activation (21). It activates NF-κB in both TLR4-dependent and -independent manners (15, 16). IGFBP5 did not alter the affinity between TLR4 and ANXA2 (**Figures 5C, D**). The overall evidence demonstrates that it disrupts the direct interplay between ANXA2 and NF-κB independently of TLR4, which accounts for the observed anti-inflammatory results. Its intervention into ANXA2-mediated NF-κB activation can be achieved by various approaches. The subunit p50 of NF-κB is a direct target of ANXA2, which stabilizes p-p65 subunit in the nucleus and sustains its transcriptional activity (16). ANXA2 undergoes phosphorylation, when it is activated (22, 23). The binding of IGFBP5 to ANXA2 would affect these steps. Considering the central role of phosphorylation cascade in signal transduction, we investigated the effects of IGFBP5 stimulus on p-ANXA2 expression. As anticipated, phosphorylation of both ANXA2 and p65 was impeded (**Figure 5F**). Via this mechanism, IGFBP5 impaired inflammatory phenotype of THP-1 cells *in vitro* (**Figure 2**). But we should realize that IGFBP5 is a specific ligand for IGF1 with the high affinity, and abundant IGF1 must abrogate its interaction with ANXA2. Being a key metabolic regulator, IGF1 is always produced at large amounts *in vivo* (10). The relationship of IGFBP5 and IGF1/IGF1R should be considered as a priority in this context.

Over 99% of circulating IGF1 is bound to IGFBPs, with IGFBP5 and IGFBP3 as the dominant players in regulating IGF1 availability. They form ternary complexes with IGF1 and a glycoprotein, resulting in a prolonged half-life of IGF1 in blood (18). Free IGF1 initiates autophosphorylation of IGF1R, and activates two primary signal cascades PI3K/AKT and MAPKs, which regulates glucose metabolism and cellular growth/proliferation respectively (10). Thereby IGF1/IGF1R alteration usually occurs in cancers and metabolic disorders (10, 24). In fact, this pathway is closely related to immune regulation too. IGF1 exerts anti-inflammatory effects across various organs, involving metabolic, digestive, nervous, and immune systems (25–28). Consistently, some inflammatory diseases like RA are associated with decreased levels of IGF1 (29). It was reported that IGF1 exerts the anti-inflammatory effects in monocytes by inhibiting p65 phosphorylation (30). This mechanism was validated (**Figure 8E**). Given its high abundance *in vivo*, IGF1 must act as an important role in maintaining immune homeostasis, in addition to metabolism regulation. High levels of IGFBP5 will reduce its availability, and consequently disrupt immune balance by diminishing inhibition of IGF1/IGF1R on p65 NF-κB. Metabolism-regulatory properties of IGF1 and IGFBP5 are also meaningful for their immune functions. By sensitizing insulin pathway, IGF1 promotes glucose uptake and oxidation (10). This is a typical metabolic phenotype of M2 monocytes/macrophages. IGFBP5 would change this status as an antagonist of IGF1/IGF1R. In line with this theory, IGFBP5 aggravated inflammation by enhancing PFKFB3-mediated endodermal glycolysis (31). Due to the above mechanisms, many inflammatory diseases are associated with IGFBP5 increase (18).

The above findings provide a new strategy to treat inflammatory diseases, but a priority is to figure out the predominant source of IGFBP5. As a metabolic regulator, IGFBP5 would be mainly released by metabolism-regulatory organs. Consistent to the hypothesis, WAT expressed much higher levels of IGFBP5 than other organs (**Figure 7B**). By comparing the data in **Figures 2C**, **6G**, we further confirmed this conclusion, and ruled out monocytes as a main source of IGFBP5. Previously, we identified WAT as a therapeutic target of RA (12). This study provides more evidence to support the idea. It is noteworthy that blood supply in WAT is limited. As the result, it responds slowly to environmental changes, and its abnormalities occur in chronic diseases only. It explains the inconsistent phenomena observed in ALI mice and RA patients (**Figures 6H** and **7A**). Long-lasting inflammation promoted IGFBP5 secretion in WAT of rheumatic subjects. This change was insignificant in ALI mice, due to the short time stimulus of LPS. But IGFBP5 expression decreased in their immune cells, which led to blood IGFBP5 decline. Hence, decreasing IGFBP5 is a feasible tactic only suitable for therapies of chronic diseases, and WAT is an ideal target in this aspect. But there are some obstacles ahead: we don’t know the exact mechanism about how IGFBP5 is expressed and secreted; given its versatile functions, this change would cause certain unfavorable consequences.

## 5 Conclusion

IGFBP5 expression exhibits a negative correlation with the inflammatory polarization of monocytes, and inhibits NF-κB activation through its interaction with ANXA2. But on the other hand, it promotes inflammation by disrupting IGF1/IGF1R pathway. In certain chronic inflammatory diseases, although immune cells release less IGFBP5, its blood levels increase due to the secretion enhancement in WAT. Because IGF1 is an abundant endogenous mediator required by immune homeostasis, excessive IGFBP5 always exerts inflammatory functions *in vivo*.

## Data Availability

The original contributions presented in the study are included in the article/**Supplementary Material**. Further inquiries can be directed to the corresponding authors.

## Supplementary material

Supplementary Table S1 Sequences of the gene-specific primers and siRNA.

Supplementary Table S2 KEGG pathway enrichment results.

Supplementary Table S3 IGFBP5-bound proteins.

## References

[1] Hillion S Arleevskaya MI Blanco P Bordron A Brooks WH Cesbron JY. The innate part of the adaptive immune system. Clin Rev Allergy Immunol. (2020) 58:151–4. doi: 10.1007/s12016-019-08740-1 31154567

[2] Deets KA Vance RE. Inflammasomes and adaptive immune responses. Nat Immunol. (2021) 22:412–22. doi: 10.1038/s41590-021-00869-6 33603227

[3] Das A Sinha M Datta S Abas M Chaffee S Sen CK. Monocyte and macrophage plasticity in tissue repair and regeneration. Am J Pathol. (2015) 185:2596–606. doi: 10.1016/j.ajpath.2015.06.001 PMC4607753 26118749

[4] Gordon S Taylor PR. Monocyte and macrophage heterogeneity. Nat Rev Immunol. (2005) 5:953–64. doi: 10.1038/nri1733 16322748

[5] Li D Wu M. Pattern recognition receptors in health and diseases. Signal Transduct Target Ther. (2021) 6:291. doi: 10.1038/s41392-021-00687-0 34344870 PMC8333067

[6] Zheng Y Wei K Jiang P Zhao J Shan Y Shi Y. Macrophage polarization in rheumatoid arthritis: signaling pathways, metabolic reprogramming, and crosstalk with synovial fibroblasts. Front Immunol. (2024) 15:1394108. doi: 10.3389/fimmu.2024.1394108 38799455 PMC11116671

[7] Fleetwood AJ Noonan J La Gruta N Kallies A Murphy AJ. Immunometabolism in atherosclerotic disorders. Nat Cardiovasc Res. (2024) 3:637–50. doi: 10.1038/s44161-024-00473-5 39196223

[8] Gauthier T Chen W. Modulation of macrophage immunometabolism: a new approach to fight infections. Front Immunol. (2022) 13:780839. doi: 10.3389/fimmu.2022.780839 35154105 PMC8825490

[9] Lei M Tao MQ Wu YJ Xu L Yang Z Li Y. Metabolic enzyme triosephosphate isomerase 1 and nicotinamide phosphoribosyltransferase, two independent inflammatory indicators in rheumatoid arthritis: evidences from collagen-induced arthritis and clinical samples. Front Immunol. (2022) 12:795626. doi: 10.3389/fimmu.2021.795626 35111160 PMC8801790

[10] Forbes BE Blyth AJ Wit JM. Disorders of IGFs and IGF-1R signaling pathways. Mol Cell Endocrinol. (2020) 518:111035. doi: 10.1016/j.mce.2020.111035 32941924

[11] Szklarczyk D Franceschini A Wyder S Forslund K Heller D Huerta-Cepas J. STRING v10: protein-protein interaction networks, integrated over the tree of life. Nucleic Acids Res. (2015) 43:D447–52. doi: 10.1093/nar/gku1003 PMC4383874 25352553

[12] Ye P Wang QH Kong WY Liu CS Wang DD Olatunji OJ. White adipose tissue, a novel anti-rheumatic target: clues from its secretion capability and adipectomy-based therapy. Brit J Pharmacol. (2024) 181:2774–93. doi: 10.1111/bph.v181.16 38644540

[13] Ye P Wang QH Liu CS Li GH Olatunji OJ Lin JT. SIRT1 inhibitors within Qing-Luo-Yin alleviated white adipose tissues-mediated inflammation in antigen-induced arthritis mice. Phytomedicine. (2024) 122:155132. doi: 10.1016/j.phymed.2023.155132 37844379

[14] Cheng XP Wang XW Sun HF Xu L Olatunji OJ Li Y. NAMPT/SIRT1 expression levels in white blood cells differentiate the different rheumatoid arthritis subsets: an inspiration from Traditional Chinese Medicine. J Inflammation Res. (2023) 16:4271–85. doi: 10.2147/JIR.S431600 PMC10543492 37791116

[15] Wu Z Xu Z Pu H Ding A Hu J Lei J. NINJ1 facilitates abdominal aortic aneurysm formation via blocking TLR4-ANXA2 interaction and enhancing macrophage infiltration. Adv Sci. (2024) 11(31):e2306237. doi: 10.1002/advs.202306237 PMC11336960 38922800

[16] Jung H Kim JS Kim WK Oh KJ Kim JM Lee HJ. Intracellular annexin A2 regulates NF-κB signaling by binding to the p50 subunit: implications for gemcitabine resistance in pancreatic cancer. Cell Death Dis. (2015) 6:e1606. doi: 10.1038/cddis.2014.558 25611381 PMC4669756

[17] Santillana N Astudillo-Guerrero C D’Espessailles A Cruz G. White adipose tissue dysfunction: pathophysiology and emergent measurements. Nutrients. (2023) 15:1722. doi: 10.3390/nu15071722 37049561 PMC10096946

[18] Ding H Wu T. Insulin-like growth factor binding proteins in autoimmune diseases. Front Endocrinol. (2018) 9:499. doi: 10.3389/fendo.2018.00499 PMC6125368 30214426

[19] Waters JA Urbano I Robinson M House CD. Insulin-like growth factor binding protein 5: Diverse roles in cancer. Front Oncol. (2022) 12:1052457. doi: 10.3389/fonc.2022.1052457 36465383 PMC9714447

[20] Poreba E Durzynska J. Nuclear localization and actions of the insulin-like growth factor 1 (IGF-1) system components: Transcriptional regulation and DNA damage response. Mutat Res Rev Mutat Res. (2020) 784:108307. doi: 10.1016/j.mrrev.2020.108307 32430099

[21] Swisher JF Khatri U Feldman GM. Annexin A2 is a soluble mediator of macrophage activation. J Leukoc Biol. (2007) 82:1174–84. doi: 10.1189/jlb.0307154 17715360

[22] Flood EC Hajjar KA. The annexin A2 system and vascular homeostasis. Vascul Pharmacol. (2011) 54:59–67. doi: 10.1016/j.vph.2011.03.003 21440088 PMC3109204

[23] Grindheim AK Saraste J Vedeler A. Protein phosphorylation and its role in the regulation of Annexin A2 function. Biochim Biophys Acta Gen Subj. (2017) 1861:2515–29. doi: 10.1016/j.bbagen.2017.08.024 28867585

[24] Aguirre GA De Ita JR de la Garza RG Castilla-Cortazar I. Insulin-like growth factor-1 deficiency and metabolic syndrome. J Transl Med. (2016) 14:3. doi: 10.1186/s12967-015-0762-z 26733412 PMC4702316

[25] Hu P Thinschmidt JS Caballero S Adamson S Cole L Chan-Ling T. Loss of survival factors and activation of inflammatory cascades in brain sympathetic centers in type 1 diabetic mice. Am J Physiol Endocrinol Metab. (2015) 308:E688–698. doi: 10.1152/ajpendo.00504.2014 PMC4398829 25714673

[26] Ge RT Mo LH Wu R Liu JQ Zhang HP Liu Z. Insulin-like growth factor-1 endues monocytes with immune suppressive ability to inhibit inflammation in the intestine. Sci Rep. (2015) 5:7735. doi: 10.1038/srep07735 25588622 PMC4295102

[27] Yamamoto N Nakagawa T Ito J. Application of insulin-like growth factor-1 in the treatment of inner ear disorders. Front Pharmacol. (2014) 5:208. doi: 10.3389/fphar.2014.00208 25309440 PMC4159992

[28] Spadaro O Camell CD Bosurgi L Nguyen KY Youm YH Rothlin CV. IGF1 shapes macrophage activation in response to immunometabolic challenge. Cell Rep. (2017) 19:225–34. doi: 10.1016/j.celrep.2017.03.046 PMC5513500 28402847

[29] Zhao YL Wu J Zhang TP Cheng QY Wang XP Gu MM. Circulating insulin-like growth factor-1 levels in patients with rheumatoid arthritis: a meta-analysis. Curr Pharm Des. (2019) 25:1091–8. doi: 10.2174/1381612825666190319124009 30892152

[30] Mack EM Smith JE Kurz SG Wood JR. cAMP-dependent regulation of ovulatory response genes is amplified by IGF1 due to synergistic effects on Akt phosphorylation and NF-κB transcription factors. Reproduction. (2012) 144:595–602. doi: 10.1530/REP-12-0225 22956516

[31] Song C Wang S Fu Z Chi K Geng X Liu C. IGFBP5 promotes diabetic kidney disease progression by enhancing PFKFB3-mediated endothelial glycolysis. Cell Death Dis. (2022) 13:340. doi: 10.1038/s41419-022-04803-y 35418167 PMC9007962

