# Supplementary material for: Interaction with IGF1 overrides ANXA2-mediated anti-inflammatory functions of IGFBP5 in vivo
Source: Front Immunol. 2025 Jan 10;15:1539317. doi: 10.3389/fimmu.2024.1539317 (PMC11757107; doi:10.3389/fimmu.2024.1539317)
Supplement: Supplementary Table S1 — Sequences of the gene-specific primers and siRNA. [file Table1.docx]

Sequences of the gene-specific primers and siRNA used in this study

| Gene | Sequences | Species |
| --- | --- | --- |
| *β-Actin* | Forward, TCCATCGGAGCCGAAGAAATC | Homo |
| *β-Actin* | Reverse, GTGTCGGTGGATCAAAGCACA | Homo |
| *β-Actin* | Forward, TGGAATCCTGTGGCATCCATGAAA | Rat |
| *β-Actin* | Reverse, TAAAACGCAGCTCAGTAACAGTCCG | Rat |
| *IGFBP5* | Forward, ACGCGTCGACATGGGCTCCTTCGTGCAC | Homo |
| *IGFBP5* | Reverse,CGCGGATCCATCACTCAACGTTGCTGCTG | Homo |
| *IGFBP5* | Forward, ACTGTGACCGCAAAGGATTC | Rat |
| *IGFBP5* | Reverse, TTGTCCACACACCAGCAGAT | Rat |
| *si-ANXA2* | Forward, CAAGAUGCUCGGGAUCUCUTT | Homo |
| *si-ANXA2* | Reverse, AGAGAUCCCGAGCAUCUUGTT | Homo |
| *si-IGF1R* | Forward, GGAGAGAACUGUCAUUUCUTT | Homo |
| *si-IGF1R* | Reverse, AGAAAUGACAGUUCUCUCCTT | Homo |
| si-*IGFBP5* | Forward, GUGACCGCAAAGGAUUCUATT | Homo |
| si-*IGFBP5* | Reverse, UAGAAUCCUUUGCGGUCACTT | Homo |
