# Supplementary material for: Interaction with IGF1 overrides ANXA2-mediated anti-inflammatory functions of IGFBP5 in vivo
Source: Front Immunol. 2025 Jan 10;15:1539317. doi: 10.3389/fimmu.2024.1539317 (PMC11757107; doi:10.3389/fimmu.2024.1539317)
Supplement: Supplementary file 4 [file Table4.docx]

Figure 2A

Beta-ACTIN


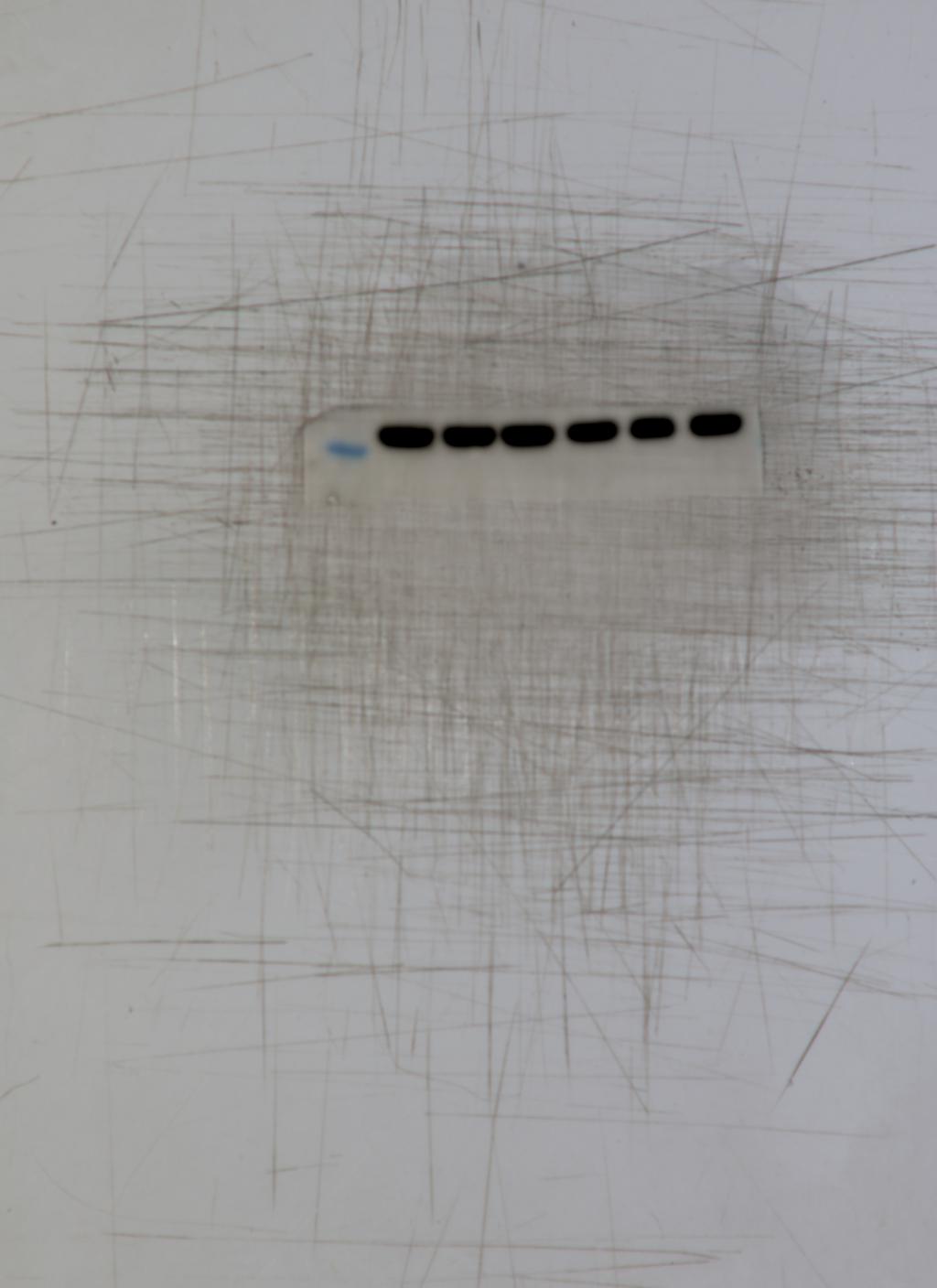


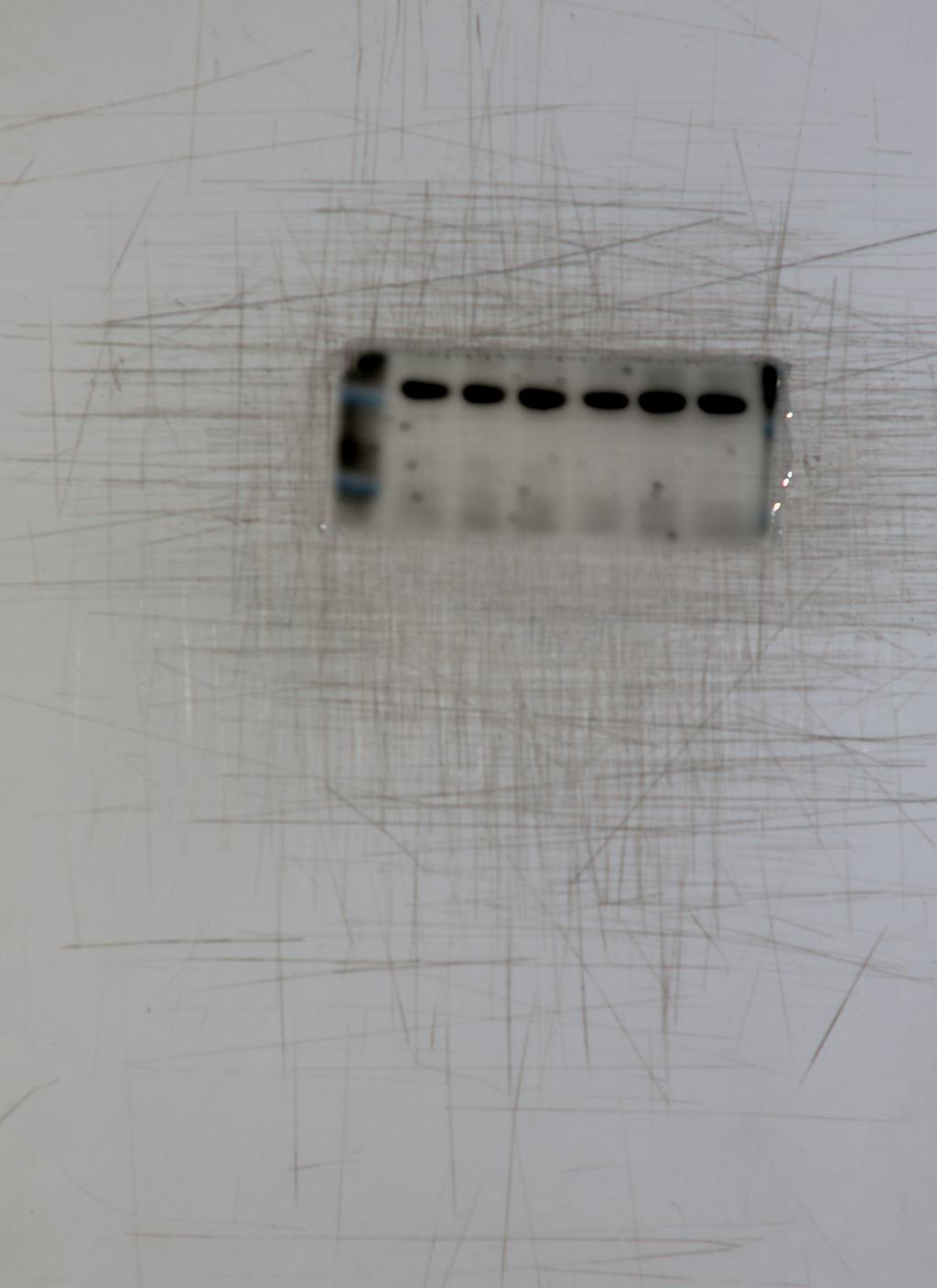


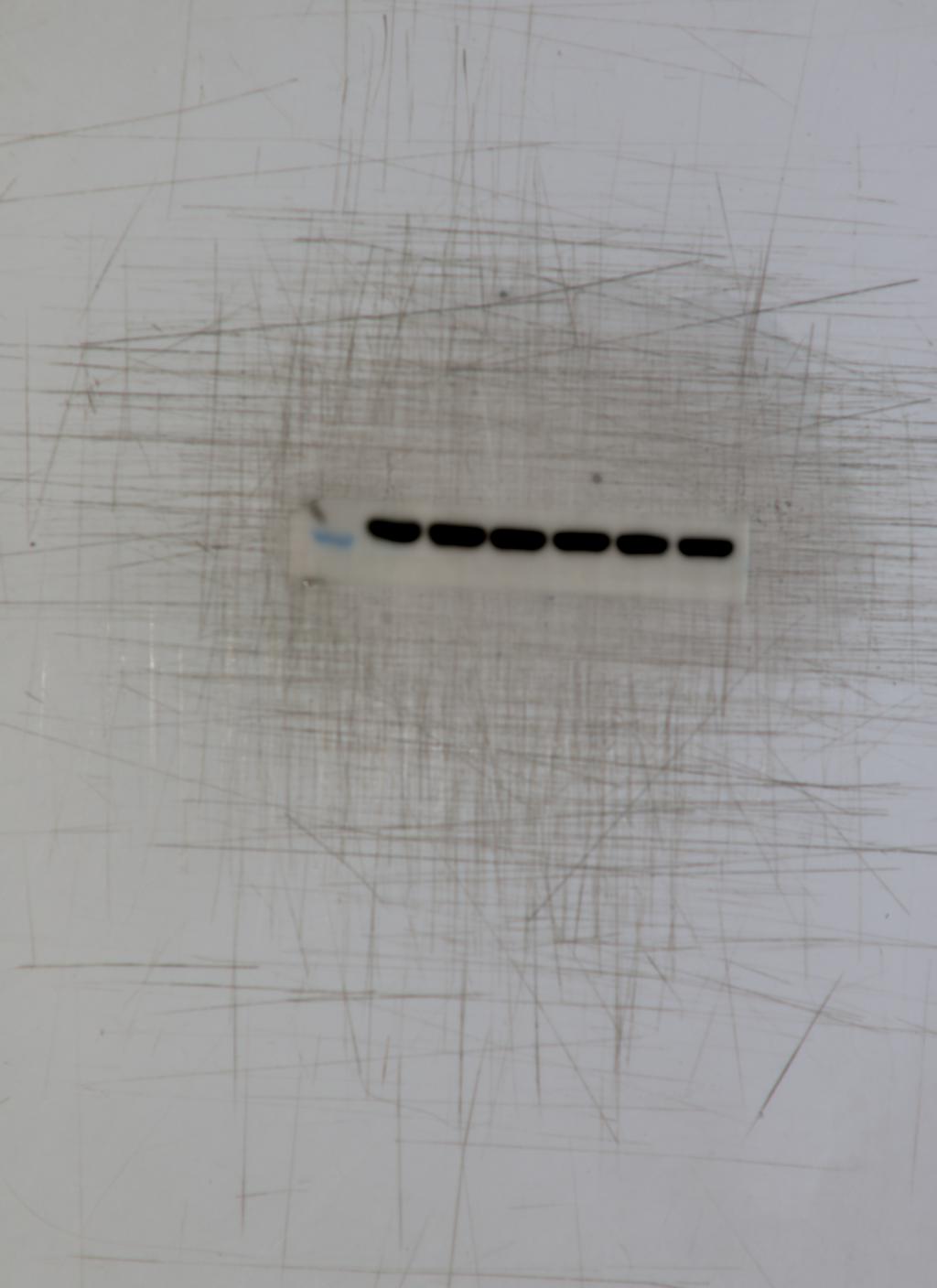


IGFBP5


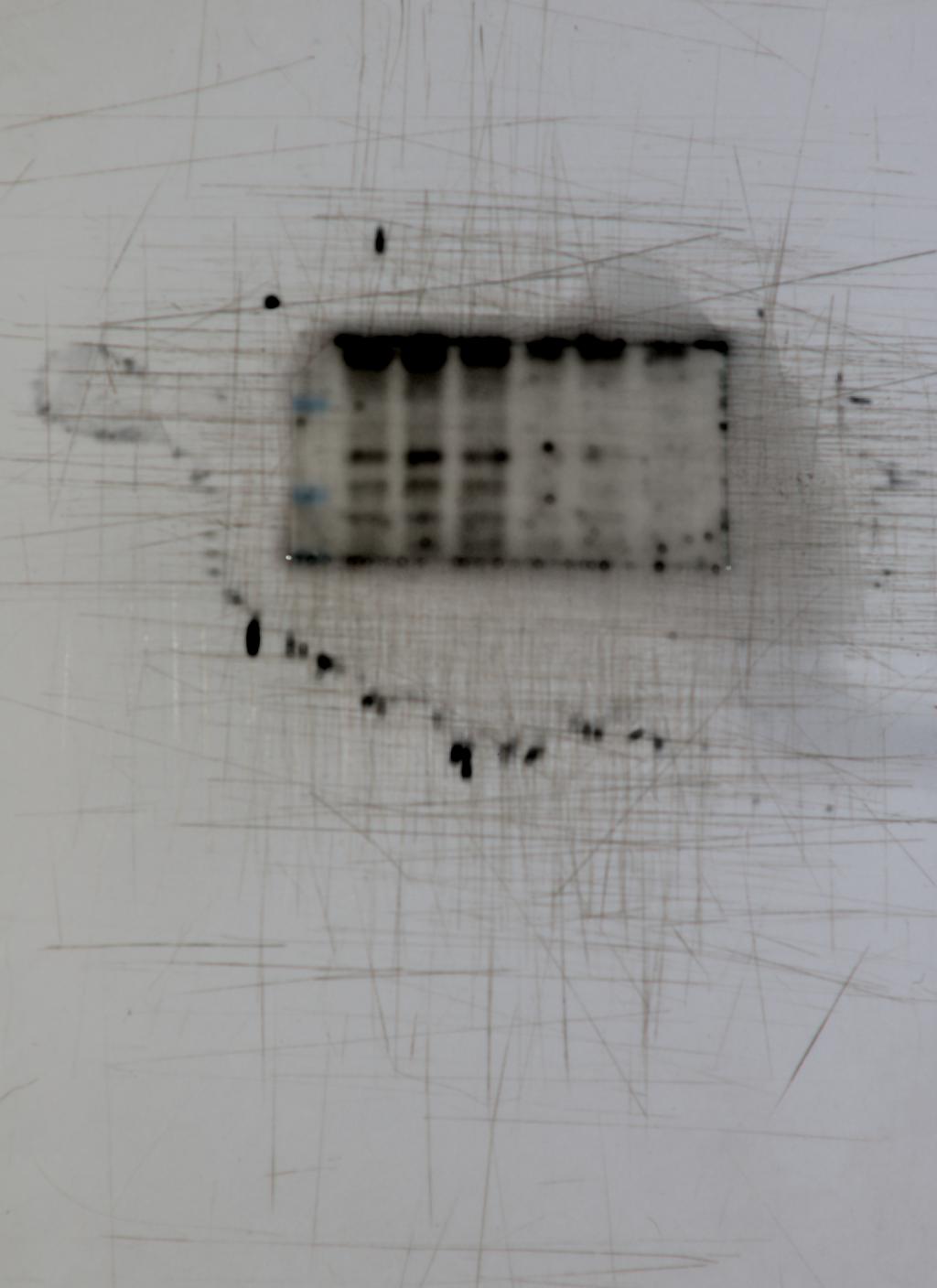


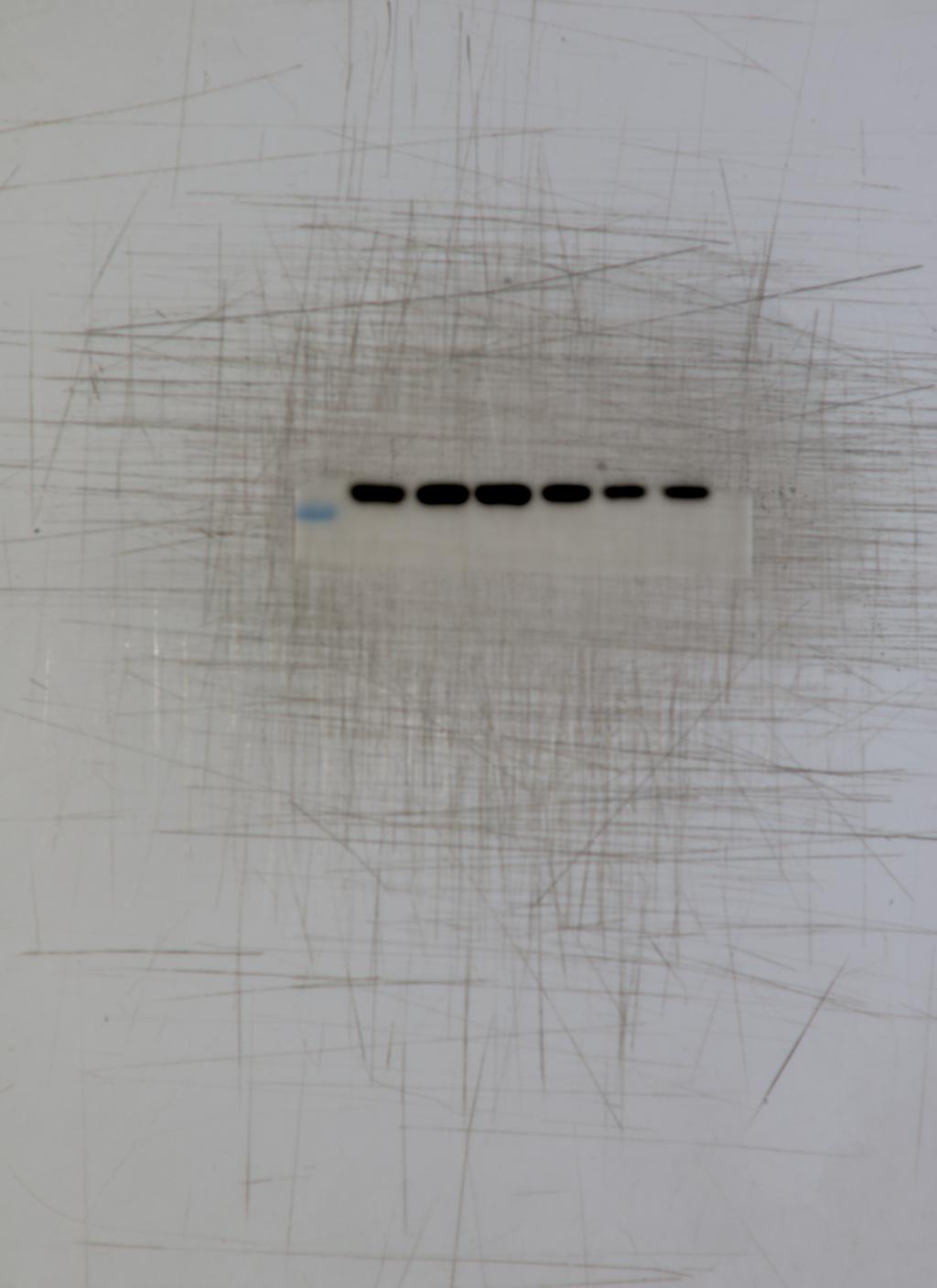


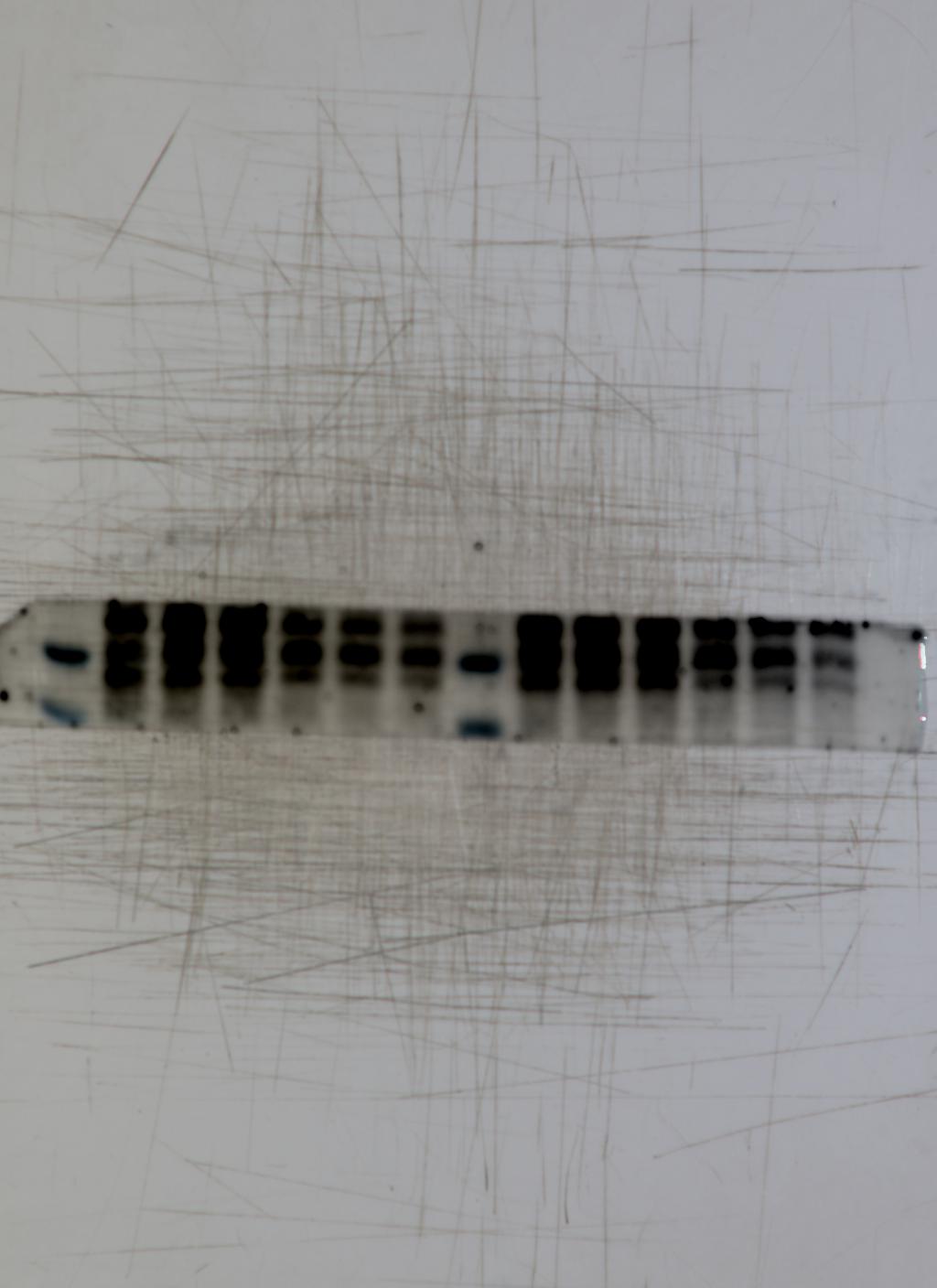


IRF7


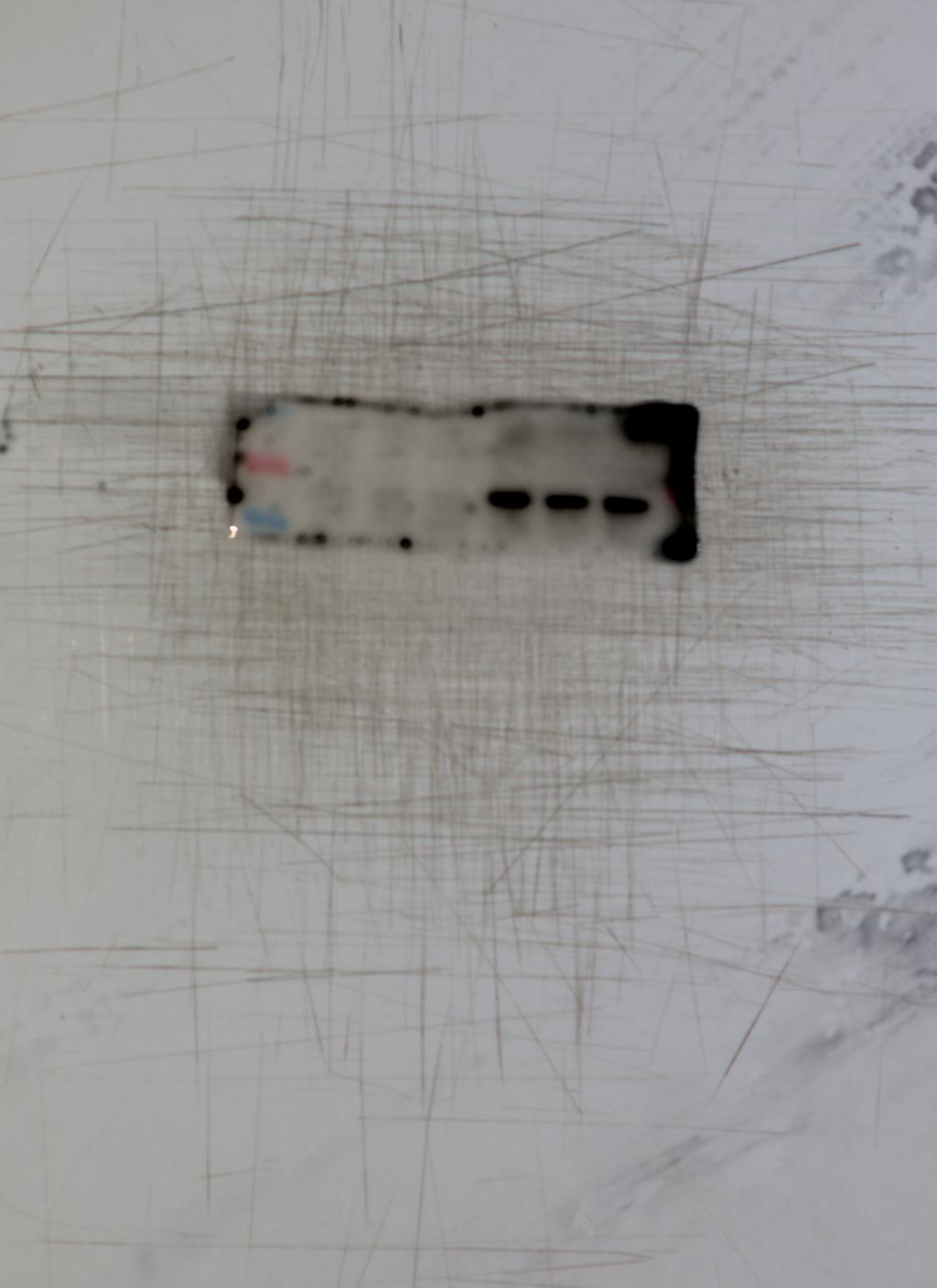


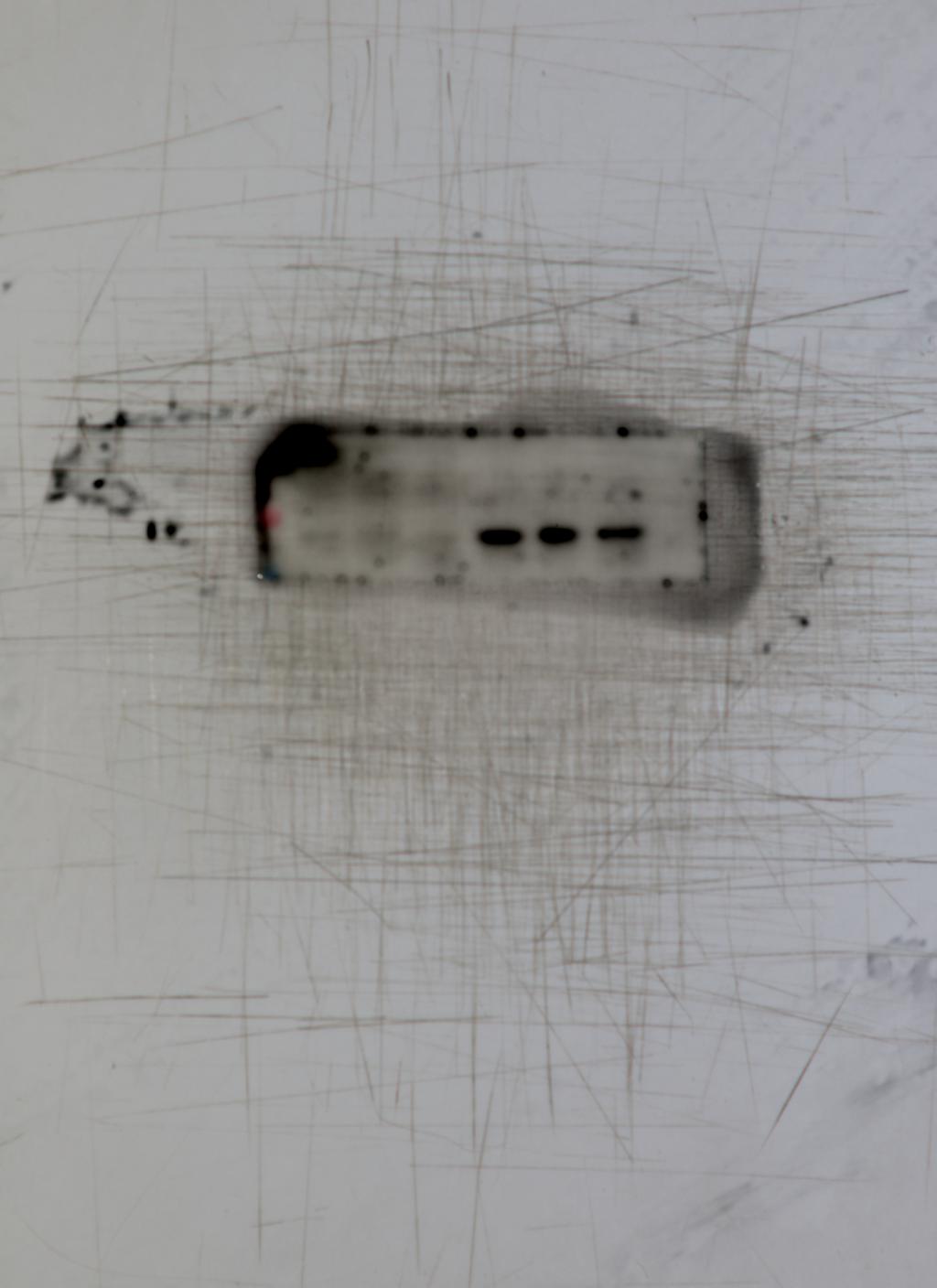


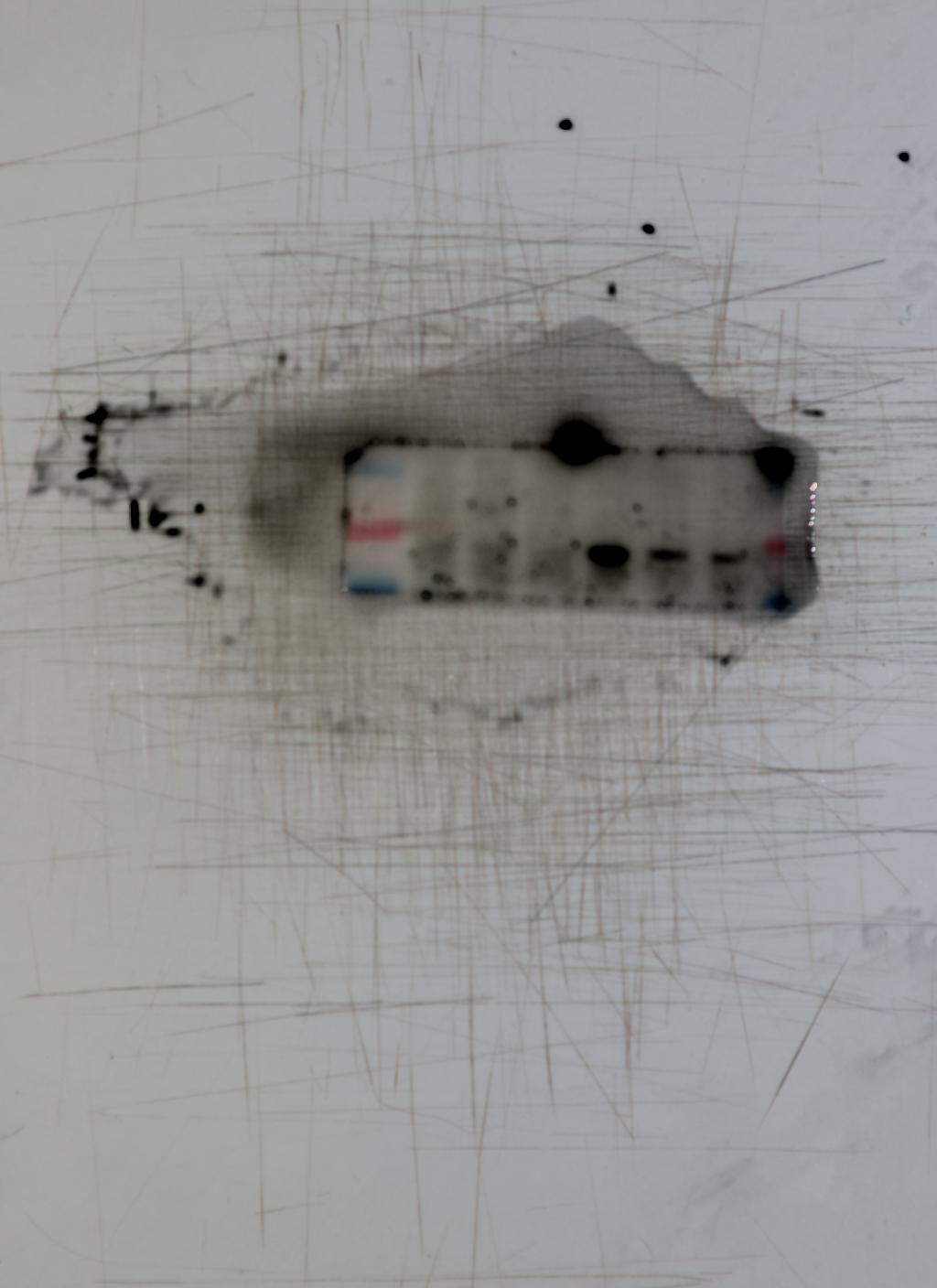


Figure 2C

Beta-ACTIN


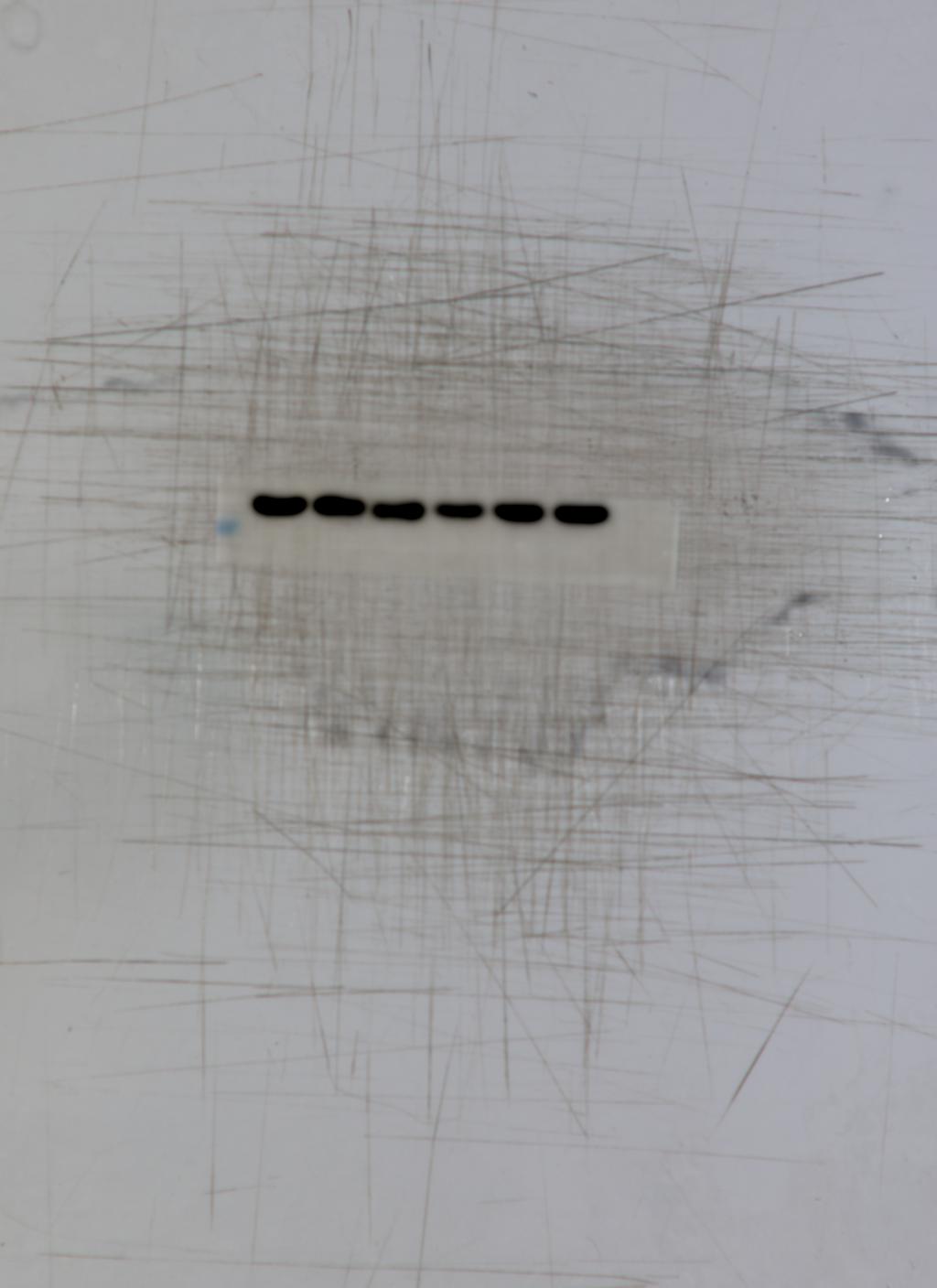


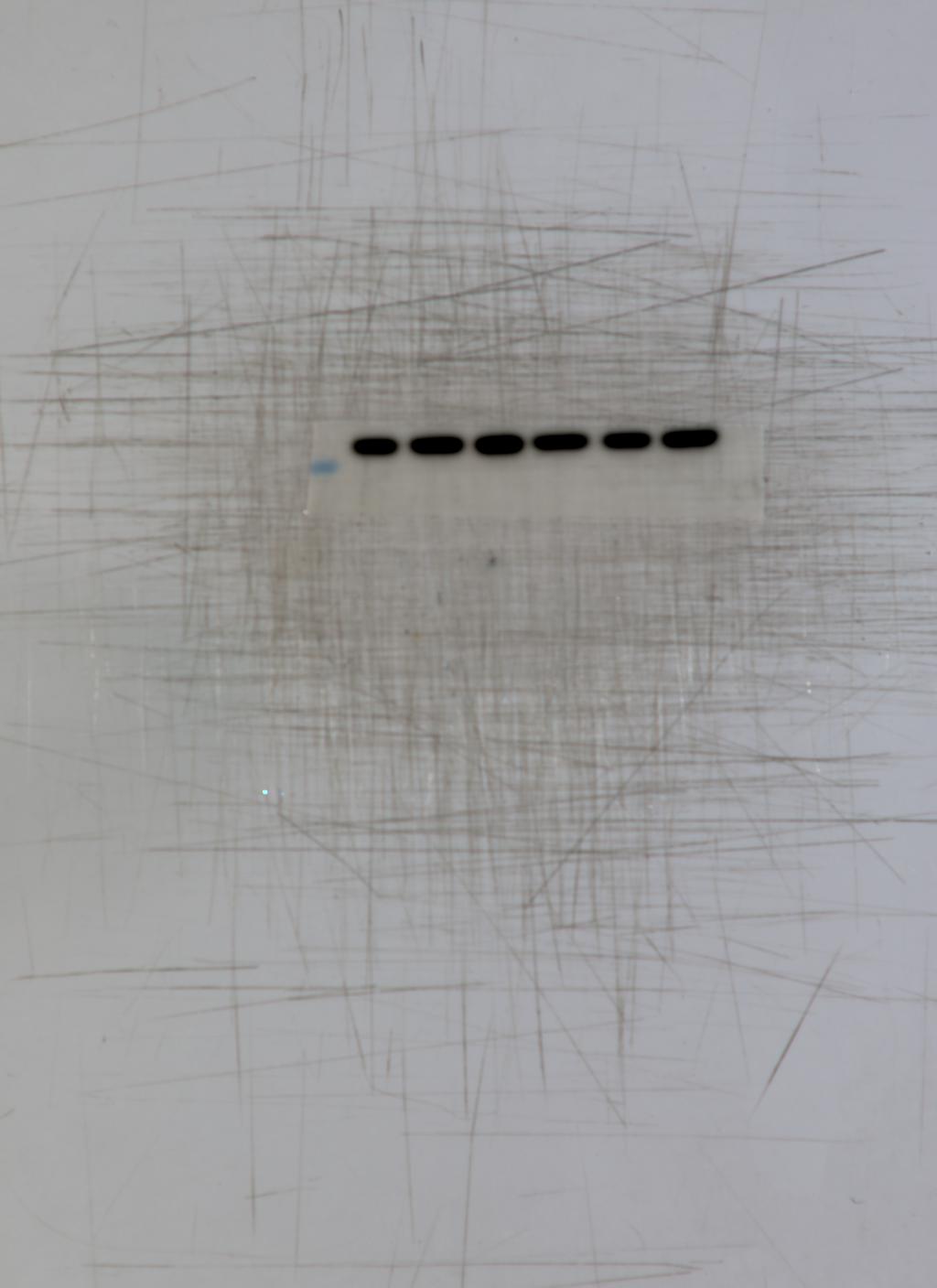


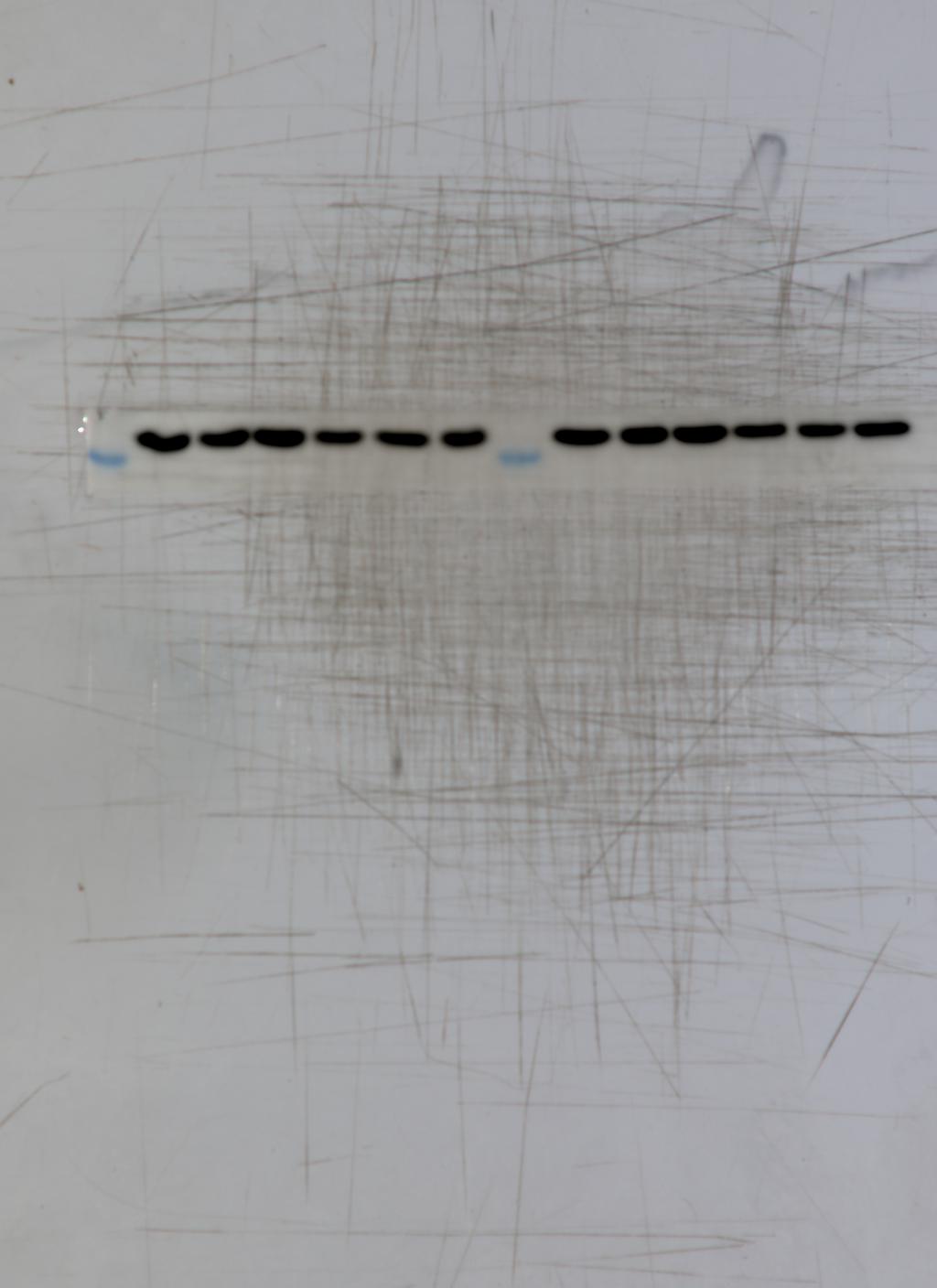


IGFBP5


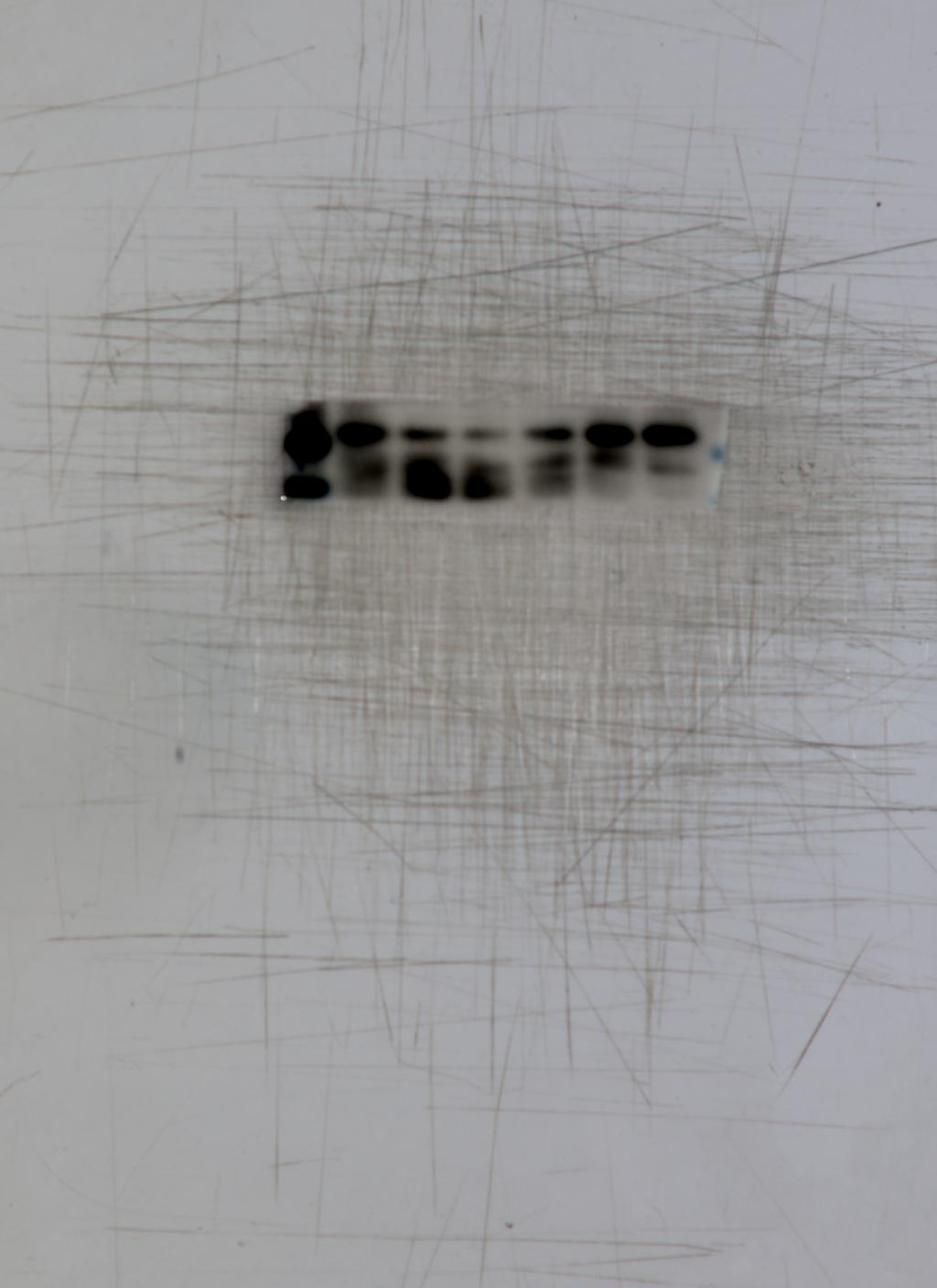


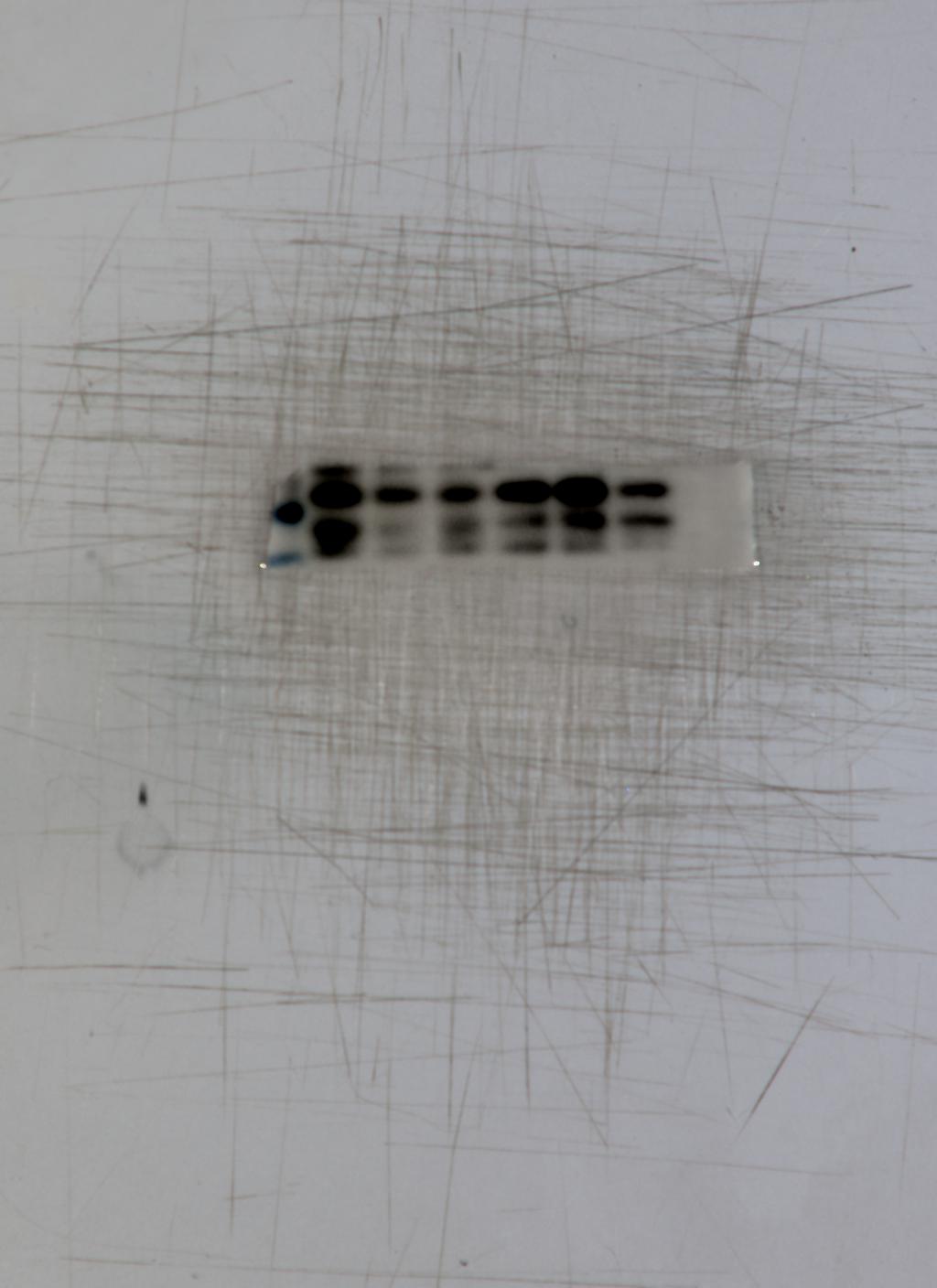


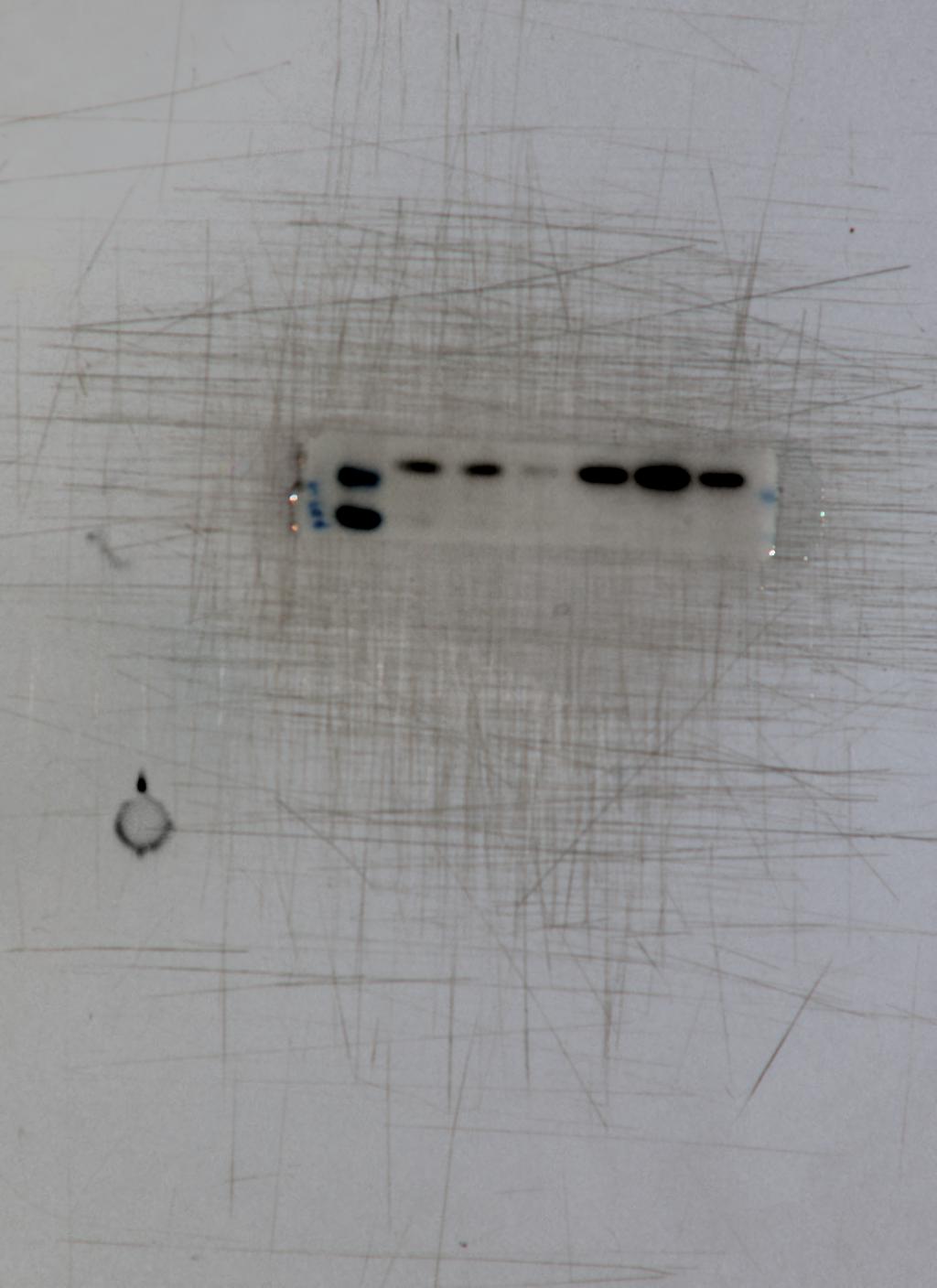


iNOS


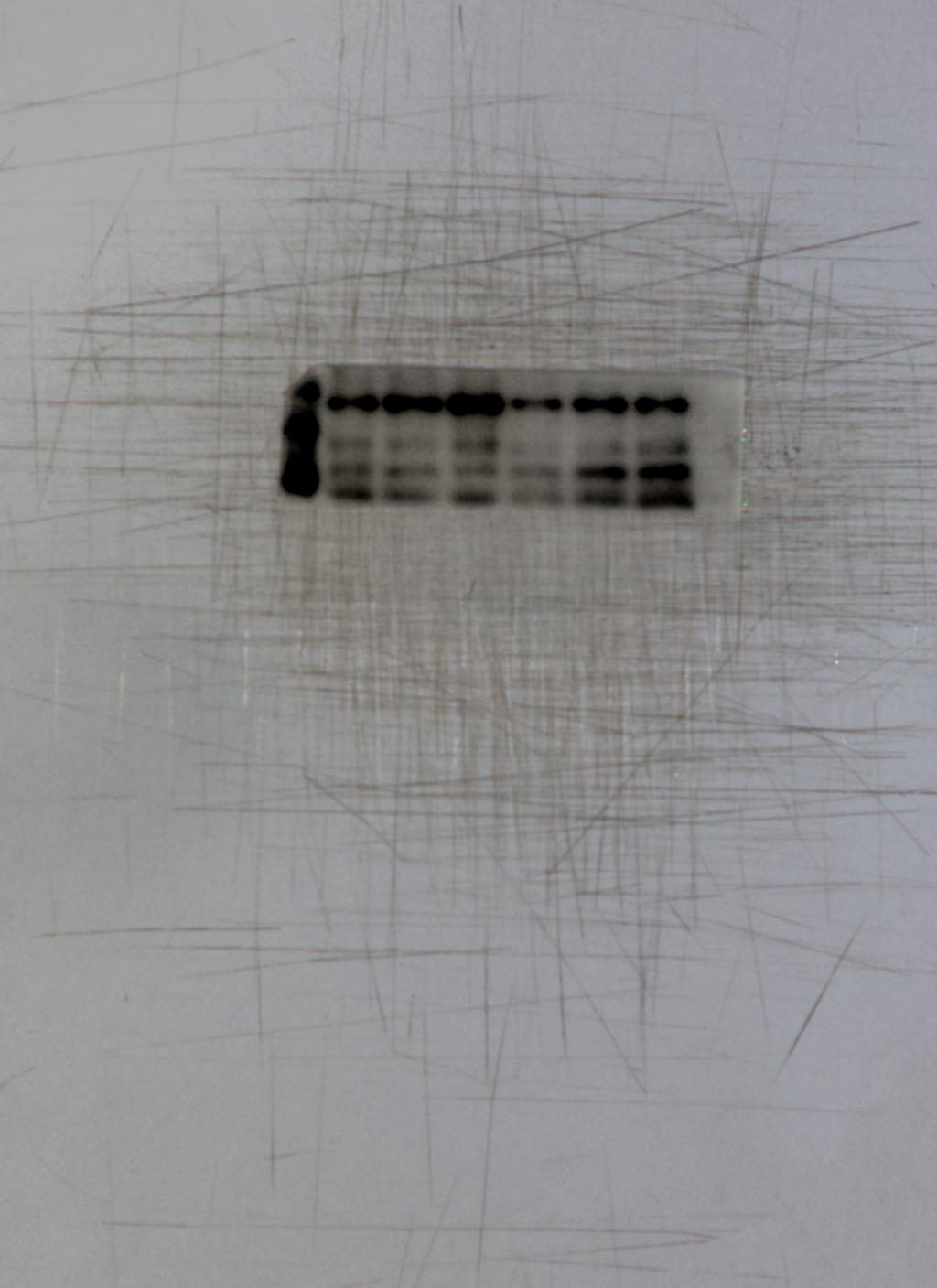


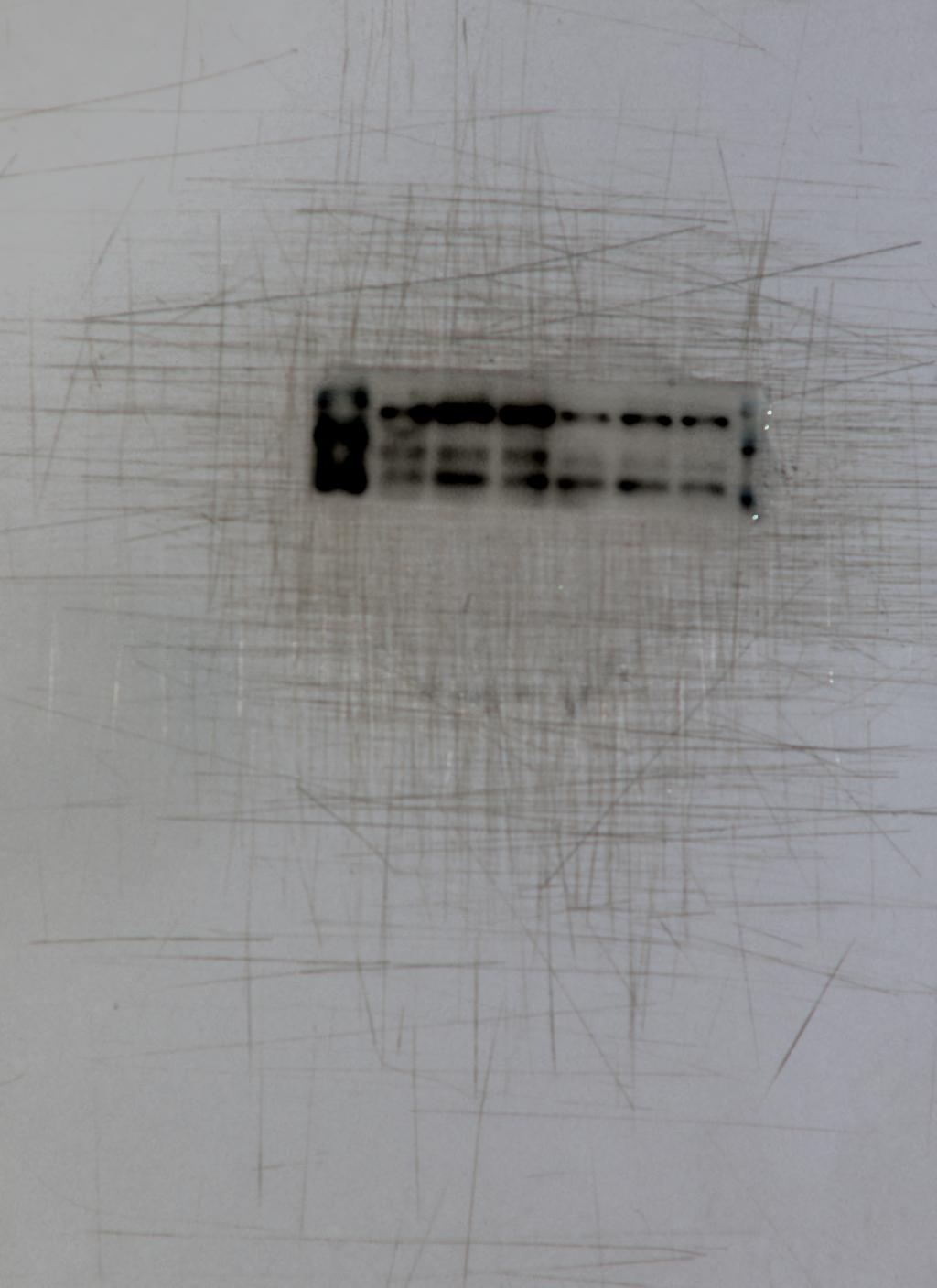


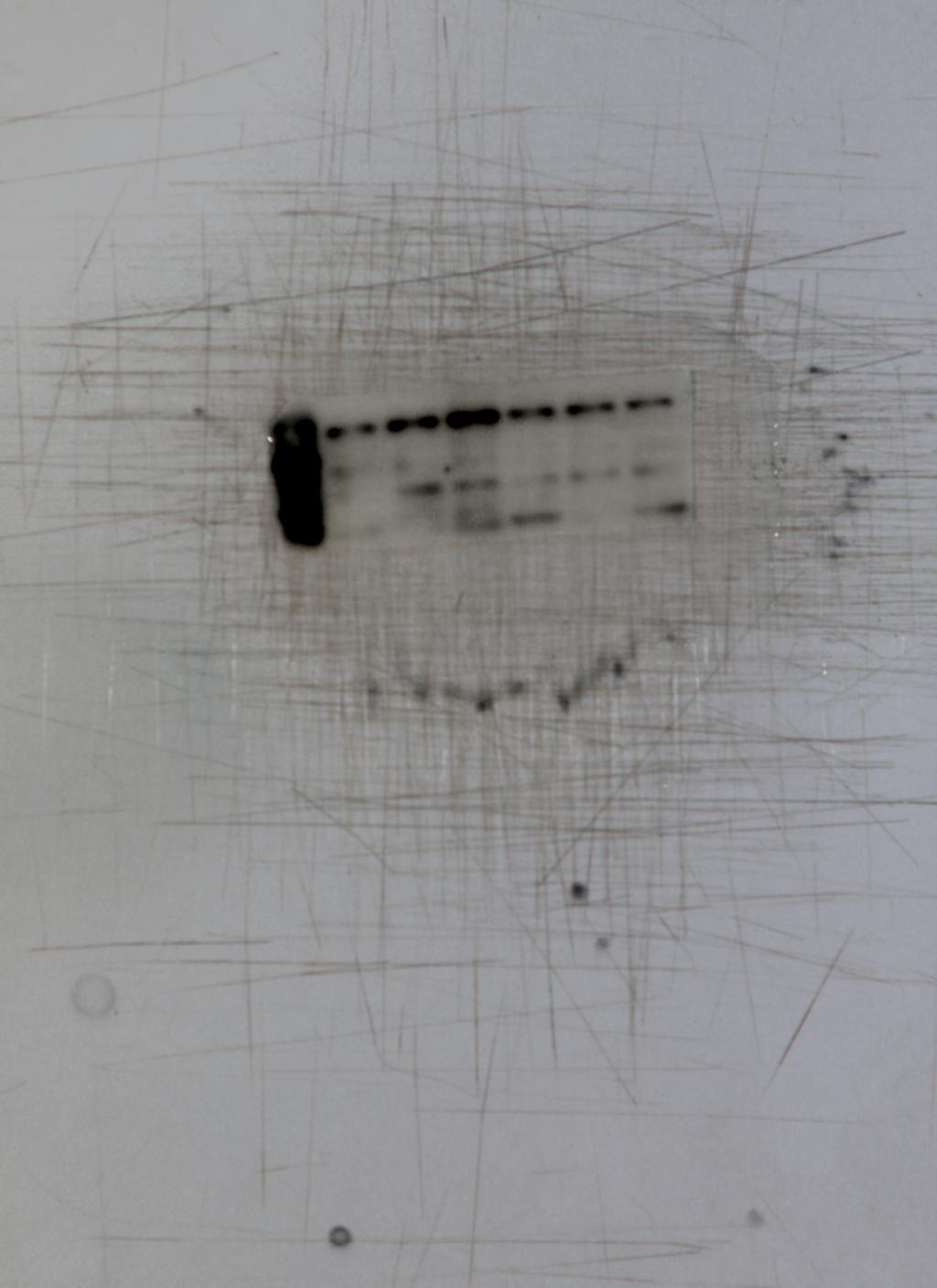


MMP3


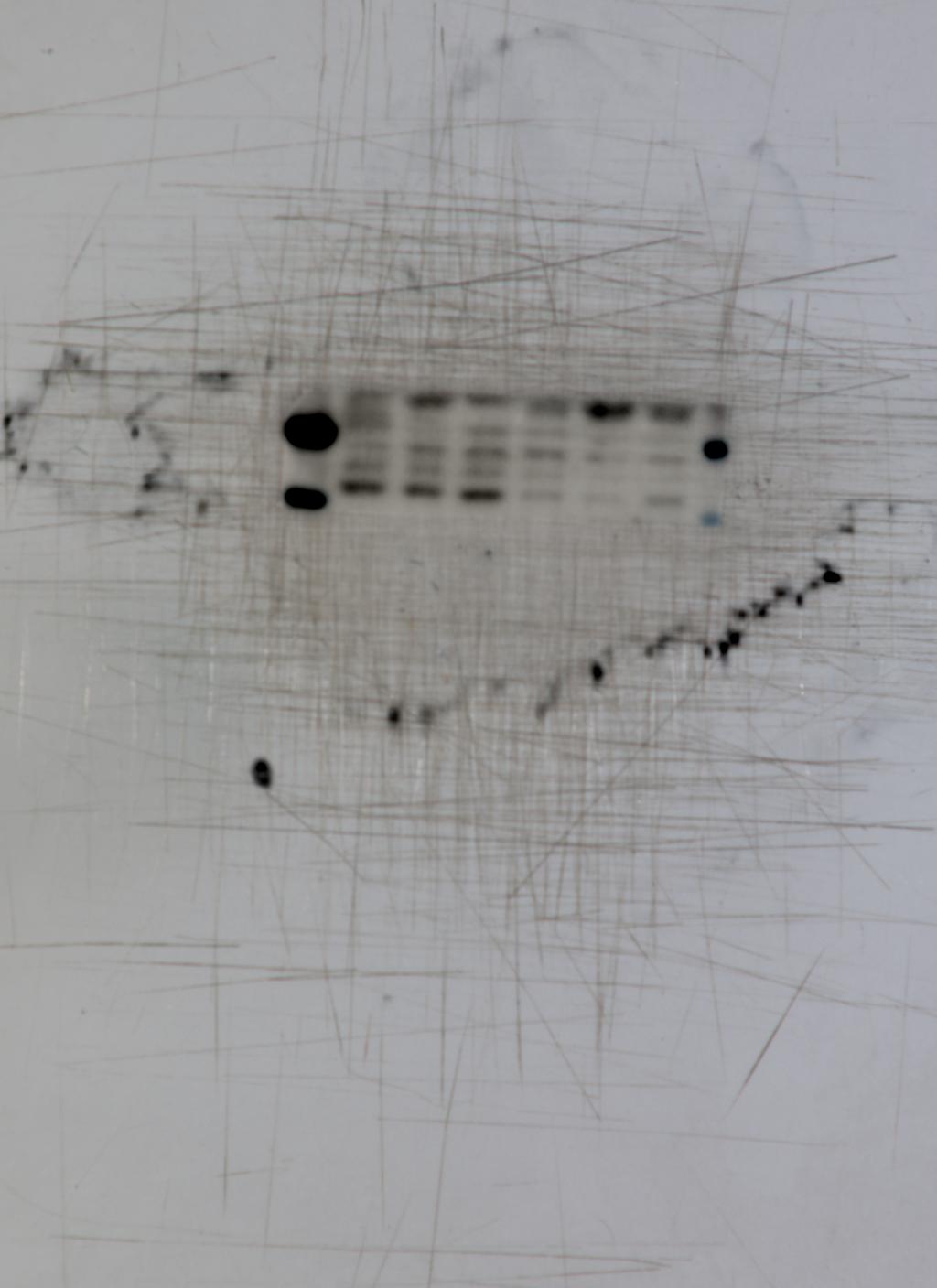


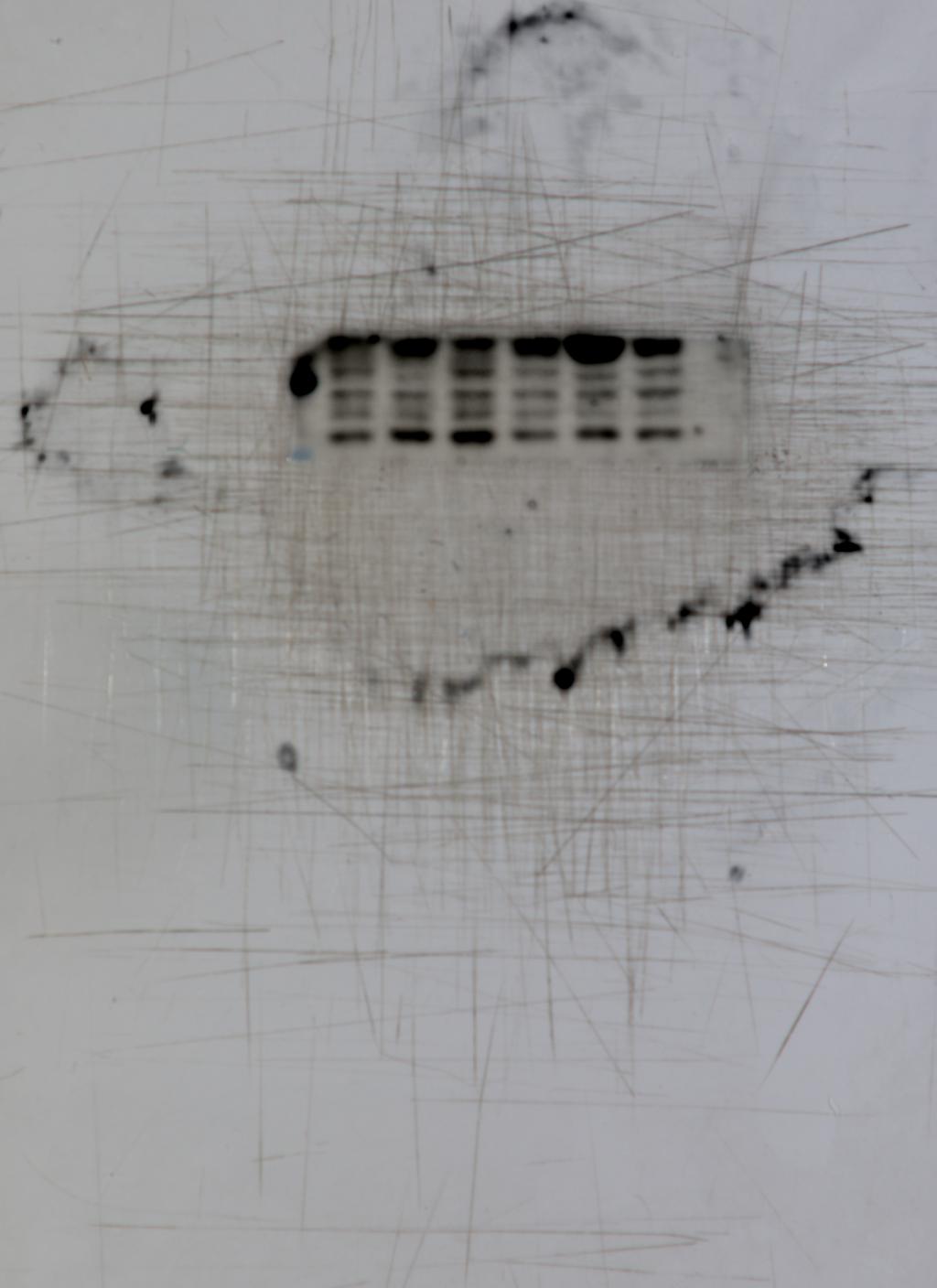


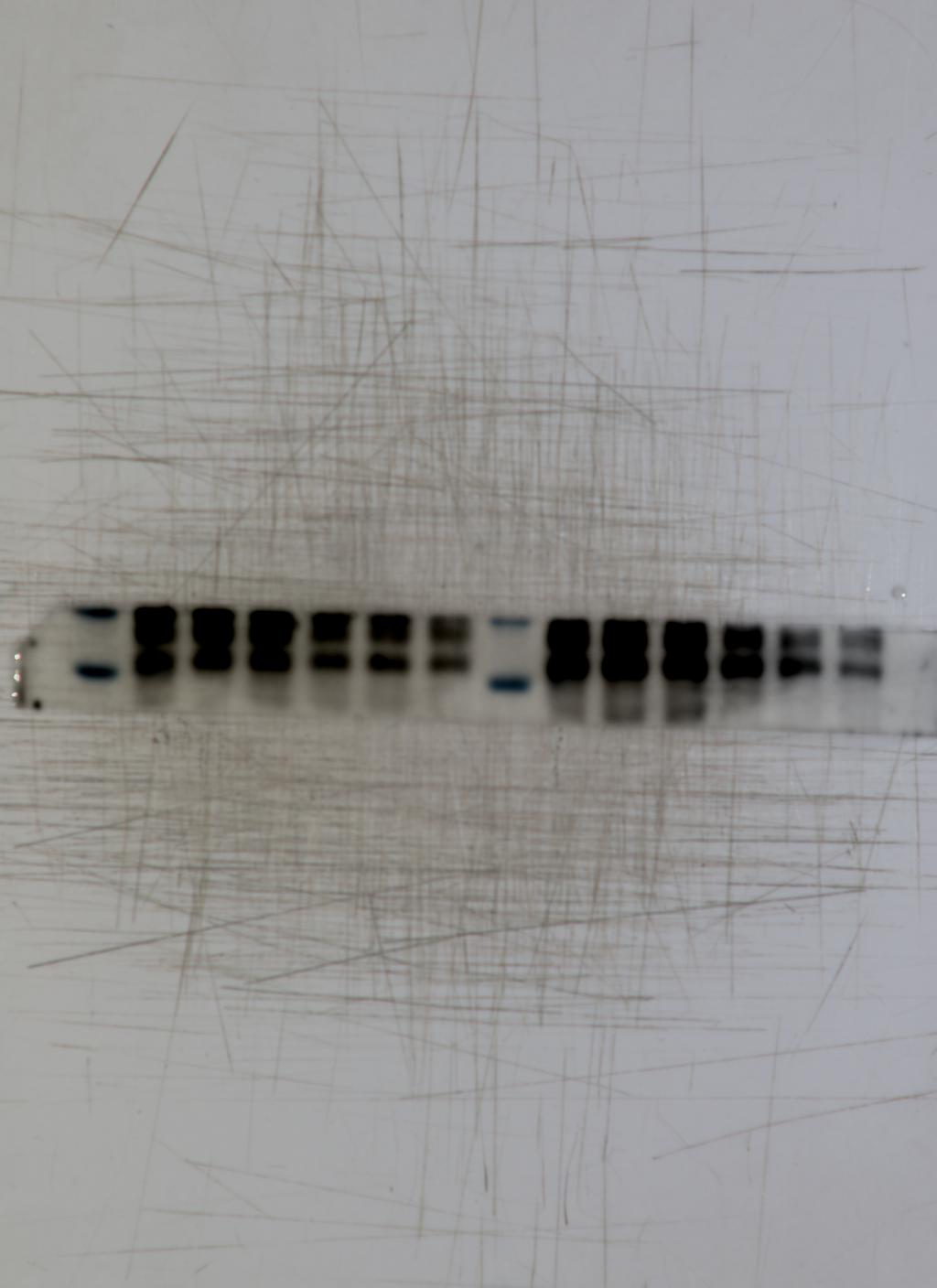


ARG-1


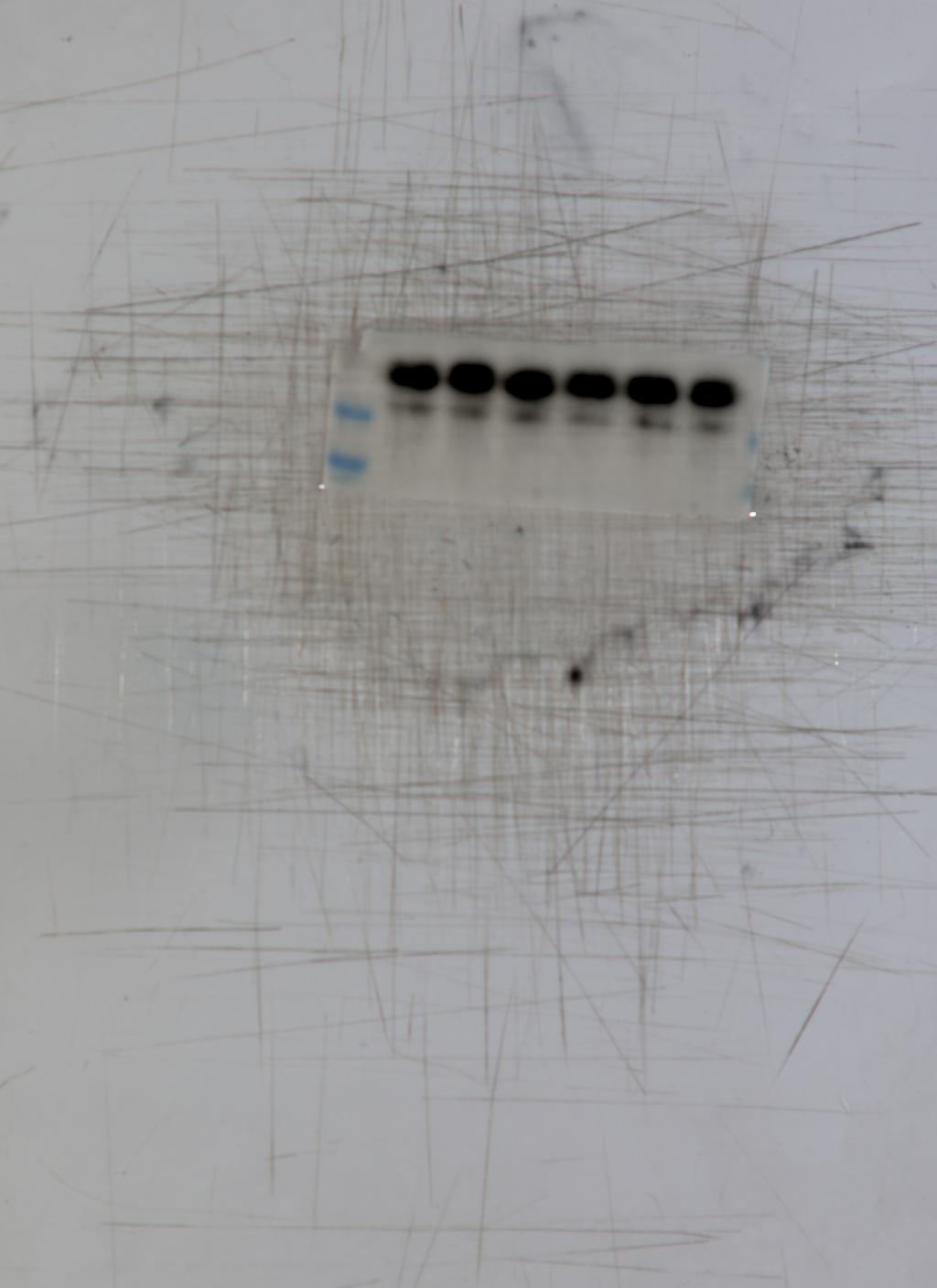


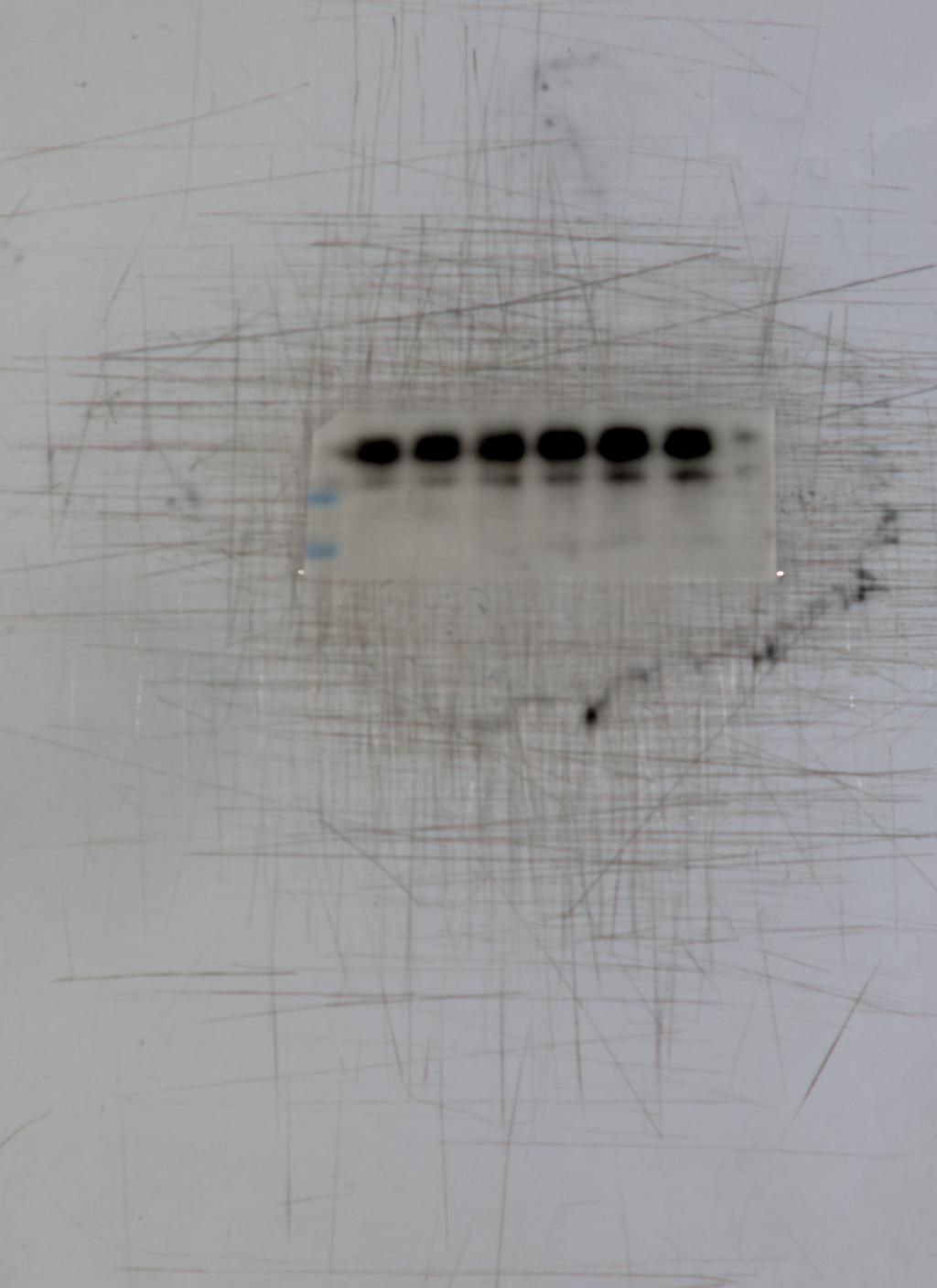


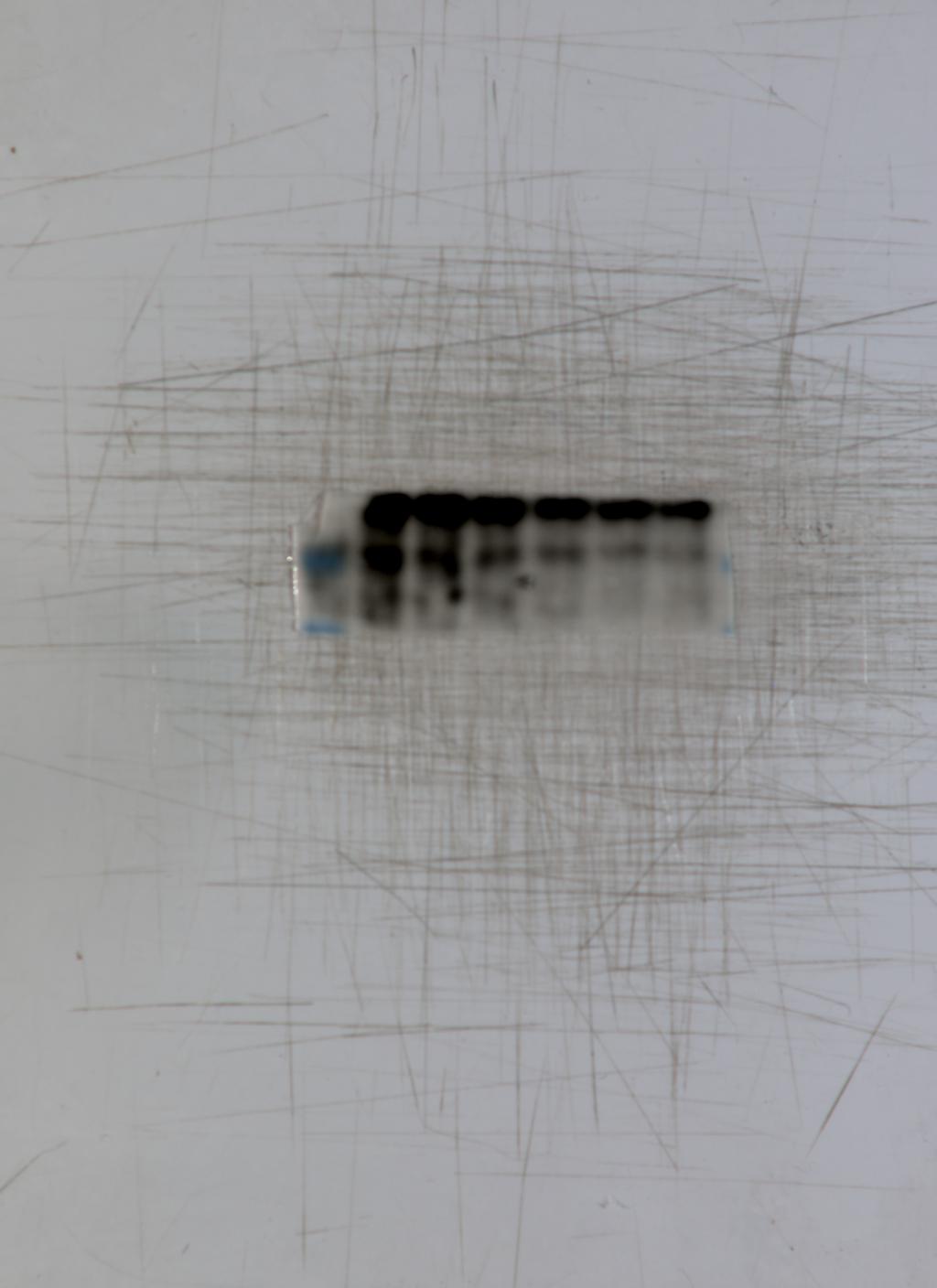


P65


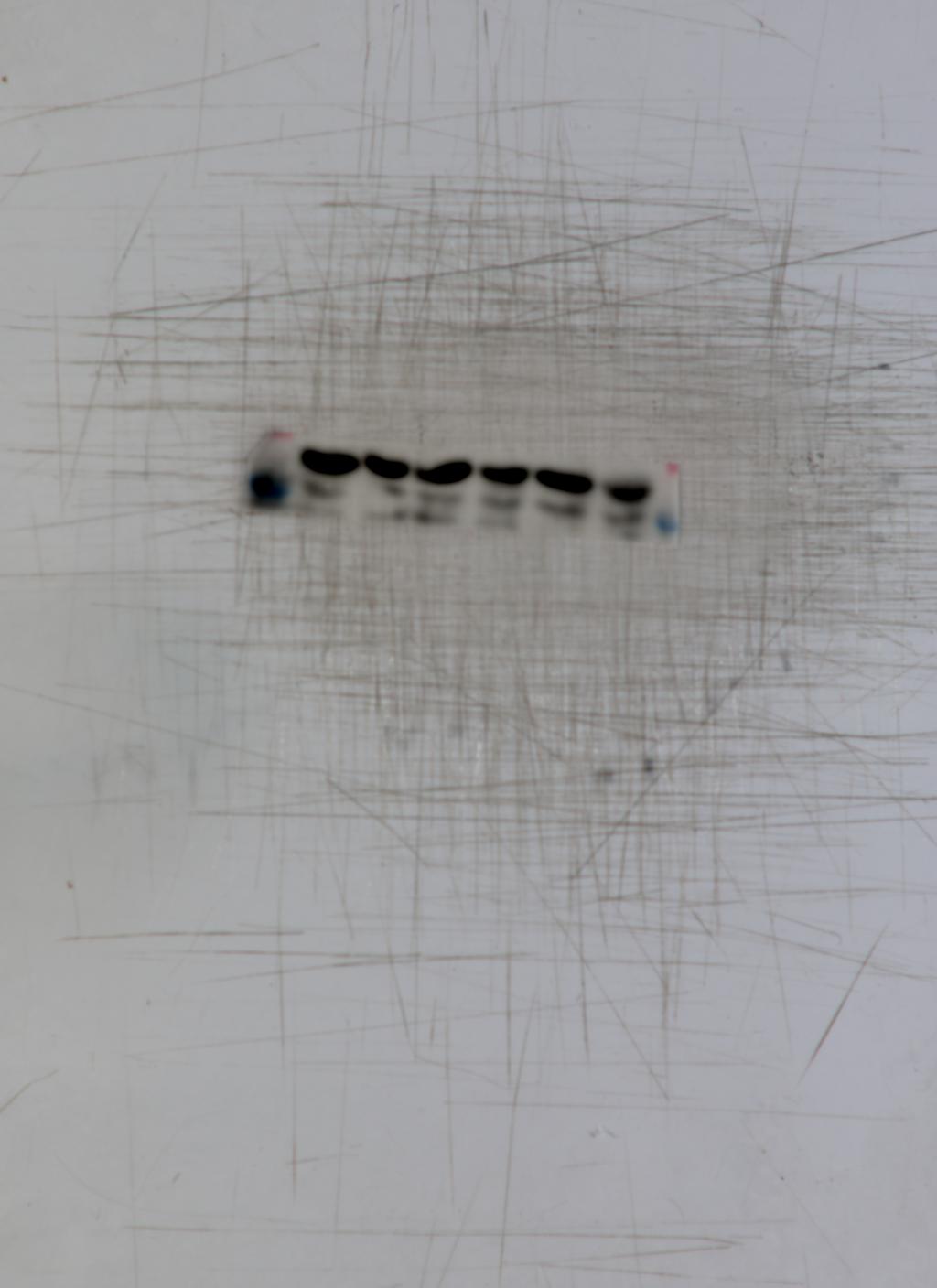


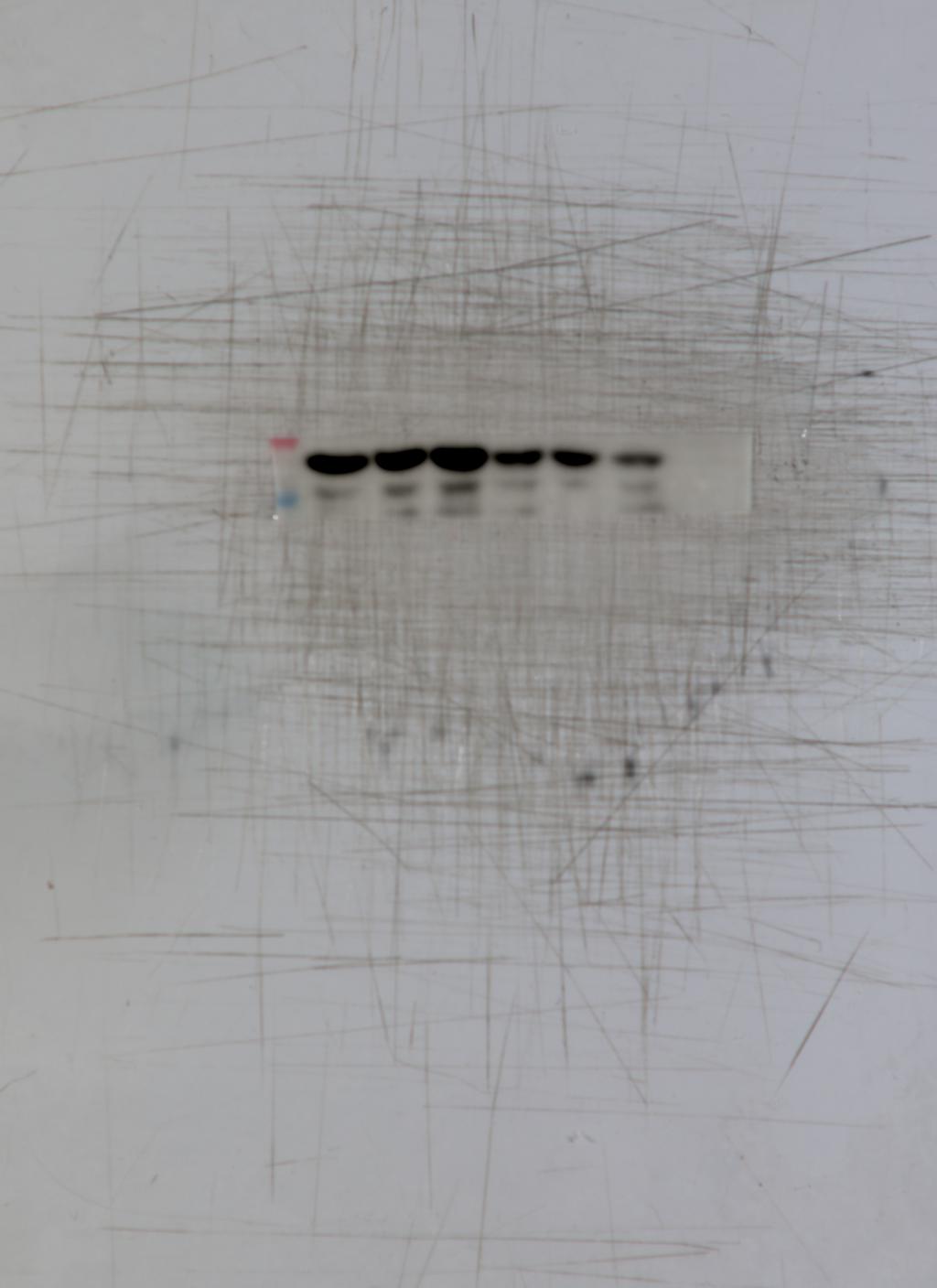


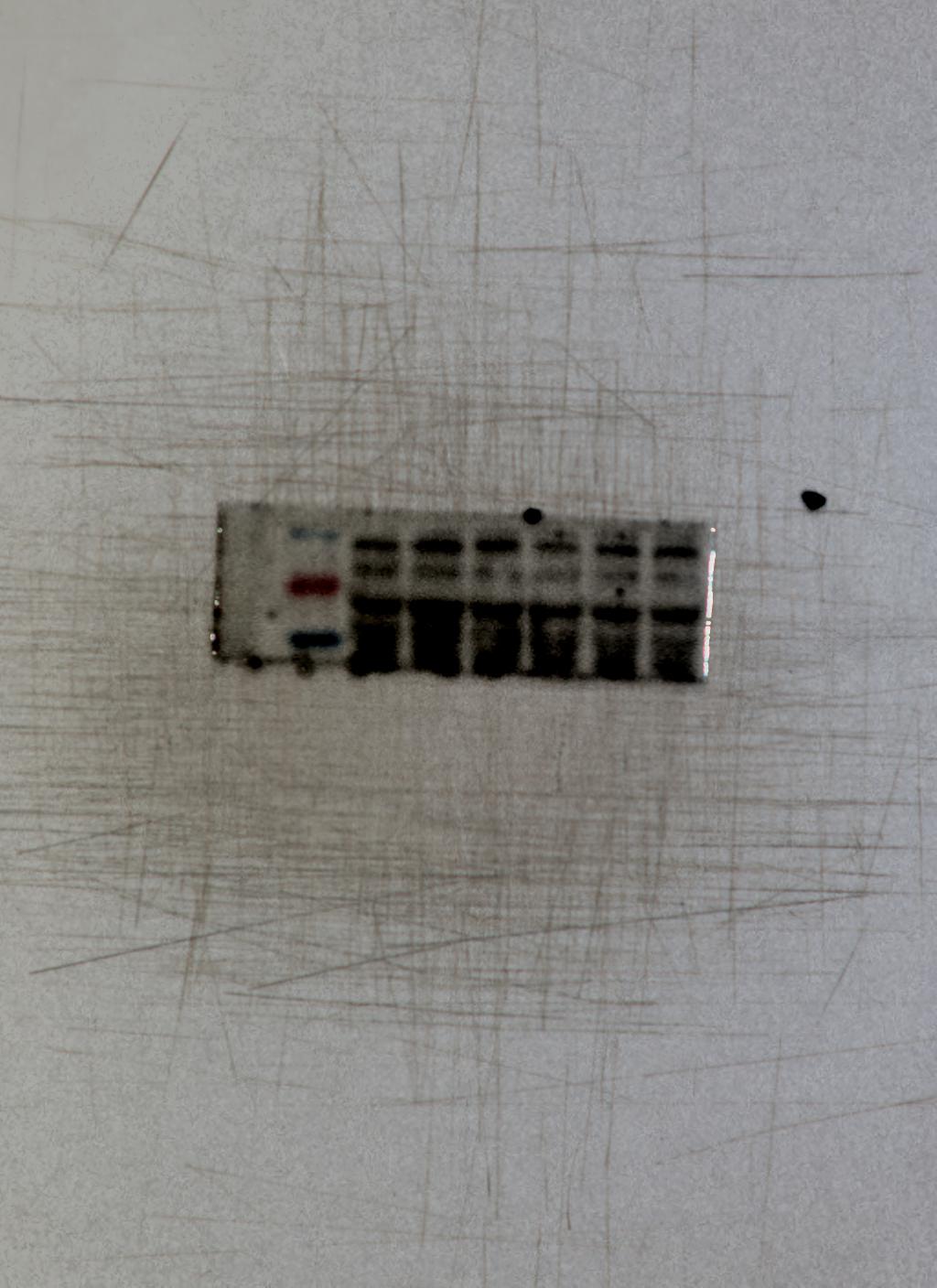


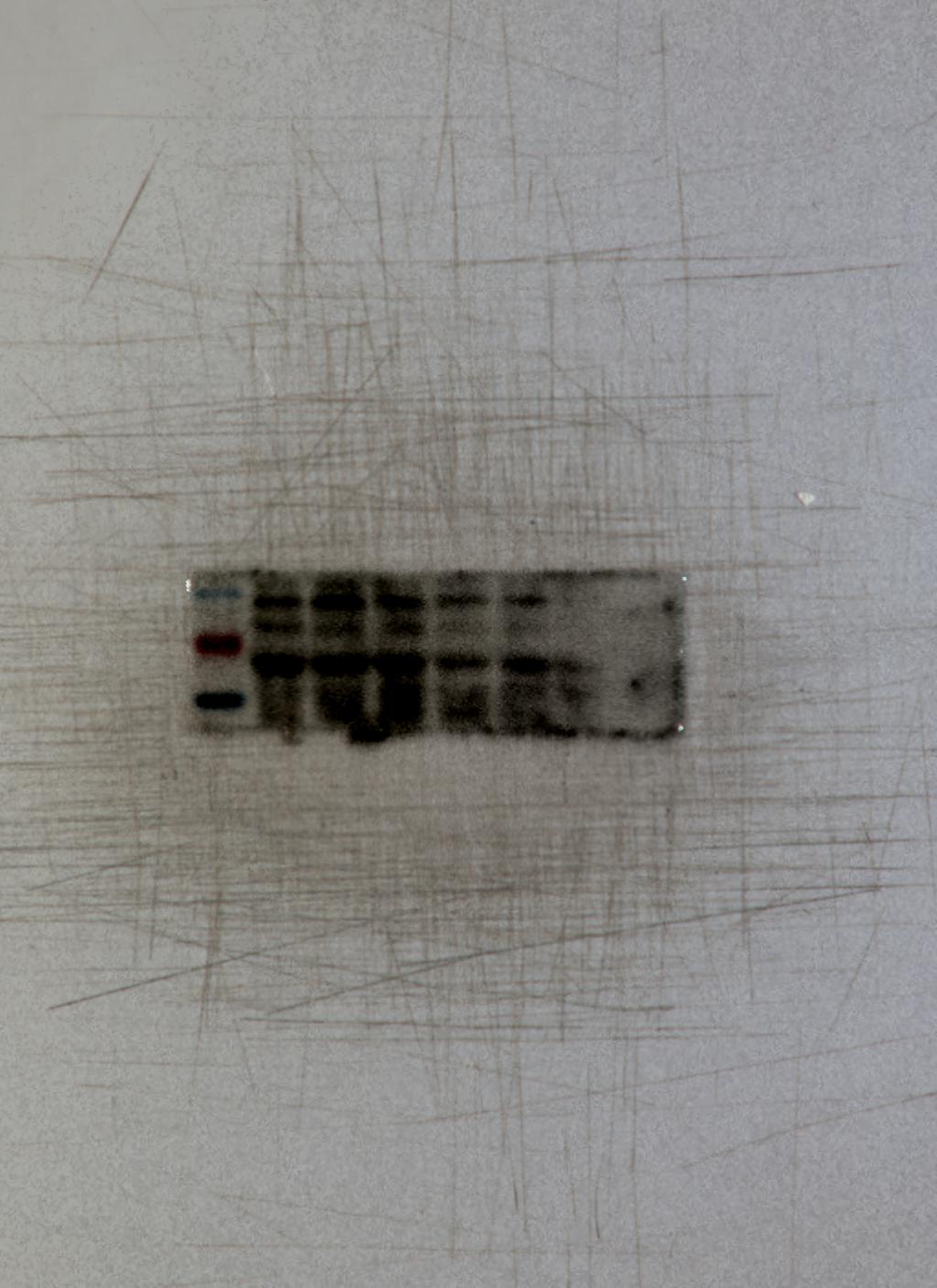


p-p65


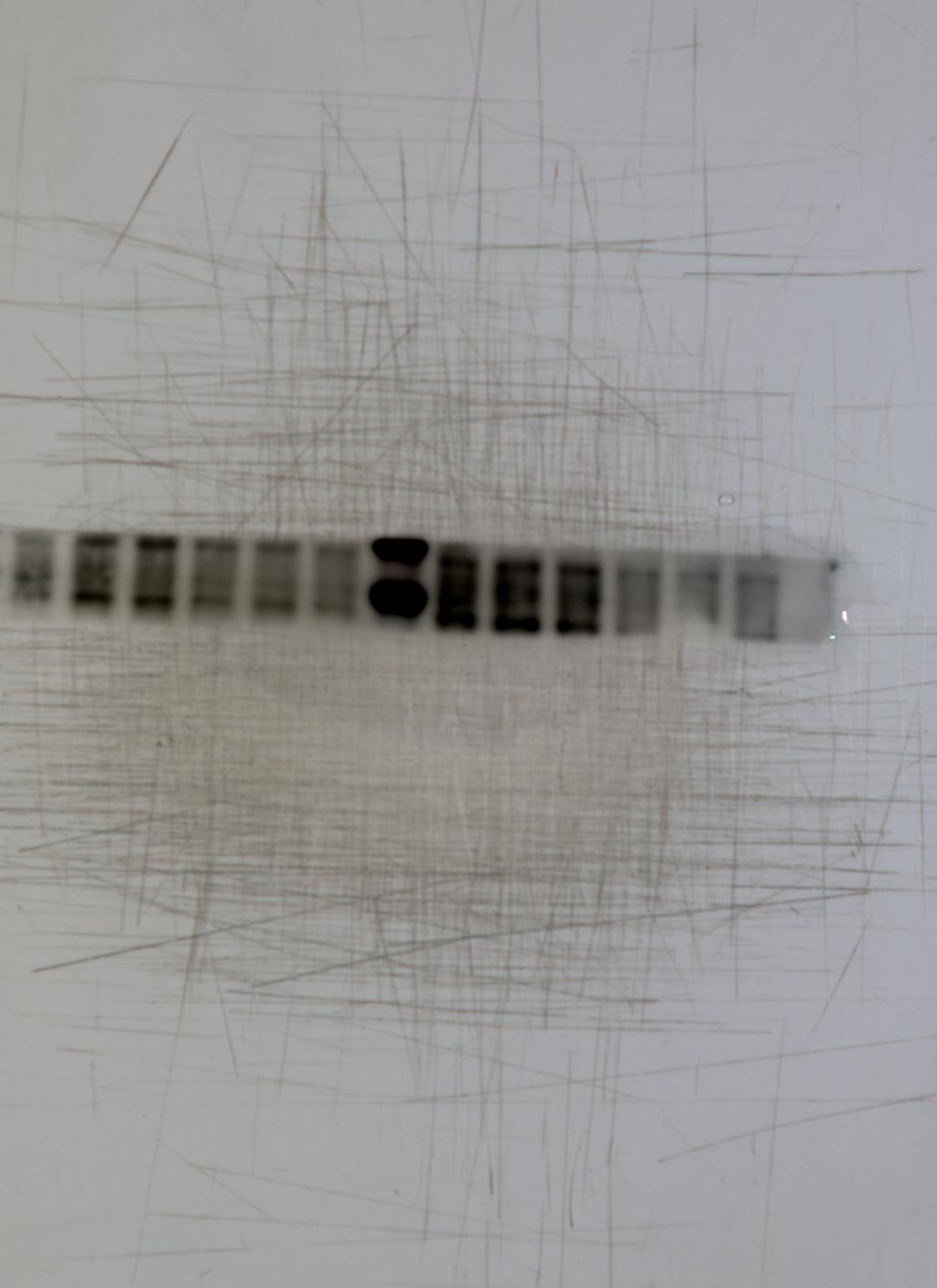


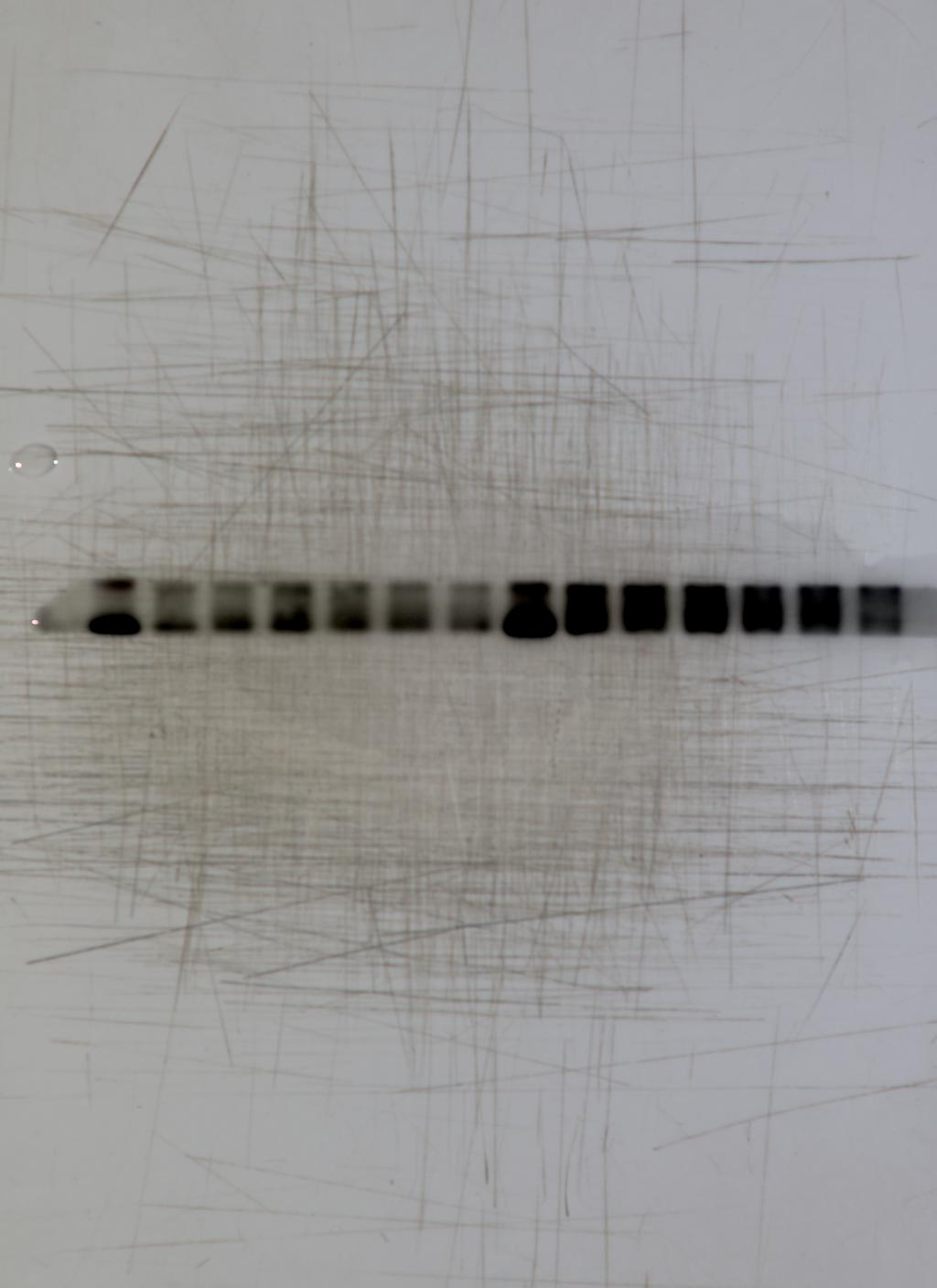


Figure 5A

IGFBP5 INPUT


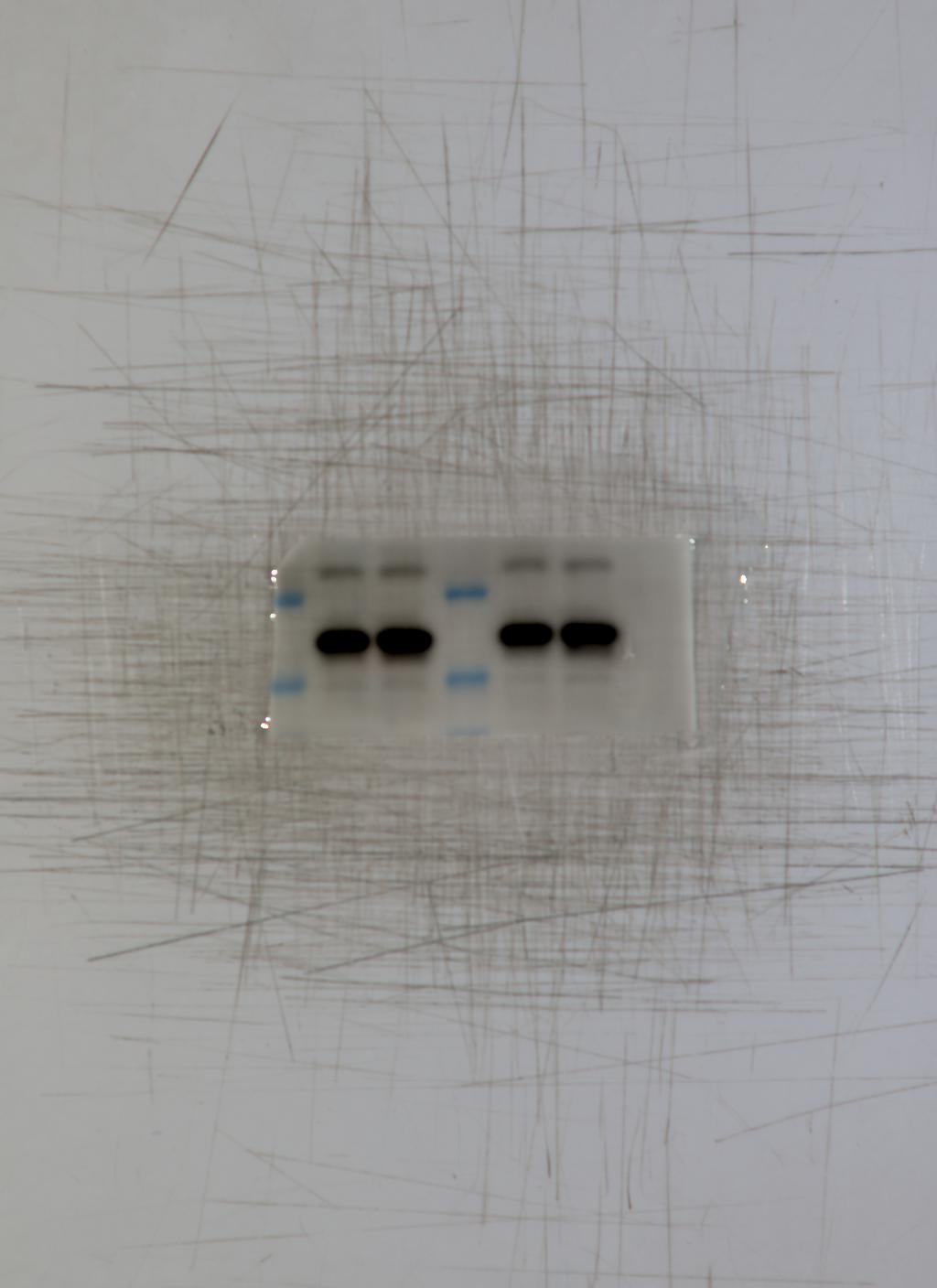


ANXA2 INPUT IP


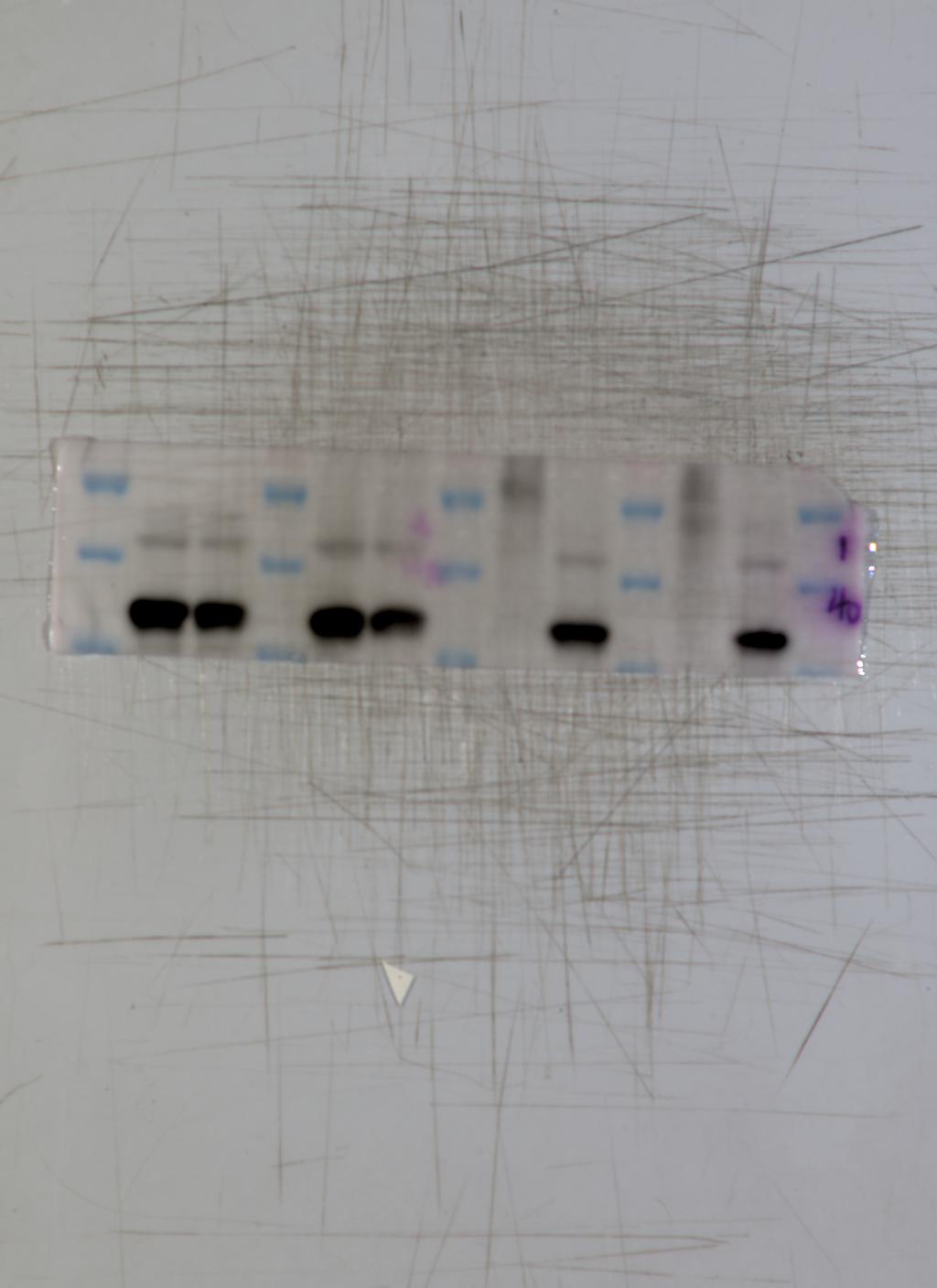


Figure 5B

IGFBP5 INPUT


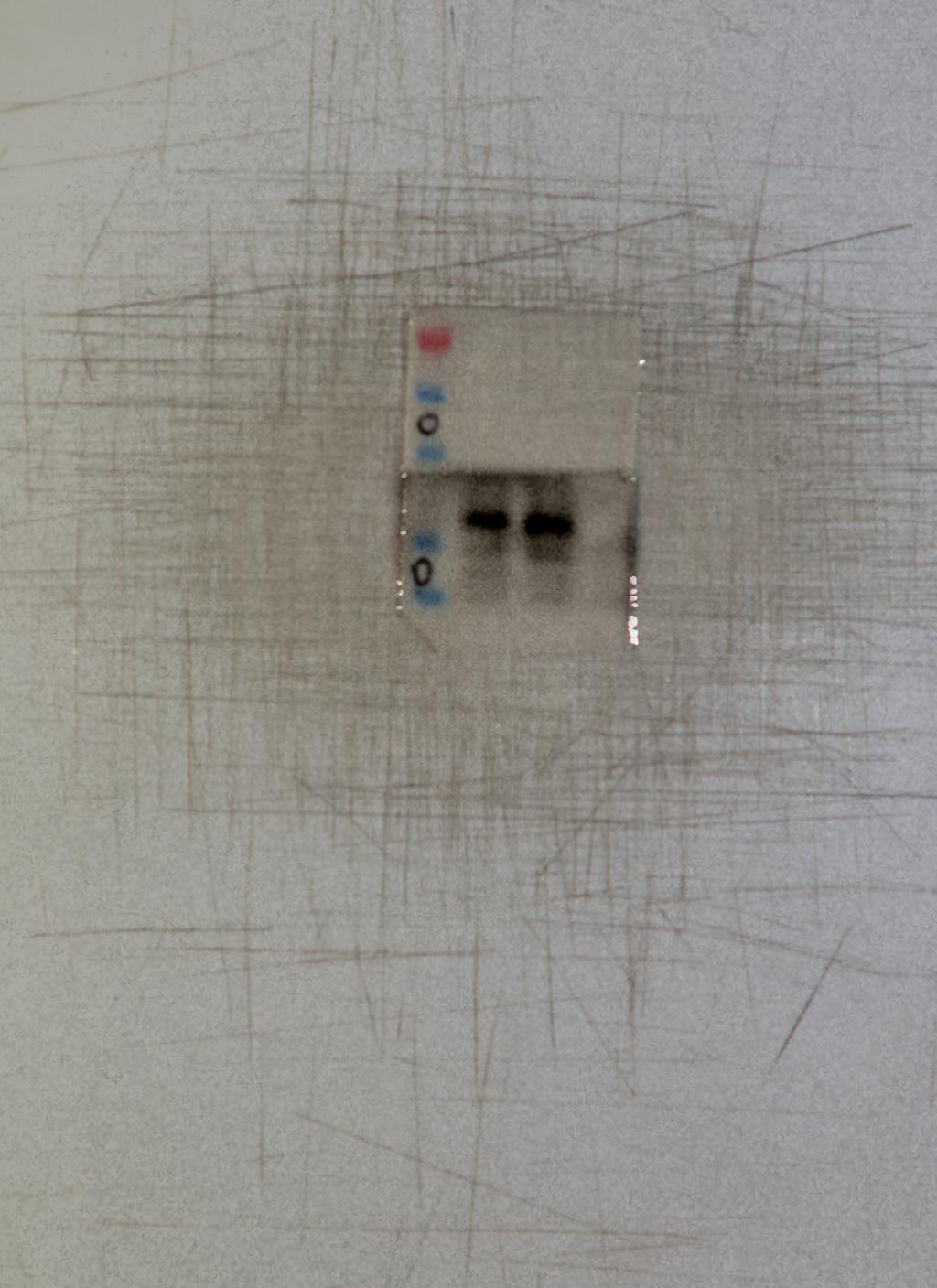


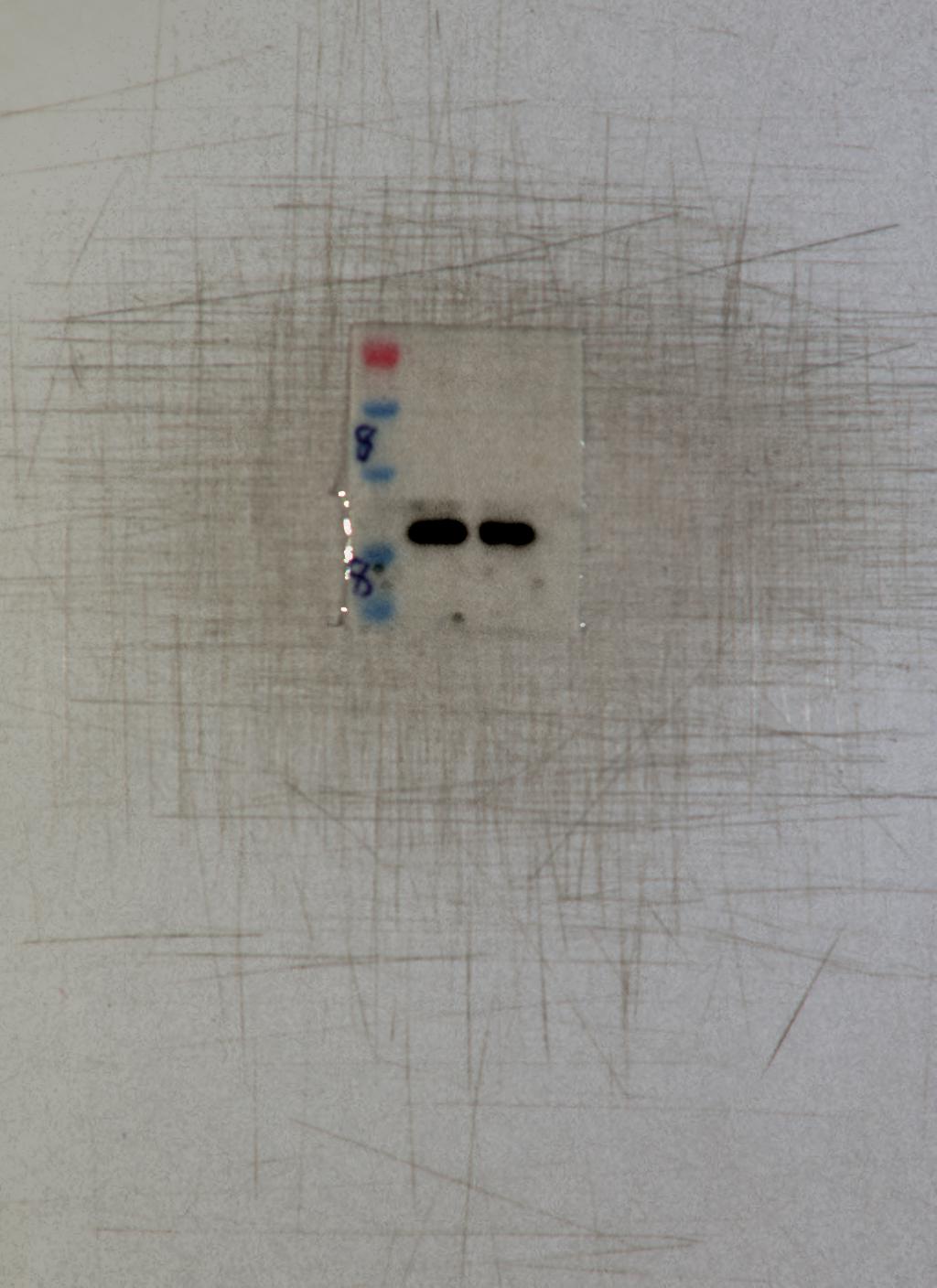


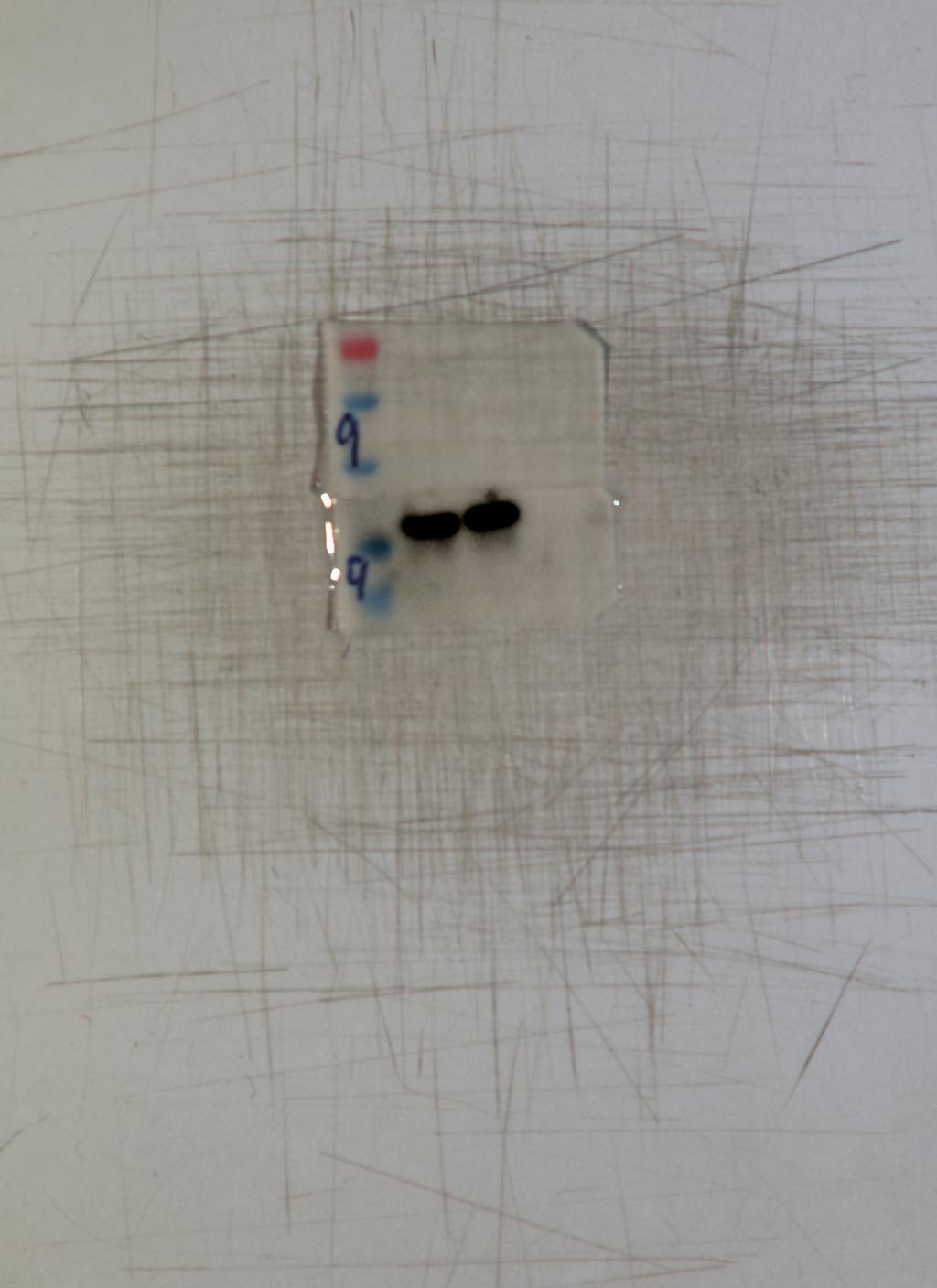


ANXA2 INPUT


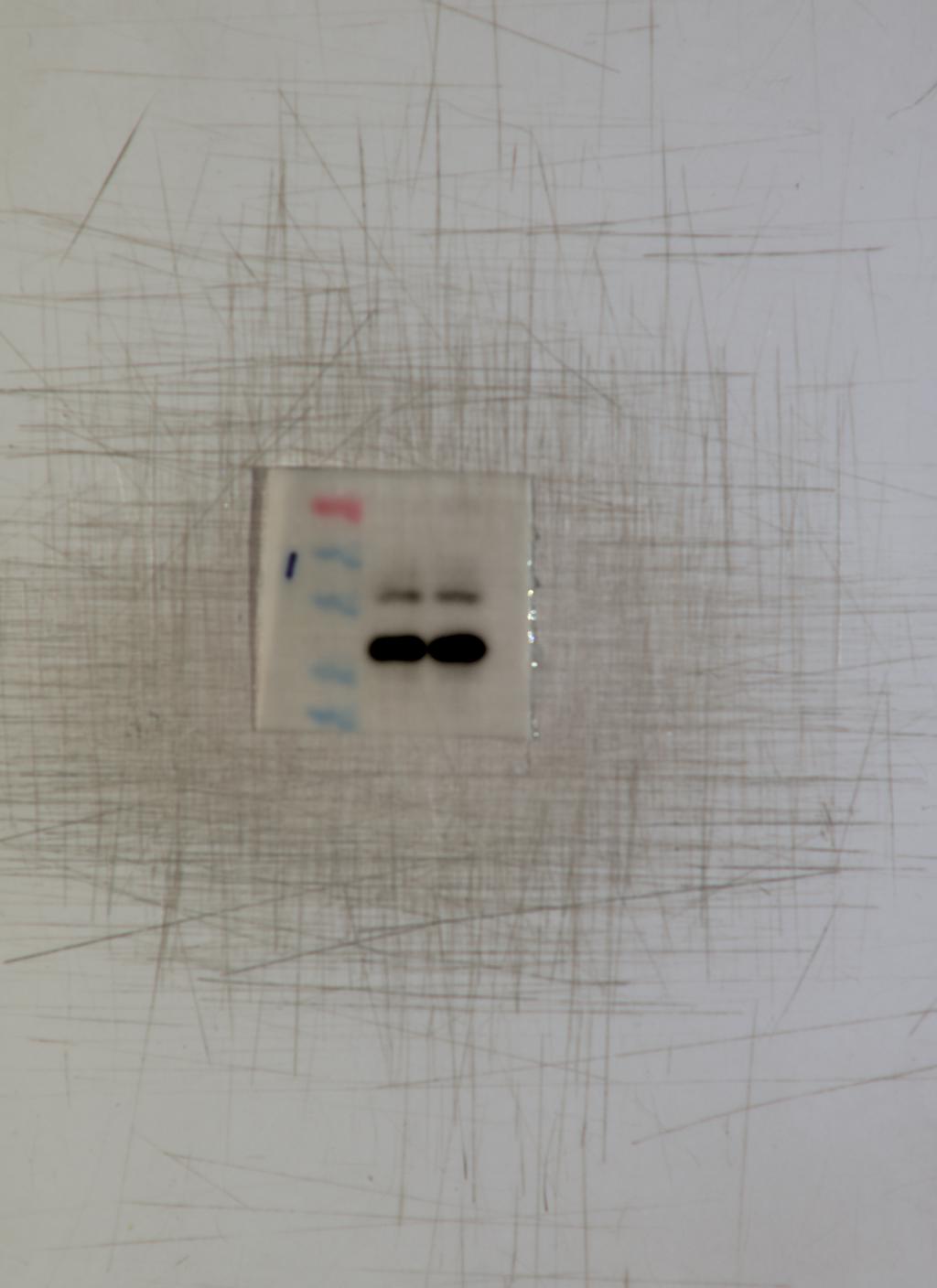

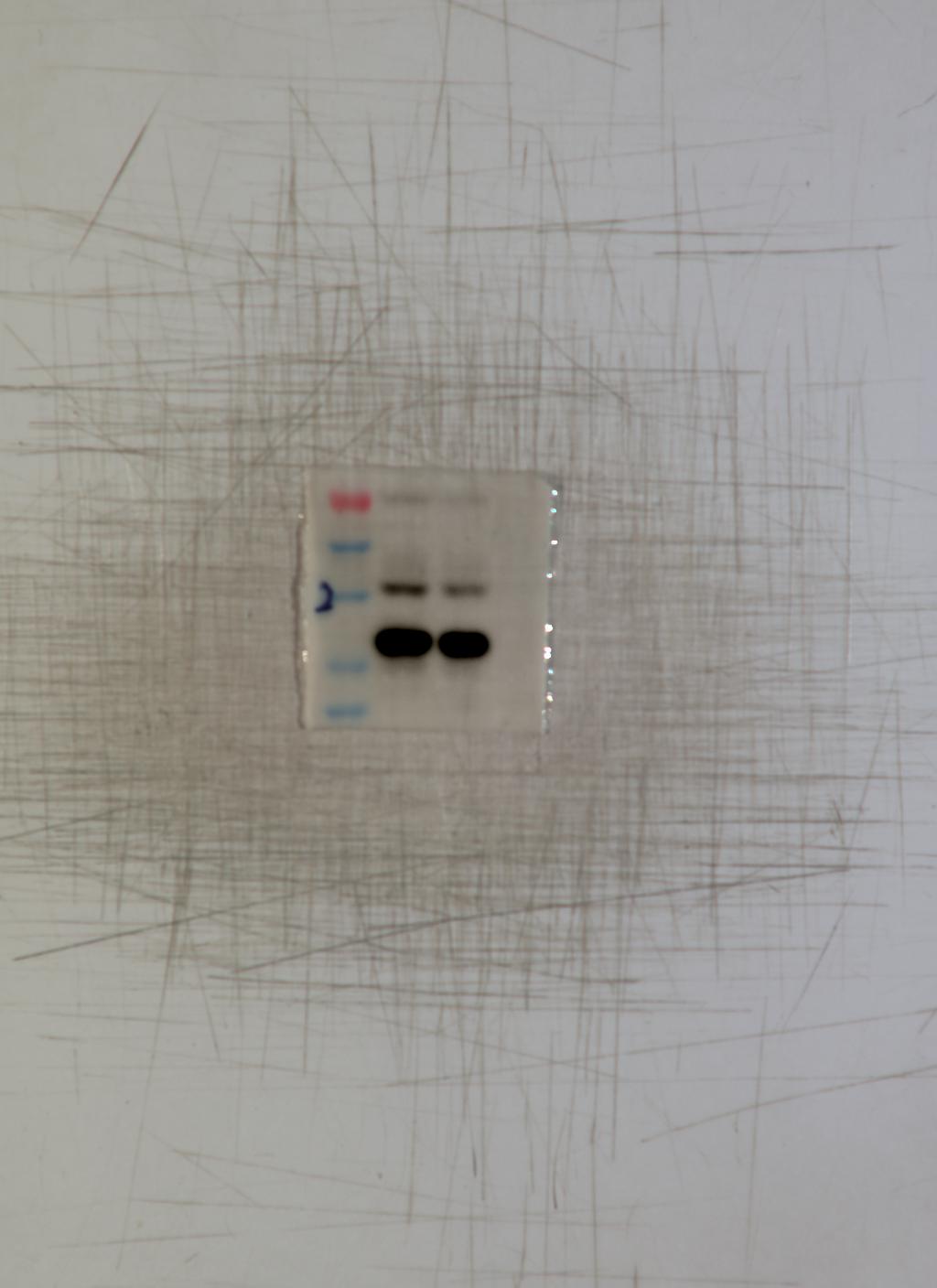

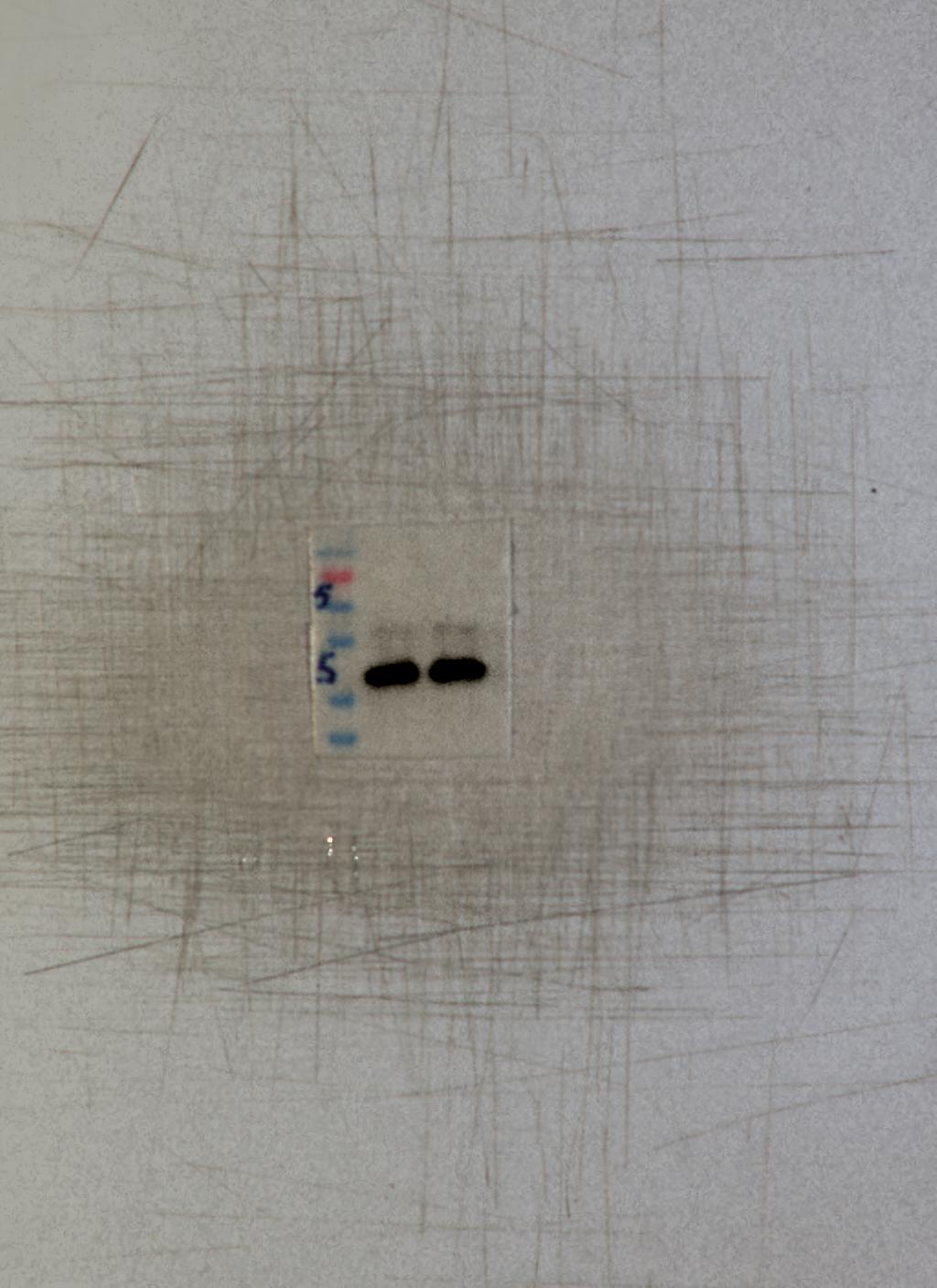


IGFBP5 IP


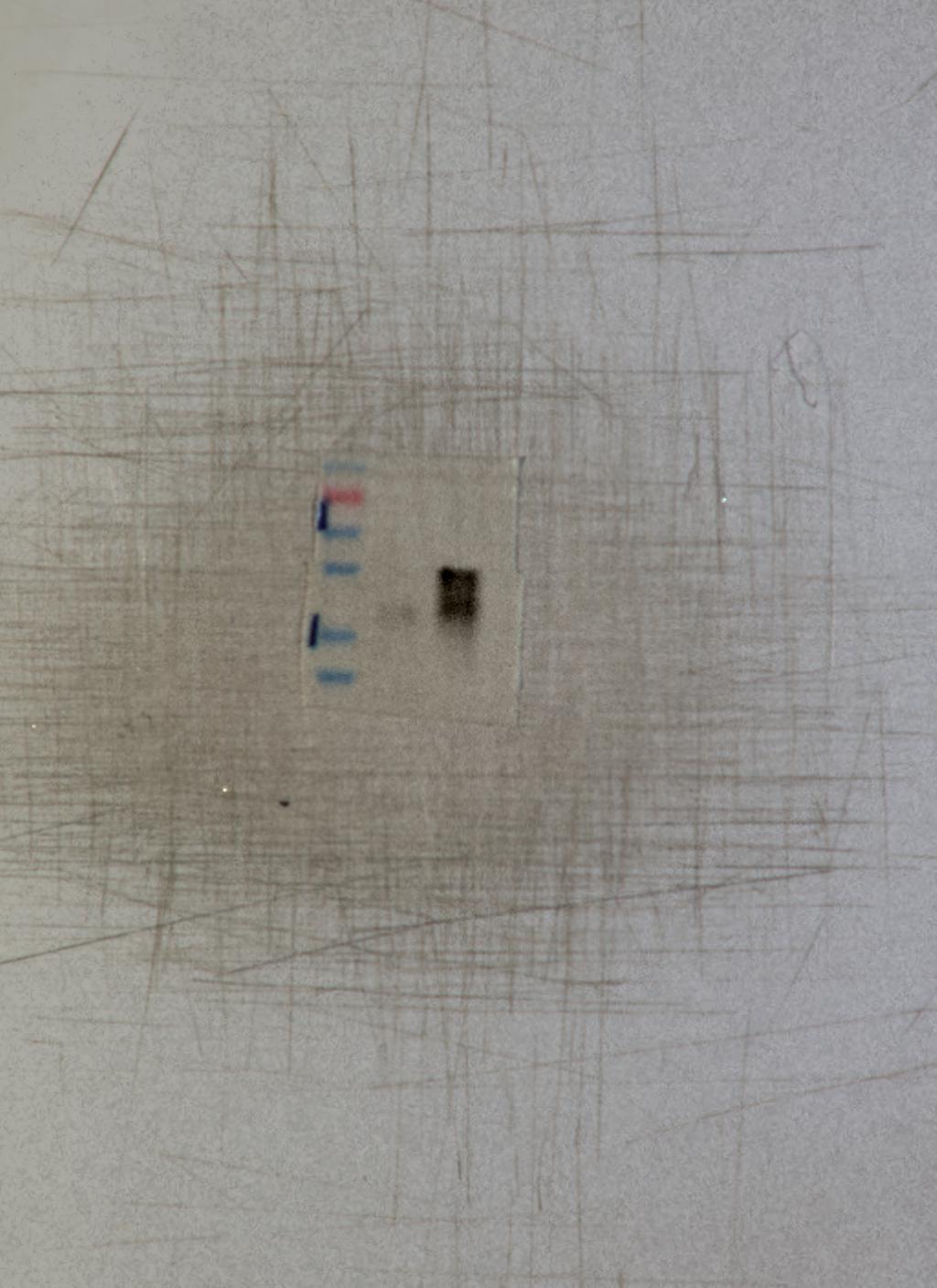


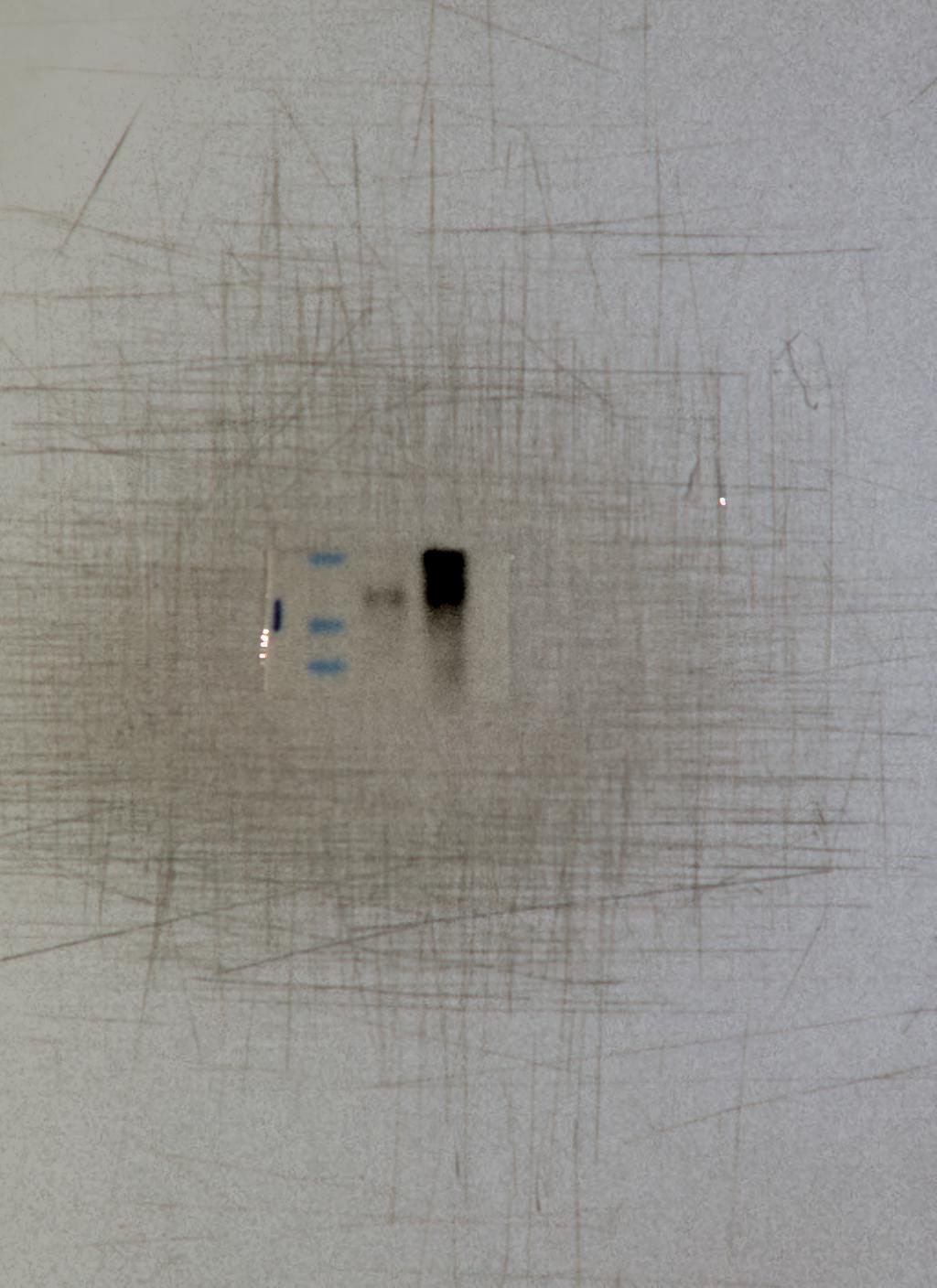

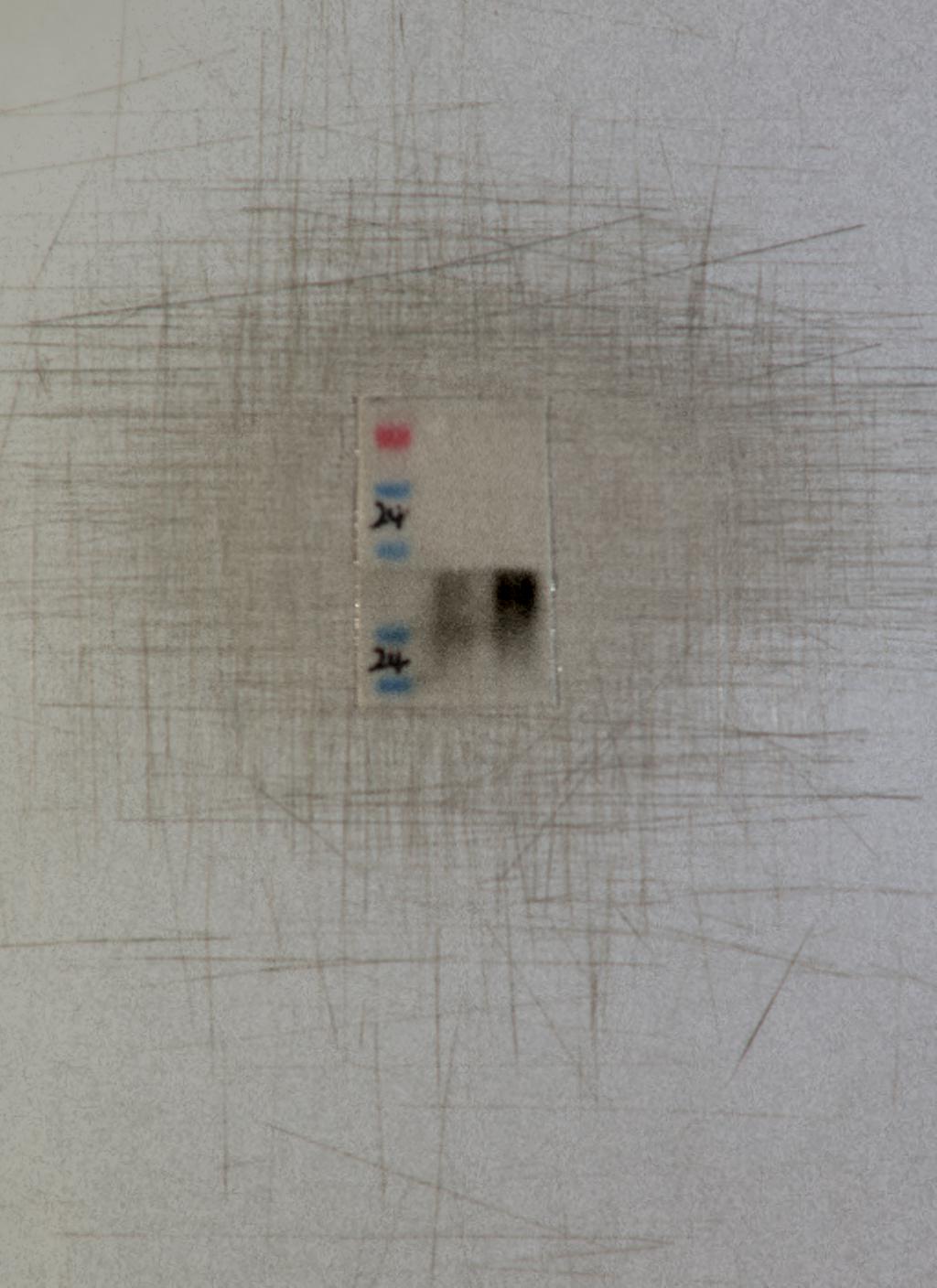


Figure 5C

TLR4 INPUT


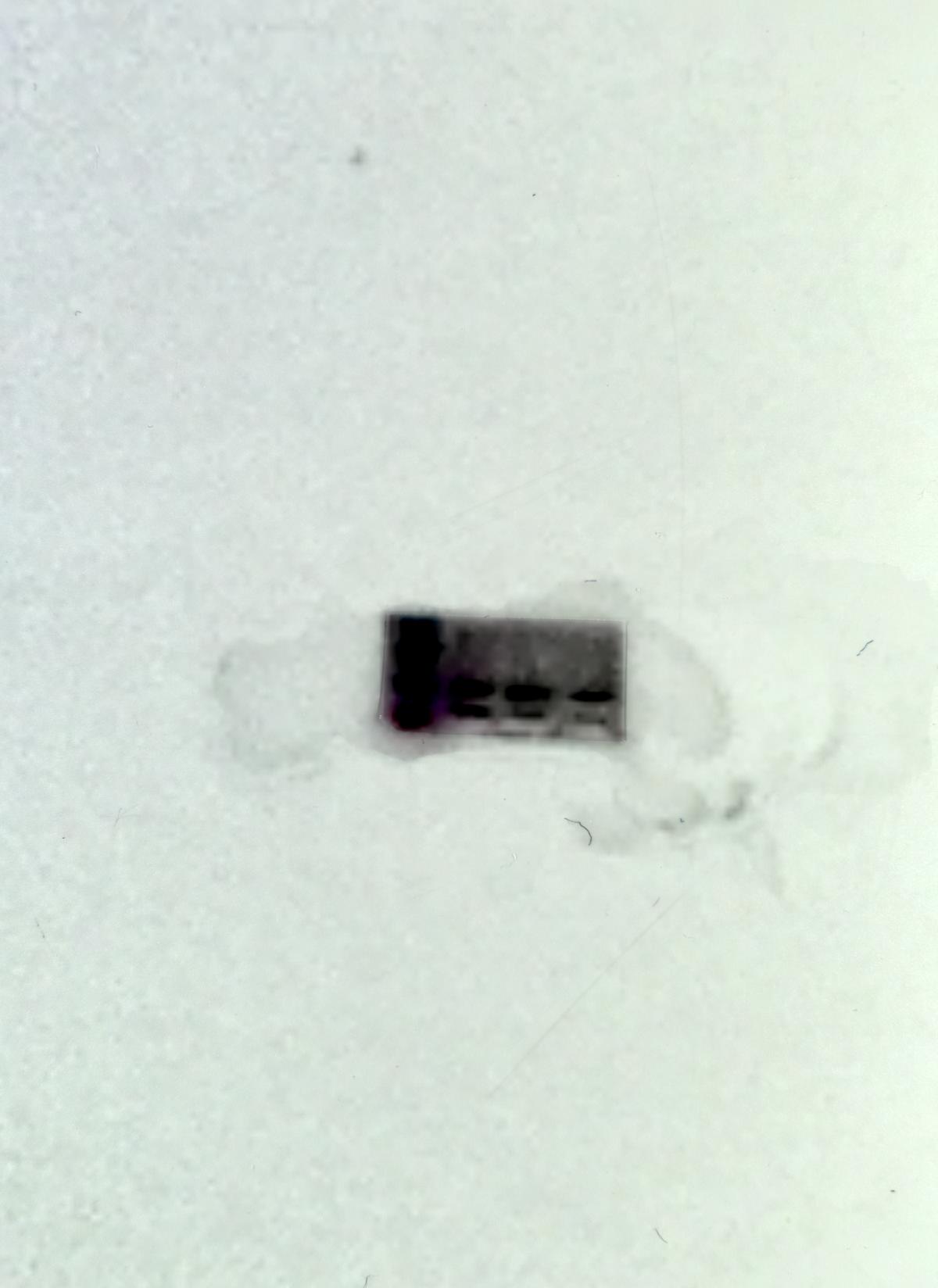


ANXA2 INPUT


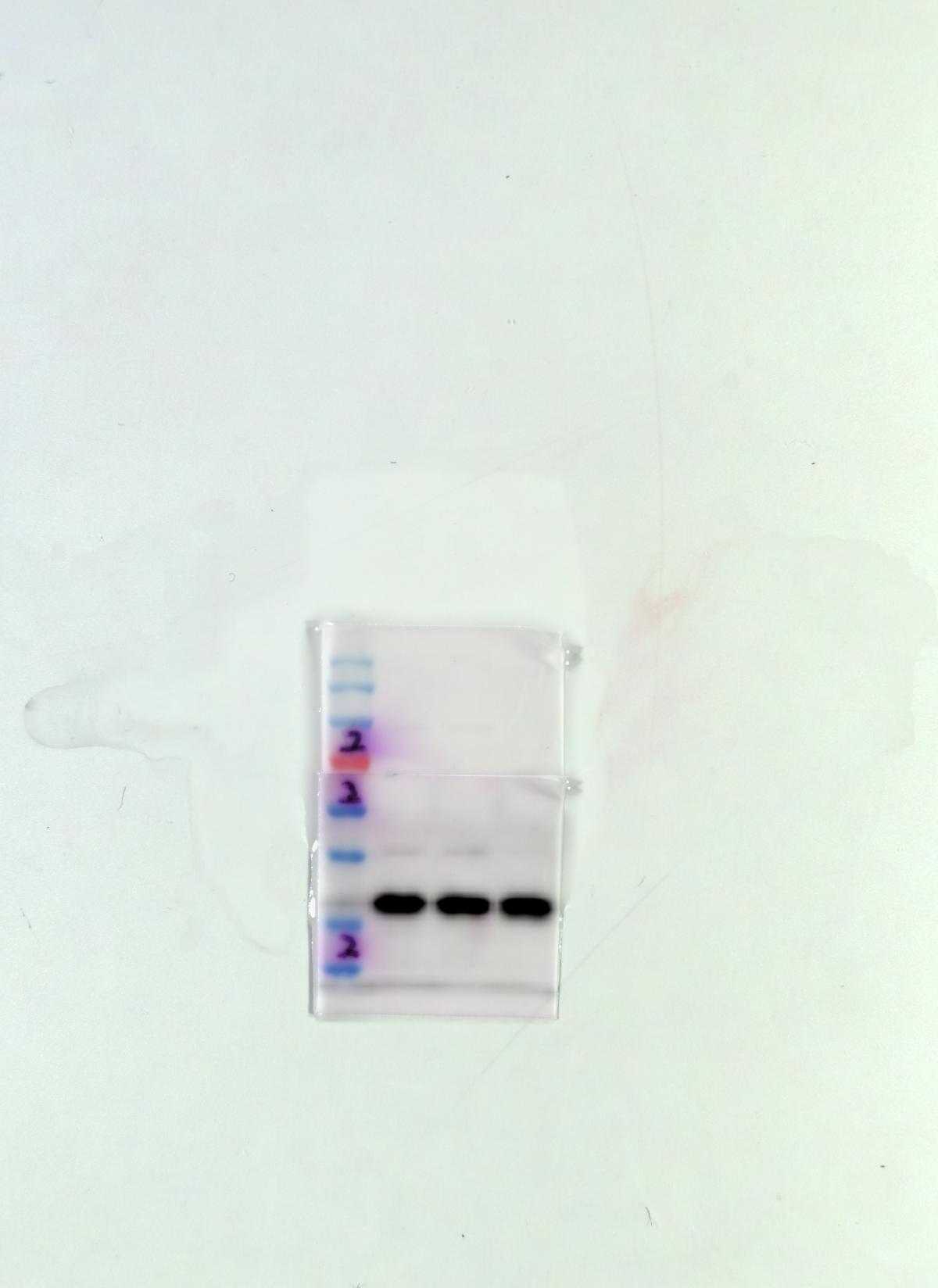

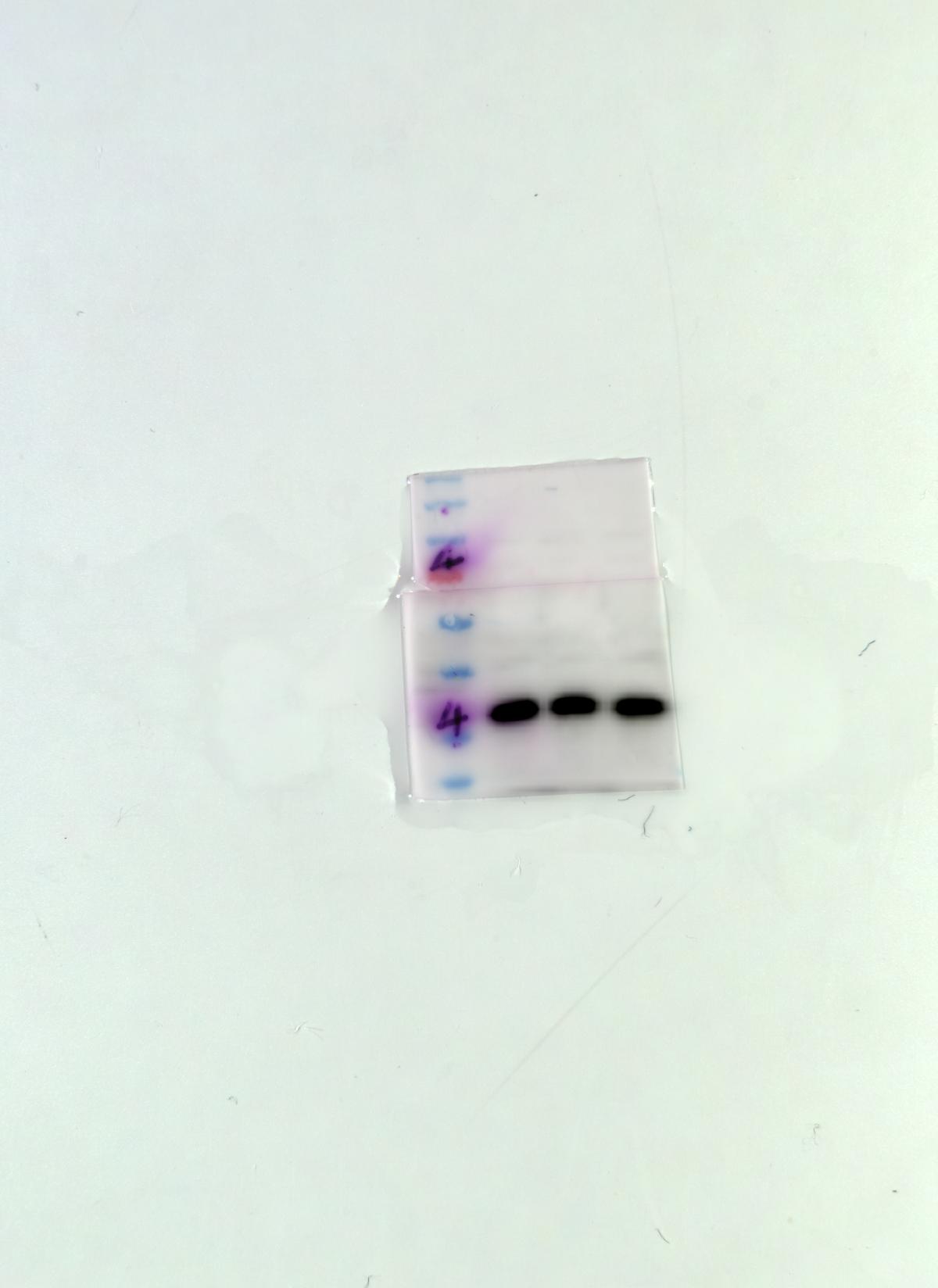


ANXA2 IP


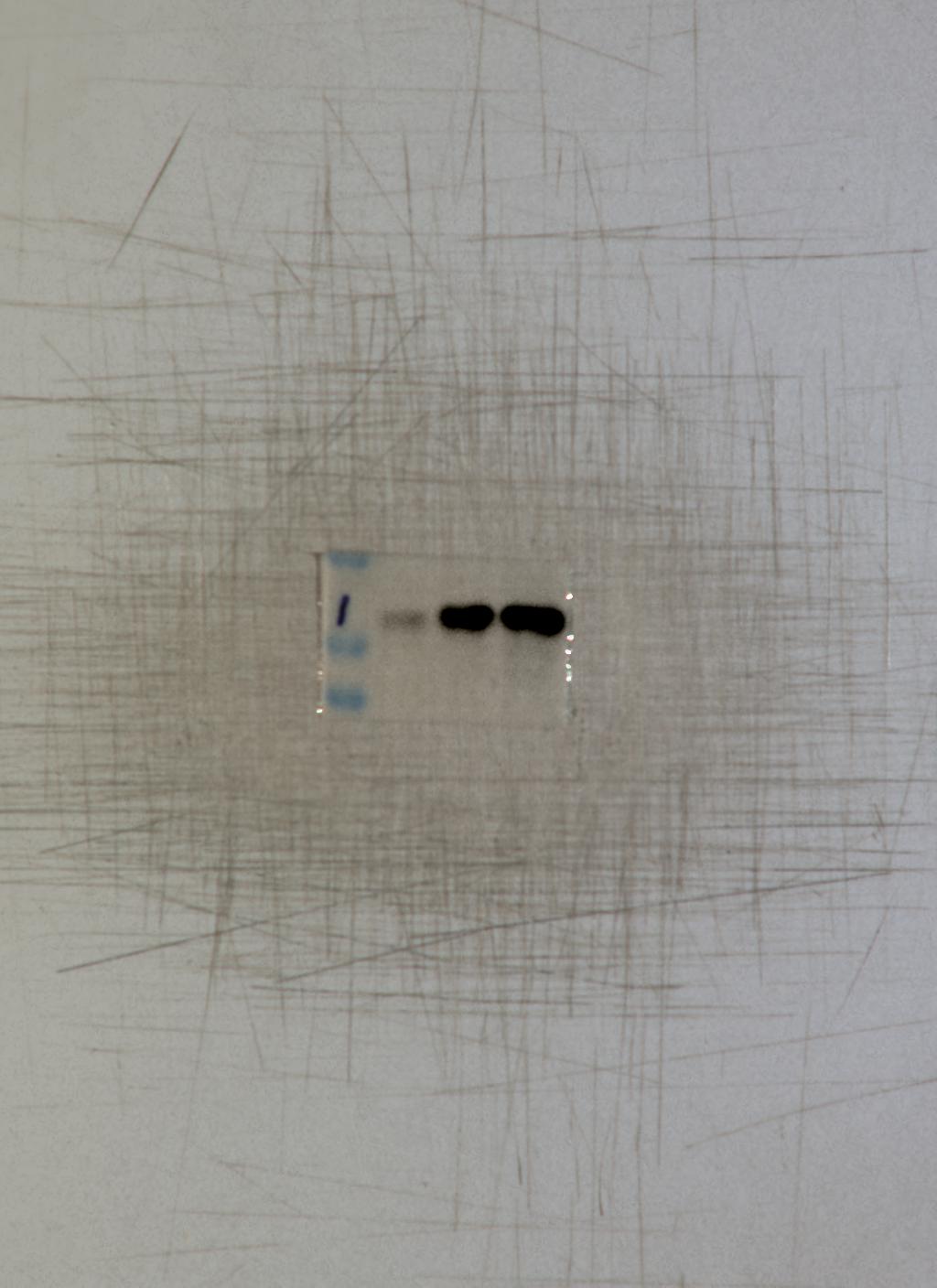


TLR4 IP


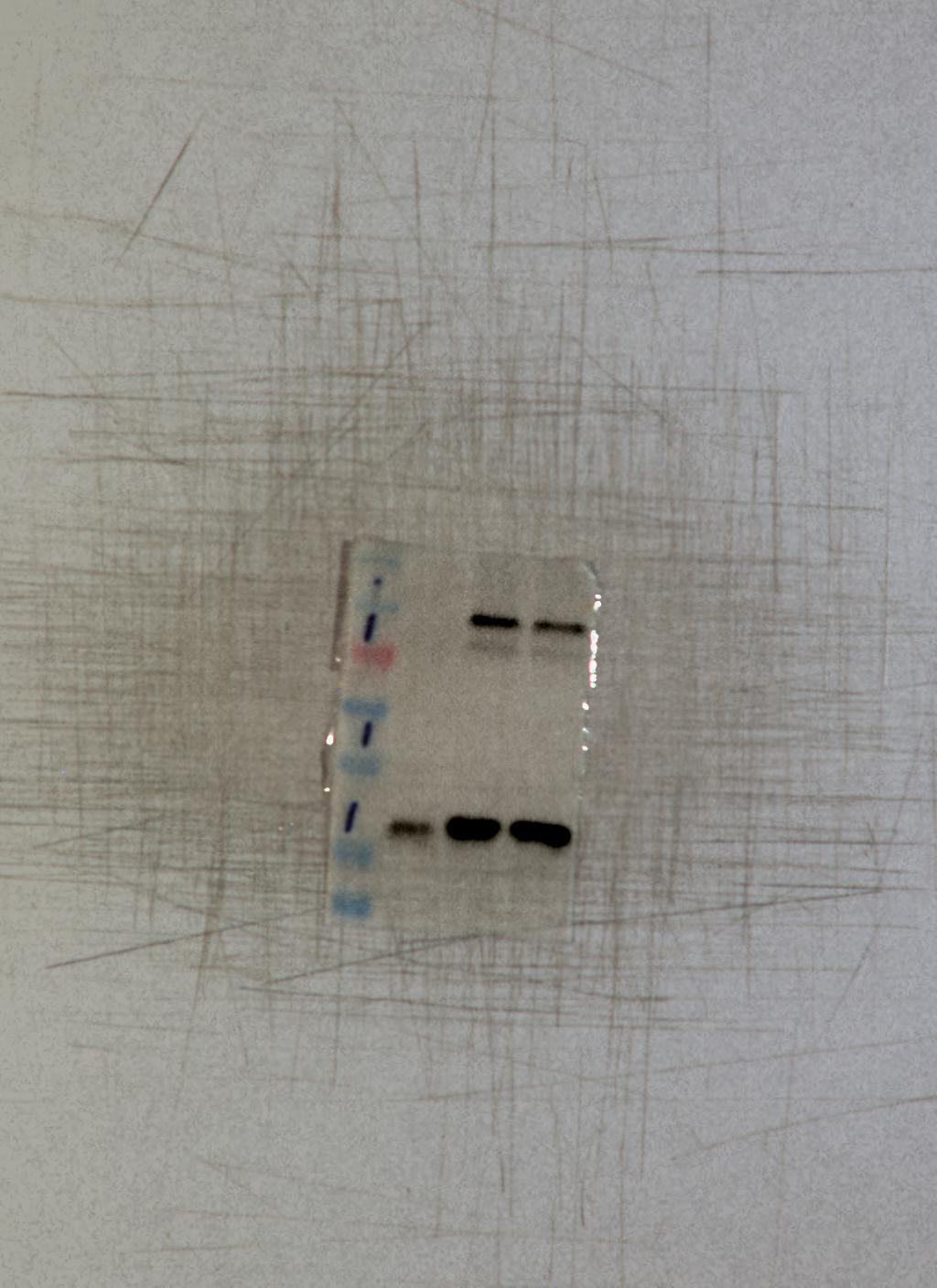


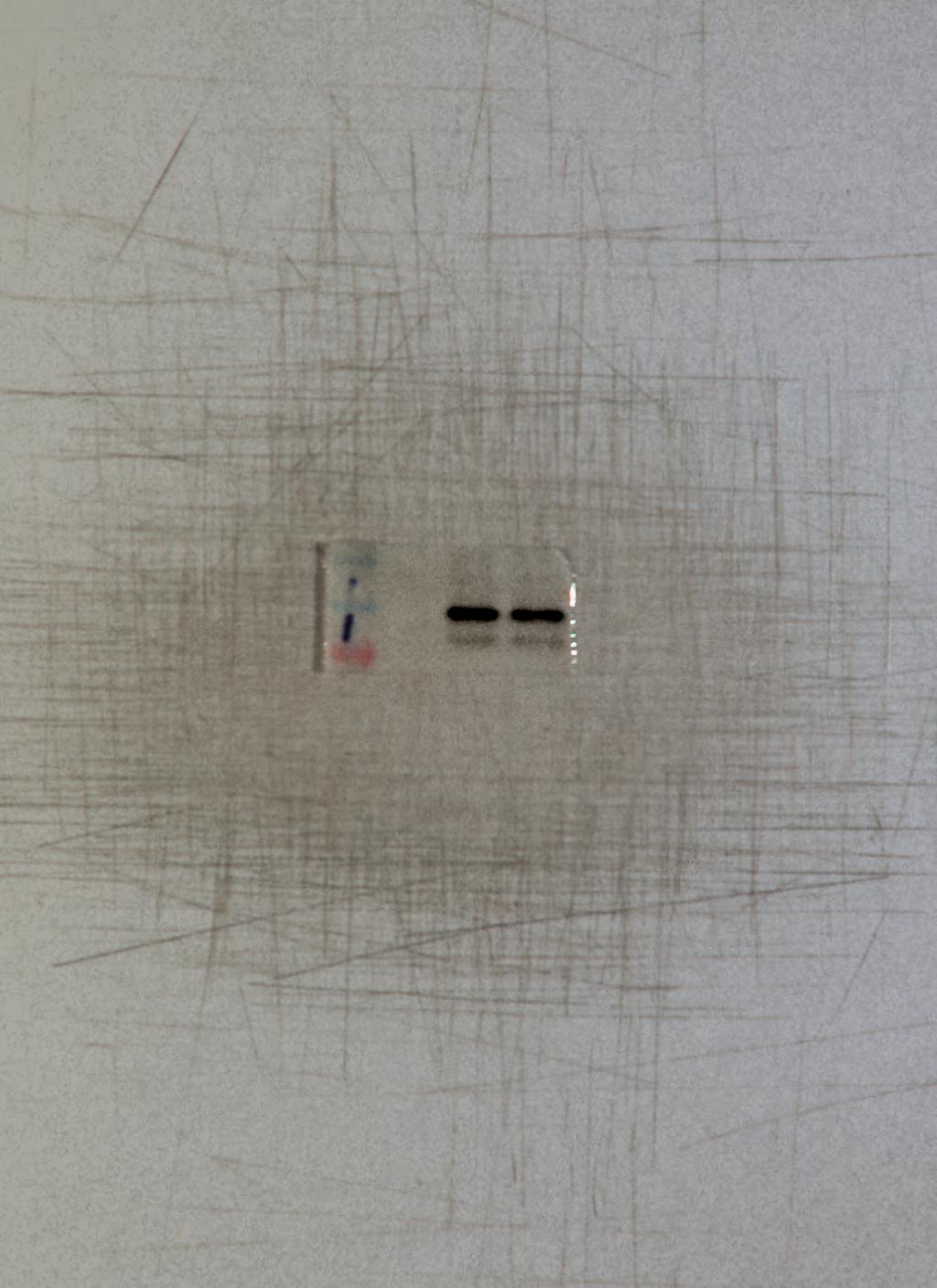


Figure 5D

TLR4 INPUT


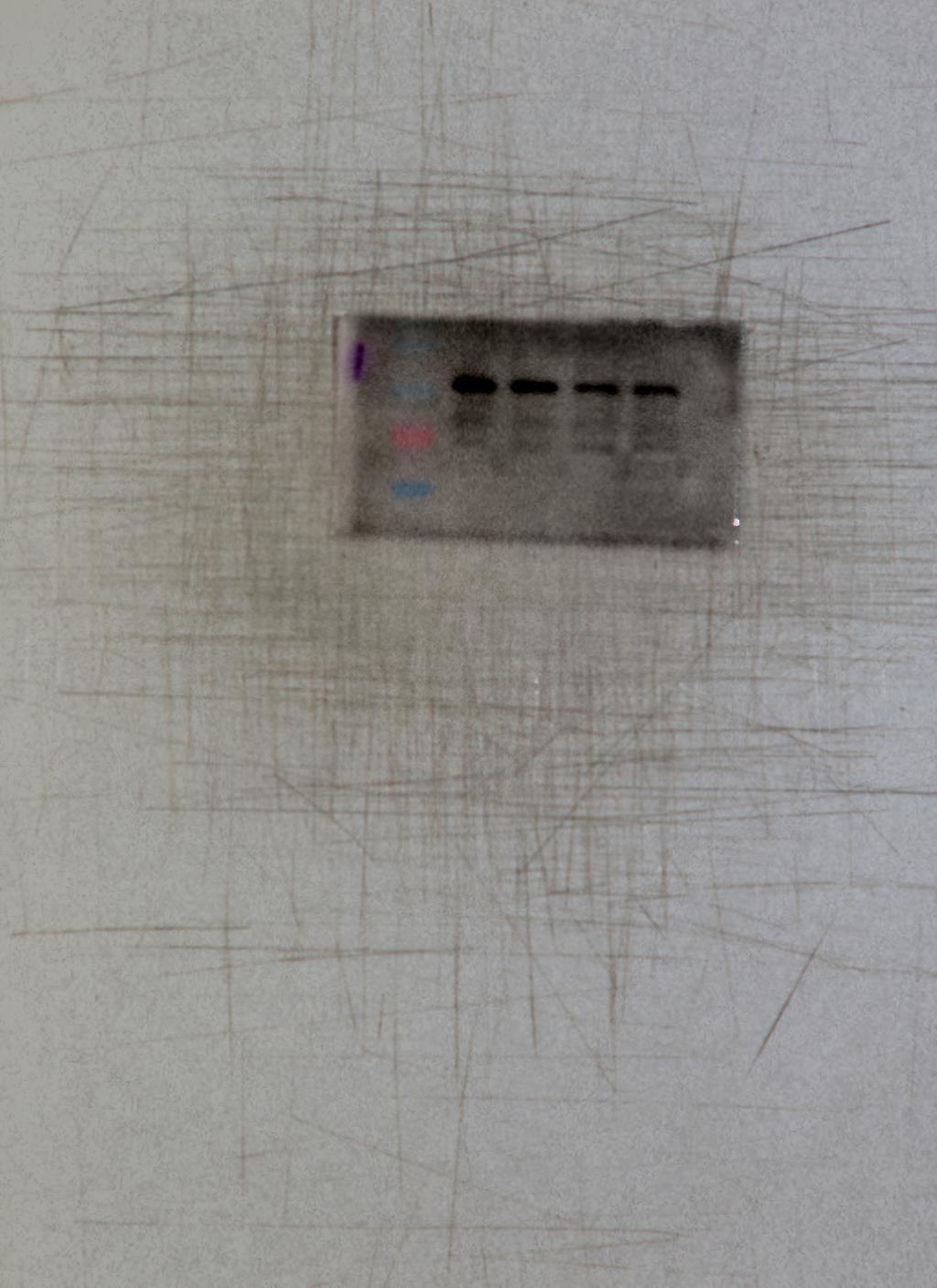

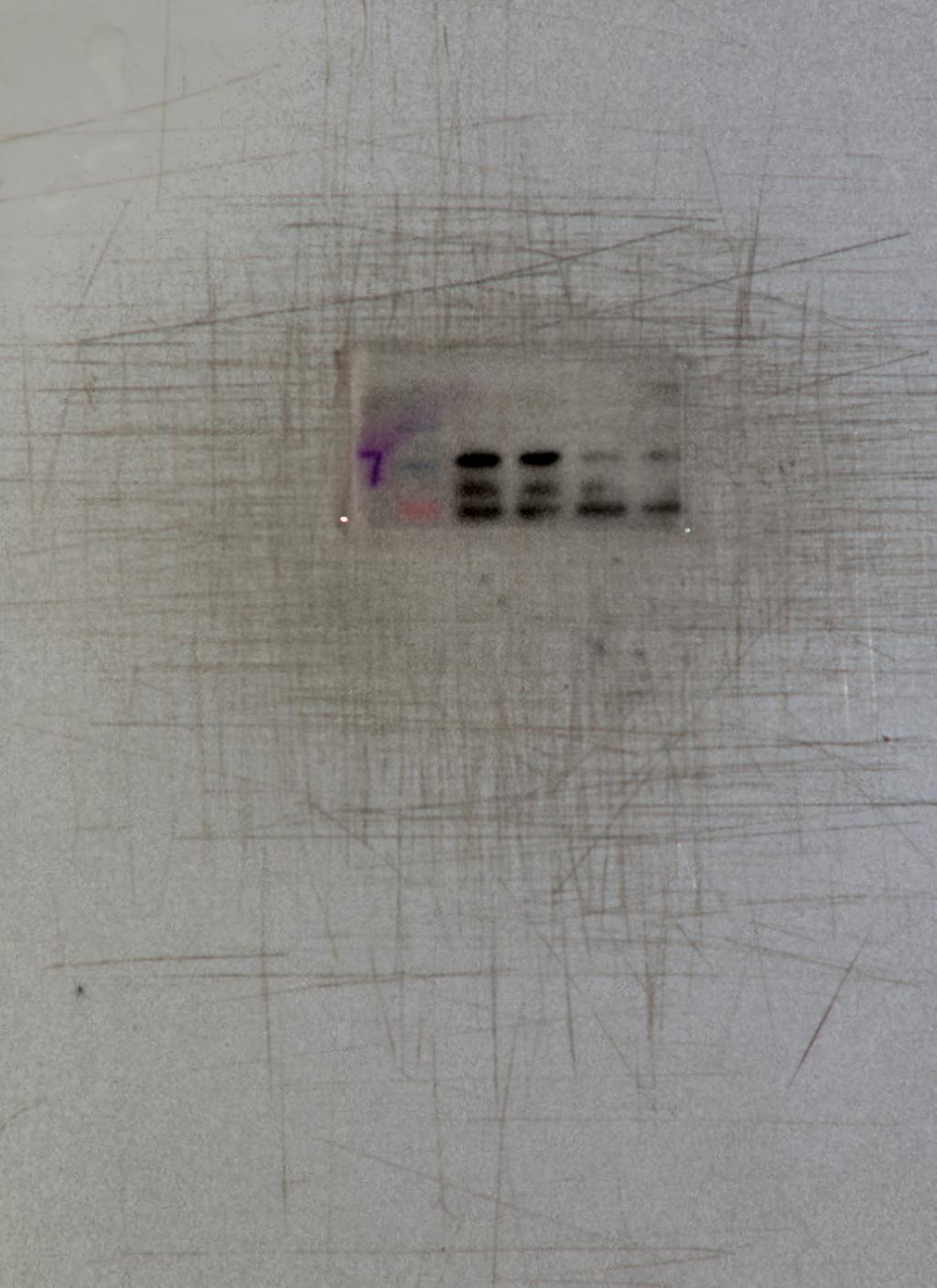


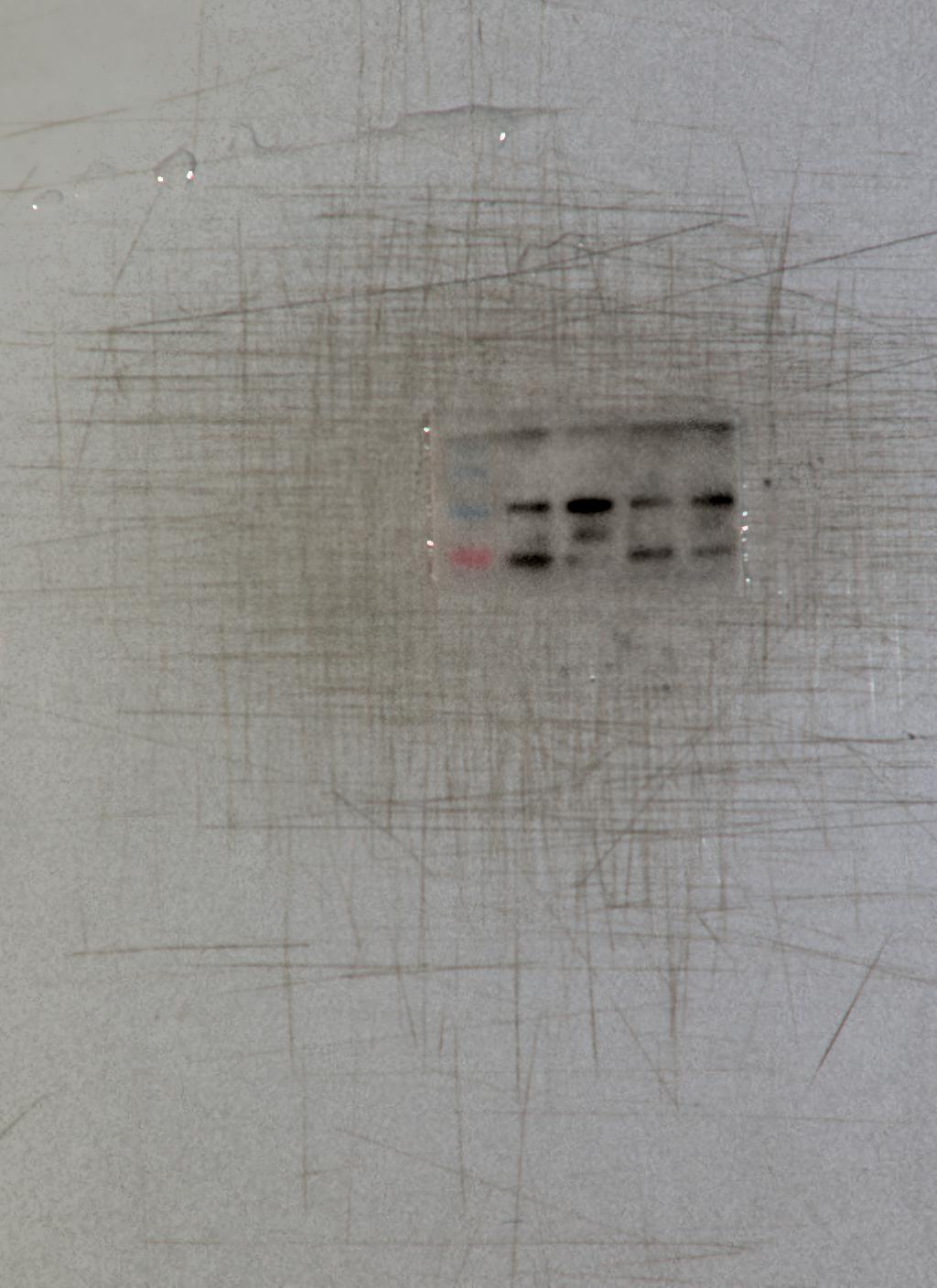


ANXA2 INPUT


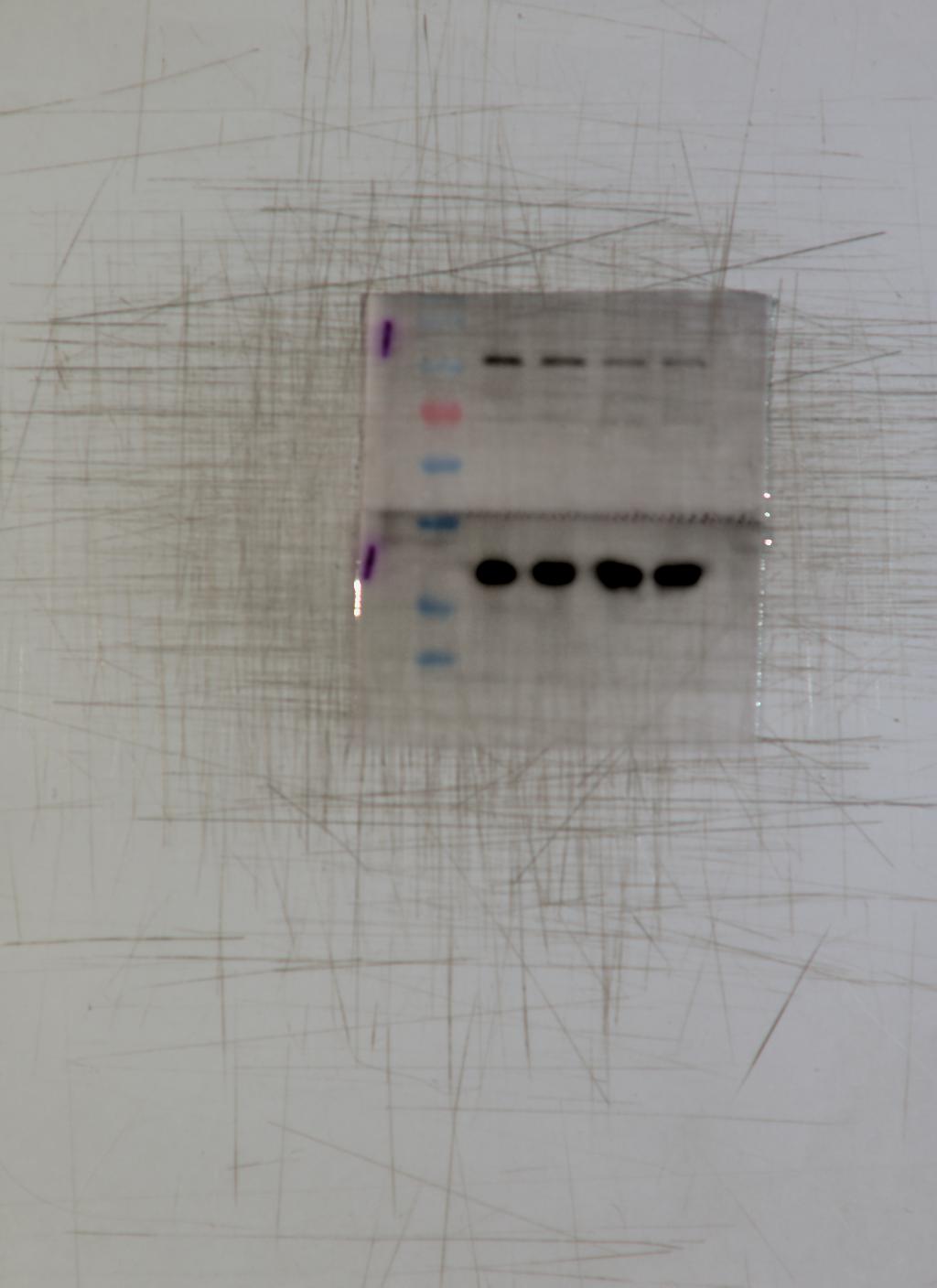


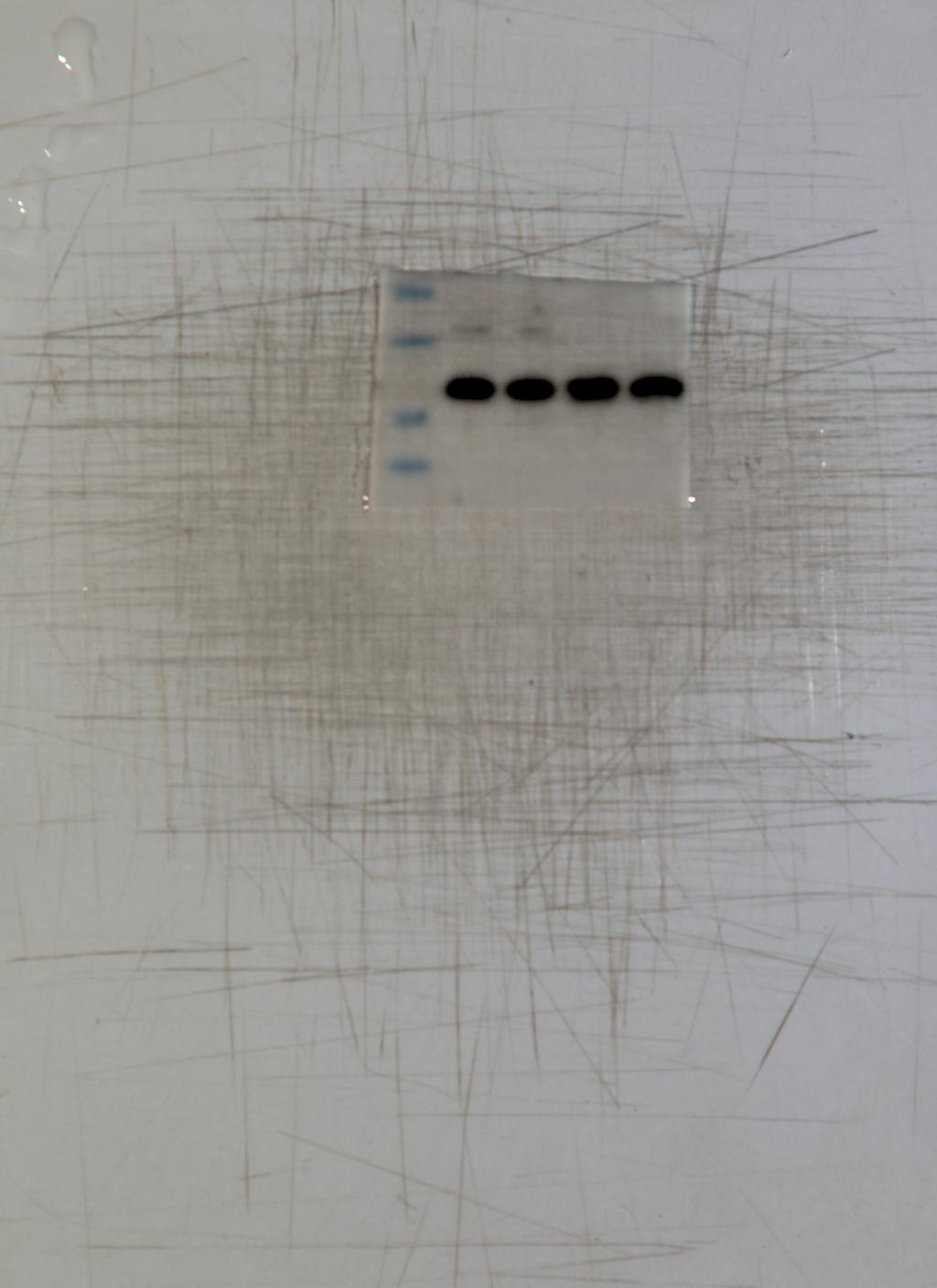


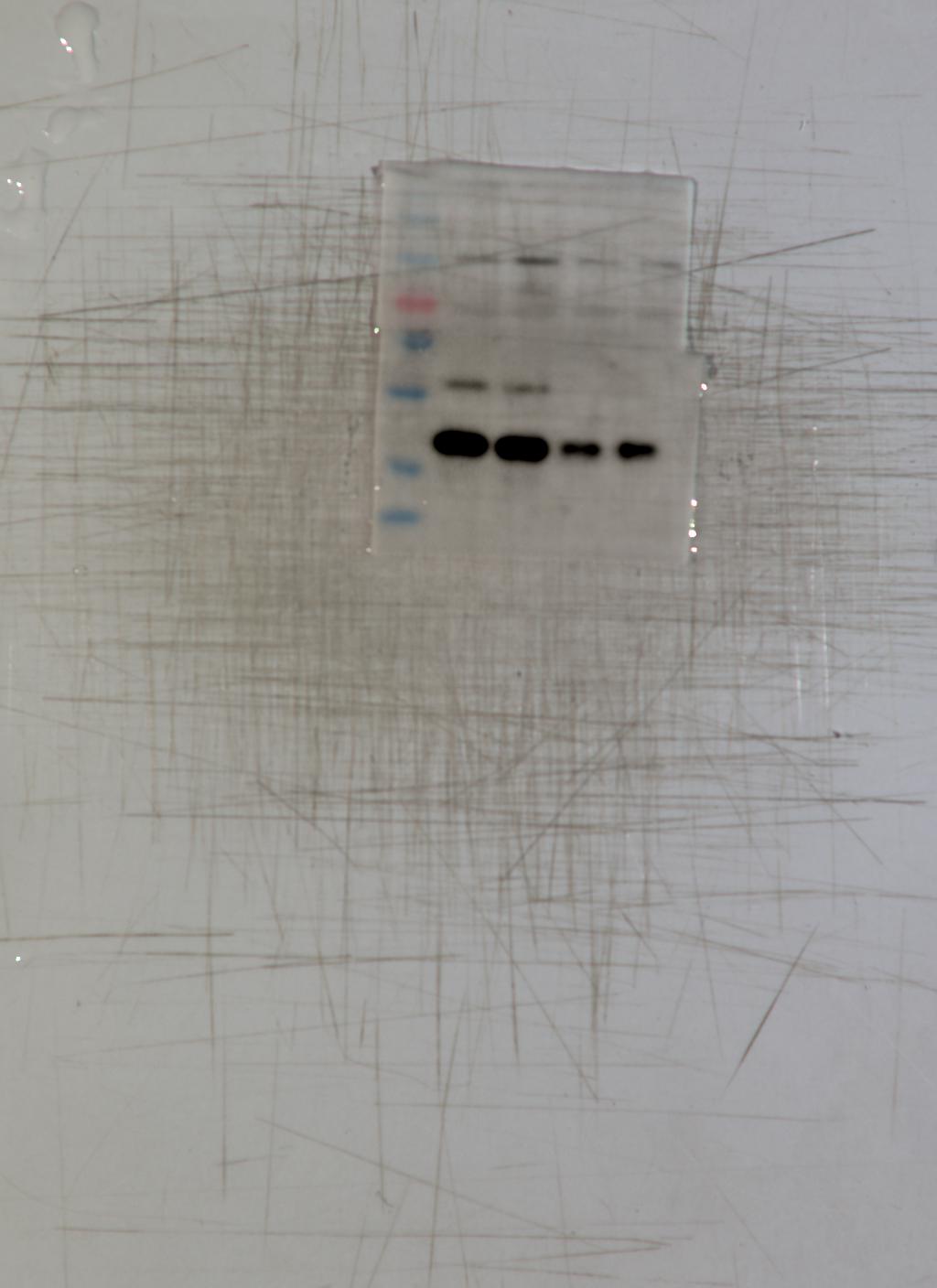


ANXA2 IP


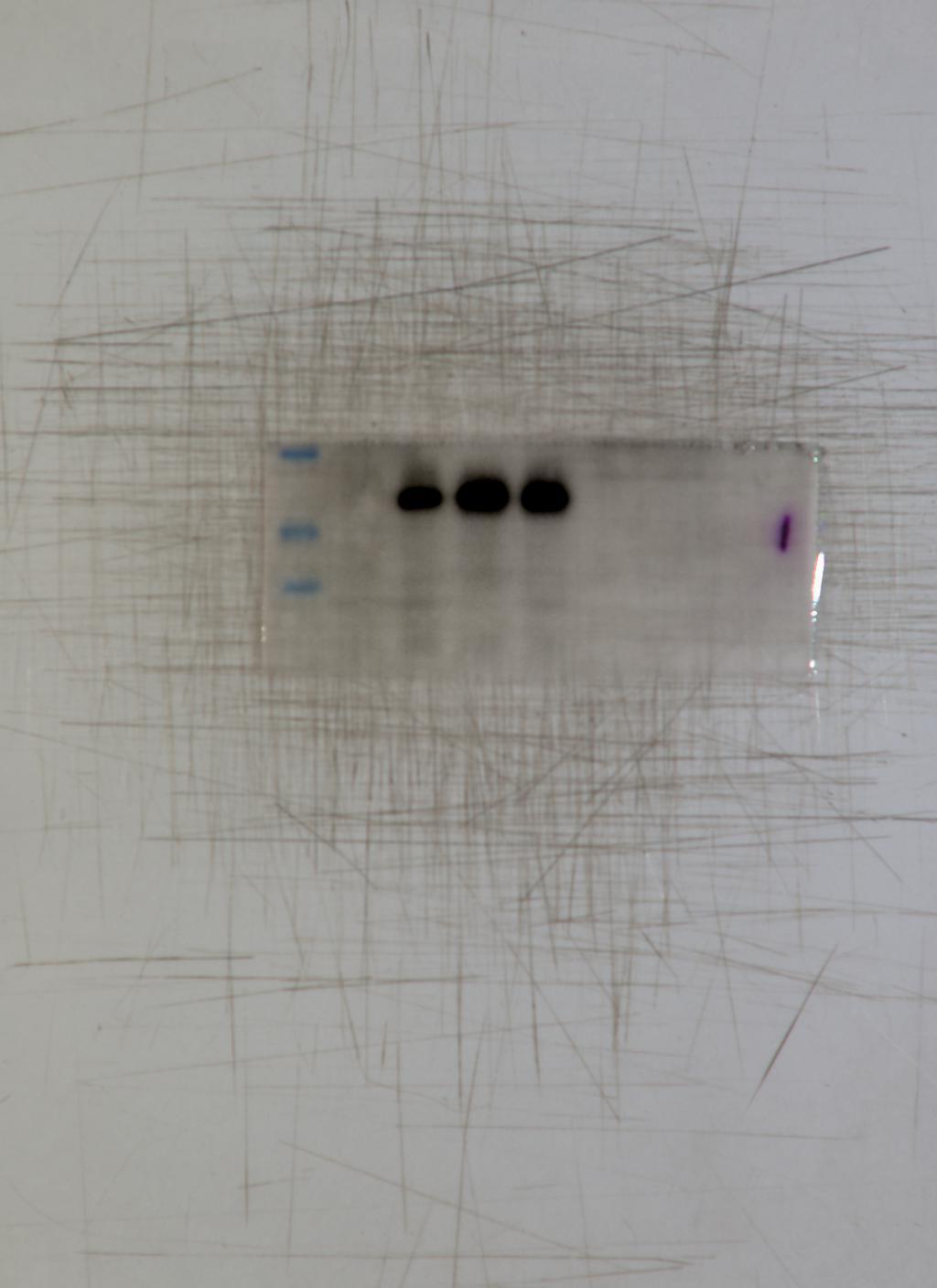


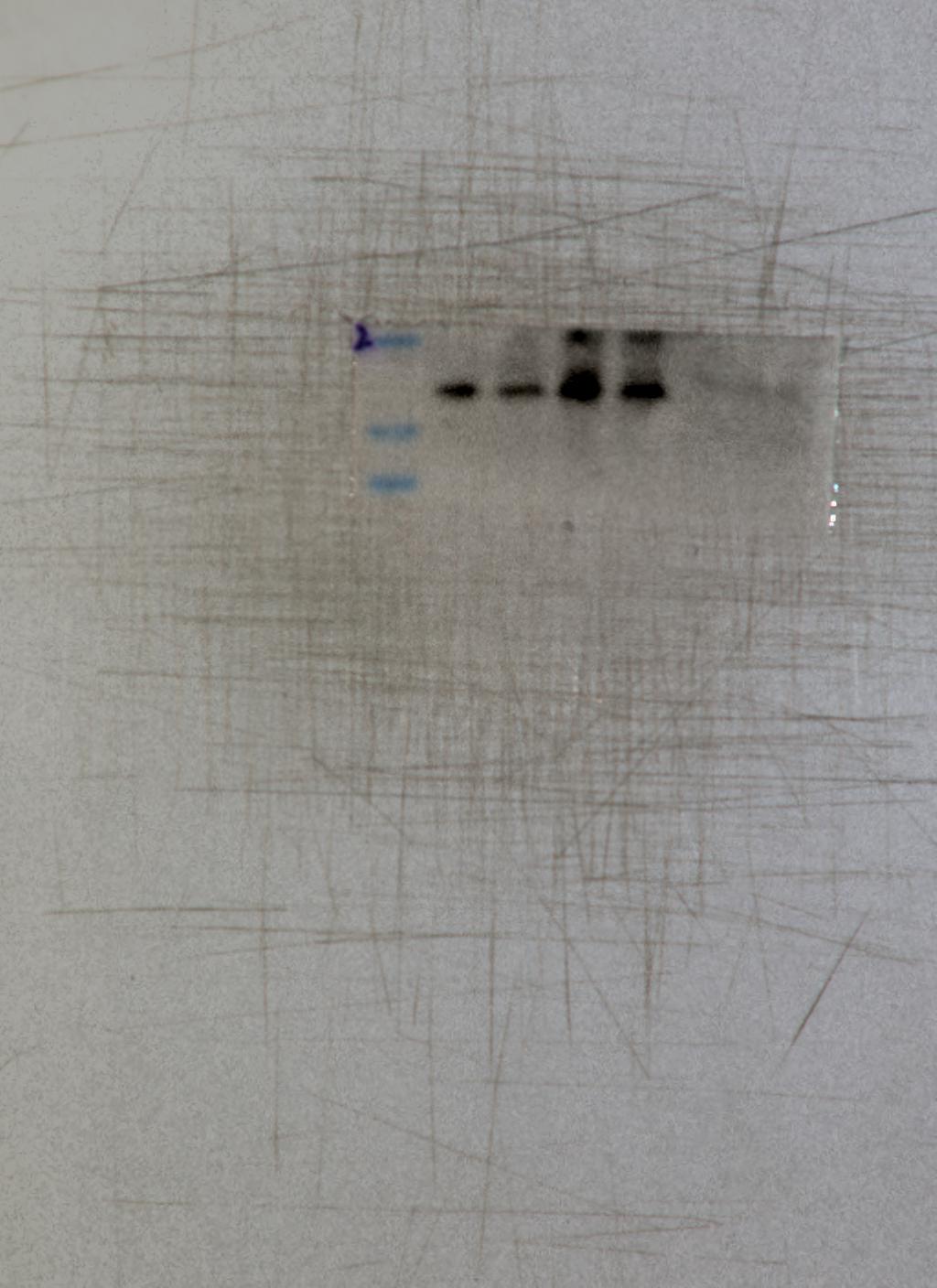

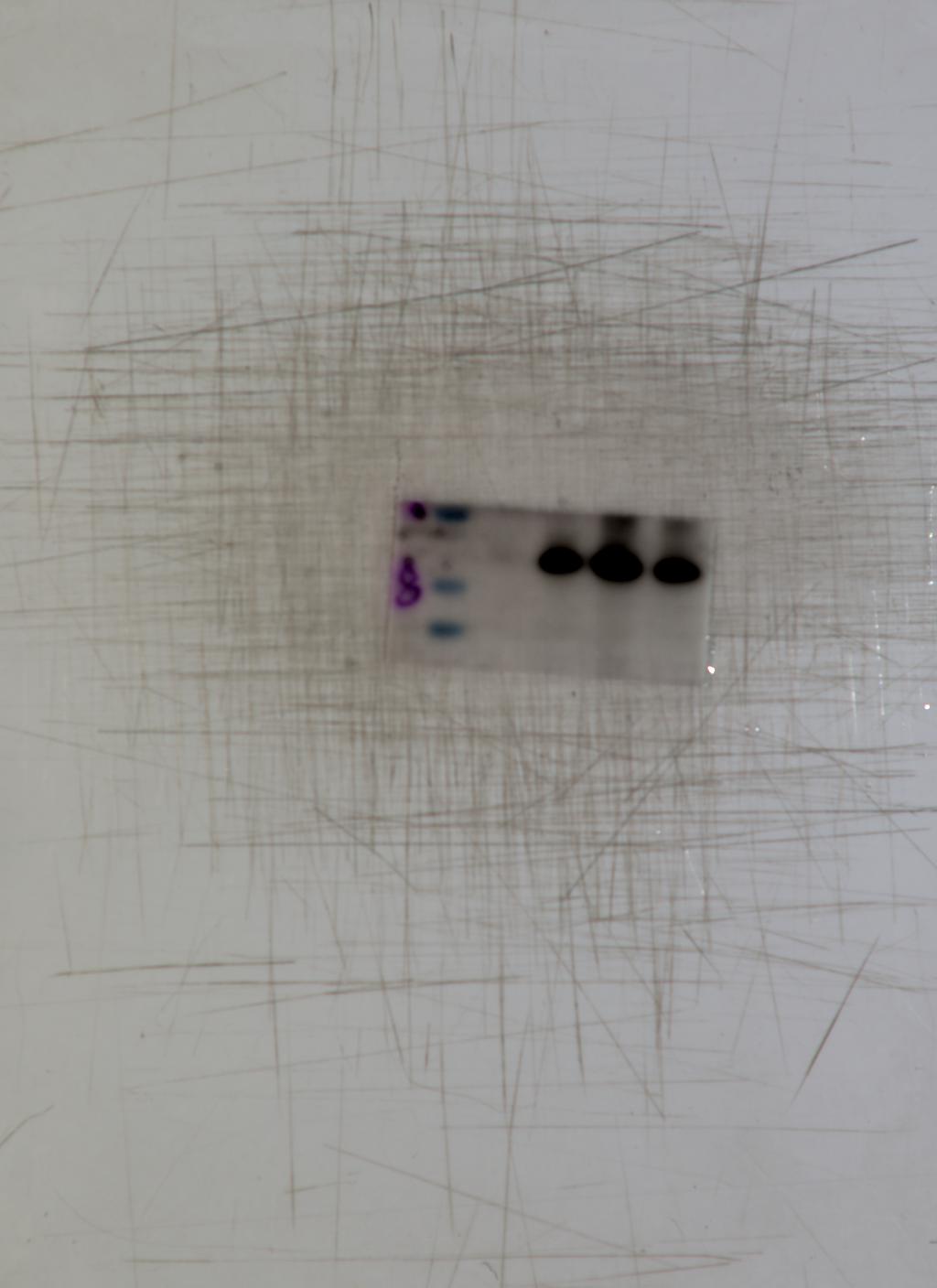


TLR4 IP


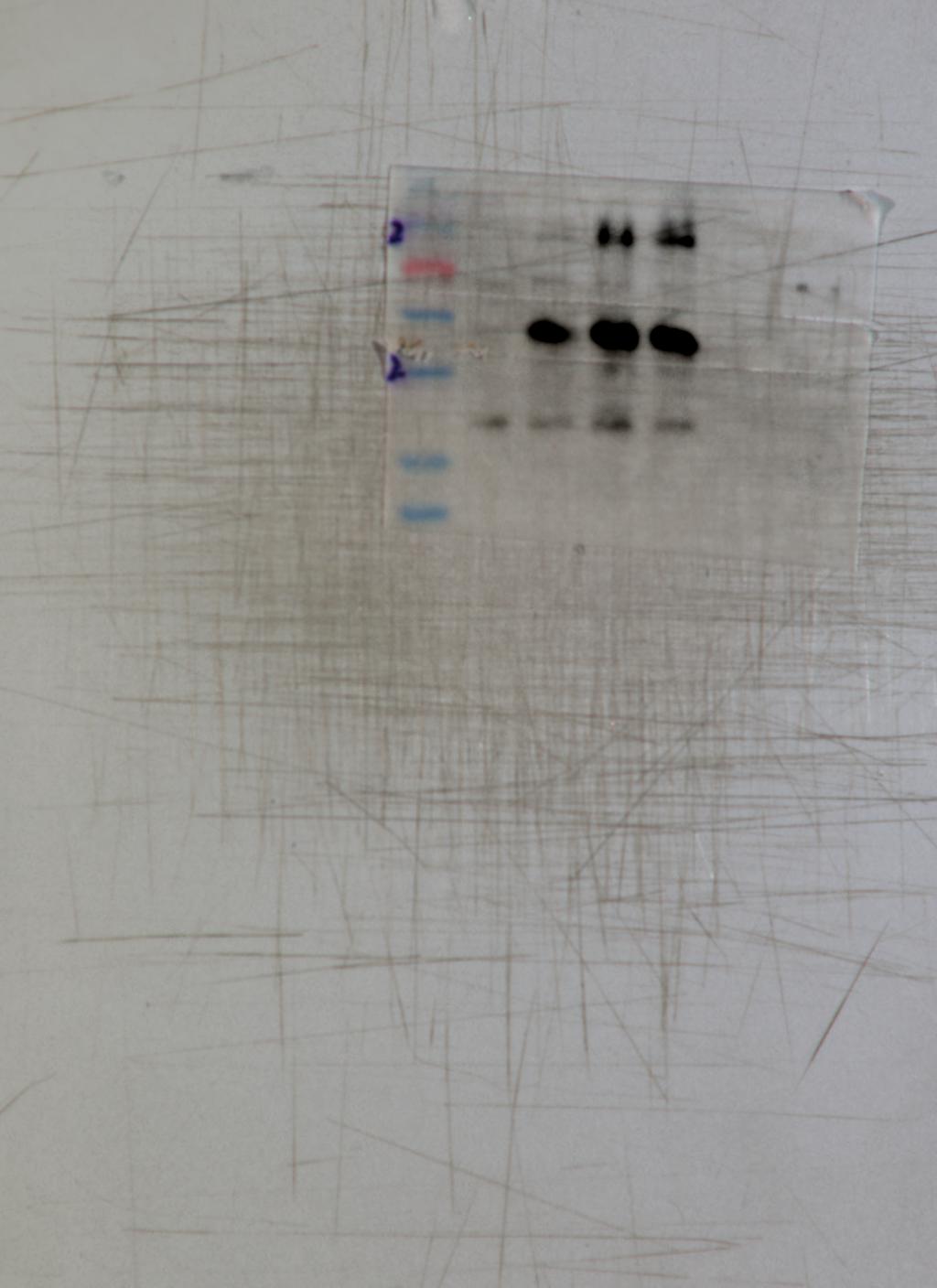


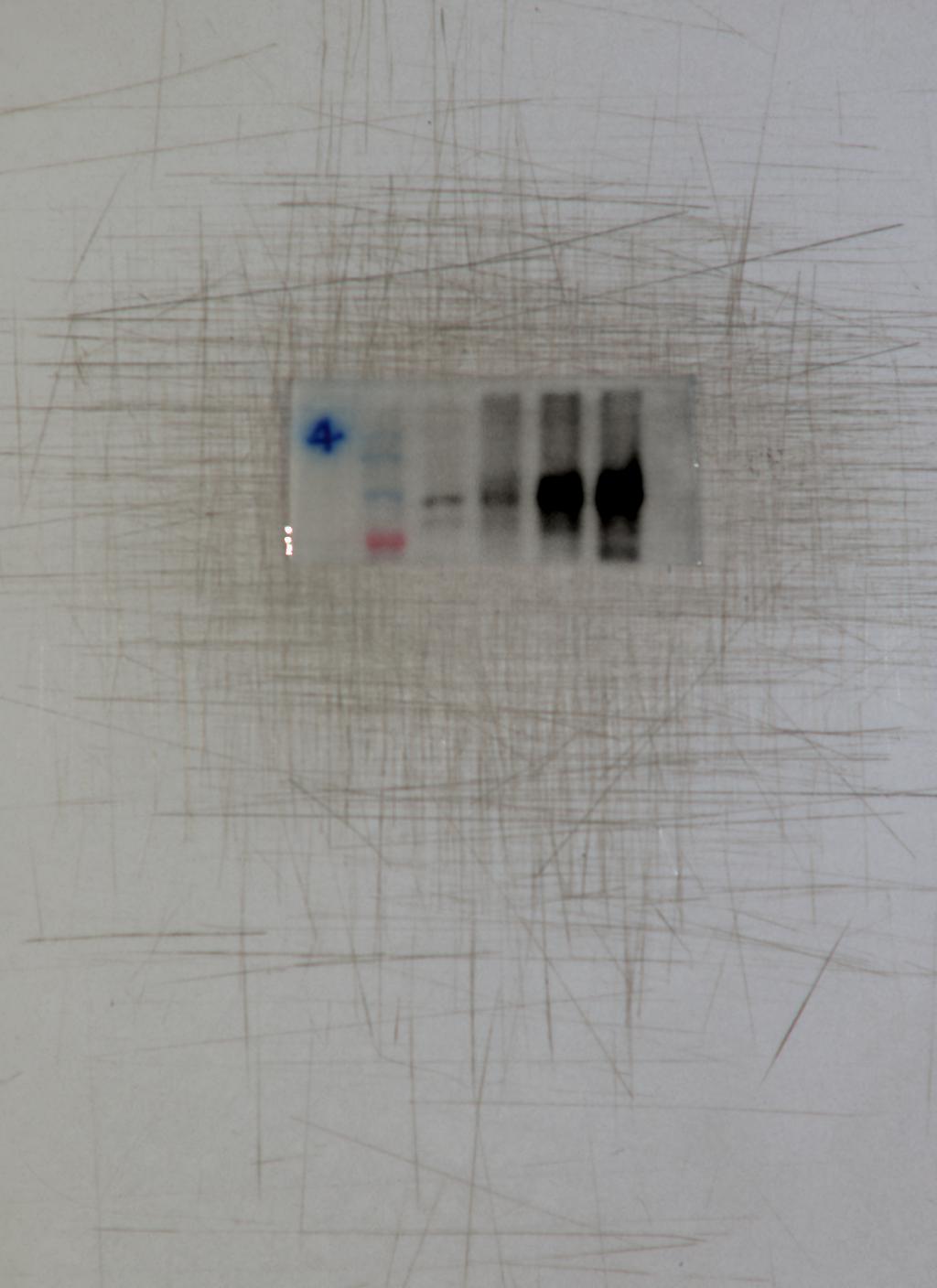


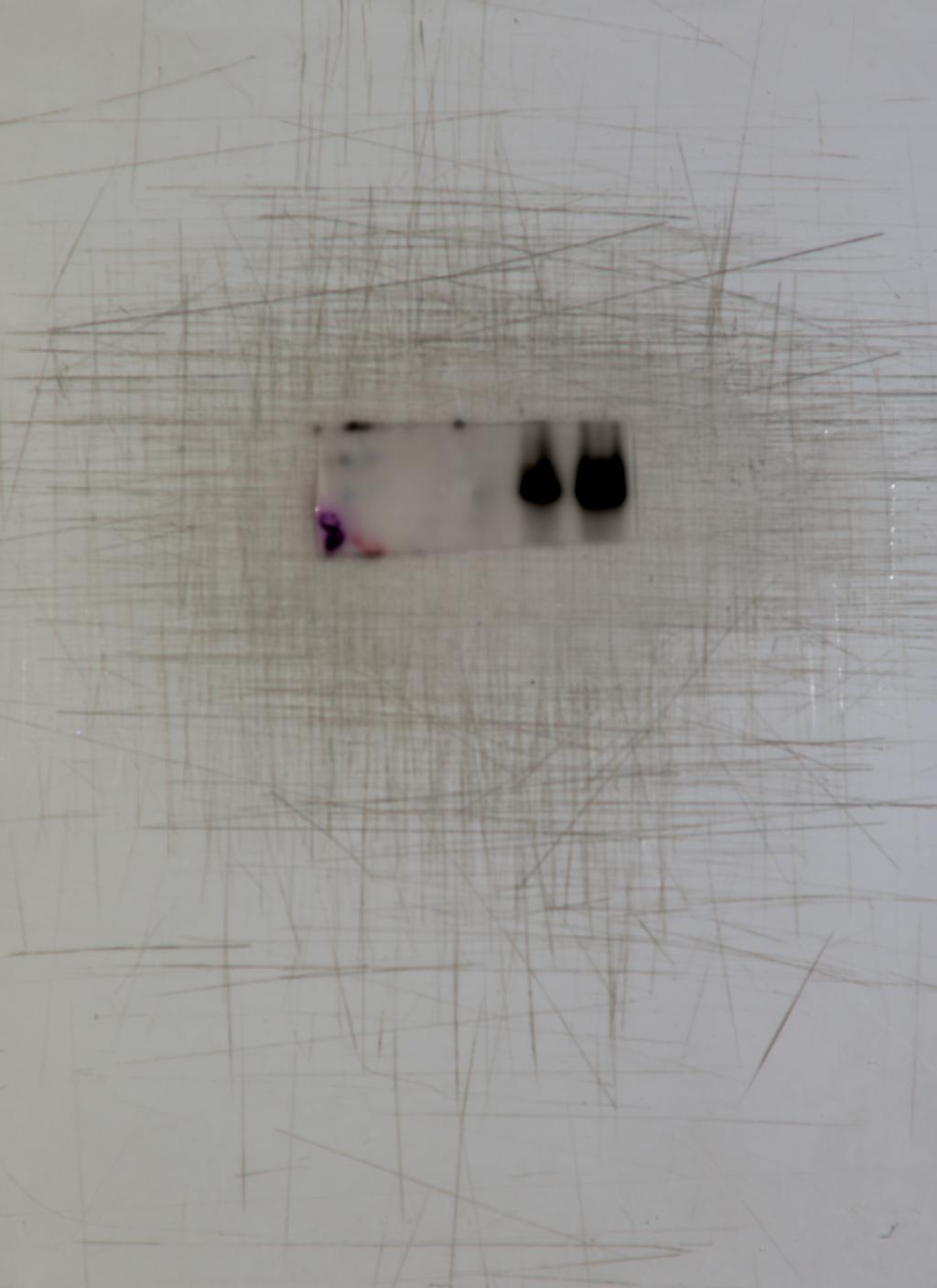


Figure 5F

Beta-ACTIN


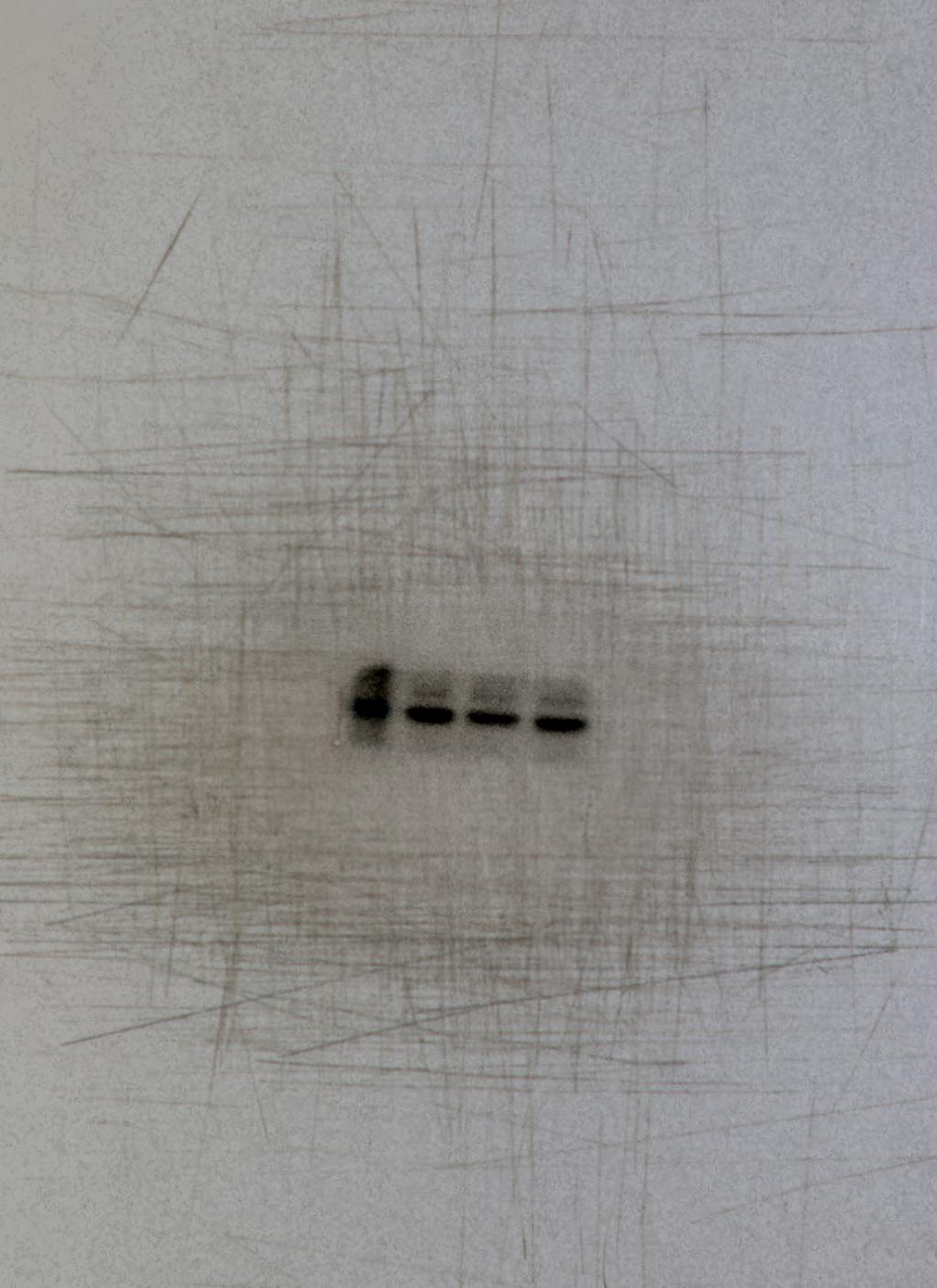

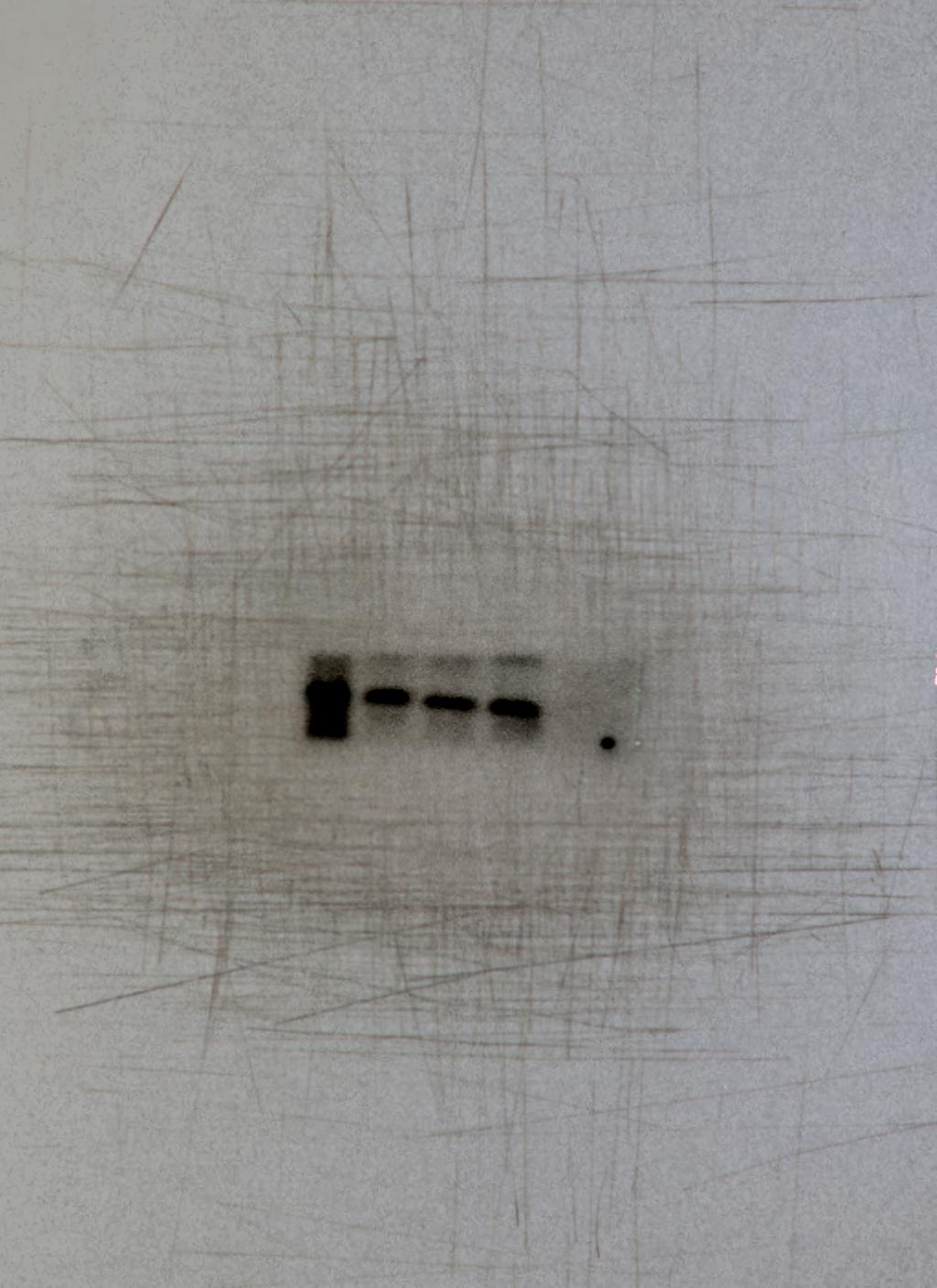

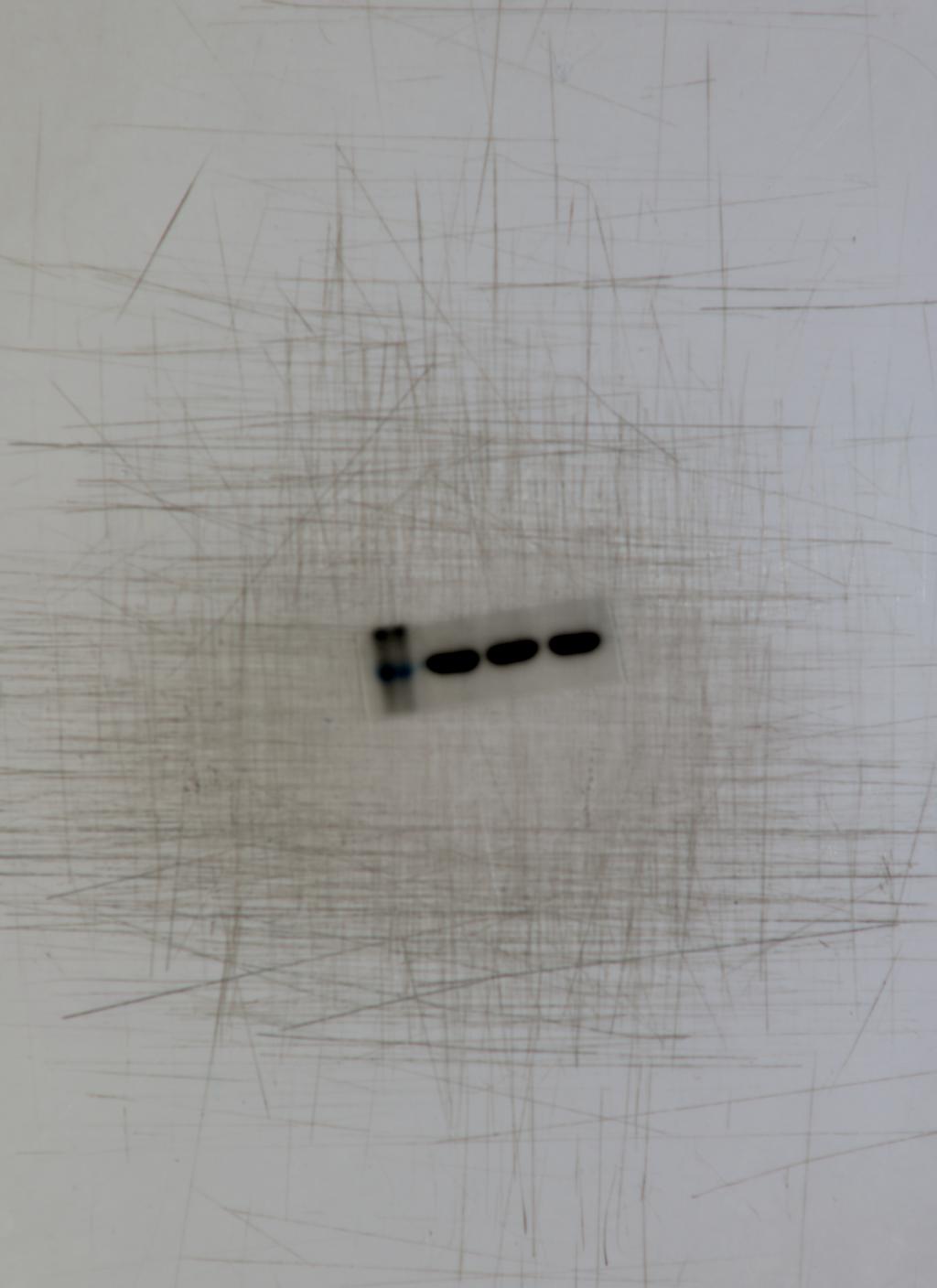


IGFBP5


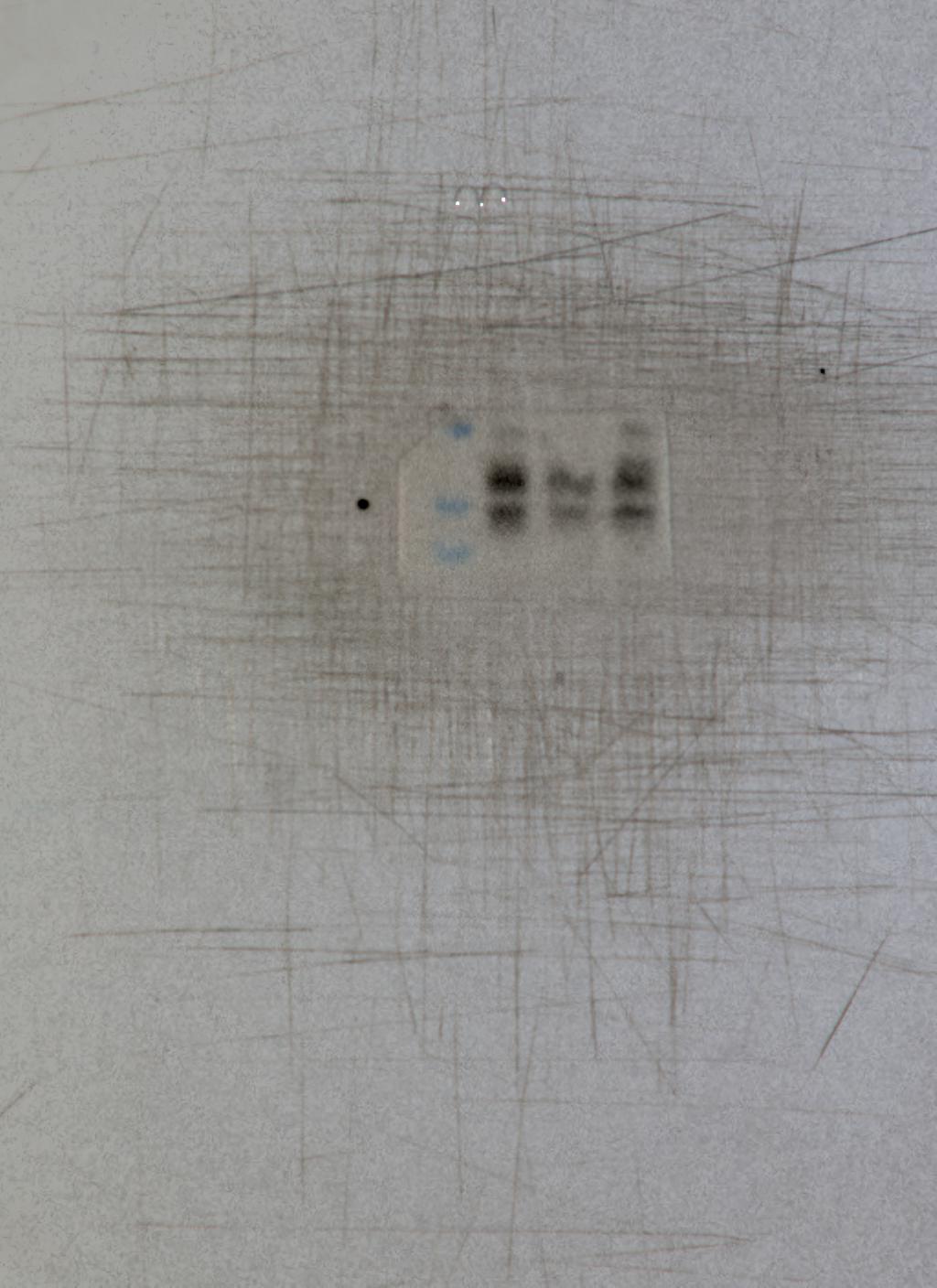


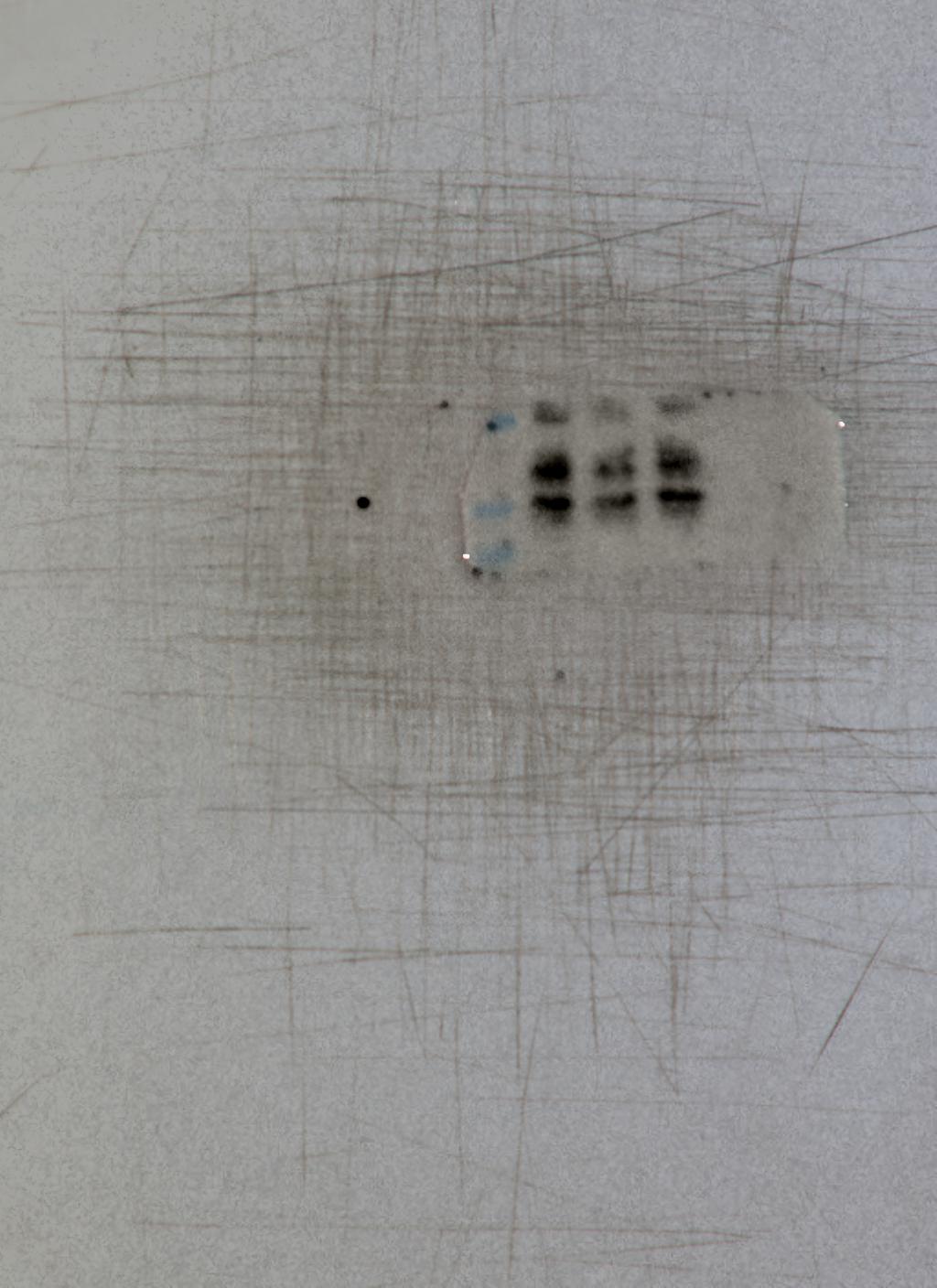


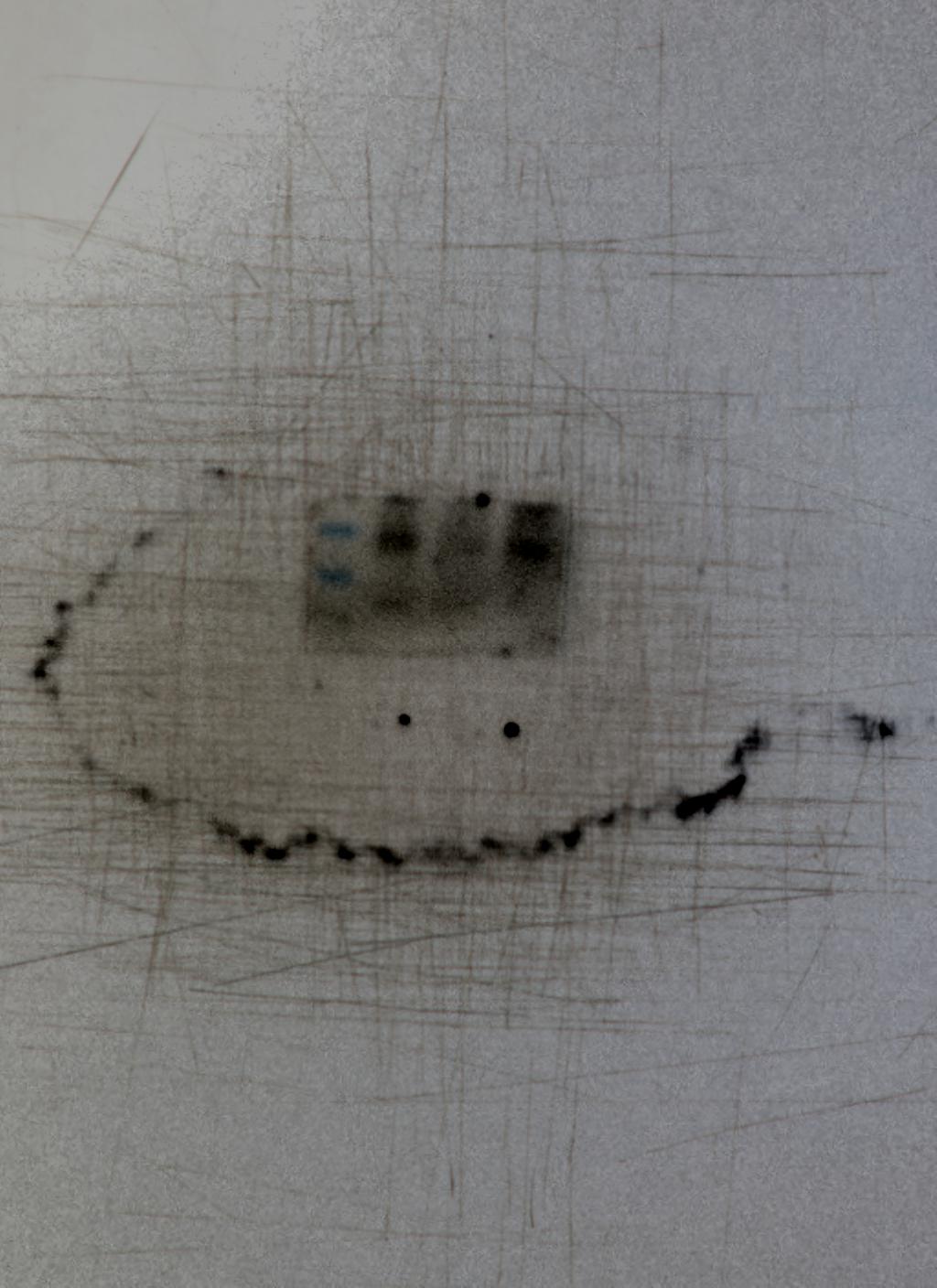

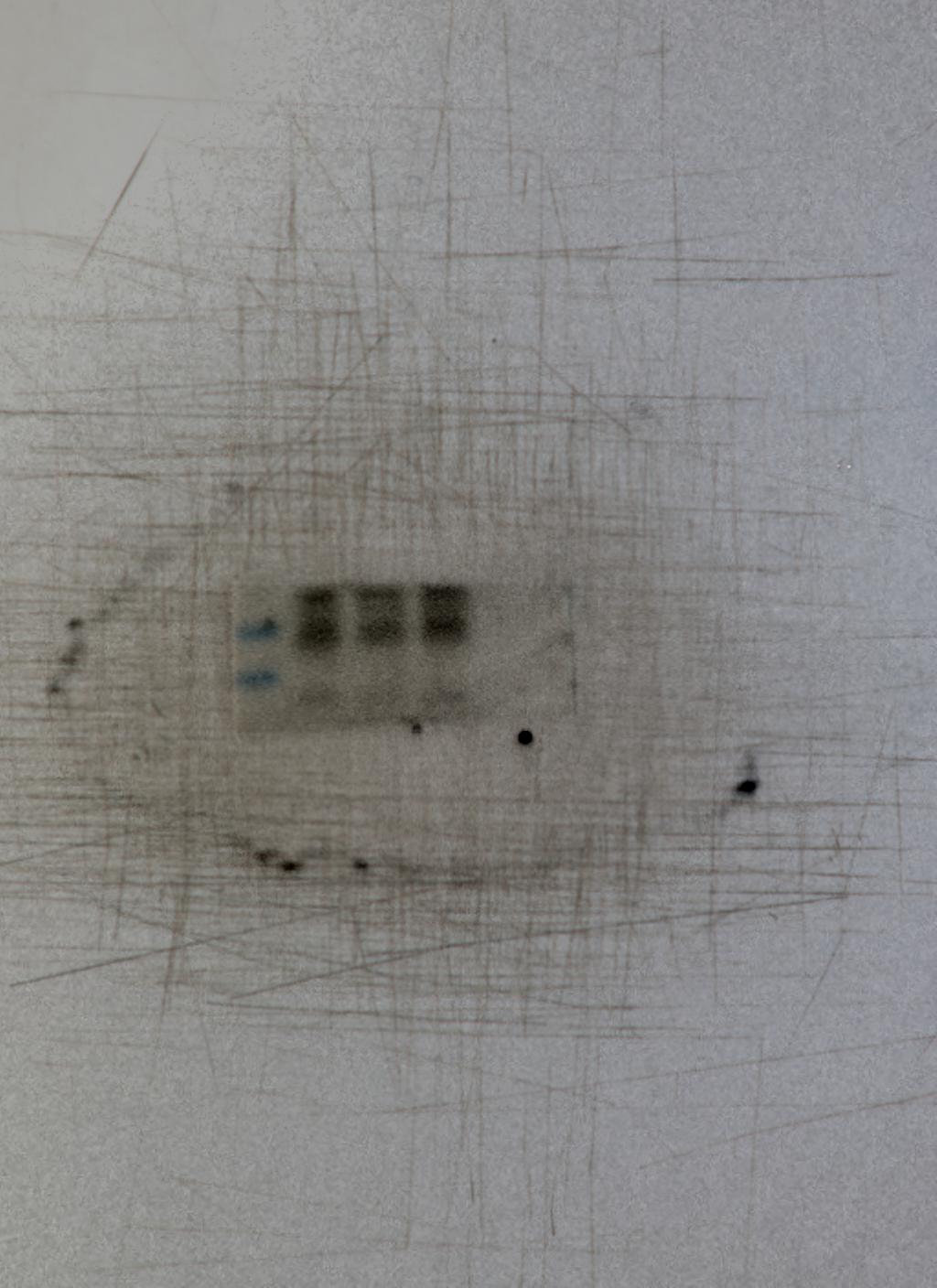

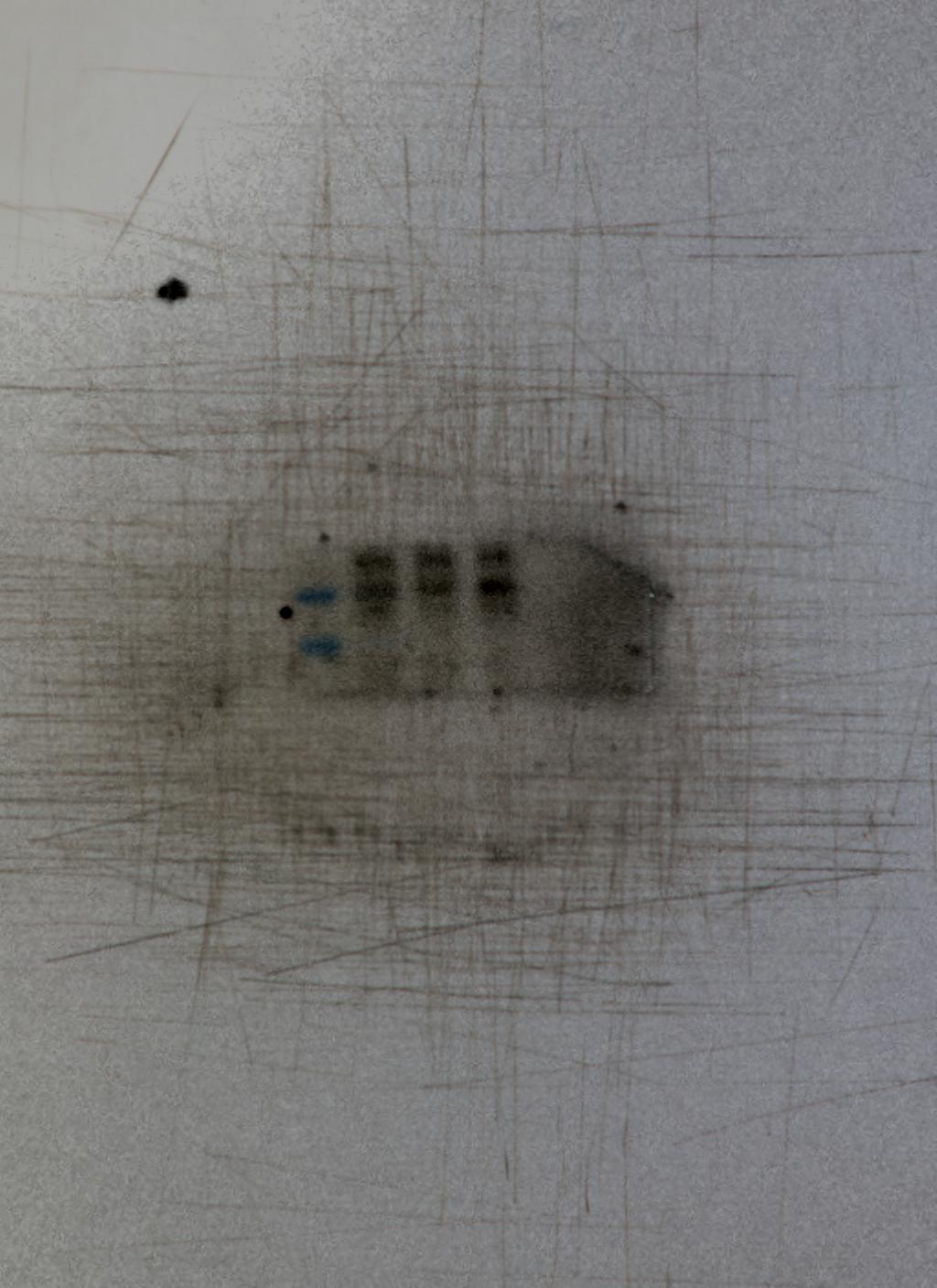


ANXA2


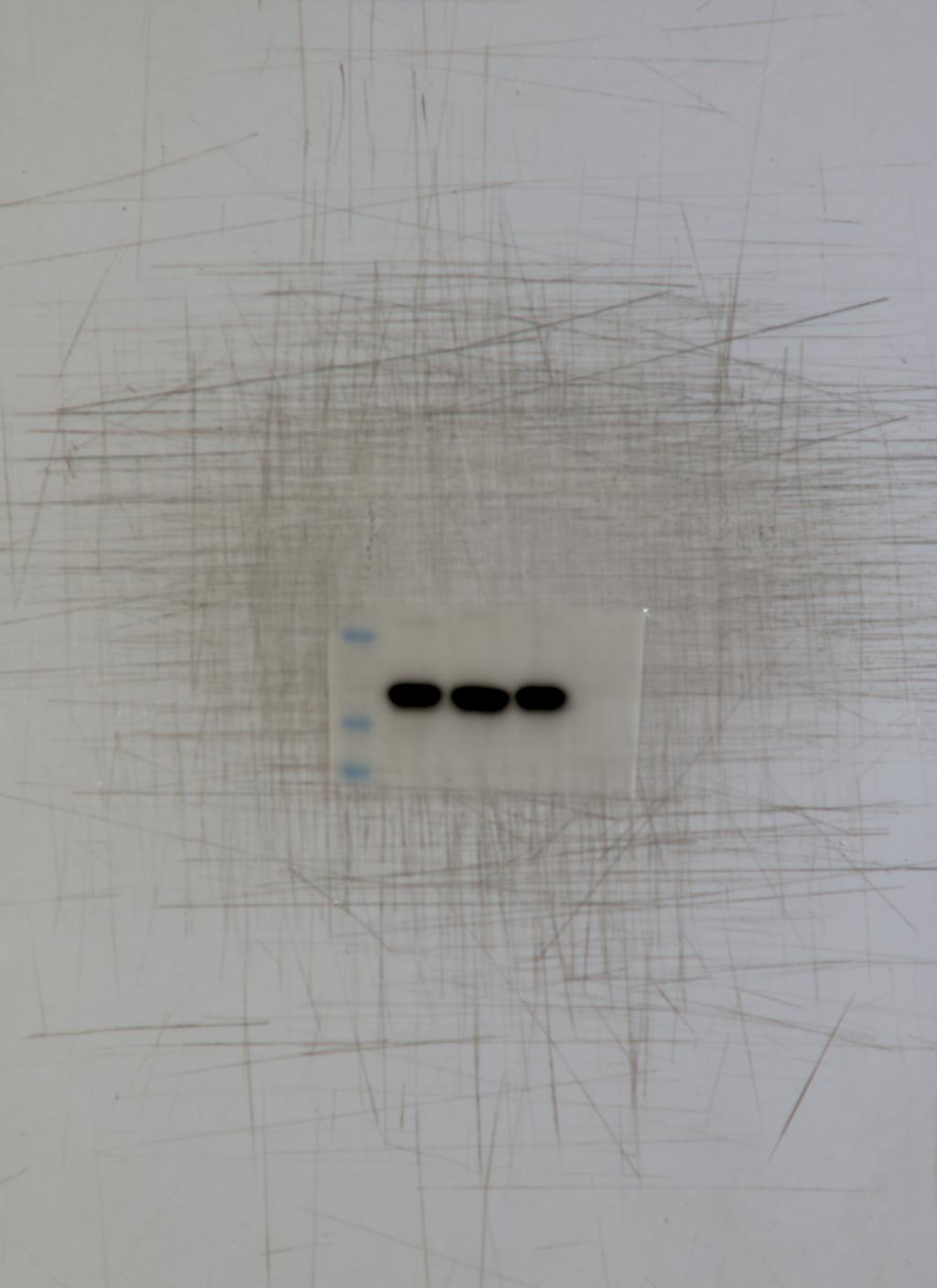

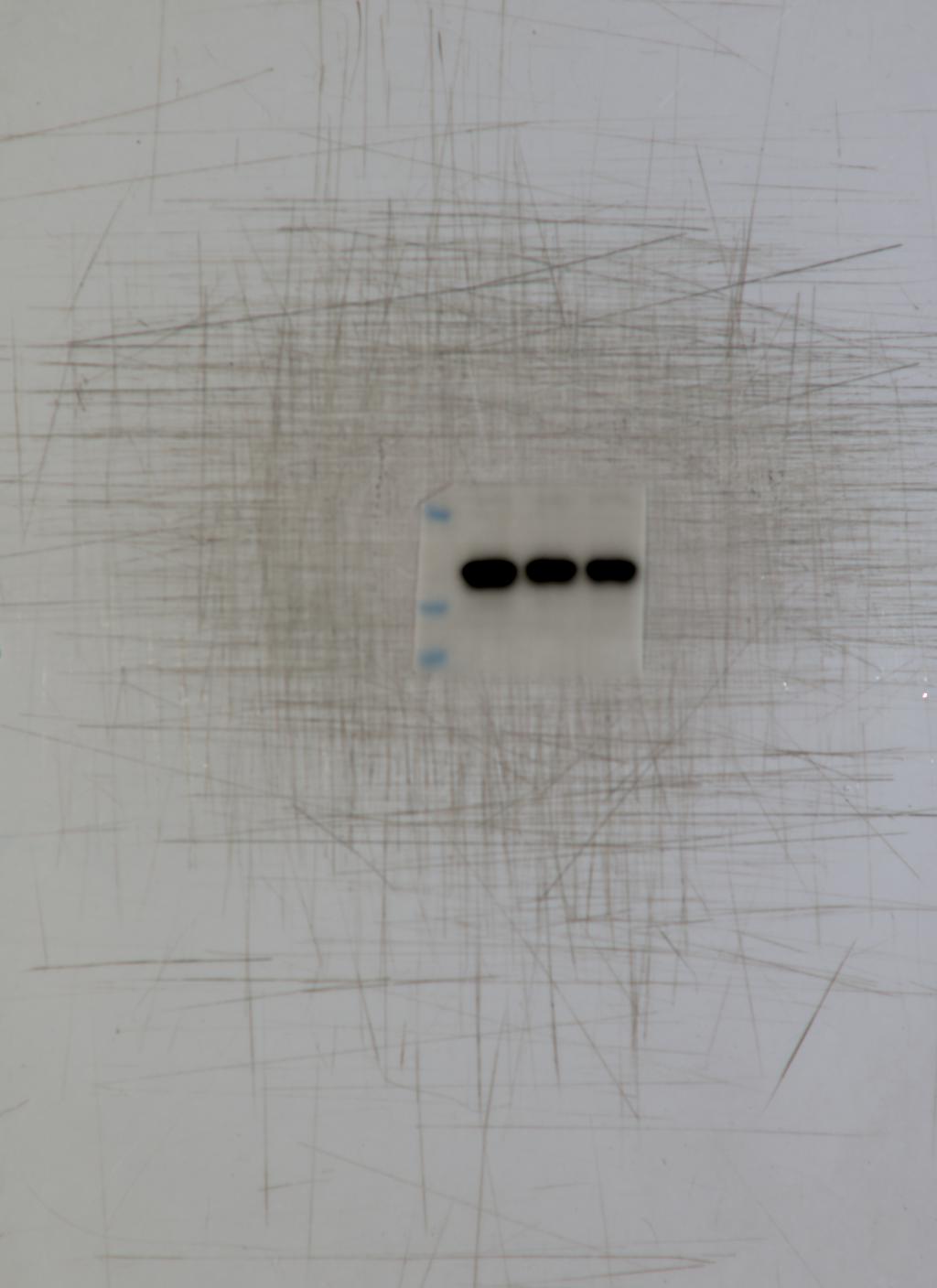

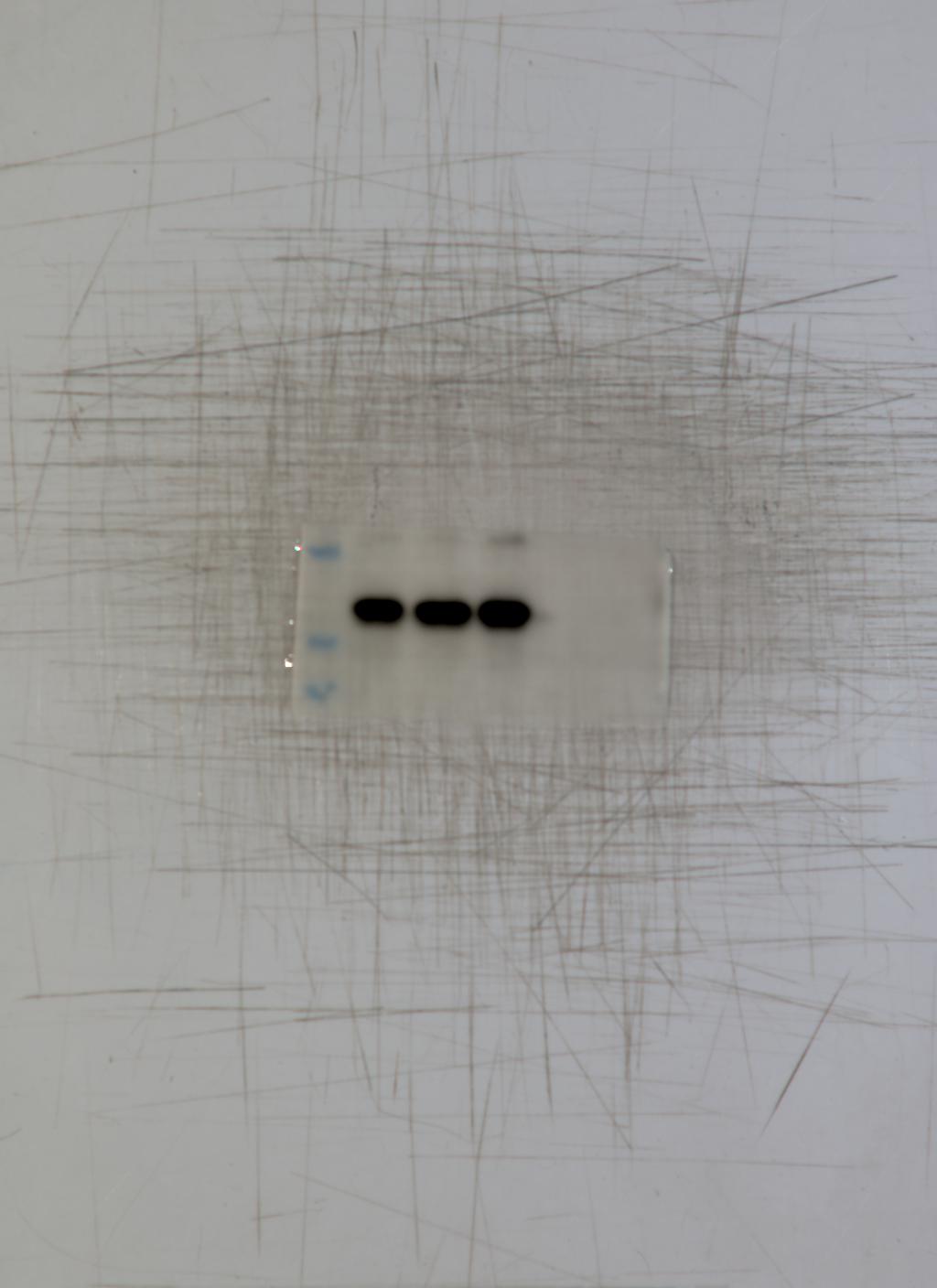


p-ANXA2


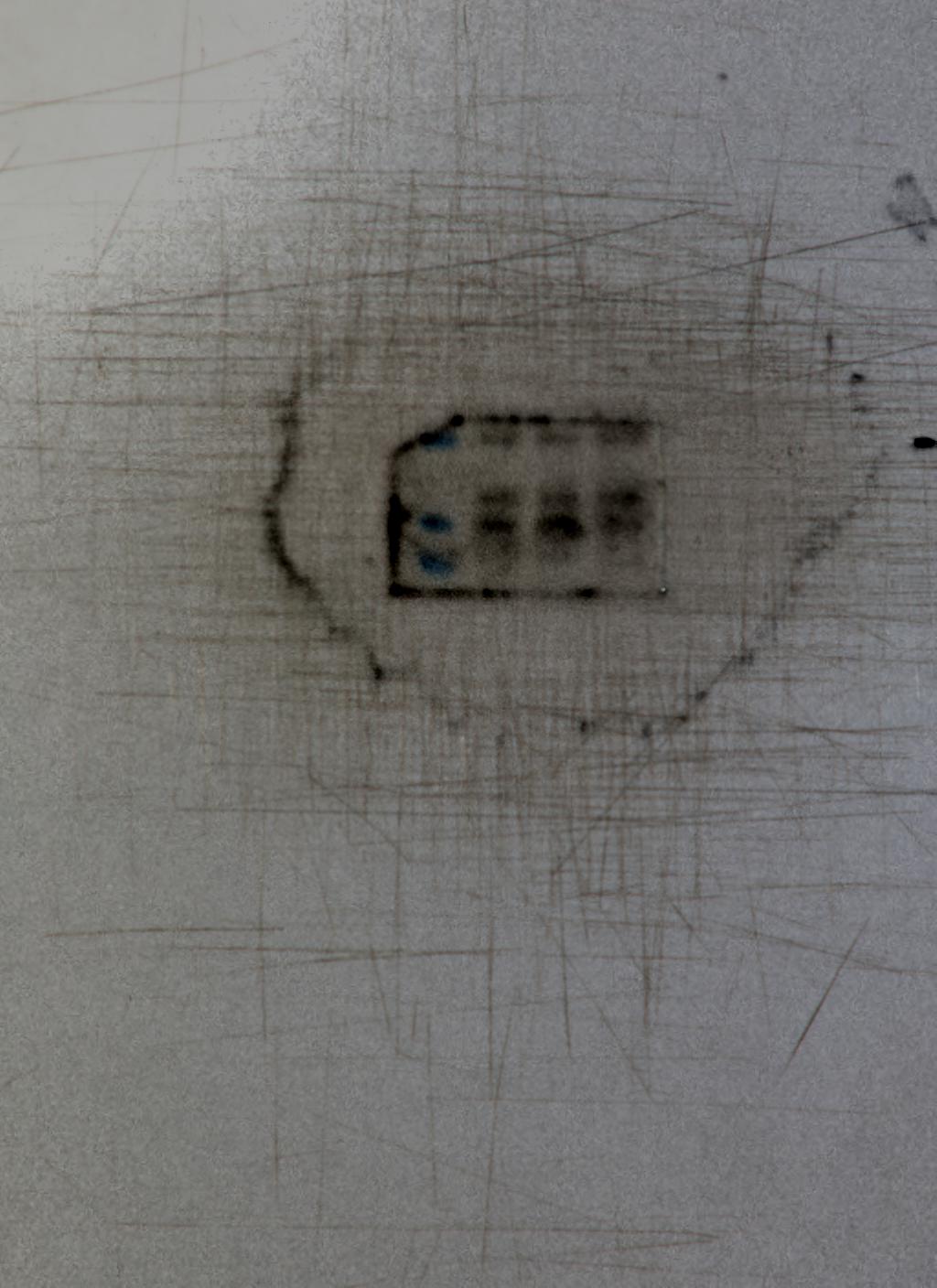


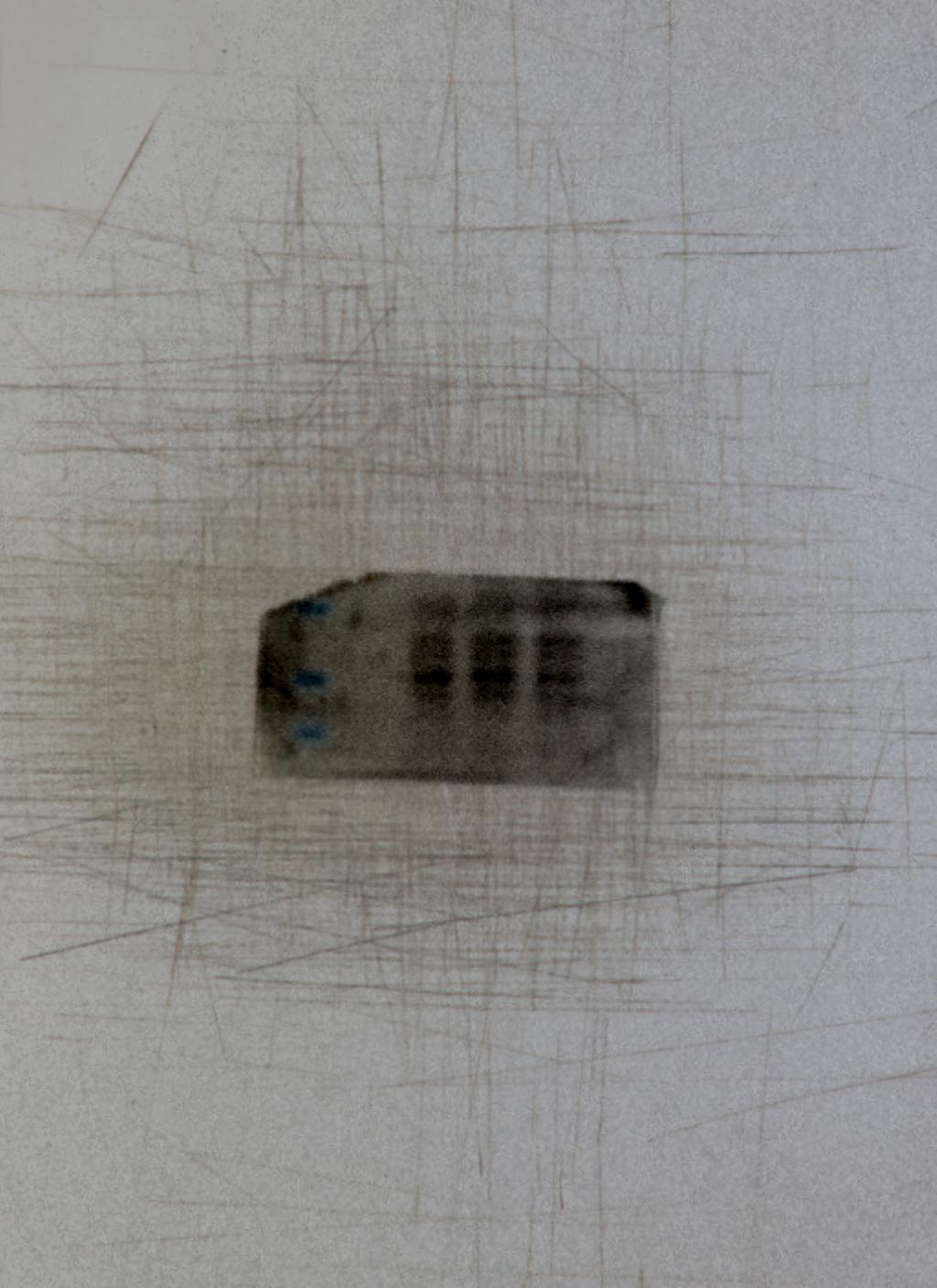


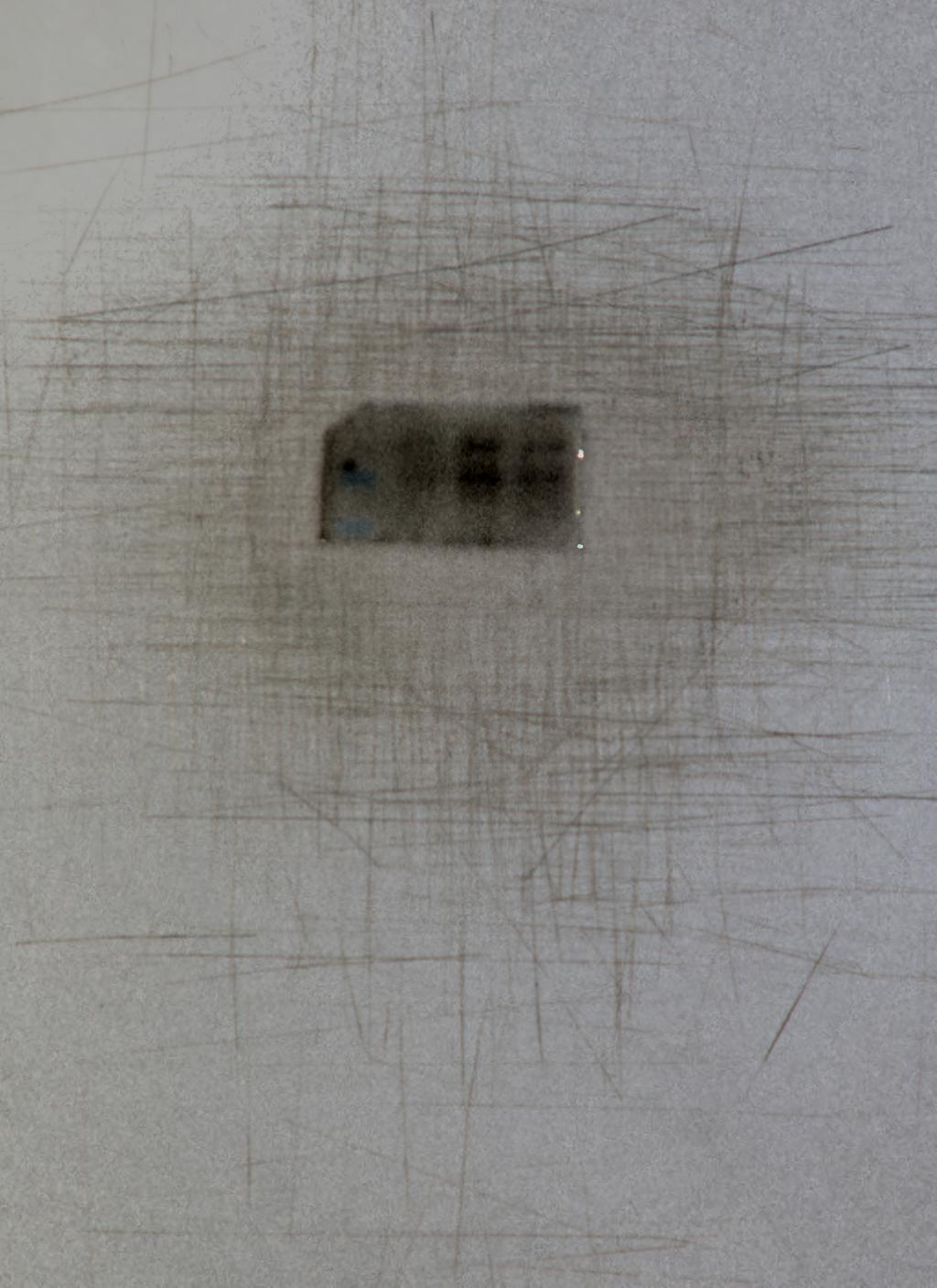


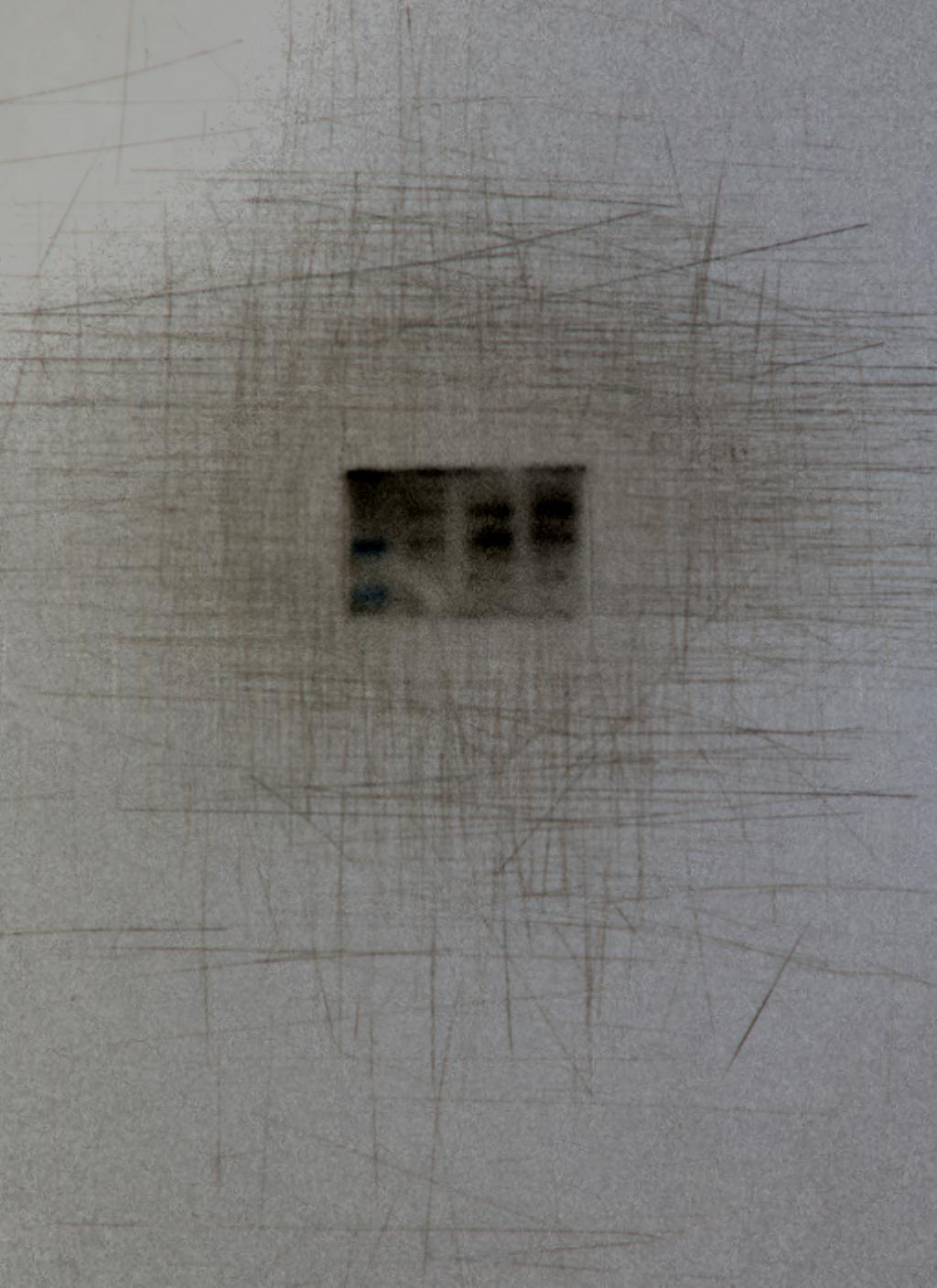


P65


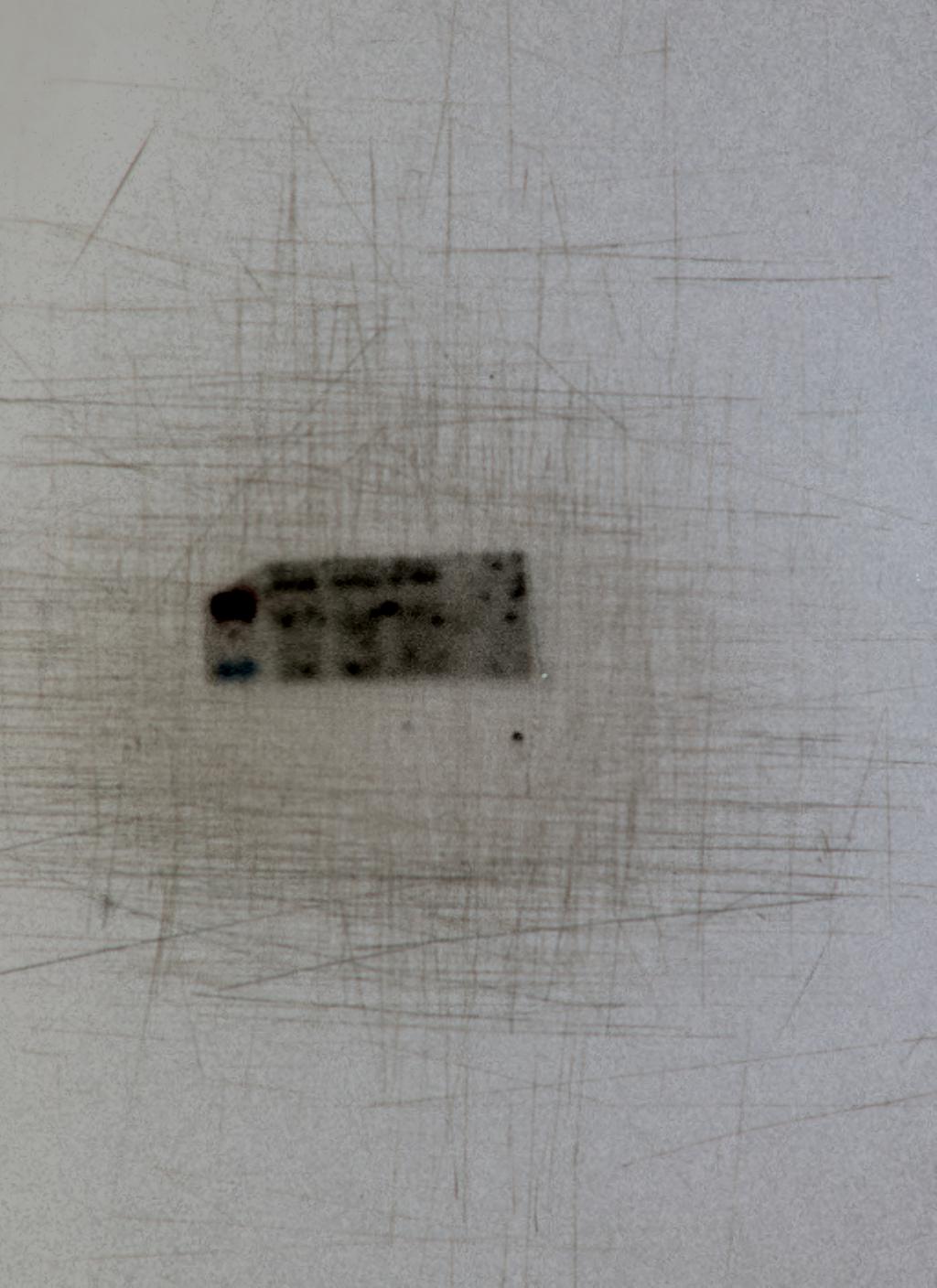


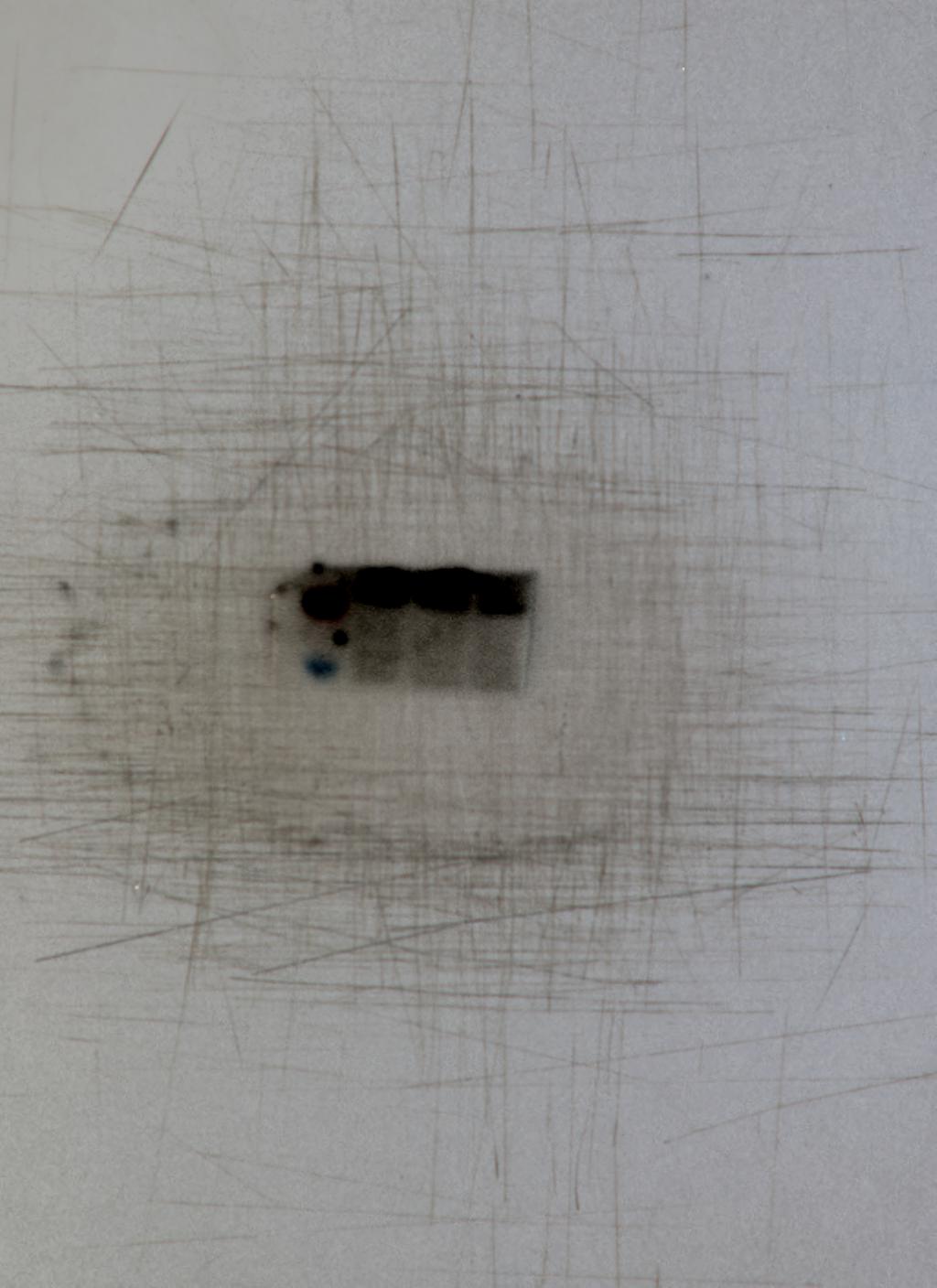


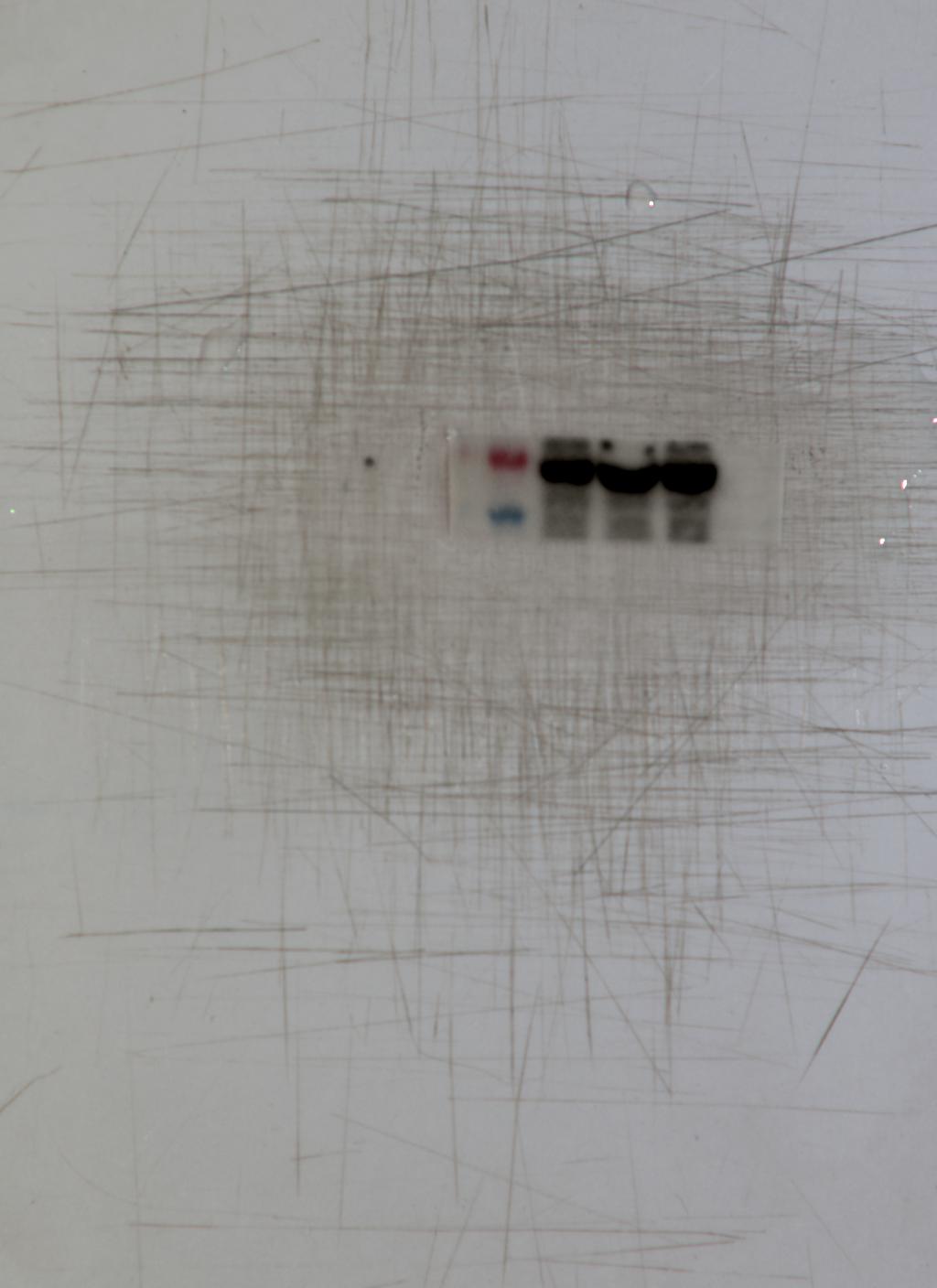


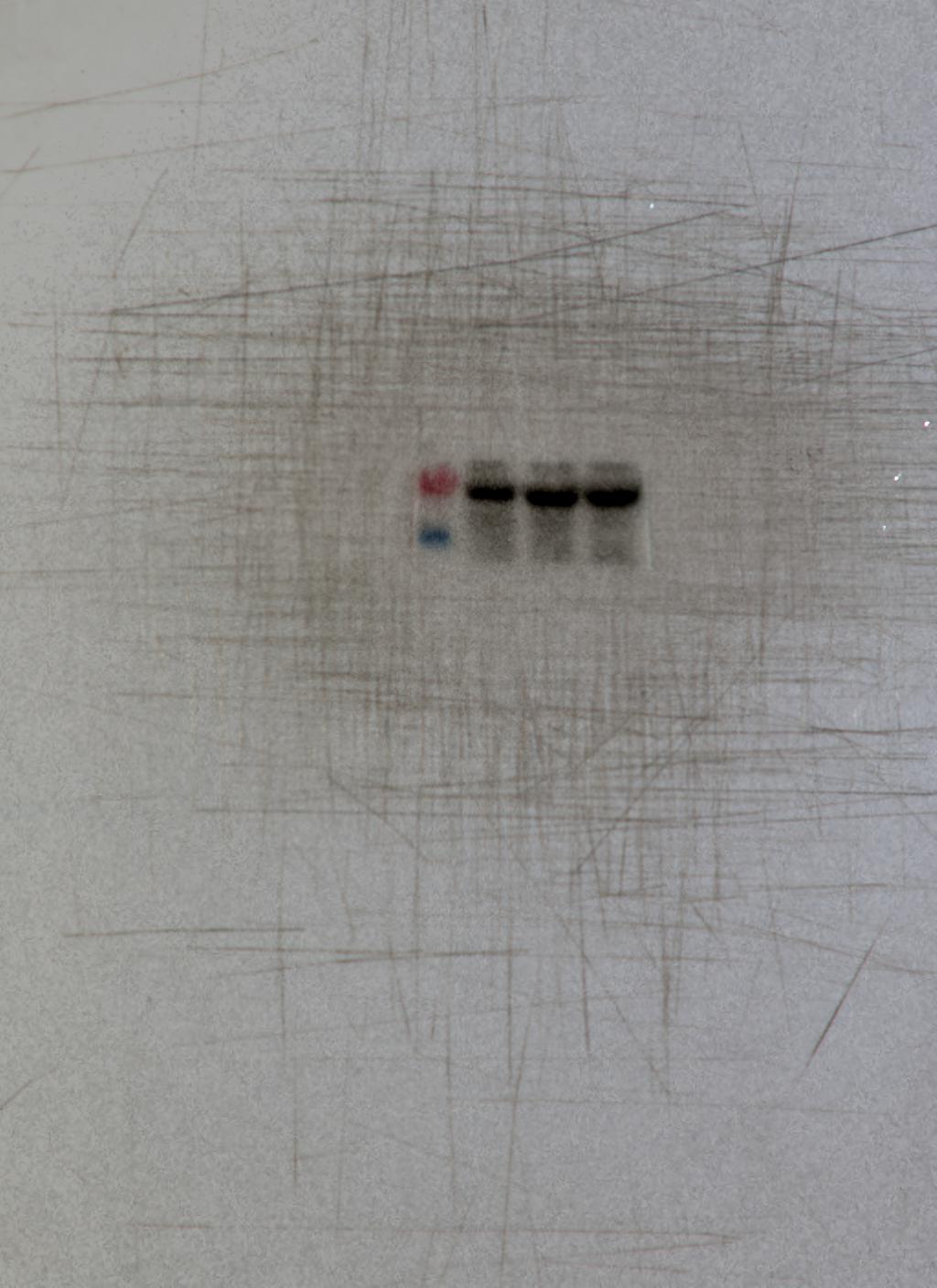


p-p65


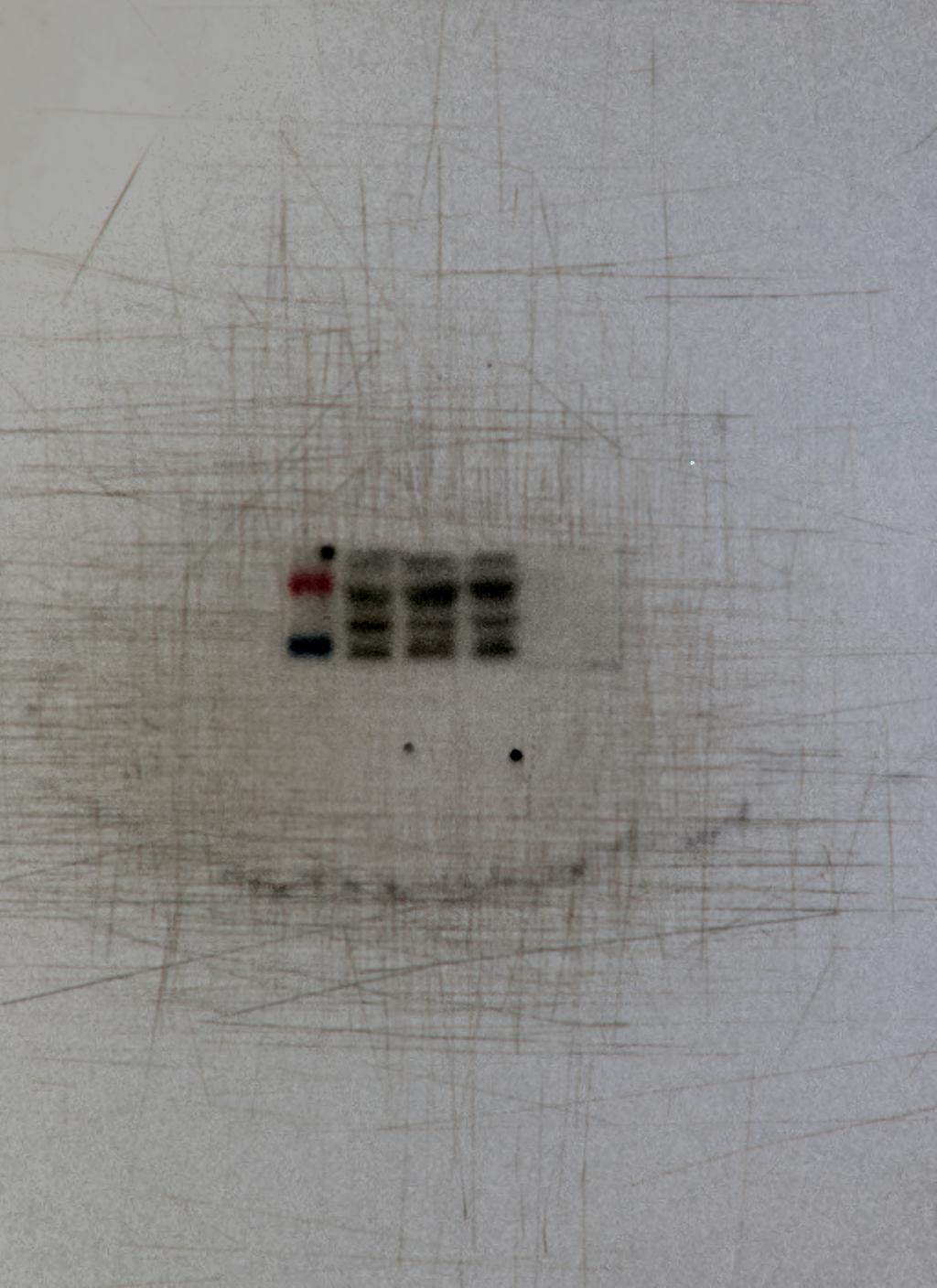


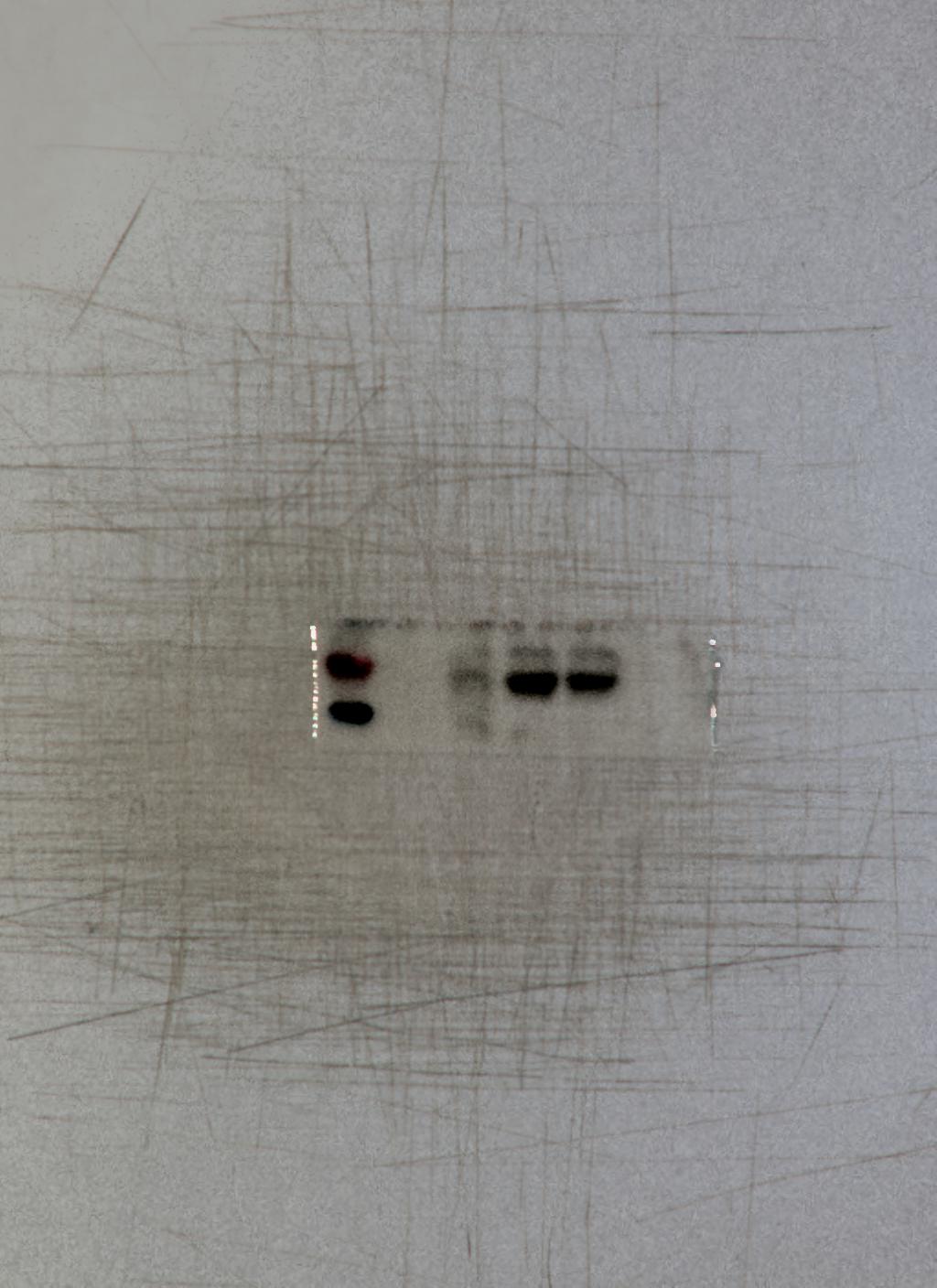


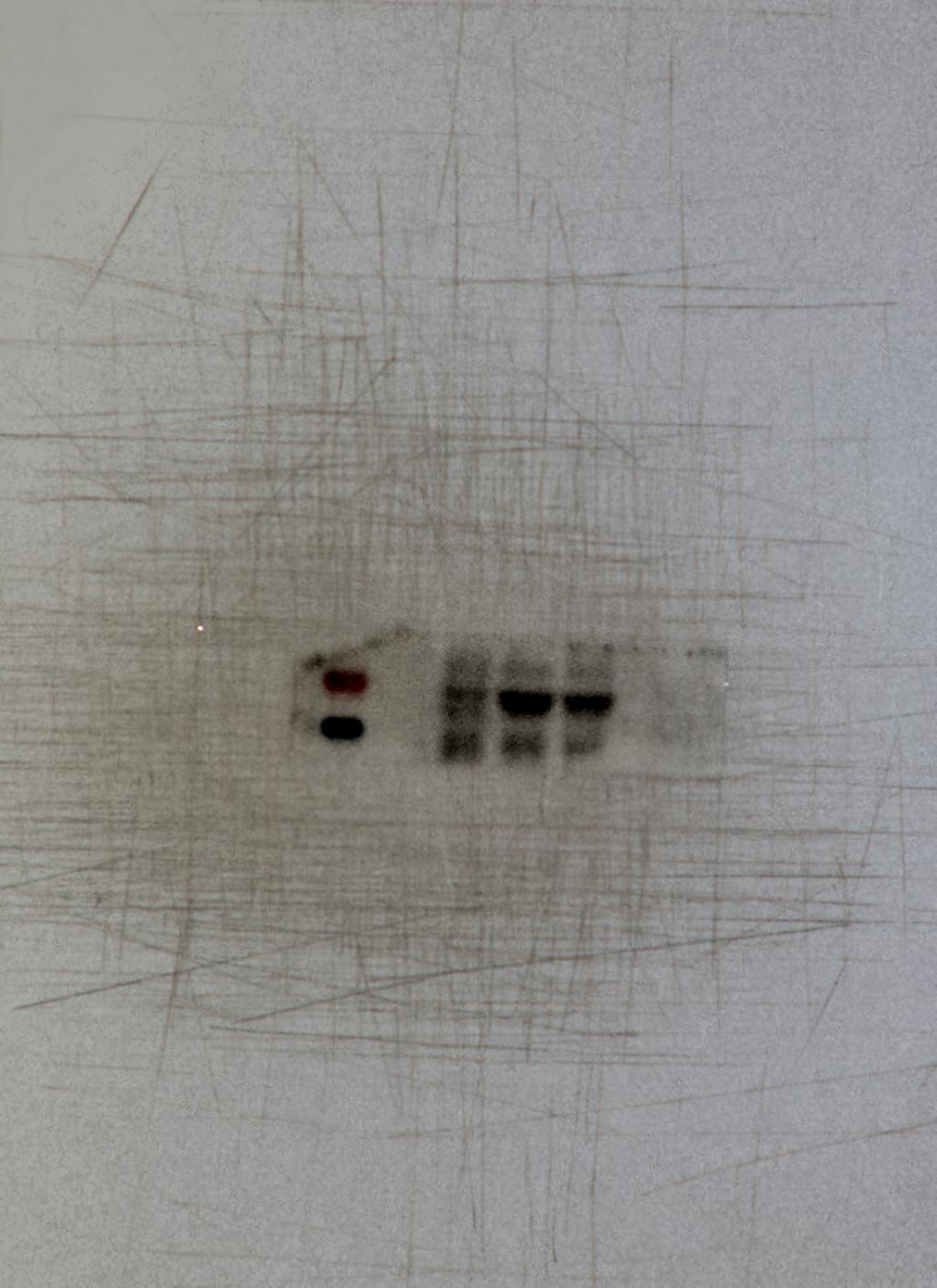


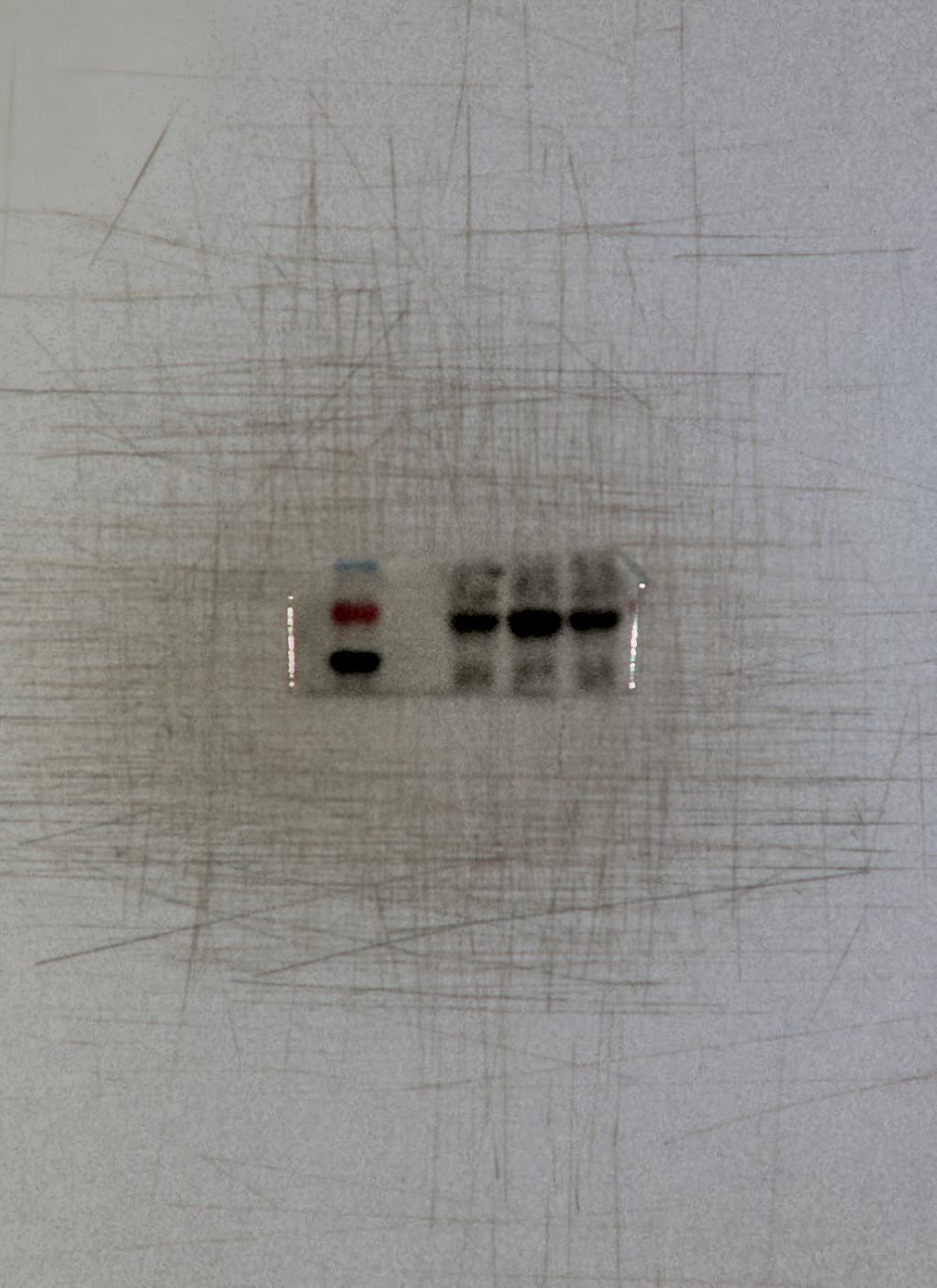


IRF7


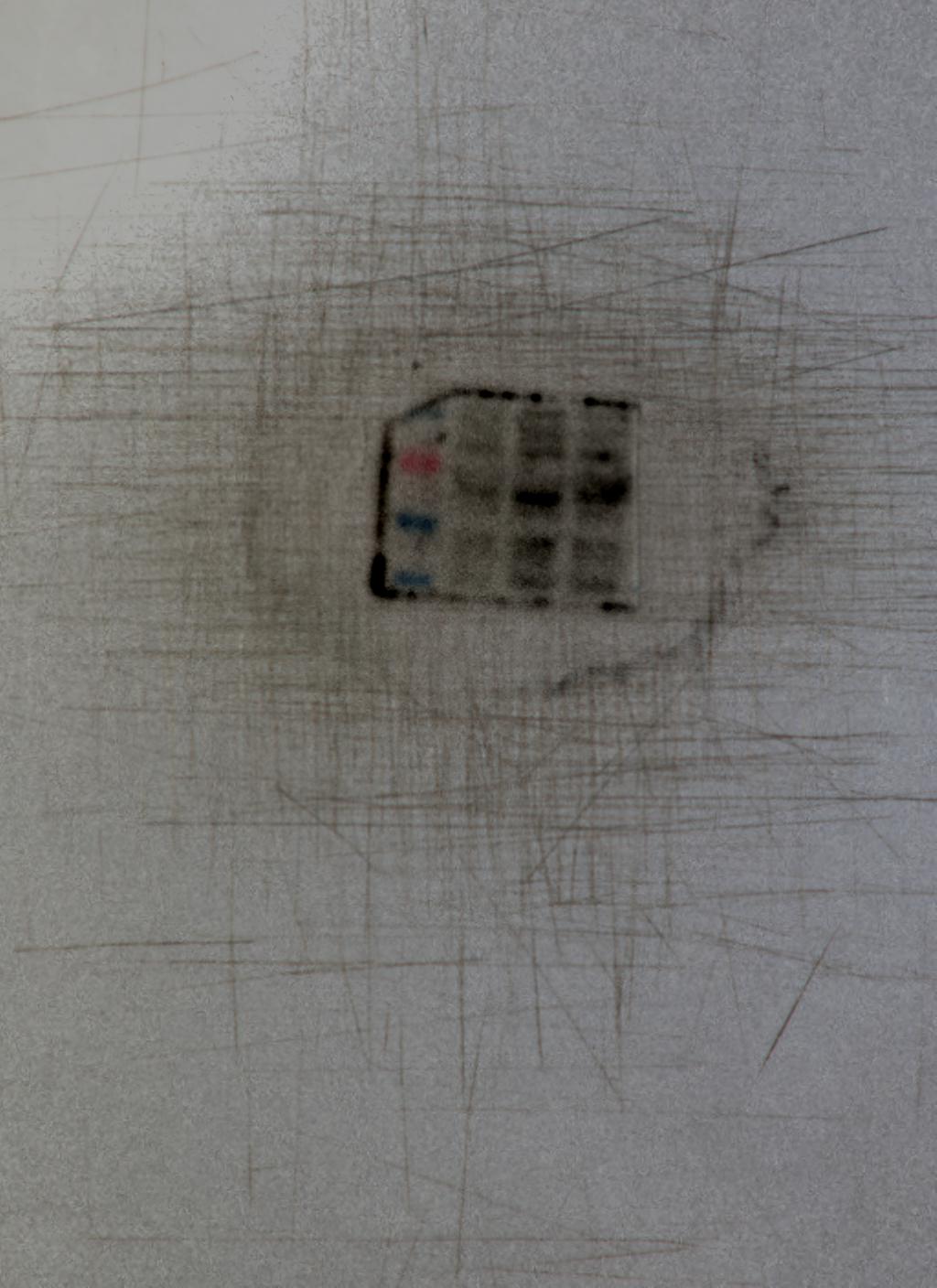


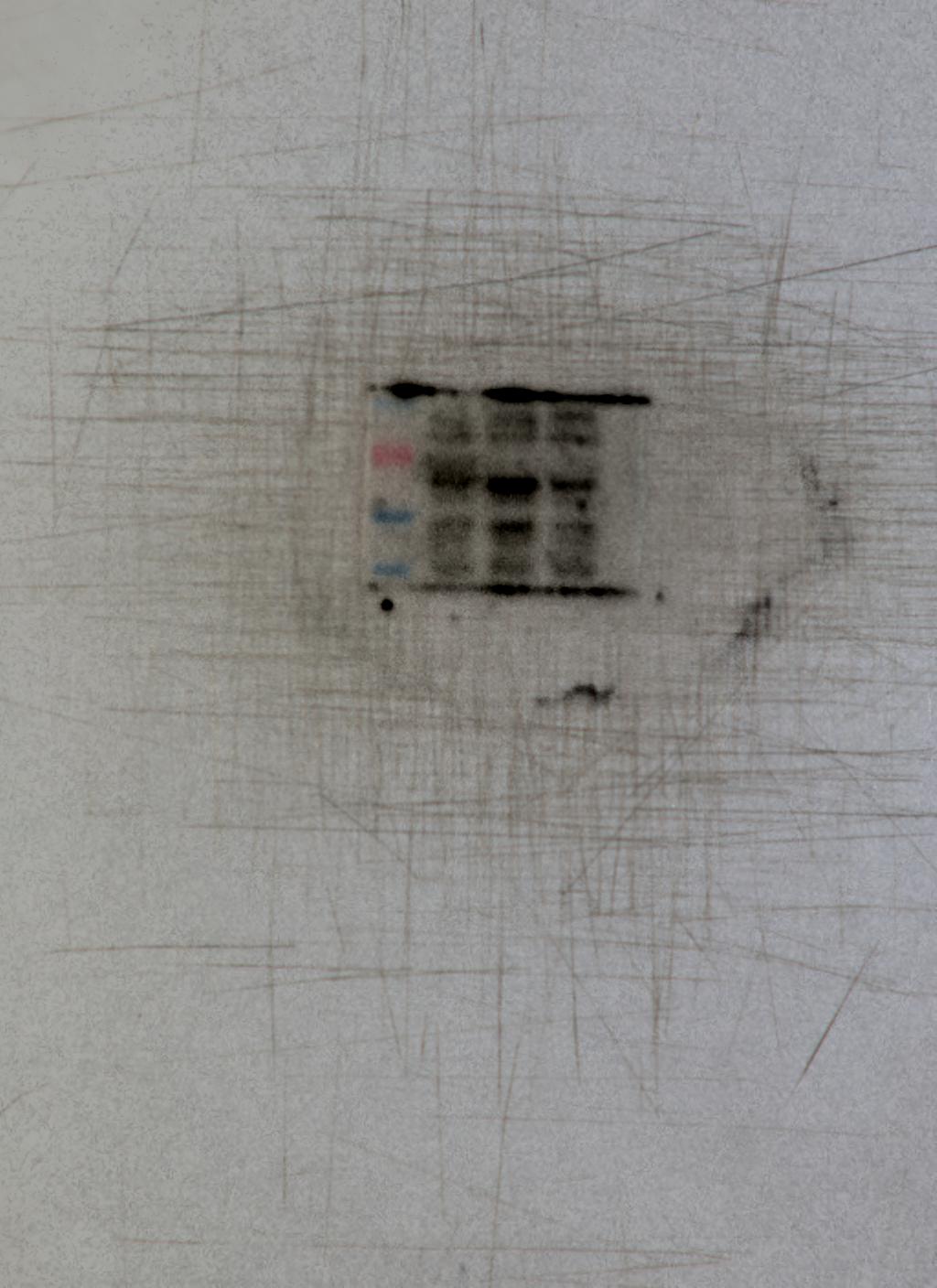


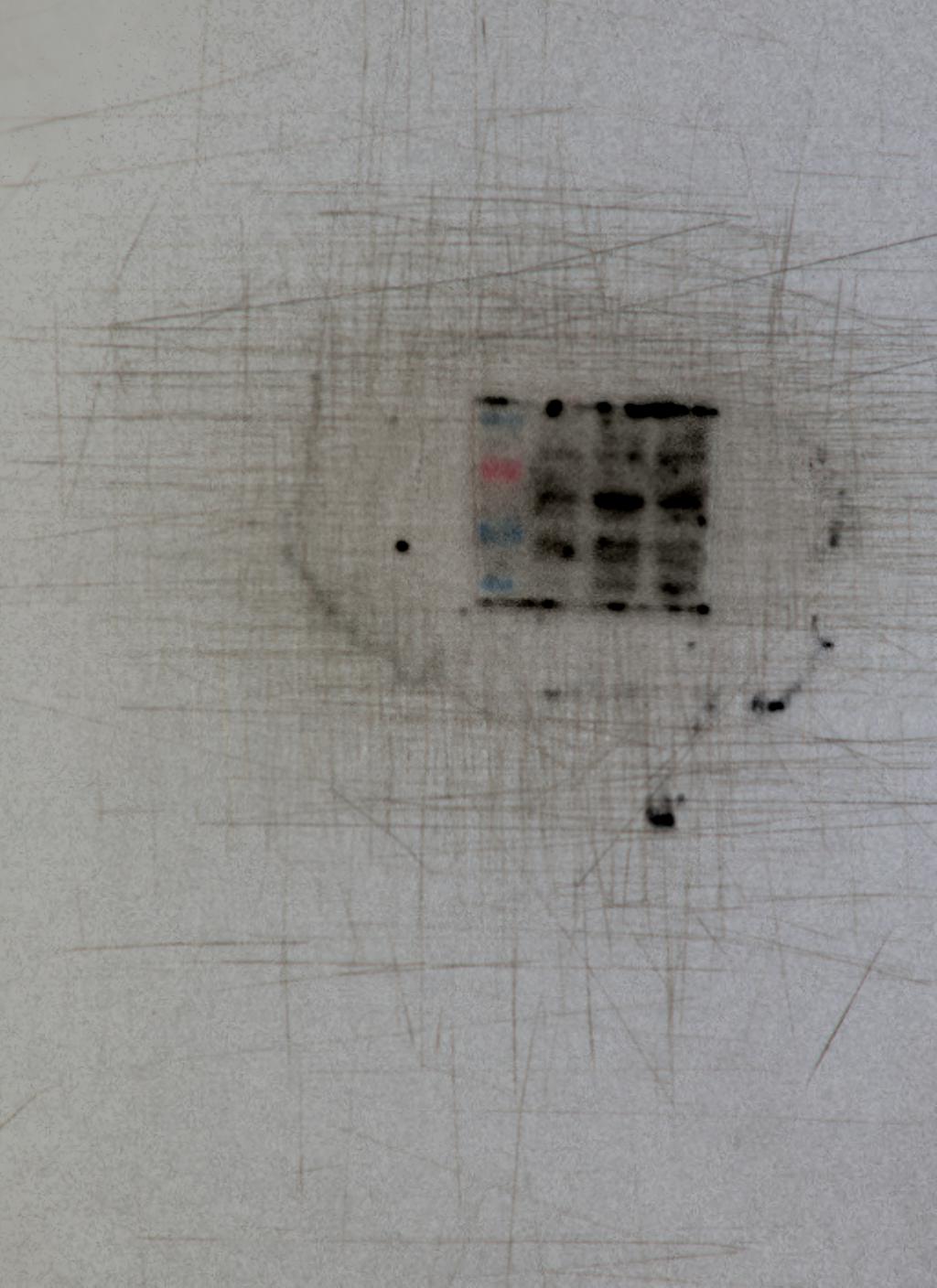


Figure 6A

Beta-ACTIN


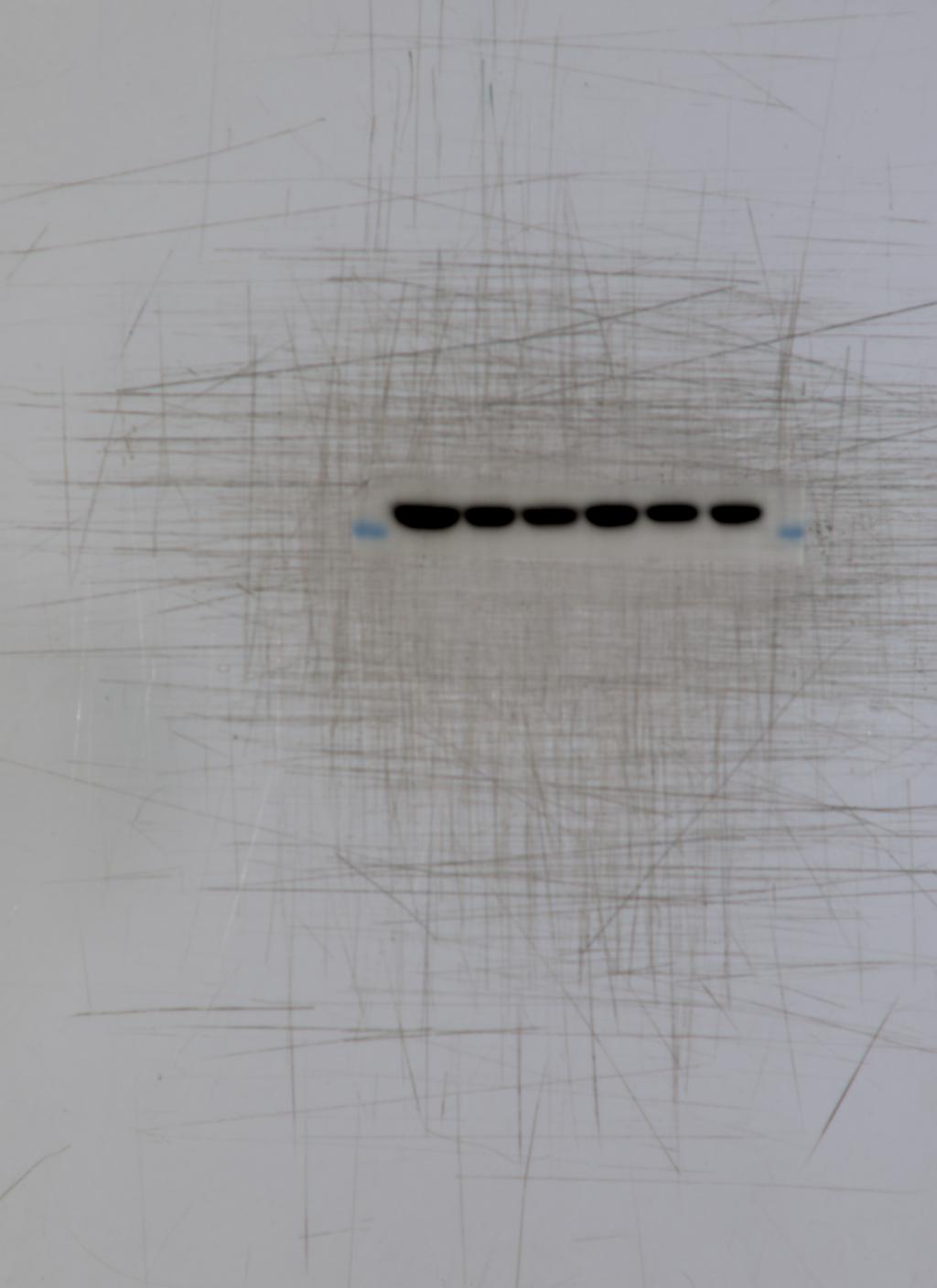

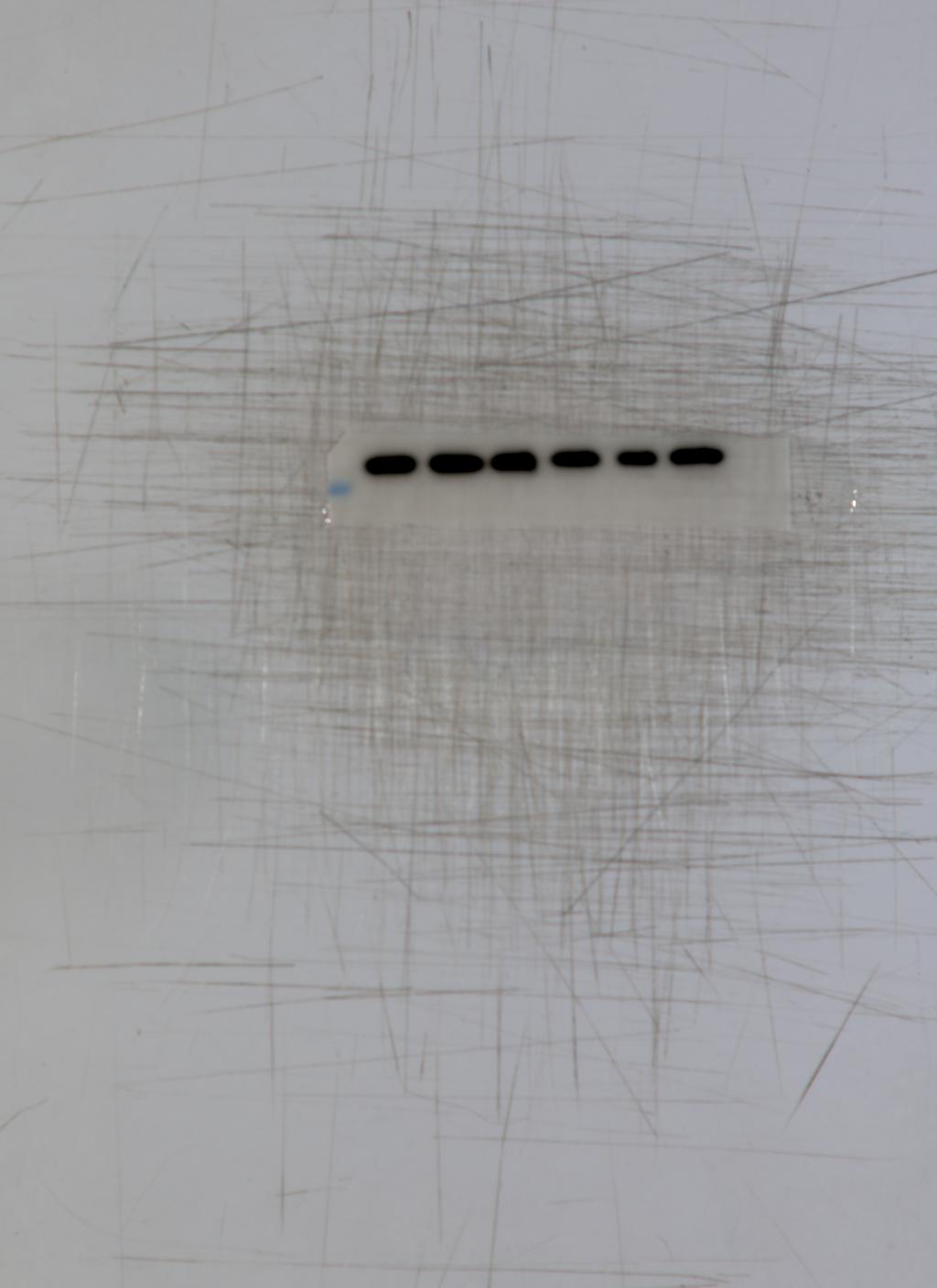


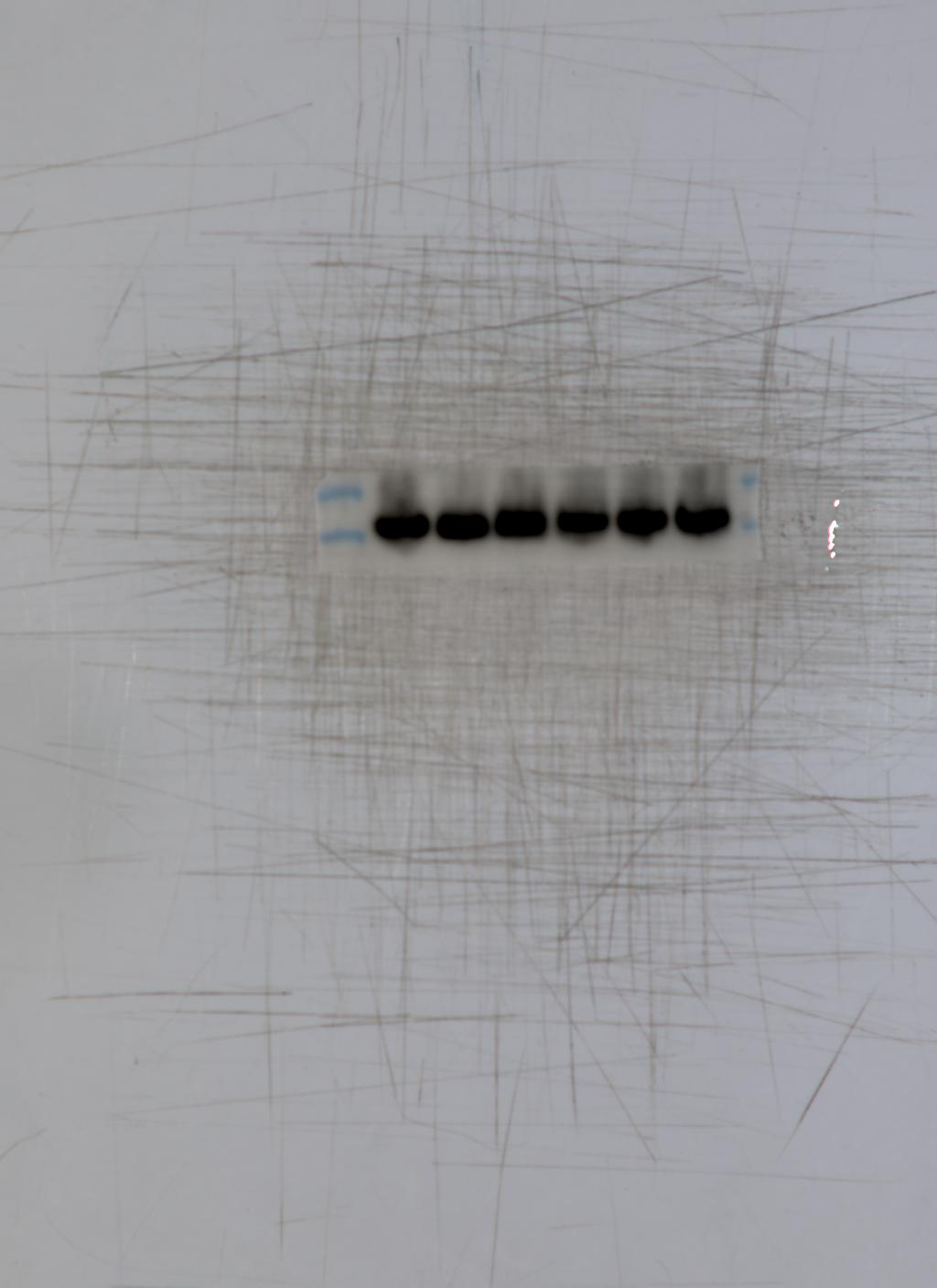


IGFBP5


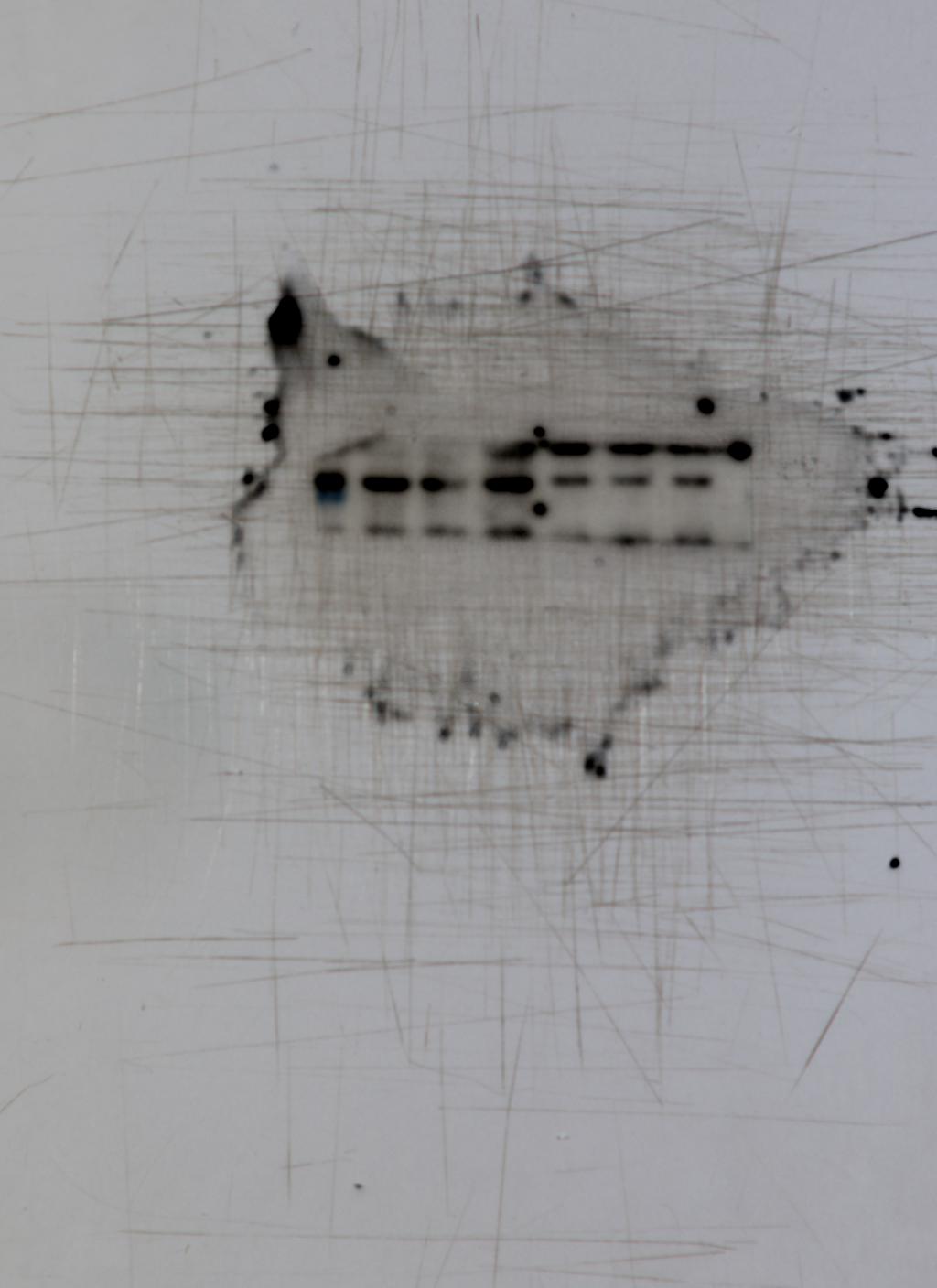


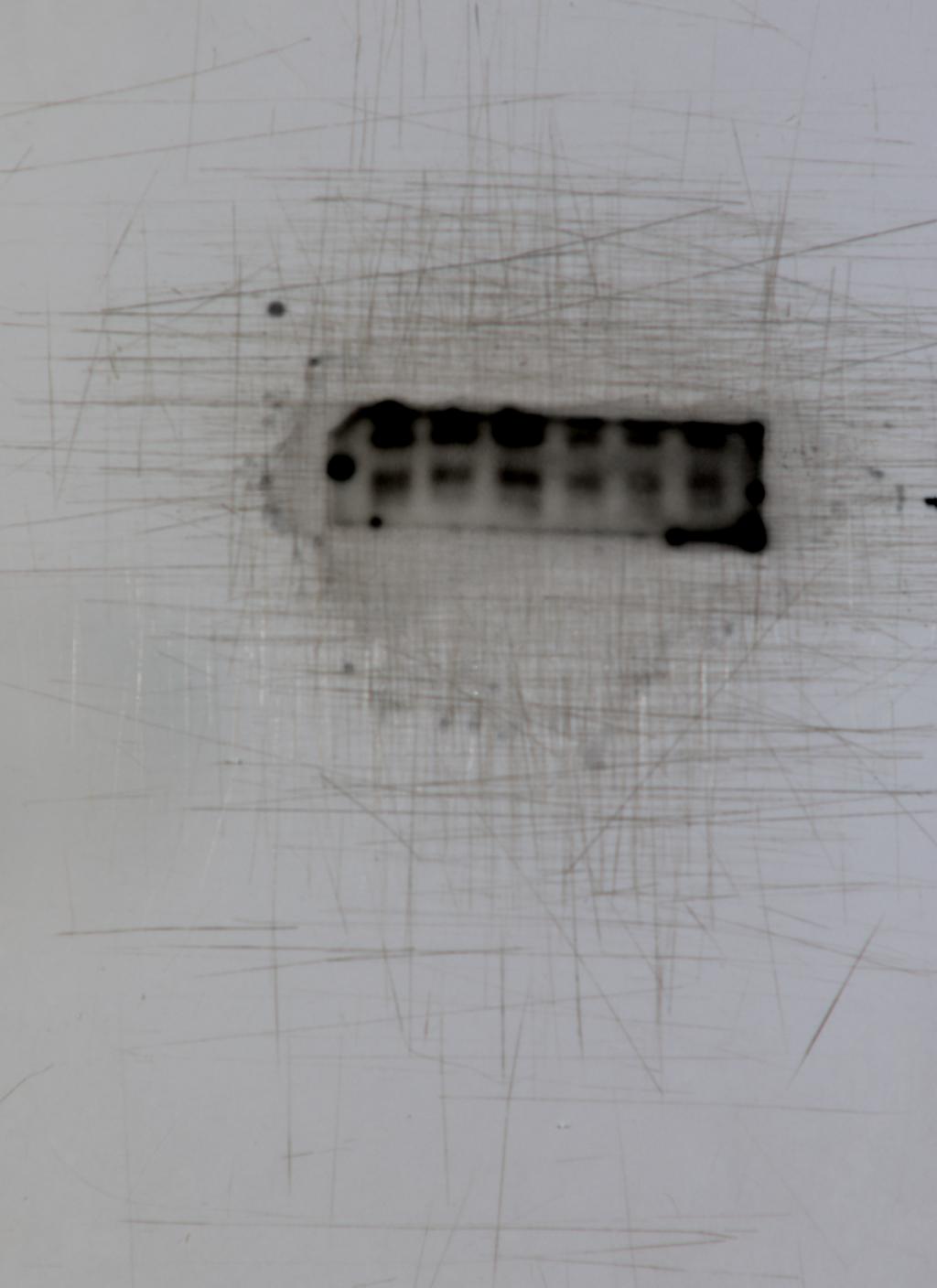


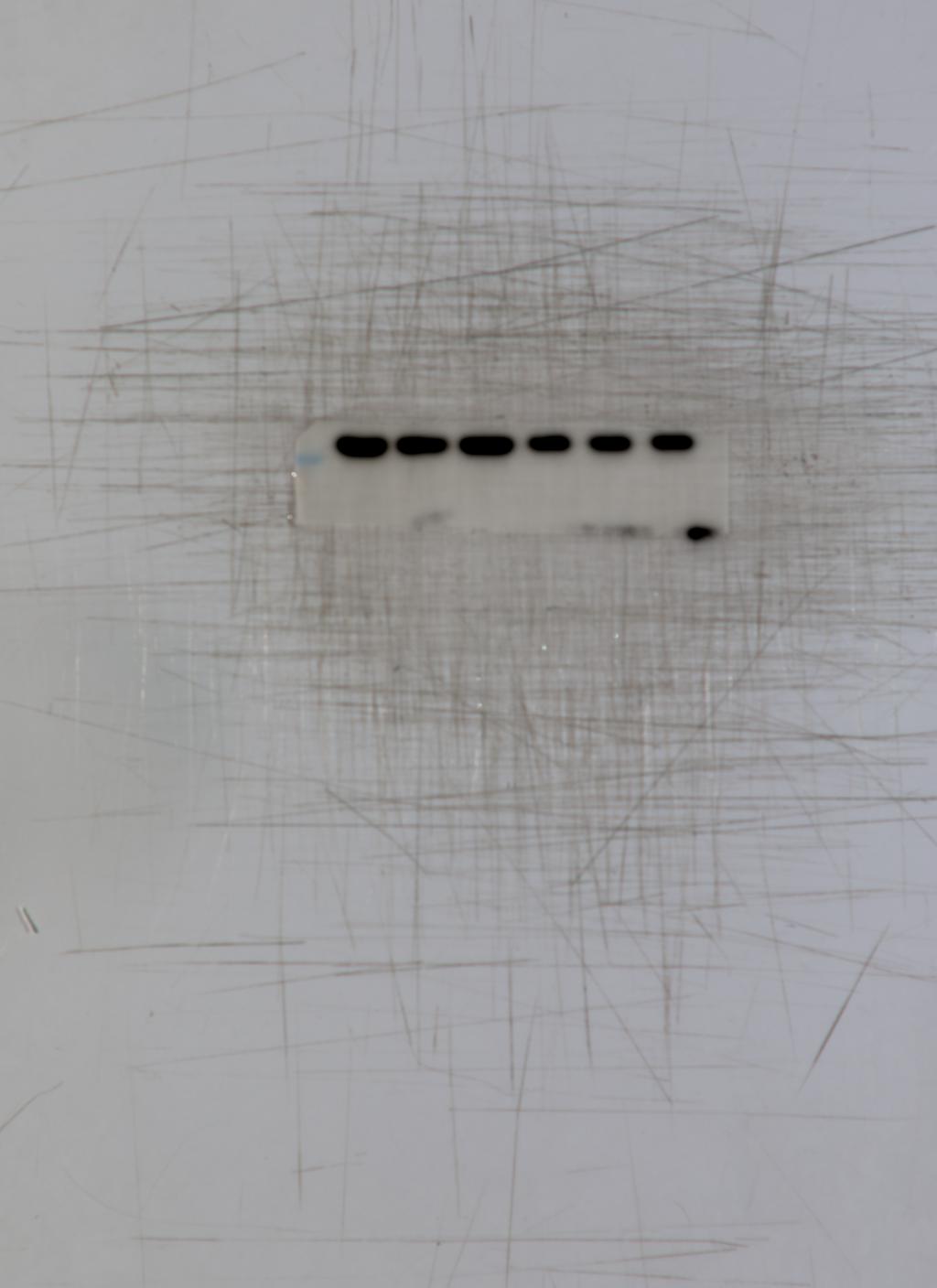


Figure 6D

Beta-ACTIN


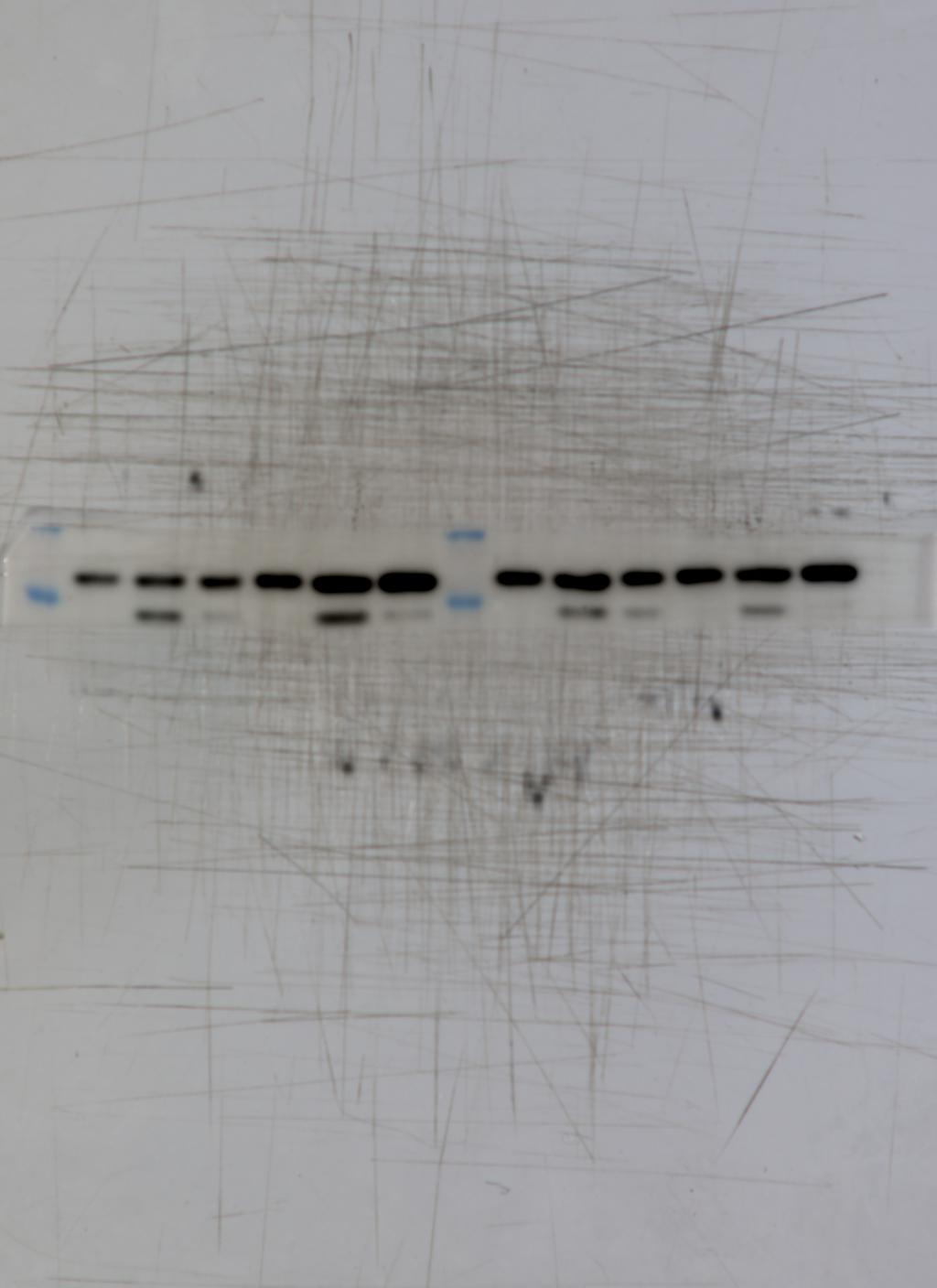


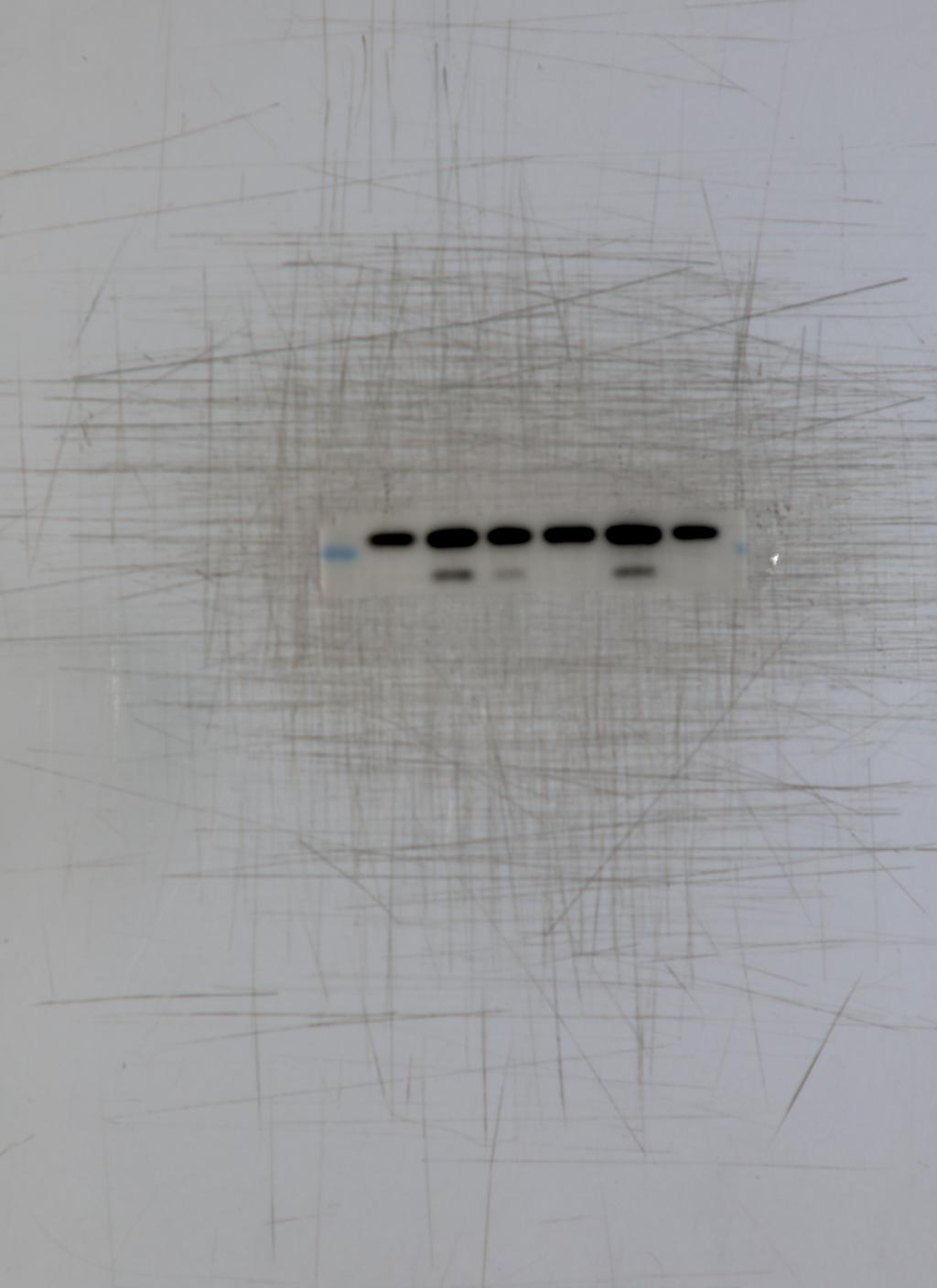


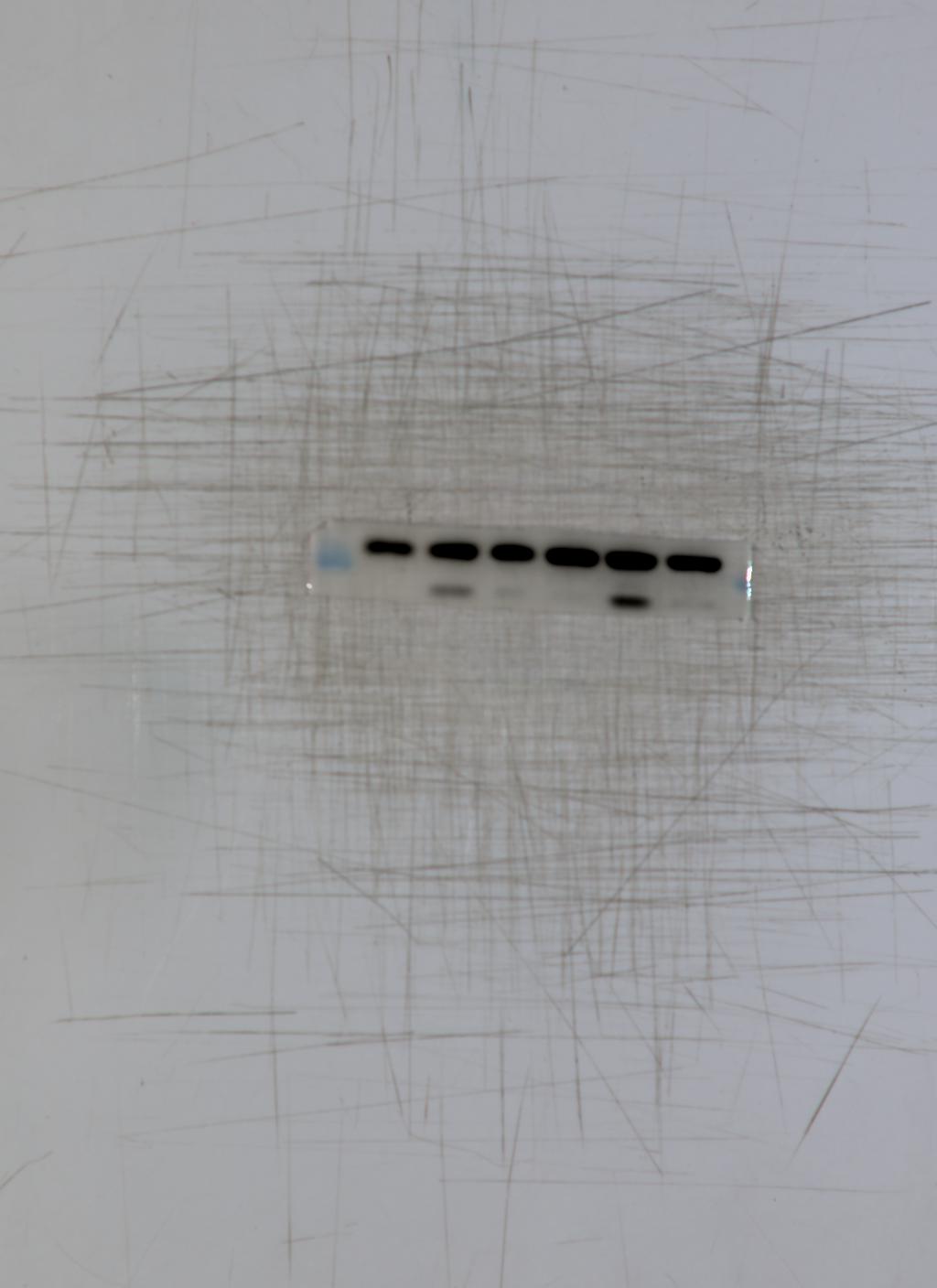


IGFBP5


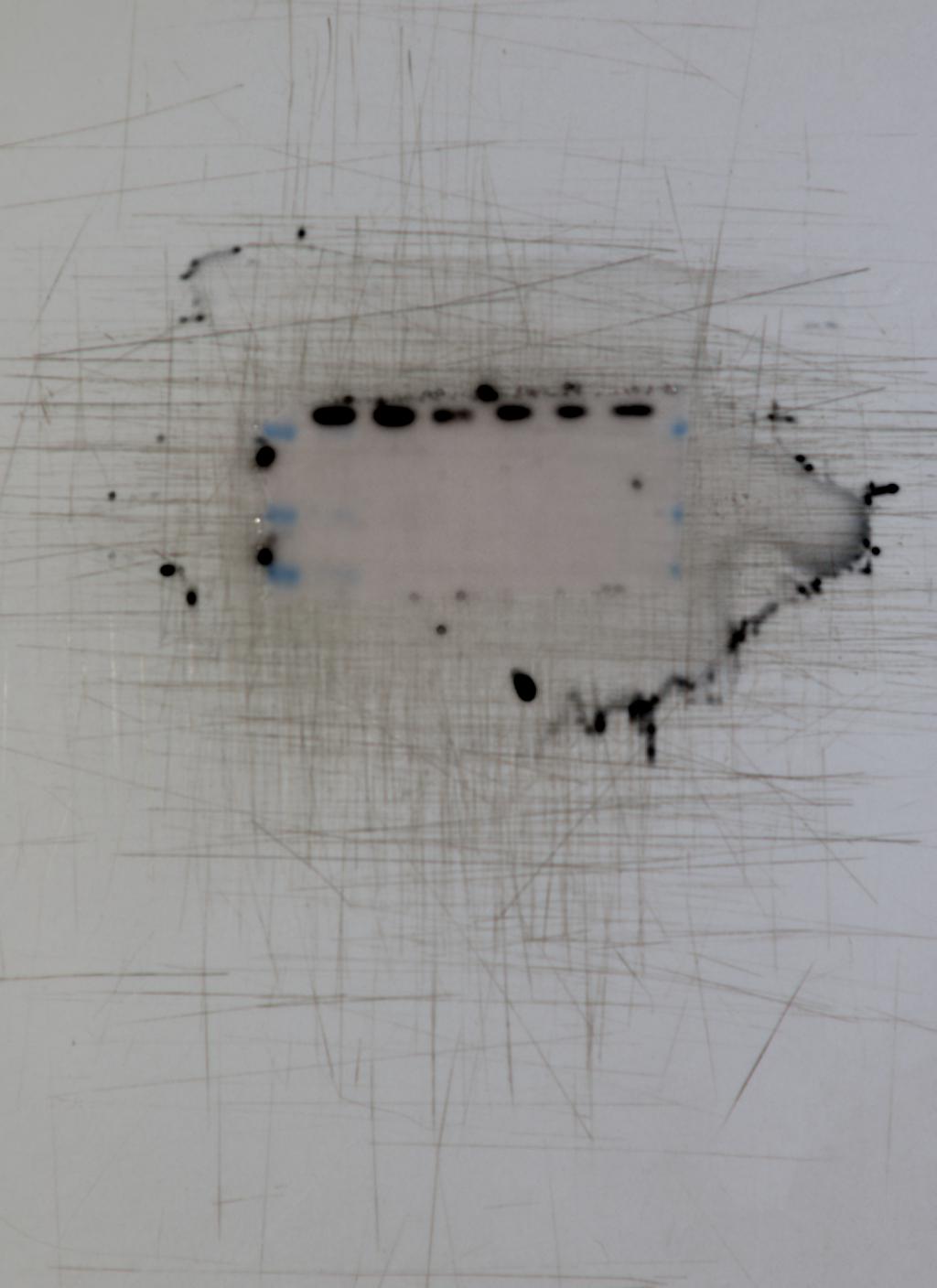


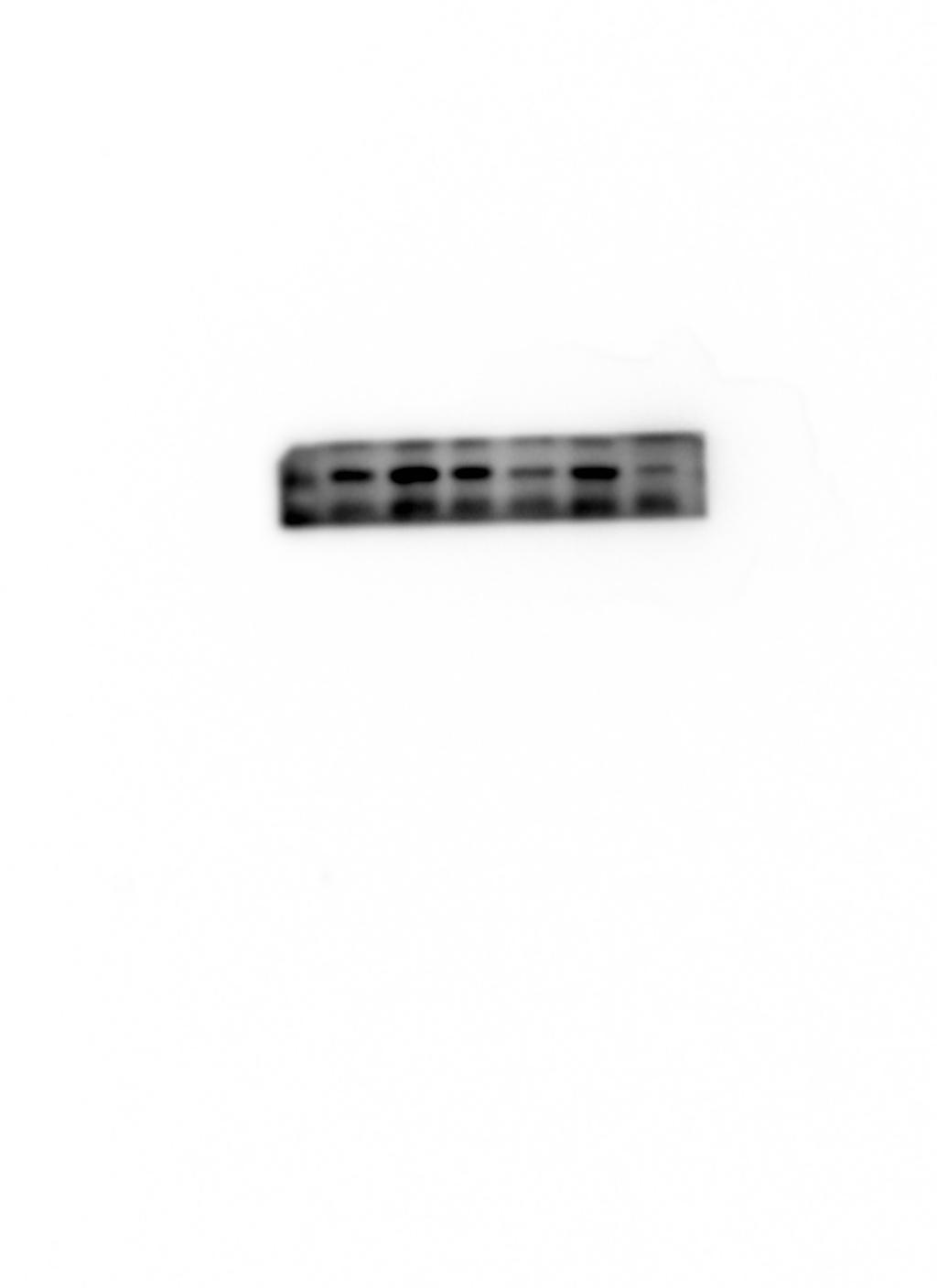


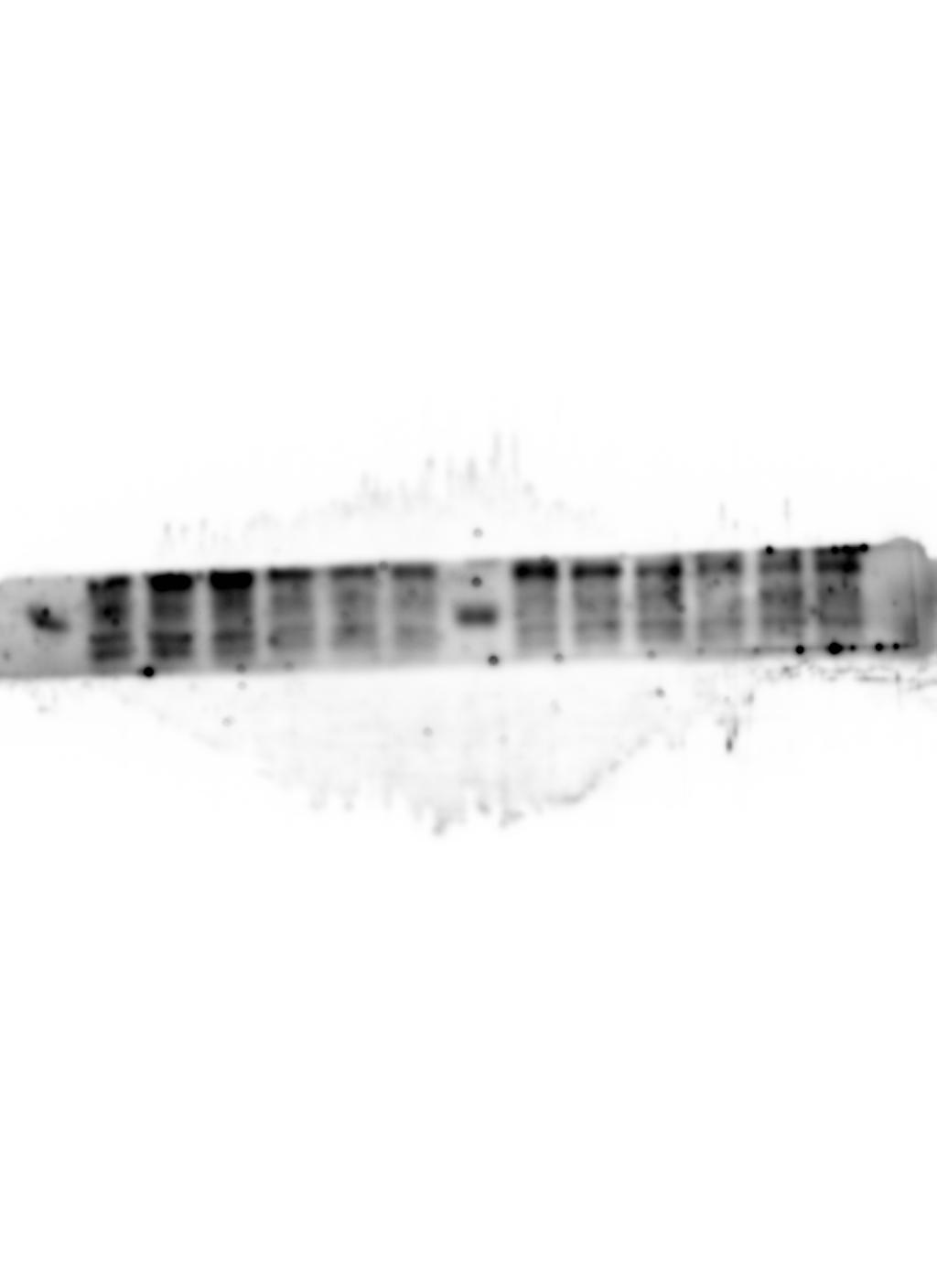

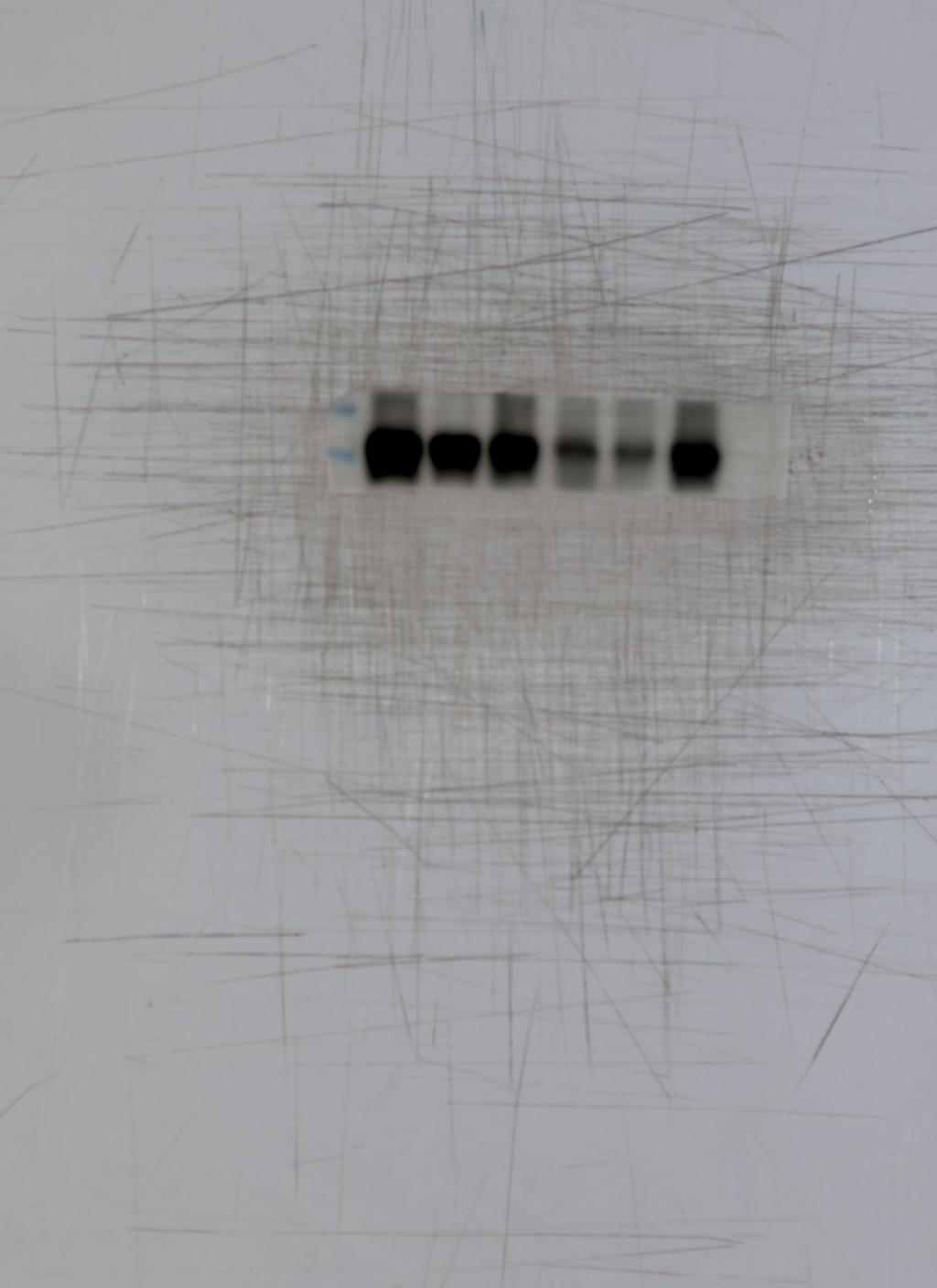


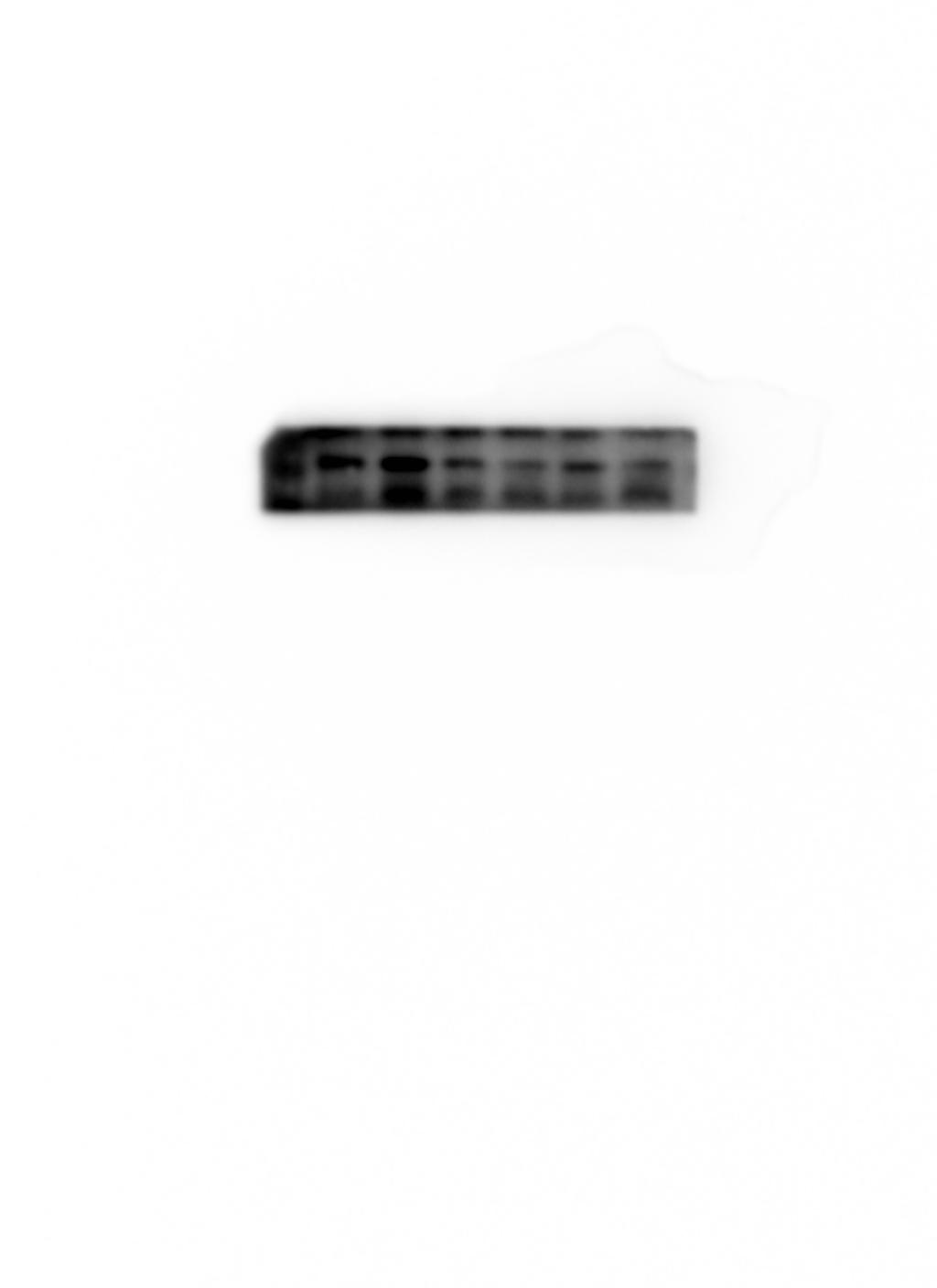


FIGURE 8E

ACTIN


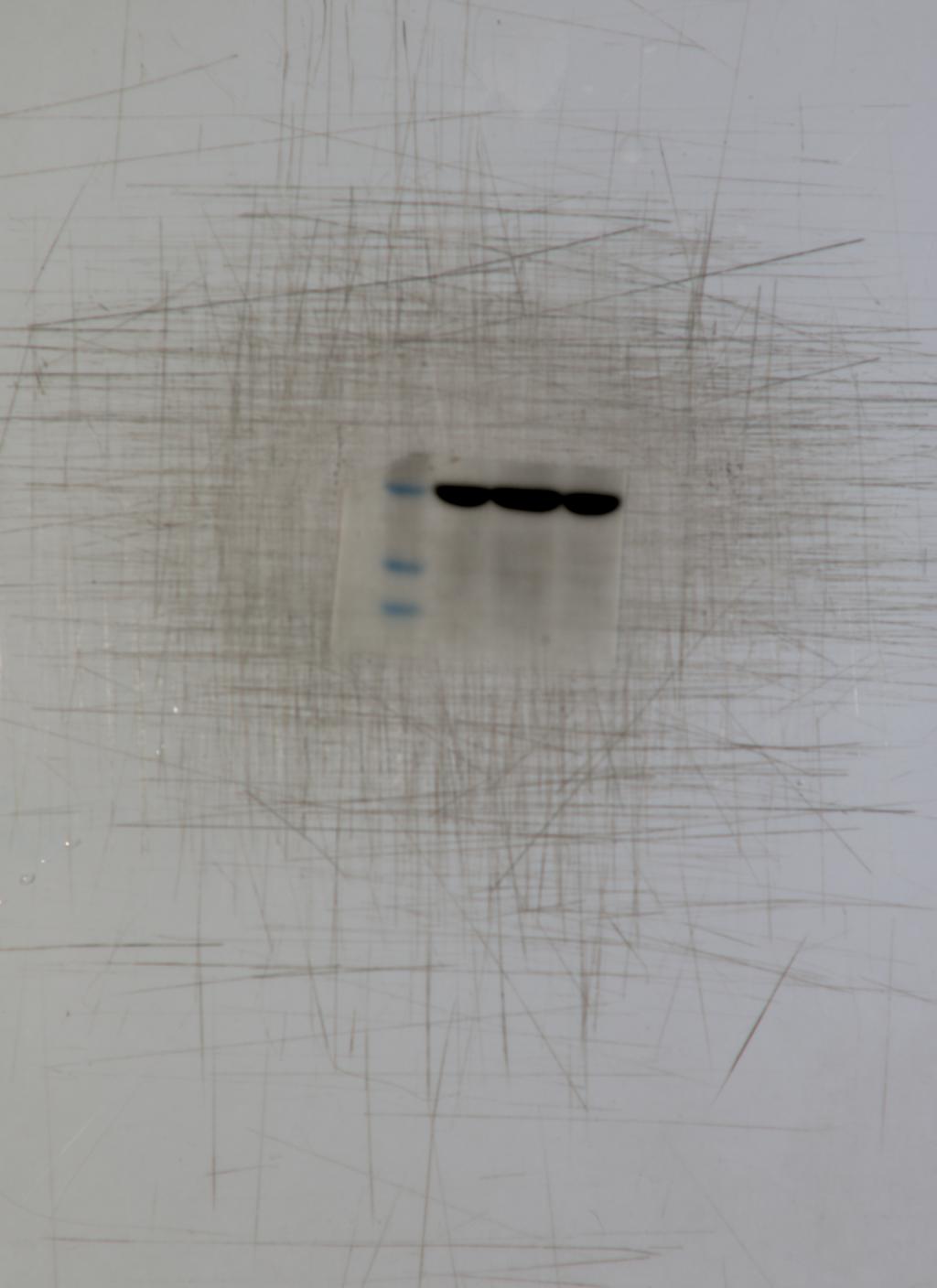


P65

P-P65
