# Supplementary figures and images for: Interaction with IGF1 overrides ANXA2-mediated anti-inflammatory functions of IGFBP5 in vivo
Source: Front Immunol. 2025 Jan 10;15:1539317. doi: 10.3389/fimmu.2024.1539317 (PMC11757107; doi:10.3389/fimmu.2024.1539317)

Figure 2

NORMAL CD86


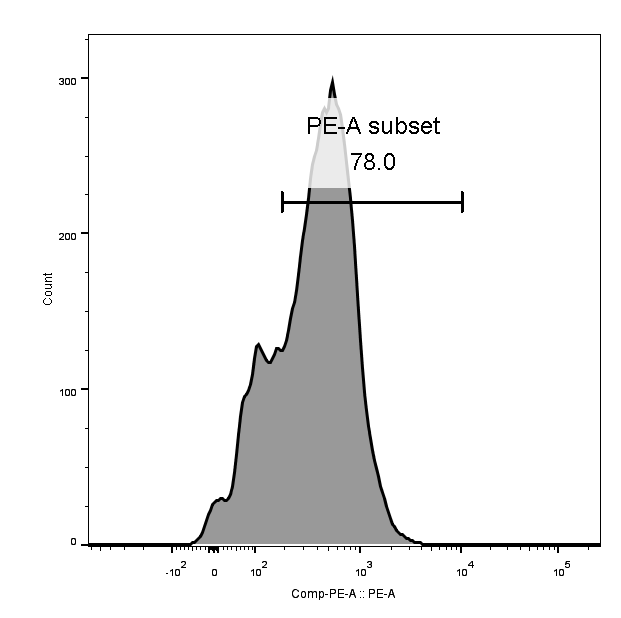

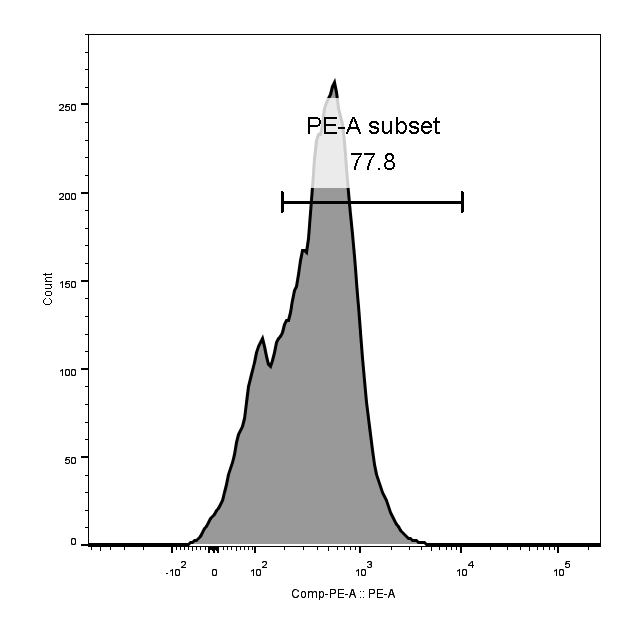

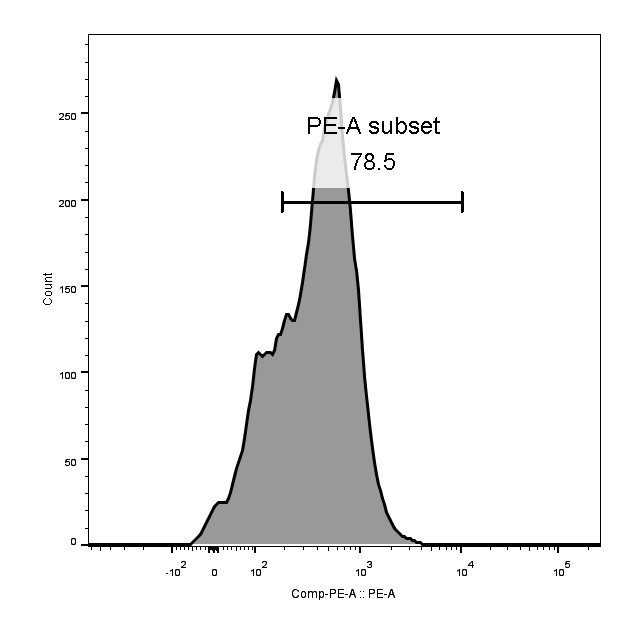

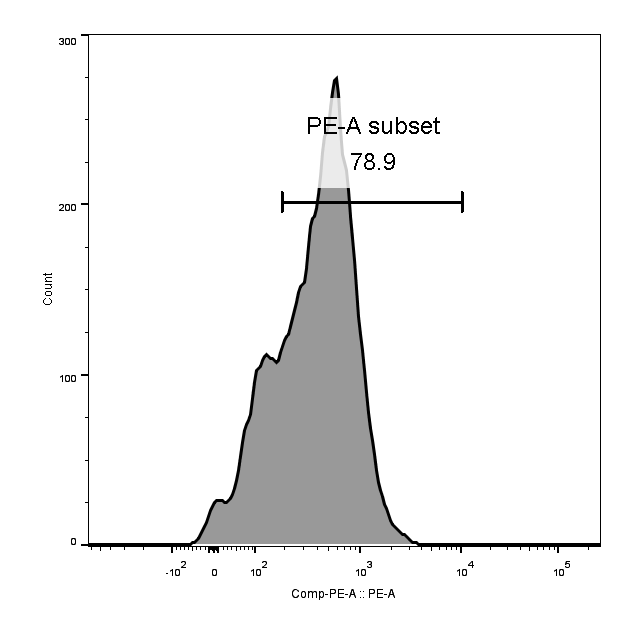


Si-IGFBP5 CD86


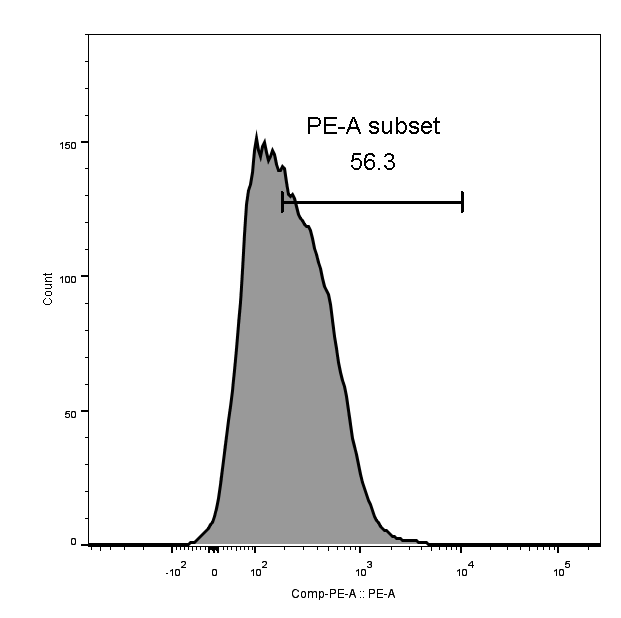

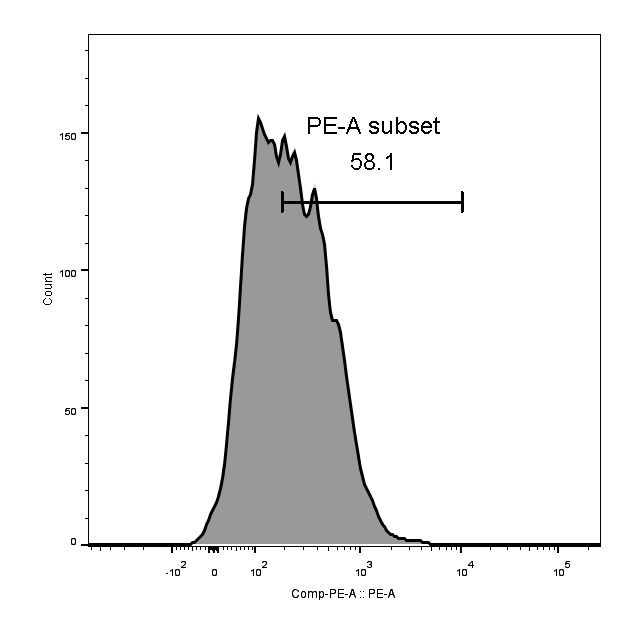

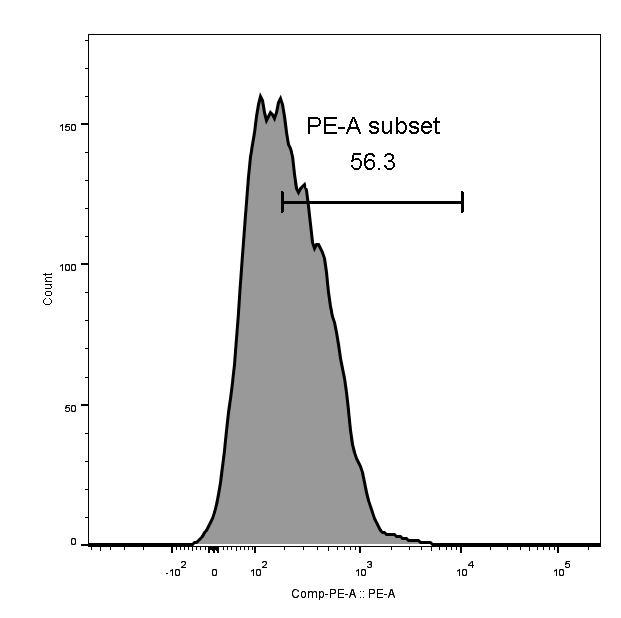


NORMAL CD206


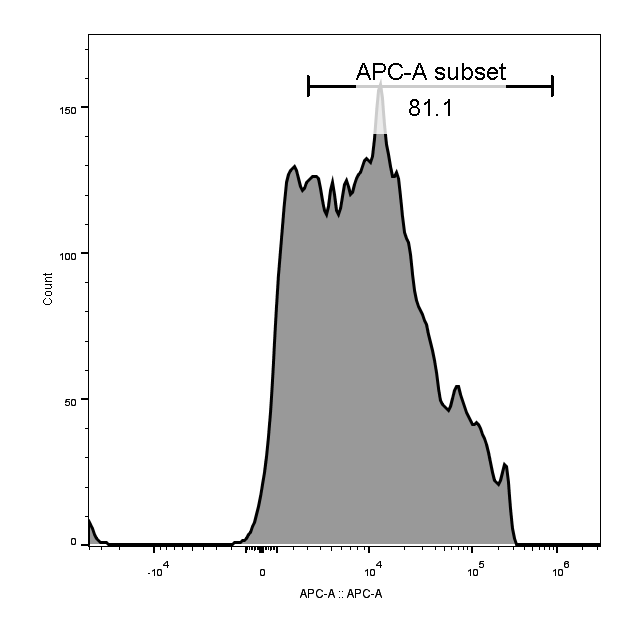

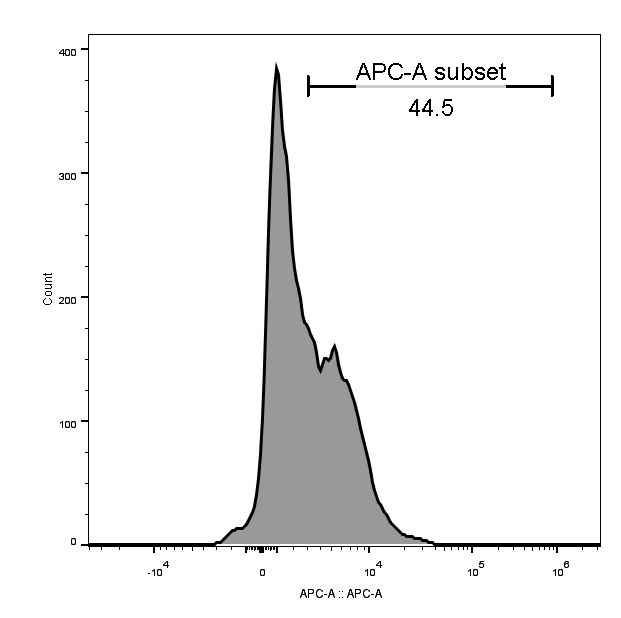

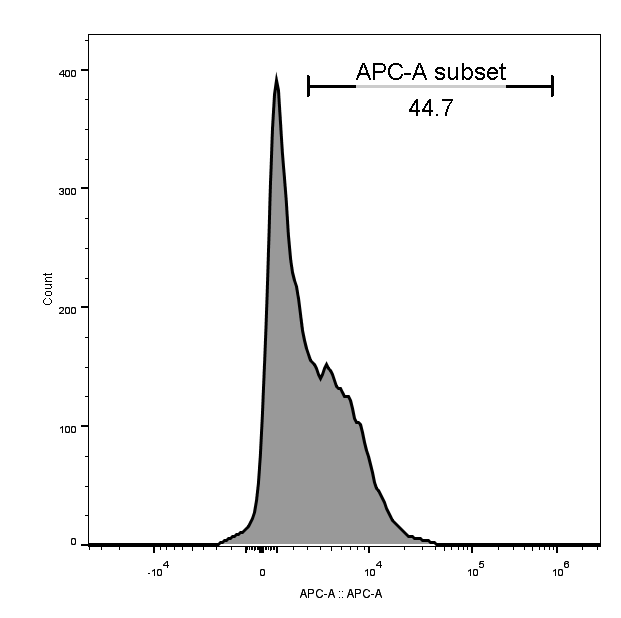

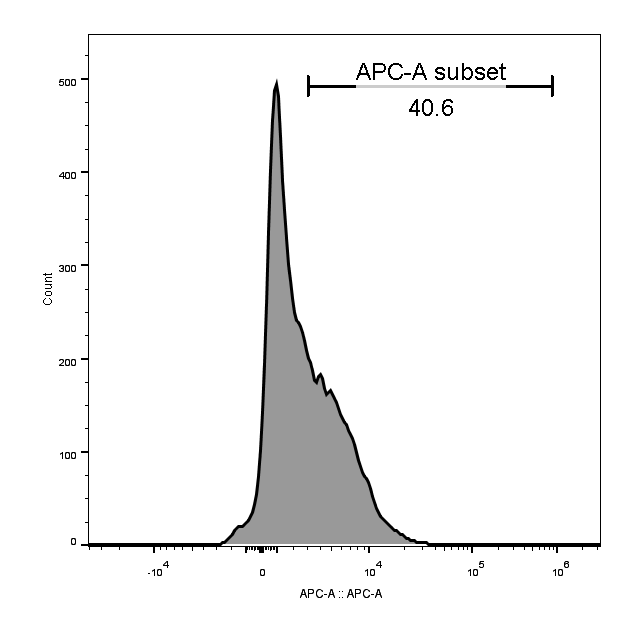


Si-IGFBP5 CD206


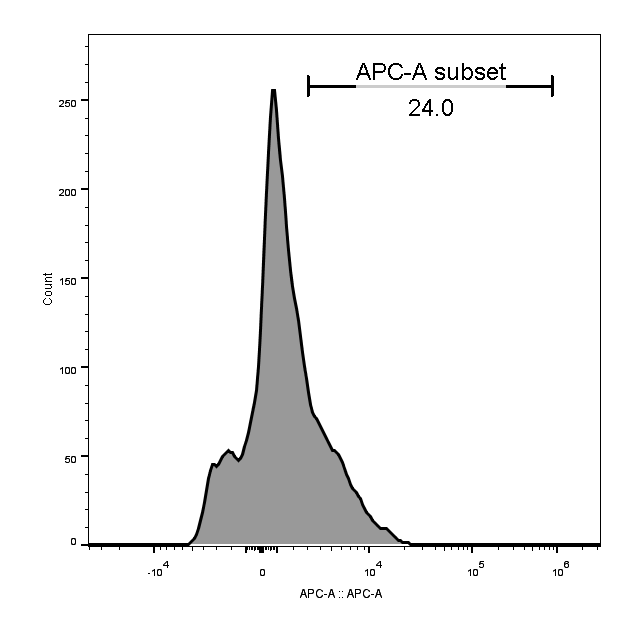

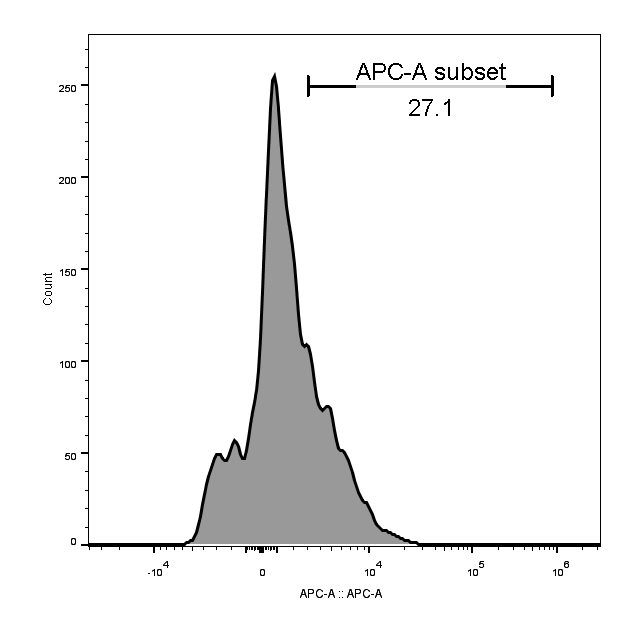

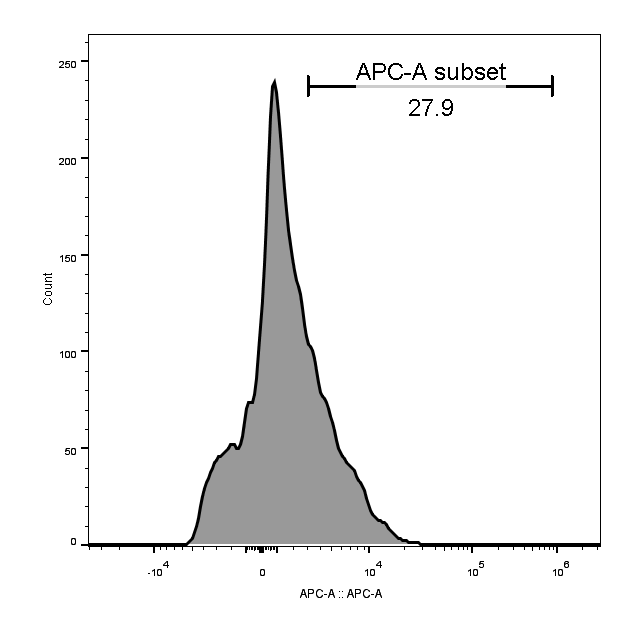


Figure 7

Analysis strategy


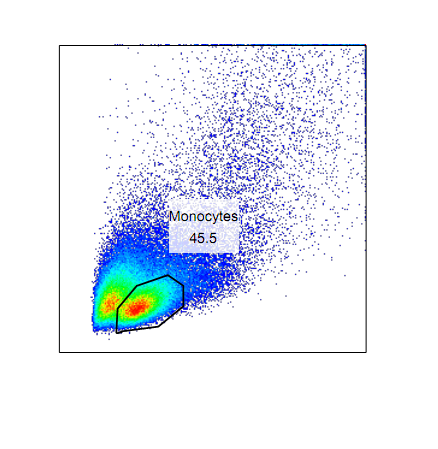


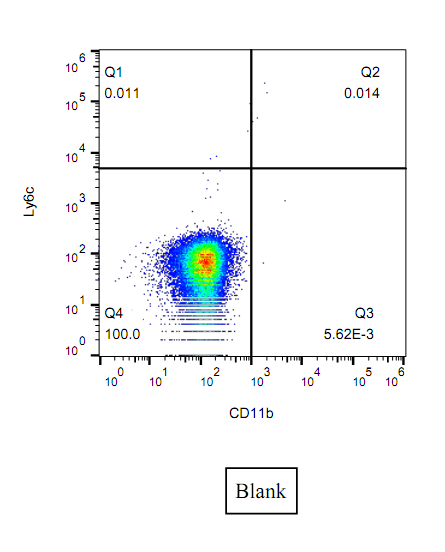

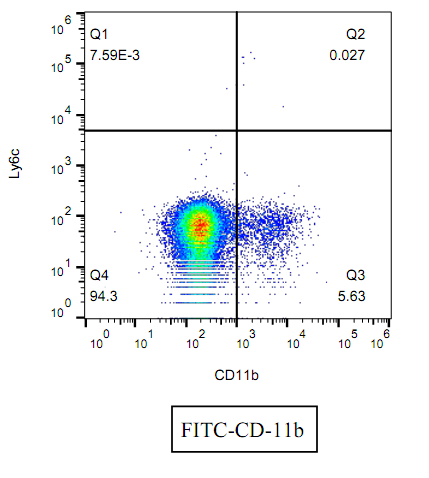

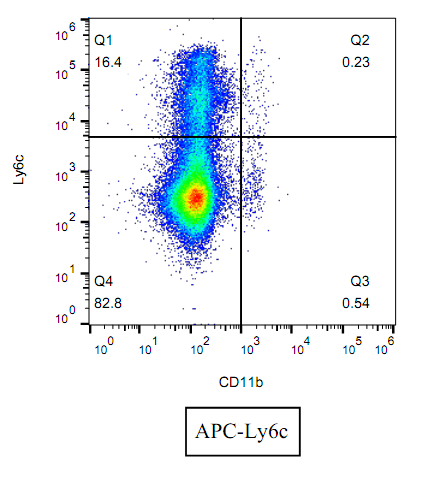


Normal


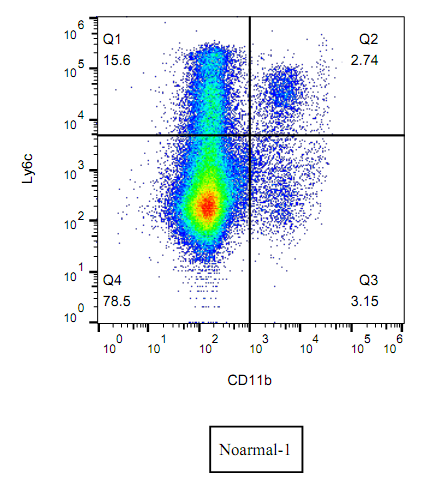

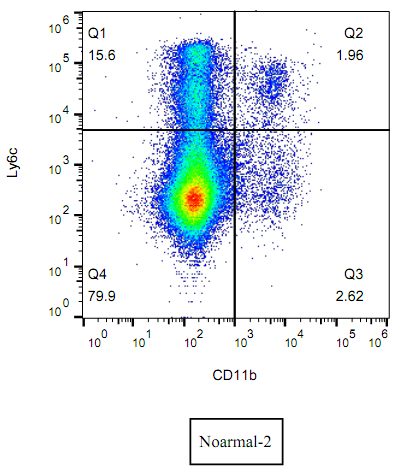

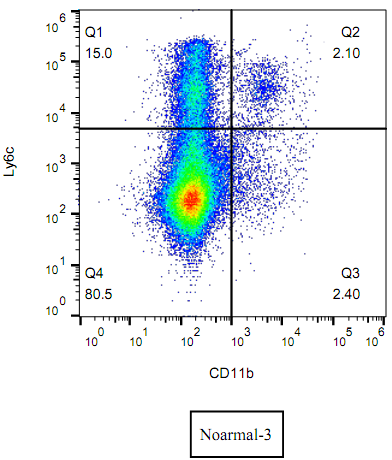

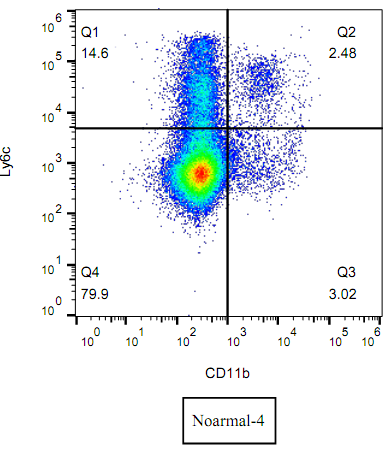

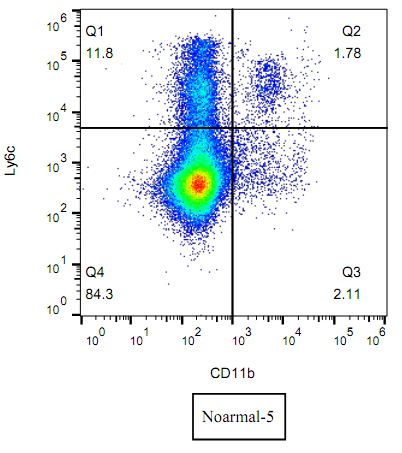


ALI


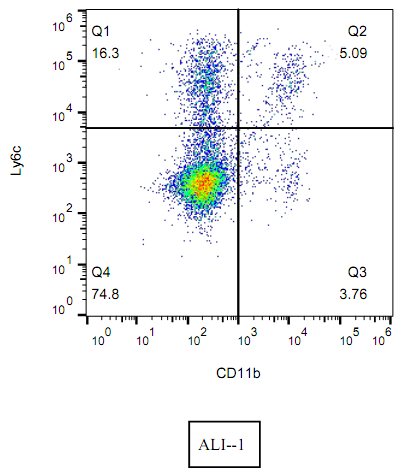

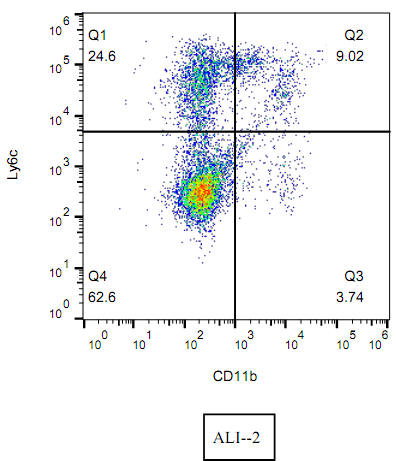

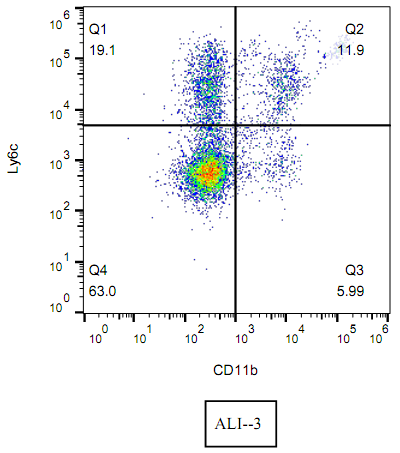

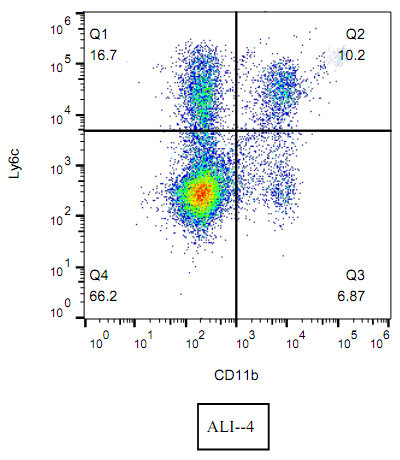

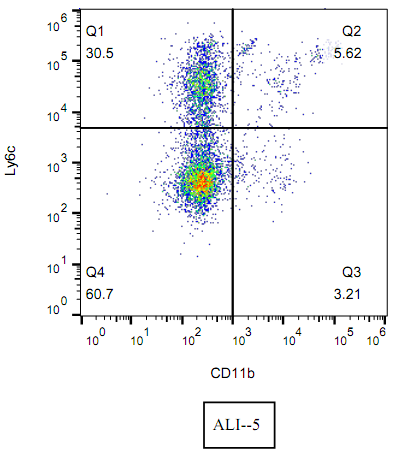


ALI+IGFBP5


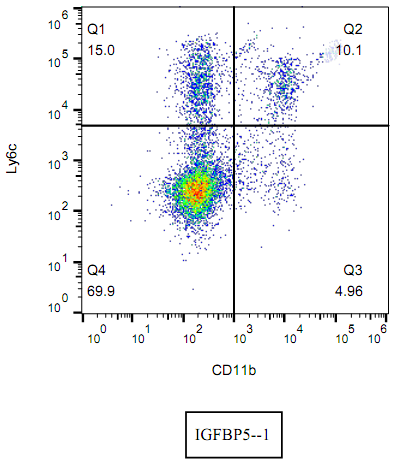


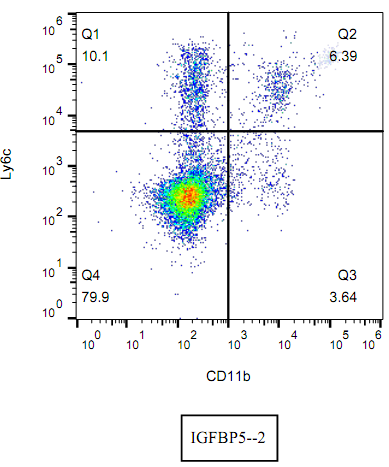

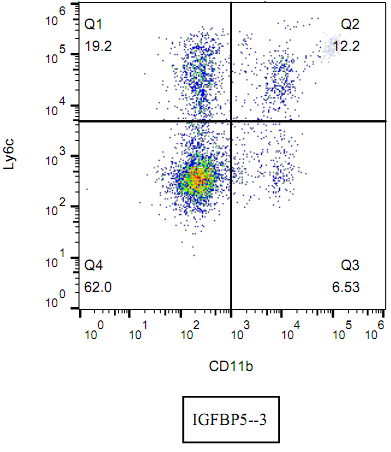

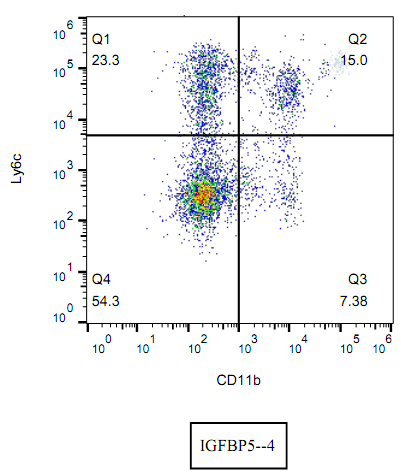

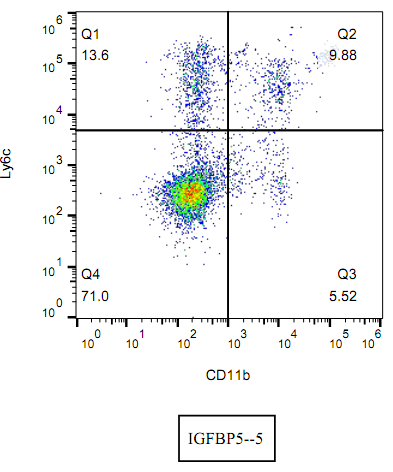

Supplement: Supplementary file 5 [file Table5.docx]
