# Supplementary material for: Interaction with IGF1 overrides ANXA2-mediated anti-inflammatory functions of IGFBP5 in vivo
Source: Front Immunol. 2025 Jan 10;15:1539317. doi: 10.3389/fimmu.2024.1539317 (PMC11757107; doi:10.3389/fimmu.2024.1539317)
Supplement: Supplementary file 6 [file DataSheet1.pdf]

frontiers

The International Society for the Advancement of Cytometry (ISAC) has highlighted the importance of including comprehensive methodological information to ensure data reproducibility and reliability. In line with this, Frontiers in Immunology now requires authors to submit a checklist for manuscripts that involve flow or mass cytometry. This checklist helps standardize the reporting process, improving the quality and transparency of published data. By doing so, we support scientific progress, making it easier for other researchers to replicate and validate experiments.

This form should be submitted with any manuscripts using flow or mass cytometry.

Sample/specimen/material description

- [ ] Total blood
- [ ] PBMCs
- [ ] Organ digests

Other Human leukemia THP-1 monocytes

Did the samples suffer any treatment before or after incubation with the antibodies?

- [ ] Drug ______________________________
- [x] Cell permeabilization ______________________
- [ ] Dye ________________________________
- [ ] Propidium iodine
- [ ] Not applicable

Other si-IGFBP5

Instrument and antibodies

Name of the Cytometer FC500, Beckman

**Antibodies and targets**	**Fluorochrome/ Metal**	**Catalog number/Company**
e.g. anti-CD4	FITC	Cat. XXX/ XXX ltd.
anti-CD86	PE	Cat:305406/Biolegend

anti-CD206	APC	Cat:321110/Biolegend

frontiers

Data analyses

1. Name of the software FlowJo_V10
2. Reference gating strategy in the manuscript or supplementary material

Gating strategy in (eg Figure X) Fig.2E and supplementary material FCM
